# Asymmetric aza-Henry reaction toward trifluoromethyl β-nitroamines and biological investigation of their adamantane-type derivatives

**DOI:** 10.3389/fchem.2024.1398946

**Published:** 2024-05-10

**Authors:** Yi Ren, Mengyuan Du, Ziyu Peng, Changwu Zheng, Gang Zhao

**Affiliations:** ^1^ School of Pharmacy, Shanghai University of Traditional Chinese Medicine, Shanghai, China; ^2^ Laboratory of Fluorine and Nitrogen Chemistry and Advanced Materials, Shanghai Institute of Organic Chemistry, Chinese Academy of Sciences, Shanghai, China

**Keywords:** trifluoromethyl, nitroamines, aza-Henry, ammonium salts, adamantane-type, anticancer

## Abstract

Amino acid-derived quaternary ammonium salts were successfully applied in the asymmetric aza-Henry reaction of nitromethane to *N*-Boc trifluoromethyl ketimines. α-Trifluoromethyl β-nitroamines were synthesized in good to excellent yields with moderate to good enantioselectivities. This reaction is distinguished by its mild conditions, low catalyst loading (1 mol%), and catalytic base. It also proceeded on a gram scale without loss of enantioselectivity. The products were transformed to a series of adamantane-type compounds containing chiral trifluoromethylamine fragments. The potent anticancer activities of these compounds against liver cancer HepG2 and melanoma B16F10 were evaluated. Six promising compounds with notable efficacy have potential for further development.

## 1 Introduction

The aza-Henry reaction involves the addition of nitroalkanes to imines, which is one of the most important reactions in forming carbon–carbon bonds (Marqués-López et al., 2009). The aza-Henry reaction readily introduces diverse functional groups, including amine and nitro moieties, into organic scaffolds (Noble and Anderson, 2013). Notably, the resulting β-nitroamines, featuring two nitrogen atoms with distinct oxidation states, serve as crucial synthons in organic synthesis. The facile conversion of β-nitroamines into amino acids, chiral diamines, and other key medicinal frameworks has continuously attracted the attention of synthetic chemists. Consequently, the aza-Henry reaction has found extensive application in the synthesis of numerous active compounds, drugs, and natural products (Noble and Anderson, 2013). In recent years, trifluoromethyl amine-containing compounds have been widely favored by synthetic chemists because they play a very important role not only in chiral drugs but also in natural products (Yoder and Kumar, 2002; Nie et al., 2011; Qiu and Qing, 2011). The catalytic enantioselective aza-Henry reaction is one of the most practical reactions that convert ketimines into the corresponding optically active α-trifluoromethyl amine compounds (Onyeagusi and Malcolmson, 2020). However, the asymmetric aza-Henry reaction of trifluoromethyl imines is still less reported (Xie et al., 2011; Zhang et al., 2011; Kutovaya et al., 2015; Wang et al., 2019a; Li et al., 2019; Krstic et al., 2023).

**SCHEME 1:**
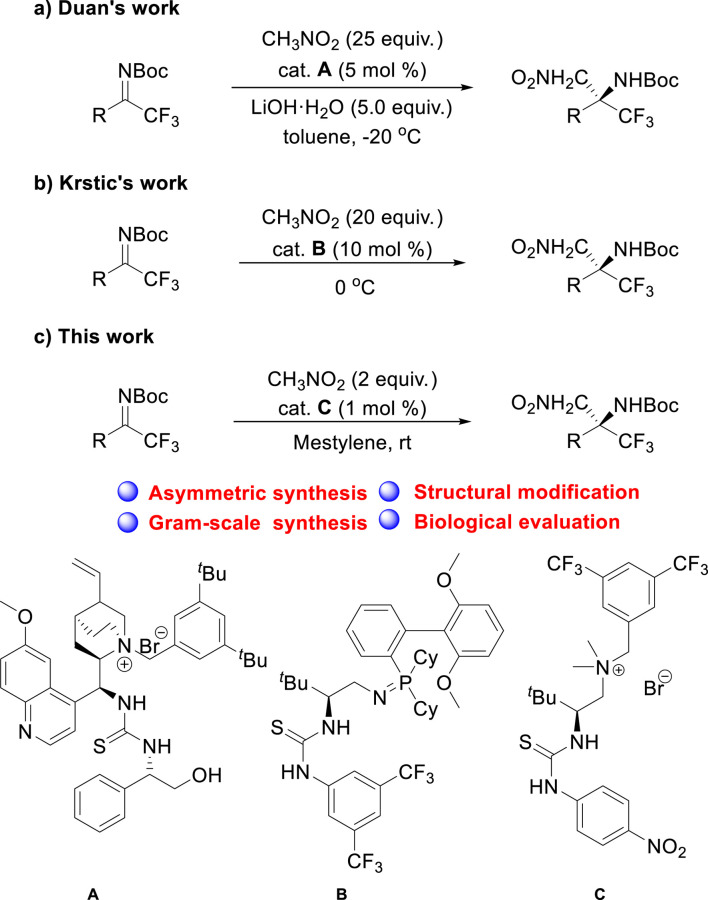
**(A–C)** Enantioselective construction of trifluoromethyl β-nitroamines via chiral ion-pair catalysts.

In recent years, there has been significant interest in the development of efficient and enantioselective methods for synthesizing chiral trifluoromethyl amines. This has led to the exploration of a variety of strategies, including the use of chiral organocatalysts and auxiliary groups. Wang (Xie et al., 2011), Liu (Zhang et al., 2011; Li et al., 2019), and Nenajdenko (Kutovaya et al., 2015) have reported several notable contributions to this field. In addition, Duan (Wang et al., 2019a) and Krstic et al. (2023) reported the asymmetric aza-Henry reaction of trifluoromethyl ketimines using different kinds of ion-pair catalysts (Schemes 1A, and B).

Despite these advances, the synthesis of chiral trifluoromethyl amines using readily prepared catalysts with low catalyst loading still remains a challenging goal for organic chemists (Onyeagusi and Malcolmson, 2020). Amino acid-derived quaternary ammonium catalysts are inexpensive, stable, and easy to prepare and have been widely used in asymmetric catalysis over the past decade (Wang et al., 2013a; Wang et al., 2013b; Zhang et al., 2016). In continuation of our efforts to synthesize chiral trifluoromethylamine analogs via organocatalysis (Du et al., 2020; Zhao et al., 2023), we now report *N-*protected trifluoromethyl ketimines as the substrate in the asymmetric aza-Henry reaction catalyzed by the easily prepared quaternary ammonium and demonstrated the products as potent anticancer agents (Scheme 1C).

## 2 Results and discussions

The aza-Henry reaction using *N*-Boc trifluoromethyl ketimines and nitromethane was designed as the model reaction. First, the widely used double-hydrogen-bonding quaternary ammonium salt **4a** derived from *L*-phenylalanine was tested in this reaction, and to our surprise, the reaction produced **3a** with moderate enantioselectivity (Table 1, entry 1). The catalysts bearing distinct amino acid backbones were then screened to investigate the performance of enantioselective control. Replacement of the catalyst backbone with *L*-*tert*-leucine **4b** and *L*-isoleucine **4c** had similar effects. The enantiomeric excess (ee) value of the product from **4b** remained unchanged, while the enantioselectivity of the product from **4c** decreased (Table 1, entries 2–3). Hydrogen bonding patterns significantly impacted enantioselectivity. Catalyst **4d** with a urea group reduced the enantioselectivity (Table 1, entry 4), while the amide catalyst **4e** flipped the enantiomer preference (Table 1, entry 5). These results demonstrate the critical importance of hydrogen bonding type for the reaction. When the thiourea moiety within the catalyst was further examined (**4f** and **4g**), it was found that aryl thiourea substituents for both the electron-donating methoxy group and electron-withdrawing nitro group could give better results (Table 1, entries 6–7). The product obtained from catalyst **4f**, in which aryl thiourea was substituted with the nitro group on the para position, gave an enantioselectivity of 70%. By adjusting the structure of the ammonium salt center (**4h** and **4i**), the ee values of the products were found to be slightly decreased (Table 1, entries 8–9). Therefore, we identified catalyst **4f** as the optimal catalyst and screened the solvent and base.

**TABLE 1:** Optimization of the reaction conditions^a^.

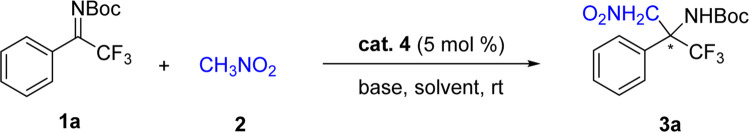						
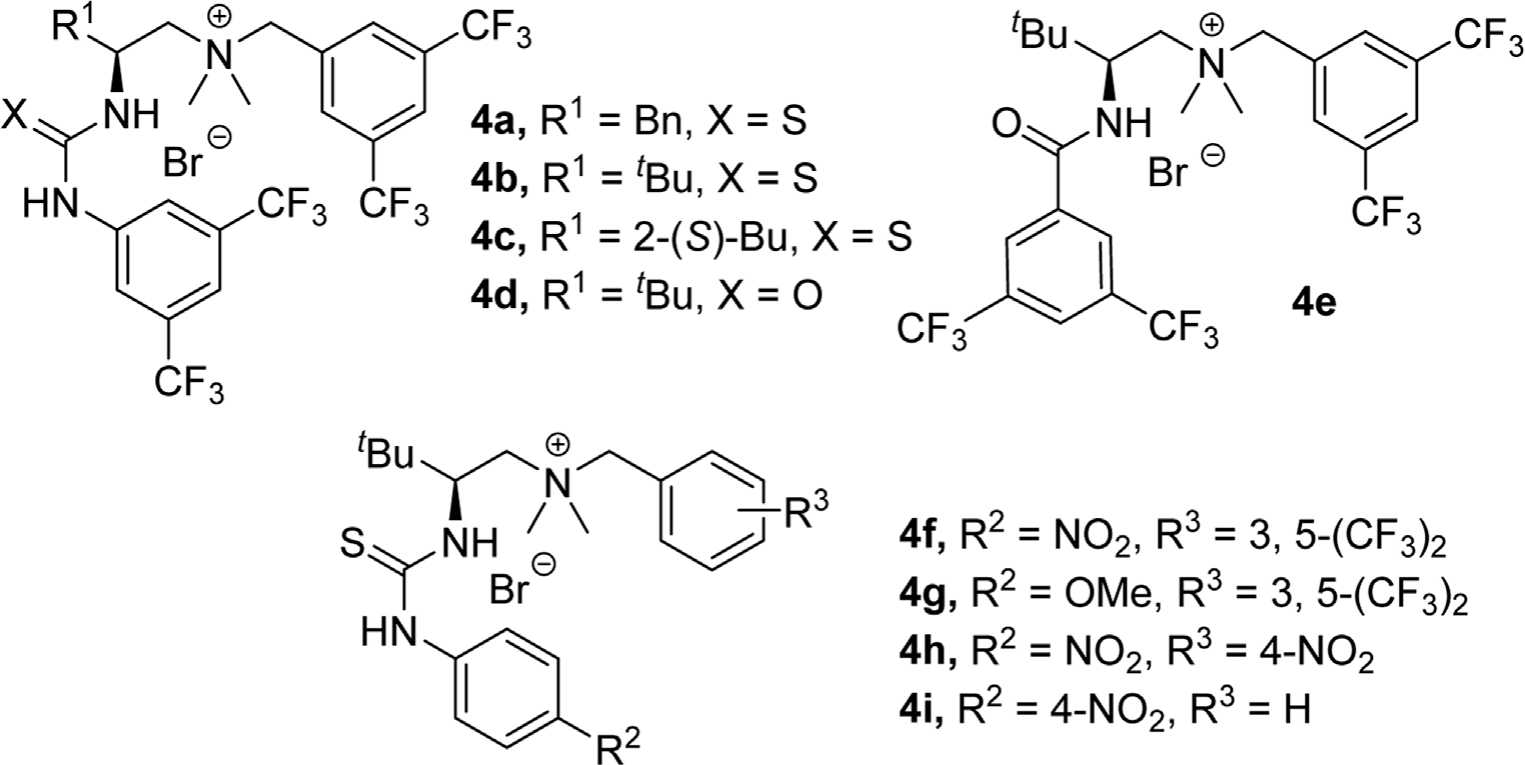						
Entry	Base (eq.)	Cat	Solvent	t	Yield/%^b^	ee/%^c^
1	K_2_CO_3_ (2.0)	**4a**	Toluene	12 h	72	58
2	K_2_CO_3_ (2.0)	**4b**	Toluene	12 h	78	58
3	K_2_CO_3_ (2.0)	**4c**	Toluene	12 h	78	55
4	K_2_CO_3_ (2.0)	**4d**	Toluene	5 h	76	46
5	K_2_CO_3_ (2.0)	**4e**	Toluene	2 day	75	−32
6	K_2_CO_3_ (2.0)	**4f**	Toluene	12 h	70	70
7	K_2_CO_3_ (2.0)	**4g**	Toluene	1 day	80	65
8	K_2_CO_3_ (2.0)	**4h**	Toluene	3 day	75	69
9	K_2_CO_3_ (2.0)	**4i**	Toluene	2 day	75	69
10	Cs_2_CO_3_ (2.0)	**4f**	Toluene	5 h	63	38
11	K_3_PO_4_ (2.0)	**4f**	Toluene	18 h	80	67
12	NaOH (2.0)	**4f**	Toluene	12 h	56	71
13^d^	K_2_CO_3_ (2.0)	**4f**	Toluene	7 day	77	76
14	K_2_CO_3_ (1.0)	**4f**	Toluene	12 h	85	75
15	K_2_CO_3_ (0.1)	**4f**	Toluene	12 h	80	74
16	K_2_CO_3_ (0.1)	**4f**	PhCF_3_	2.5 day	65	68
17	K_2_CO_3_ (0.1)	**4f**	PhCl	2 day	88	71
18	K_2_CO_3_ (0.1)	**4f**	PhF	2.5 day	72	68
19	K_2_CO_3_ (0.1)	**4f**	THF	2.5 day	74	59
20	K_2_CO_3_ (0.1)	**4f**	2-Me-THF	2 day	78	65
21	K_2_CO_3_ (0.1)	**4f**	Mesitylene	1 day	89	80
22^e^	K_2_CO_3_ (0.1)	**4f**	Mesitylene	2.5 day	88	80
23	‒	**4f**	Mesitylene	1 day	ND	‒

^a^ Unless otherwise noted, the reaction was performed with 0.10 mmol of **1a**, 0.20 mmol of **2**, catalyst **4** (5 mol%), and base in 1.0 mL solvent.

^b^ Isolated yield.

^c^ Determined by chiral HPLC analysis.

^d^ 0°C.

^e^ **4f** (1 mol%) was used.

Additional base screening indicated that K_2_CO_3_ was the best base for further screening based on the reaction time, yield, and enantioselectivity (Table 1, entries 10–12). When the reaction was conducted at 0°C, both the yield and enantioselectivity of the reaction were slightly improved, but the reaction time was also extended to 7 days (Table 1, entry 13). The enantioselectivity of the product was improved slightly by decreasing the equivalent amount of K_2_CO_3_ (Table 1, entries 14–15), which is very essential for large-scale synthesis since only catalytic amounts of the base are required in the proton transfer process. Finally, we screened solvents such as trifluorotoluene, chlorobenzene, fluorobenzene, tetrahydrofuran, 2-methyl tetrahydrofuran, and mesitylene (Table 1, entries 16–21) and found that the enantioselectivity of the product increased up to 80% when mesitylene was applied. Reduction of the catalyst loading to 1 mol% resulted in the ee value of the product remaining unchanged, with a slightly longer reaction time needed (Table 1, entry 22). No product was detected without a base (Table 1, entry 23). The absolute stereochemistry of **3a** was determined to be *S* by comparing its optical rotation with the reported value (Krstic et al., 2023).

Under the optimized conditions (Table 1, entry 22), the scope of the substrate of this reaction was next explored. A series of *N*-Boc trifluoromethyl ketimines were investigated under standard reaction conditions. The experimental results showed that the substituents on the aryl ring regardless of the electron-withdrawing and electron-donating groups were well-tolerated and the corresponding products could be obtained in high yields with good enantioselectivities (Table 2, **3a**-**3h**). It is worth mentioning that the ee value of **3d** could be increased up to 99% in 75% yield after a single recrystallization step. The reaction is also compatible with naphthalene cycles and heteroaromatic cycles (Table 2, **3i** and **3j**). When the trifluoromethyl group within the substrate was replaced by the perfluoropropyl group, good yield and enantioselectivity were also achieved (Table 2, **3k**). In addition, trifluoromethyl ketimine containing an alkynyl group was applied in the reaction, which gave the product **3l** in good yield and moderate enantioselectivity (Table 2, **3l**) (Dai et al., 2019; Ran et al., 2020; Yang et al., 2020; Li et al., 2021).

**TABLE 2:** Aza-Henry reaction of *N*-Boc trifluoromethyl ketimines catalyzed by the ion-pair catalyst^a^.

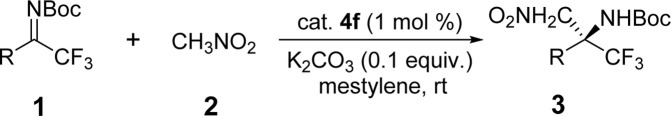
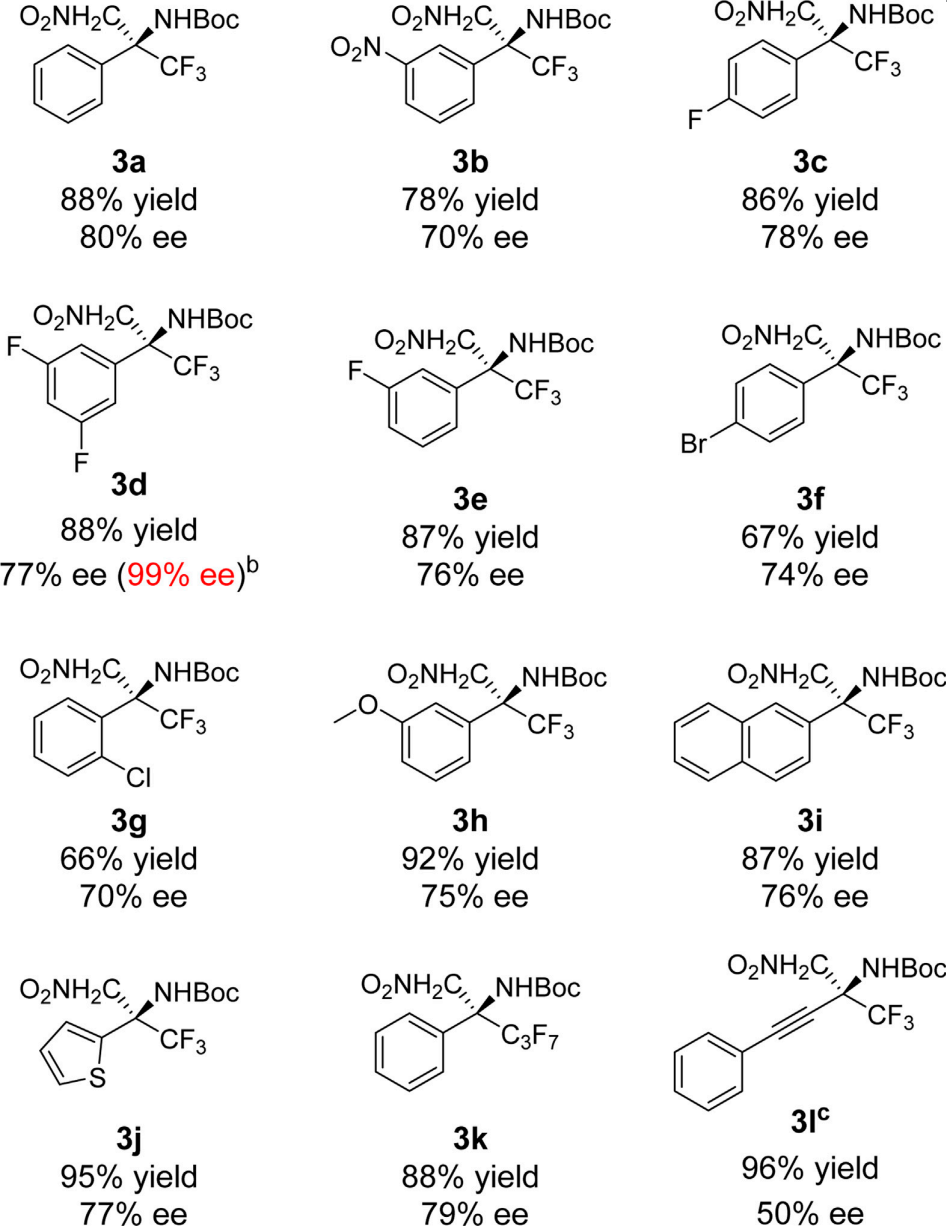

^a^ Unless otherwise noted, the reaction was performed with 1 (0.1 mmol), 2 (0.20 mmol), catalyst 4f (1 mol%), and base (0.1 equiv.) in solvent (1.0 mL) for 3 days.

^b^ After recrystallization.

^c^ Catalyst 4e (5 mol%) and K_2_CO_3_ (2.0 equiv.) were used.

**SCHEME 2:**
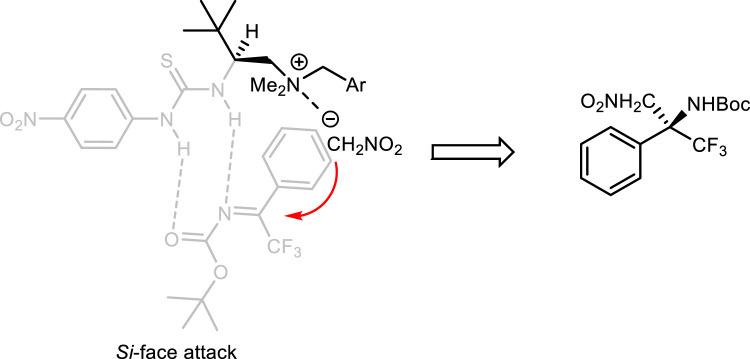
Proposed transition state leading to the desired configuration.

Based on the previous results, (Wang et al., 2013a; Wang et al., 2013b; Zhang et al., 2016; Du et al., 2020), a transition state was proposed. The substrate **1a** with an *N*-Boc group may interact with thiourea in the catalyst **4f** via N–H and O–H hydrogen bonding. The face selectivity of the complex was controlled by the steric hindrance, and the imine should be attacked from the *Si*-face by nitromethane anions, which were activated by the ammonium salt center through electrostatic interaction (Scheme 2).

To demonstrate the efficiency and applicability of this reaction, gram-scale experiments with six representative substrates (**1a, 1c-1e, 1h,** and **1j**) were carried out successfully (Table 3). The following one-pot reduction of their products **3a, 3c-3e, 3h,** and **3j** with NiCl₂,·6H₂O, and NaBH₄ in methanol afforded the desired products in good yields in two steps, with no observed loss of enantioselectivity (**5a** and **5c**). As a result, 1.8–2.6 g of the valuable chiral diamine intermediates **5a**–**5f** was obtained easily, which serve as crucial fragments to access adamantane-type derivatives.

**TABLE 3:** Gram-scale synthesis of trifluoromethyl 1,2-diamines in one pot^a^.

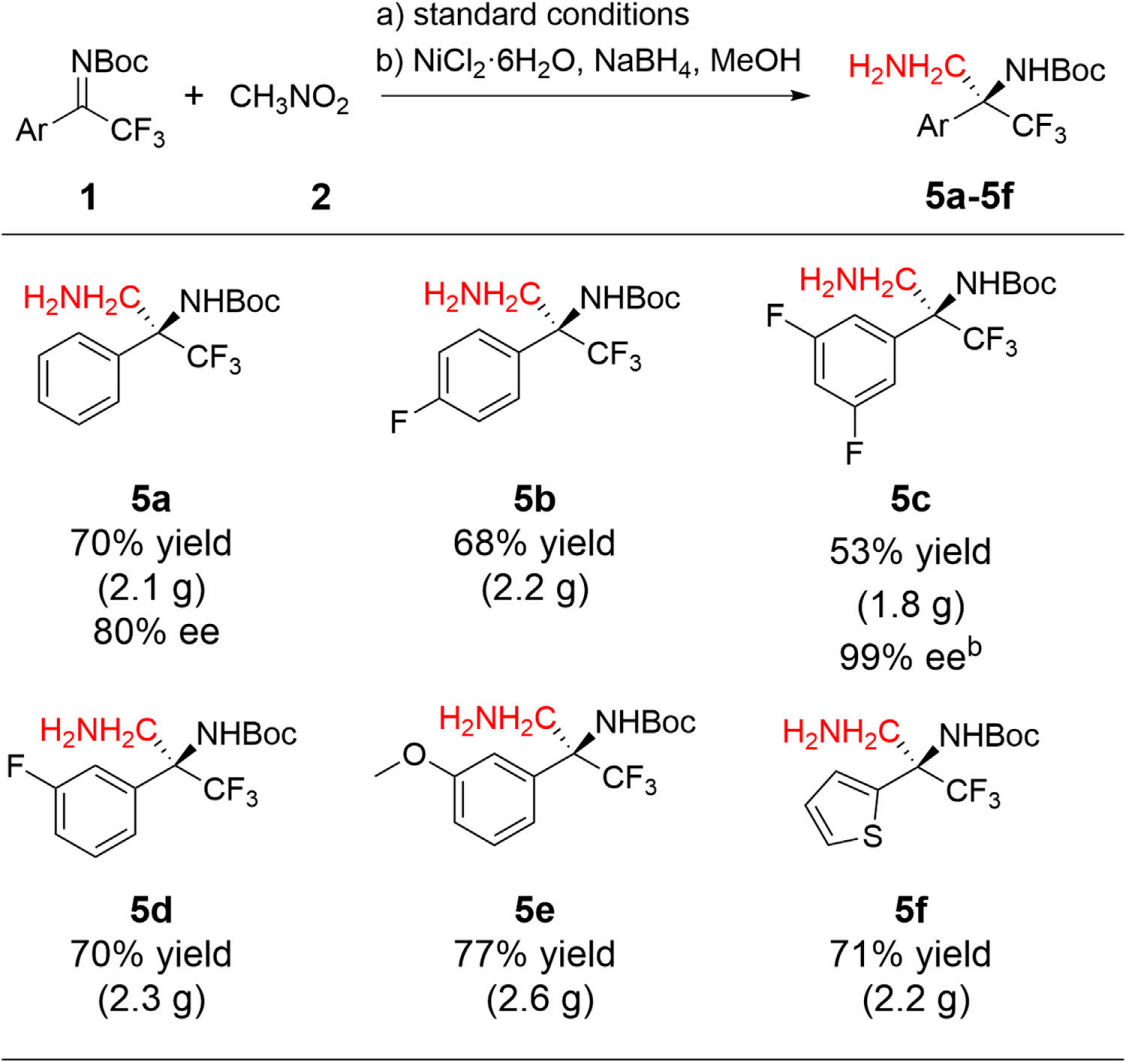

^a^ Step 1: standard conditions; Step 2: NiCl₂,·6H₂O (1.0 equiv.), NaBH₄ (7.0 equiv.), and MeOH.

^b^ After recrystallization in step 1.

**SCHEME 3:**
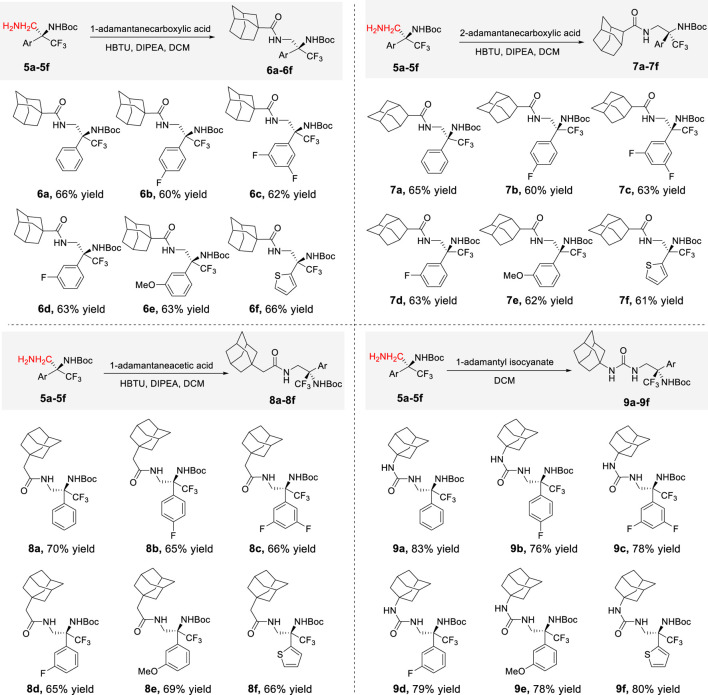
Transformations of chiral trifluoromethyl amines to diverse adamantane-type derivatives.

Intrigued by the promising biological activity of polycyclic polyprenylated acylphloroglucinol derivatives (Yang et al., 2018; Wang et al., 2019b; Phang et al., 2020; Wang et al., 2021), we aimed to integrate a key structural element, adamantane, into these molecules. Adamantane, the quintessential diamond-like molecule with its symmetrical and rigid cage structure, boasts a unique combination of valuable properties, including potent antioxidant activity, high lipid solubility, impressive thermal stability, and comparatively low toxicity (Spilovska et al., 2016). These exceptional attributes have propelled adamantane-type derivatives to the forefront of diverse applications, from systemic therapies to topical treatments. Herein, chiral diamines **5a**–**5f** were successfully derived into a series of adamantane-type derivatives by introducing 1-adamantanecarboxylic acid (**6a**–**6f**), 2-adamantanecarboxylic acid (**7a**–**7f**), and 1-adamantaneacetic acid (**8a**–**8f**) through amide condensation, along with urea derivatives (**9a**–**9f**), through condensation with 1-adamantanecarboxylic acid isocyanate (Scheme 3). The enantioselectivities of adamantane-type derivatives derived from **5a** and **5c** remained consistent with those of **5a** and **5c** (for details, see Supplementary Material), indicating that these processes did not affect the enantiomeric excess (ee) values.

**SCHEME 4:**
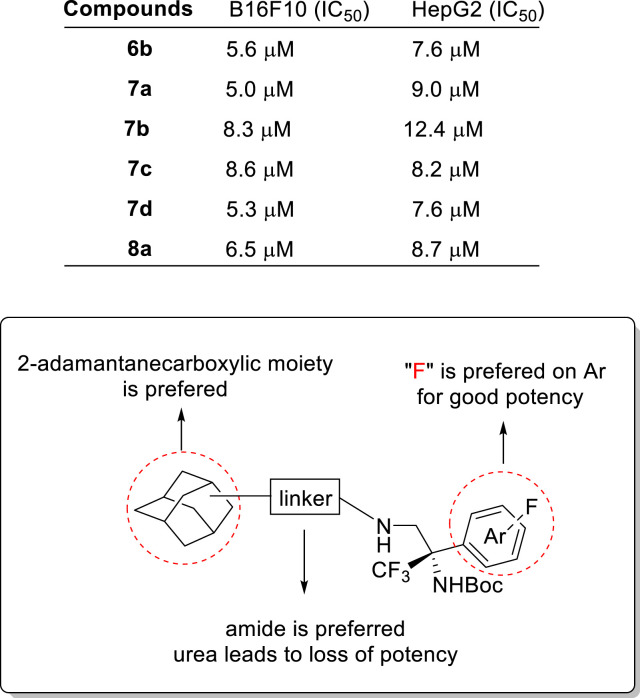
IC_50_ of selected adamantane-type compounds against two cell lines and SARs.

Based on the published literature and our ongoing research, the incorporation of adamantane units into therapeutic scaffolds has shown significant potential in recent years. Some adamantane-type compounds have demonstrated promising biological activities, such as anti-psychiatric, anticancer and neurological disorders, and anti-inflammatory effects (Burmistrov et al., 2022; Tao et al., 2024; Gutti et al., 2023; Ragshaniya et al., 2024). To explore this further, we have utilized the uniqueness of the adamantane moiety in our synthetic compounds with the introduction of various functional groups that may enhance the potency as well as selectivity. Thus, the synthesized 24 adamantane-type derivatives were evaluated against two cancer cell lines: liver carcinoma HepG2 and melanoma B16F10. The initial impact of these compounds on cell viability was assessed. Based on these findings, a subset of compounds demonstrating promising cytotoxicity was selected for further investigation. The IC_50_ values of the chosen compounds were subsequently determined for both HepG2 and B16F10 cell lines (Scheme 4). Compound **6b** with a 1-adamantane structure exhibited remarkable bioactivity against both HepG2 and B16F10, showcasing its potential as a broad-spectrum anticancer agent. Compounds **7a**–**7d** with a 2-adamantane structure collectively displayed impressive bioactivity against both cancer lines, highlighting the efficacy of their structural motifs. Compound **8a**, formed through condensation with 1-adamantaneacetic acid, also exhibited similar bioactivity against liver cancer HepG2 and melanoma B16F10; however, urea derivatives (**9a**–**9f**) did not show any good results in the bioassay, which may be attributed to the size of the molecular cavity itself, and it is consistent with the fact that amides are much more likely to be seen in medicinal chemistry. Preliminary findings indicate that the incorporation of 2-adamantanecarboxylic acid can lead to relatively favorable bioactivity, offering essential insights for the structural modification of compounds with antitumor properties. Based on these results, the structure–activity relationship is concluded in Scheme 4.

## 3 Conclusion

In summary, we present the asymmetric aza-Henry reaction involving nitromethane and *N*-Boc trifluoromethyl ketimines catalyzed by readily prepared quaternary ammonium salts derived from amino acids. The reaction demonstrates efficiency under mild conditions and with low catalyst loading. It exhibits good tolerance, yielding the desired products in high yields with notable enantioselectivities. A series of adamantane-type derivatives containing chiral trifluoromethylamine fragments were designed and synthesized successfully. The biological evaluation of these compounds against liver cancer HepG2 and melanoma B16F10 cell lines identified promising lead compounds, allowing the establishment of a structure–activity relationship.

## 4 Materials and methods

For experimental procedures and compound characterization data, see the Supplementary Material.

### 4.1 Experimental section

#### 4.1.1 General procedure for the asymmetric aza-Henry reaction of *N*-Boc ketimines

To a solution of quaternary ammonium salt (0.001 mmol, 1 mol%) in mesitylene (1.0 mL) was added nitromethane (2.0 equiv.) and base (0.1 equiv.). After stirring for 5 min, the corresponding *N*-Boc trifluoromethyl ketimine (0.1 mmol) was added. The reaction was monitored by TLC until it was completed, and the residue was purified by flash silica gel column chromatography to give trifluoromethyl β-nitroamines.

#### 4.1.2 General procedure for the gram-scale synthesis of trifluoromethyl 1,2-diamines in one pot

To a solution of quaternary ammonium salt (0.1 mmol, 1 mol%) in mesitylene (50.0 mL) was added nitromethane (2.0 equiv.) and base (0.1 equiv.). After stirring for 10 min, the corresponding *N*-Boc trifluoromethyl ketimine (10.0 mmol) was added. The reaction was monitored by TLC until it was completed; then, the solvent was removed under vacuum, MeOH (50.0 mL) and NiCl_2_·6H_2_O (1.0 equiv.) were added to the mixture, NaBH_4_ (7.0 equiv.) was added in portions to a stirred methanol solution at 0°C, and the system was stirred at 0°C for 2 h. The reaction was monitored by TLC. Saturated aqueous NH_4_Cl (5.0 mL) was added to quench the reaction, and the resulting mixture was stirred at room temperature until no gas evolved. Anhydrous Na_2_SO_4_ was added into the mixture. After filtration, the filtrate was concentrated, and the residue was purified by silica gel column chromatography to give the trifluoromethyl 1,2-diamines.

#### 4.1.3 General procedure for the synthesis of 6a–6f

To a solution of trifluoromethyl 1,2-diamine (1.0 mmol, 1.0 equiv.) in DCM (5.0 mL) was added 1-adamantanecarboxylic acid (1.5 equiv.), HBTU (1.5 equiv.), and DIPEA (2.0 equiv.), and the reaction was monitored by TLC until it was completed, and the residue was purified by flash silica gel column chromatography to give **6a**–**6f**.

#### 4.1.4 General procedure for the synthesis of 7a–7f

To a solution of trifluoromethyl 1,2-diamine (1.0 mmol, 1.0 equiv.) in DCM (5.0 mL) was added 2-adamantanecarboxylic acid (1.5 equiv.), HBTU (1.5 equiv.), and DIPEA (2.0 equiv.), and the reaction was monitored by TLC until it was completed, and the residue was purified by flash silica gel column chromatography to give **7a**–**7f**.

#### 4.1.5 General procedure for the synthesis of 8a–8f

To a solution of trifluoromethyl 1,2-diamine (1.0 mmol, 1.0 equiv.) in DCM (5 mL) was added 1-adamantaneacetic acid (1.5 equiv.), HBTU (1.5 equiv.), and DIPEA (2.0 equiv.), and the reaction was monitored by TLC until the reaction was completed, and the residue was purified by flash silica gel column chromatography to give **8a**–**8f**.

#### 4.1.6 General procedure for the synthesis of 9a–9f

To a stirred solution of trifluoromethyl 1,2-diamine (1.0 mmol, 1.0 equiv.) in DCM (5.0 mL) was added 1-adamantyl isocyanate (1.5 equiv.), and the reaction was monitored by TLC until the reaction was completed, and the residue was purified by flash silica gel column chromatography to give **9a**–**9f**.

## Data Availability

The datasets presented in this study can be found in online repositories. The names of the repository/repositories and accession number(s) can be found in the article/Supplementary Material.
