# Supplementary material for: Asymmetric aza-Henry reaction toward trifluoromethyl β-nitroamines and biological investigation of their adamantane-type derivatives
Source: Front Chem. 2024 May 10;12:1398946. doi: 10.3389/fchem.2024.1398946 (PMC11116722; doi:10.3389/fchem.2024.1398946)

Asymmetric Aza-Henry Reaction towards Trifluoromethyl β-Nitroamines and Biological Investigation of Their Adamantane-type derivatives

Yi Ren[1], Mengyuan Du[2], Ziyu Peng[2] Changwu Zheng[1*], Gang Zhao[2*]

[1]School of Pharmacy, Shanghai University of Traditional Chinese Medicine, Shanghai 201203, China. E-mail: zhengcw@shutcm.edu.cn

[2]Key Laboratory of Synthetic Chemistry of Natural Substances, Shanghai Institute of Organic Chemistry, Chinese Academy of Sciences, 345 Lingling Road, Shanghai 200032, P. R. China. E-mail: zhaog@sioc.ac.cn

General Information

Unless otherwise noted, all solvents used in the reaction were commercially available without further purification. Purification of corresponding products was carried out by flash chromatography using silica gel (100-200 mesh, 300–400 mesh). TLC was visualized by UV fluorescence (254 nm) or KMnO4, phosphomolybdic acid. The ^{1}H NMR spectra were recorded on a Bruker 400 or an Agilent 400 (400 MHz), an Agilent 500 (500 MHz). All chemical shifts (δ) were given in ppm. The experimental data were reported as follows: chemical shift, integration, multiplicity (s = single, d = doublet, t = triplet, q = quartet, br = broad, m = multiplet) and coupling constants (Hz). ^{13}C NMR spectra were recorded on a Bruker 400 (101 MHz) or an Agilent 500 (126 MHz). ^{19}F NMR spectra were recorded on an Agilent 400 (376 MHz). High-resolution mass spectra (HRMS) were recorded on a Bruker APEXIII 7.0 Tesla ESI-FT mass spectrometer or an Agilent TOF/LC-MS 1260-6230 mass spectrometer. Optical rotations were measured on an Anton Paar MCP 5500 Polarimeter at λ = 589 nm. Cell line sources are from Shanghai Institutes for Biological Sciences, Chinese Academy of Sciences, Cell Resource Center.

Experimental data

Compound **3a**

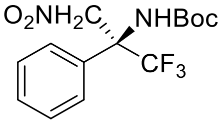

88% yield; ^{1}**H NMR** ($CDCl_3$, 400 MHz) δ 7.31-7.35 (m, 5H), 5.35-5.45 (m, 3H), 1.35 (s, 9H); ^{13}**C NMR** ($CDCl_3$, 101 MHz) δ 153.6, 132.6, 129.7, 129.0, 126.2, 123.9 (q, *J* = 284.4 Hz), 81.8, 73.1, 63.9 (q, *J* = 28.0 Hz), 28.0; ^{19}**F NMR** ($CDCl_3$, 376 MHz) δ -79.7; **HRMS** (EI): calcd for [$C_{14}H_{17}F_3N_2O_4$] requires 334.1135; found 334.1129; enantiomeric excess: 80%, determined by HPLC (Chiralpak AD-H, hexane/i-PrOH 90/10, flow rate 1.0 mL/min; t_{mjnor} = 6.8 min, t_{major} = 7.6 min, λ = 254 nm); $[\alpha]_D^{25}$ = 16.2 (*c* = 1.0, $CHCl_3$).

Compound **3b**

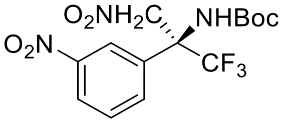

78% yield; ^{1}**H NMR** ($CDCl_3$, 400 MHz) δ 8.31-8.34 (m, 2H), 7.77-7.79 (m, 1H), 7.64-7.69 (m, 1H), 5.45-5.56 (m, 3H), 1.46 (s, 9H); ^{13}**C NMR** ($CDCl_3$, 101 MHz) δ 153.5, 148.6, 135.0, 132.2, 130.1, 124.7, 123.4 (q, *J* = 284.3 Hz), 121.9, 82.7, 73.0, 63.8 (q, *J* = 28.3 Hz), 28.0; ^{19}**F NMR** ($CDCl_3$, 376 MHz) δ -75.0; **HRMS** (EI): calcd for [M-C_4H_8] ($C_{10}H_8F_3N_3O_6$) requires 323.0360; found 323.0364; enantiomeric excess: 70%, determined by HPLC (Chiralpak AD-H, hexane/i-PrOH 95/5, flow rate 1.0 mL/min; t_{major} = 24.0 min, t_{mjnor} = 50.7 min, λ = 254 nm); $[\alpha]_D^{25}$ = 7.7 (*c* = 1.0, $CHCl_3$).

Compound **3c**

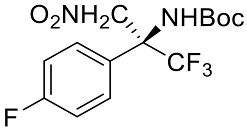

86% yield; ^{1}**H NMR** ($CDCl_3$, 400 MHz) δ 7.44-7.40 (m, 2H), 7.12 (t, *J* = 8.8Hz, 2H), 5.46 (br, 3H), 1.45 (s, 9H); ^{13}**C NMR** ($CDCl_3$, 101 MHz) δ 164.5, 162.6, 153.6, 128.3 (d, *J* = 8.5 Hz), 123.7 (q, *J* = 284.3 Hz), 116.1 (d, *J* = 21.8 Hz), 82.0, 73.1, 63.7 (q, *J* =

28.1 Hz), 28.1; ^{19}**F NMR** ($CDCl_3$, 376 MHz) δ -75.3, -111.4; **HRMS** (EI): calcd for [$C_{14}H_{16}F_4N_2O_4$] requires 352.1041; found 352.1038; enantiomeric excess: 78%, determined by HPLC (Chiralpak AD-H, hexane/i-PrOH 95/5, flow rate 1.0 mL/min; t_{mjnor} = 9.6 min, t_{major} = 12.2 min, λ = 254 nm); $[\alpha]_D^{25}$ = 20.6 (*c* = 1.0, $CHCl_3$).

Compound **3d**

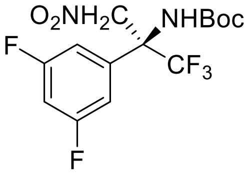

88% yield; ^{1}**H NMR** (400 MHz, $CDCl_3$) δ 6.98 (d, *J* = 7.0 Hz, 2H), 6.90 (t, *J* = 8.4 Hz, 1H), 5.49 (s, 1H), 5.46 (s, 1H), 5.36 (d, *J* = 12.1 Hz, 1H), 1.46 (s, 9H); ^{13}**C NMR** (101 MHz, $CDCl_3$) δ 163.14 (dd, *J* = 250.1, 12.8 Hz), 153.44, 136.57 (t, *J* = 9.1 Hz), 123.38 (q, *J* = 286.4 Hz), 110.01 (d, *J* = 28.8 Hz), 105.43 (t, *J* = 25.1 Hz), 82.43, 72.84, 63.57 (dd, *J* = 56.9, 28.6 Hz), 28.03; ^{19}**F NMR** (376 MHz, $CDCl_3$) δ -74.91, -107.39; **HRMS** (ESI): calcd for [$C_{14}H_{15}F_5N_2O_4Na$] requires 393.08442; found 393.08434; enantiomeric excess (After recrystallization): >99%, determined by HPLC (Chiralpak IC, hexane/i-PrOH 95/5, flow rate 1.0 mL/min; t_{major} = 4.1 min, t_{minor} = 5.2 min, λ = 254 nm); $[\alpha]_D^{25}$ = 30.0 (*c* = 0.5, $CHCl_3$).

Procedure of the recrystallization: Compound **3d** (1.0 g) was dissolved in 8.0 mL petroleum ether, which was heated to reflux for 3 mins until all the solids were dissolved. The solution was cooled to rt gradually over 30 mins. The solid (750 mg, 75% yield, >99% ee) was obtained via filtration.

Compound **3e**

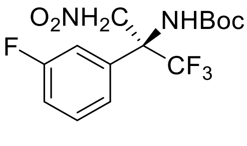

87% yield; ^{1}**H NMR** (400 MHz, $CDCl_3$) δ 7.41 (dd, *J* = 14.2, 8.2 Hz, 1H), 7.23 – 7.09 (m, 3H), 5.54 – 5.41 (m, 3H), 1.45 (s, 9H); ^{13}**C NMR** (101 MHz, $CDCl_3$) δ 162.89 (d, *J* = 247.4 Hz), 153.51, 135.19 (d, *J* = 7.1 Hz), 130.54 (d, *J* = 8.3 Hz), 123.63 (q, *J* = 286.3 Hz), 121.77, 116.80 (d, *J* = 21.0 Hz), 114.01 (d, *J* = 24.5 Hz), 82.09, 73.06, 63.67

(q, J = 28.7 Hz), 28.03; **^{19}F NMR** (376 MHz, $CDCl_3$) δ -74.97, -111.02; **HRMS** (ESI): calcd for [$C_{14}H_{16}F_4N_2O_4Na$] requires 375.09384; found 375.09373; enantiomeric excess: 76%, determined by HPLC (Chiralpak IC, hexane/i-PrOH 95/5, flow rate 1.0 mL/min; t_{major} = 4.9 min, t_{minor} = 6.5 min, λ = 254 nm); $[\alpha]_D^{25}$ = 25.0 (c = 0.5, $CHCl_3$).

Compound **3f**

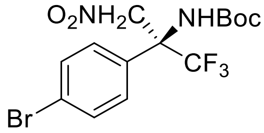

67% yield; **^{1}H NMR** ($CDCl_3$, 400 MHz) δ 7.55-7.77 (m, 2H), 7.30 (d, J = 8.8 Hz, 2H), 5.41-5.48 (m, 3H), 1.45 (s, 9H); **^{13}C NMR** ($CDCl_3$, 100 MHz) δ 153.5, 132.2, 131.7, 127.9, 124.2, 123.5 (q, J = 284.4 Hz), 82.1, 72.9, 63.8 (q, J = 28.1 Hz), 28.1; **^{19}F NMR** ($CDCl_3$, 376 MHz) δ -75.1; **HRMS** (EI): calcd for [M-OC_4H_{10}] ($C_{10}H_6F_3N_2O_3Br$) requires 337.9508; found 337.9504; enantiomeric excess: 74%, determined by HPLC (Chiralpak AD-H, hexane/i-PrOH 95/5, flow rate 1.0 mL/min; t_{mjnor} = 6.4 min, t_{major} = 7.5 min, λ = 254 nm); $[\alpha]_D^{25}$ = 14.8 (c = 1.0, $CHCl_3$).

Compound **3g**

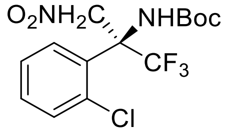

66% yield; **^{1}H NMR** ($CDCl_3$, 400 MHz) δ 7.52-7.54 (m, 1H), 7.44-7.47 (m, 1H), 7.34-7.36 (m, 2H), 6.01 (br, 1H), 5.50-5.53 (m, 1H), 5.33 (br, 1H), 1.38 (s, 9H); **^{13}C NMR** ($CDCl_3$, 100 MHz) δ 152.7, 132.8, 132.5, 130.7, 129.19, 129.16, 127.5, 124.3 (q, J = 286.6 Hz), 81.4, 74.6, 64.2 (q, J = 29.1 Hz), 28.0; **^{19}F NMR** ($CDCl_3$, 376 MHz) δ -76.14; **HRMS** (EI): calcd for [M-C_4H_8] ($C_{10}H_8F_3N_2O_4Cl$) requires 312.0119; found 312.0115; enantiomeric excess: 70%, determined by HPLC (Chiralpak AD-H, hexane/i-PrOH 95/5, flow rate 1.0 mL/min; t_{major} = 8.5 min, t_{mjnor} = 10.6 min, λ = 254 nm); $[\alpha]_D^{25}$ = 8.8 (c = 0.5, $CHCl_3$).

Compound **3h**

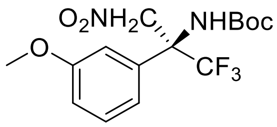

92% yield; **^{1}H NMR** ($CDCl_3$, 400 MHz) δ 7.34 (d, *J* = 8.4 Hz, 1H), 6.94-6.99 (m, 3H), 5.45-5.53 (m, 3H), 3.80 (s, 3H), 1.45 (s, 9H); **^{13}C NMR** ($CDCl_3$, 100 MHz) δ 159.9, 153.5, 134.0, 129.9, 123.8 (q, *J* = 285.0 Hz), 118.1, 114.5, 112.9, 81.7, 73.0, 63.8 (q, *J* = 27.9 Hz), 55.3, 28.0; **^{19}F NMR** ($CDCl_3$, 376 MHz) δ -74.8; **HRMS** (ESI): calcd for $[M+Na]^+$ $(C_{15}H_{19}F_3N_2O_5Na)^+$ requires 387.1138; found 387.1136; enantiomeric excess: 75%, determined by HPLC (Chiralpak PC-II, hexane/i-PrOH 95/5, flow rate 1.0 mL/min; t_{major} = 5.9 min, t_{mjnor} = 7.2 min, λ = 254 nm); $[\alpha]_D^{25}$ = 4.3 (*c* = 1.0, $CHCl_3$).

Compound **3i**

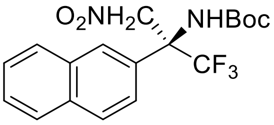

87% yield; **^{1}H NMR** ($CDCl_3$, 400 MHz) δ 7.85-7.90 (m, 4H), 7.51-7.56 (m, 3H), 5.67-5.58 (m, 3H), 1.44 (s, 9H); **^{13}C NMR** ($CDCl_3$, 100 MHz) δ 153.6, 133.4, 132.8, 130.0, 128.9, 128.5, 127.5, 127.4, 126.9, 126.2, 124.0 (q, *J* = 284.4 Hz), 123.0, 81.9, 73.3, 64.1 (q, *J* = 28.0 Hz), 28.1; **^{19}F NMR** ($CDCl_3$, 376 MHz) δ -74.6; **HRMS** (ESI): calcd for $[M+Na]^+$ $(C_{18}H_{19}F_3N_2O_4Na)^+$ requires 407.1189; found 407.1186; enantiomeric excess: 76%, determined by HPLC (Chiralpak PC-II, hexane/i-PrOH 98/2, flow rate 1.0 mL/min; t_{major} = 9.4 min, t_{mjnor} = 12.6 min, λ = 254 nm); $[\alpha]_D^{25}$ = 6.7 (*c* = 1.0, $CHCl_3$).

Compound **3j**

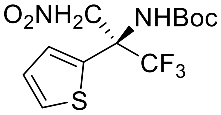

95% yield; **^{1}H NMR** ($CDCl_3$, 400 MHz) δ 7.41 (dd, *J* = 8.8 Hz, 0.8 Hz, 1H), 7.09-7.10 (m, 1H), 7.03-7.05 (m, 1H), 5.54- 5.57 (m, 1H), 5.45-5.48 (m, 2H), 1.47 (s, 9H); **^{13}C NMR** ($CDCl_3$, 100 MHz) δ 153.5, 136.3, 127.4, 127.3, 126.7, 123.3 (q, *J* = 284.7 Hz), 82.0, 72.7, 62.7 (q, *J* = 29.4 Hz), 28.1; **^{19}F NMR** ($CDCl_3$, 376 MHz) δ -76.1; **HRMS** (EI): calcd for $[C_{12}H_{15}F_3N_2O_4S]$ requires 340.0699; found 340.0170; enantiomeric excess: 77%, determined by HPLC (Chiralpak AD-H, hexane/i-PrOH 95/5, flow rate 1.0 mL/min; t_{mjnor} = 8.7 min, t_{major} = 9.6 min, λ = 254 nm); $[\alpha]_D^{25}$ = 16.5 (*c* = 1.0, $CHCl_3$).

Compound **3k**

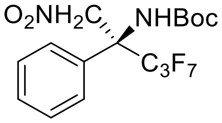

88% yield; **^{1}H NMR** ($CDCl_3$, 400 MHz) δ 7.43 (br, 5H), 5.78 (d, *J* = 12.8 Hz, 1H), 5.51 (d, *J* = 12.4 Hz, 1H), 5.39 (br, 1H), 1.49 (s, 9H); **HRMS** (ESI): calcd for $[M+Na]^+$ $(C_{16}H_{18}F_7N_2O_4Na)^+$ requires 457.0969; found 457.0967; enantiomeric excess: 79%, determined by HPLC (Chiralpak AD-H, hexane/i-PrOH 99/1, flow rate 1.0 mL/min; t_{major} = 7.0 min, t_{mjnor} = 8.3 min, λ = 220 nm); $[\alpha]_D^{25}$ = 14.9 (*c* = 1.0, $CHCl_3$).

Compound **3l**

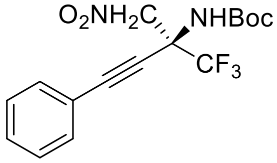

96% yield; **^{1}H NMR** ($CDCl_3$, 400 MHz) δ 7.45-7.48 (m, 2H), 7.38-7.42 (m, 1H), 7.31-7.35 (m, 2H), 5.89 (d, *J* = 12.4Hz, 1H), 5.28 (br, 1H), 4.91 (d, *J* = 12.0Hz, 1H), 1.48 (s, 9H); **^{13}C NMR** ($CDCl_3$, 100 MHz) δ 153.0, 132.2, 130.0, 128.4, 122.8 (q, *J* = 286.0 Hz), 119.9, 88.9, 82.0, 78.1, 72.8, 57.7 (q, *J* = 32.5 Hz), 28.1; **^{19}F NMR** ($CDCl_3$, 376 MHz) δ -80.8; **HRMS** (EI): calcd for $[M-C_4H_8]$ $(C_{12}H_9F_3N_2O_4)$ requires 302.0509; found 302.0518; enantiomeric excess: 50%, determined by HPLC (Chiralpak AD-H, hexane/i-PrOH 98/2, flow rate 1.0 mL/min; t_{mjnor} = 10.6 min, t_{major} = 12.0 min, λ = 254 nm); $[\alpha]_D^{25}$ = -12.8 (*c* = 1.0, $CHCl_3$).

Compound **5a**

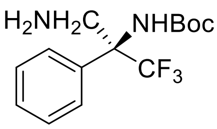

2.1g, 70% yield (over two steps); **^{1}H NMR** (400 MHz, $CDCl_3$) δ 7.51–7.29 (m, 5H), 5.79 (s, 1H), 3.65 (d, *J* = 12.3 Hz, 1H), 3.27 (d, *J* = 10.5 Hz, 1H), 1.38 (s, 11H); **^{13}C NMR** (101 MHz, $CDCl_3$) δ 154.08, 136.15, 128.43, 128.24, 126.29, 126.05 (q, *J* = 287.4 Hz), 80.44, 65.31 (q, *J* = 23.6 Hz), 46.75, 28.07; **^{19}F NMR** (376 MHz, $CDCl_3$) δ -72.00; **HRMS** (ESI): calcd for $[C_{14}H_{20}F_3N_2O_2]$ requires 305.14714; found 305.14733; enantiomeric excess: 80%, determined by HPLC (Chiralpak IF, hexane/i-PrOH 95/5,

flow rate 1.0 mL/min; t_{major} = 9.5 min, t_{mjnor} = 11.6 min, λ = 254 nm); $[\alpha]_D^{25}$ = 24.9 (*c* = 0.5, $CHCl_3$).

Compound **5b**

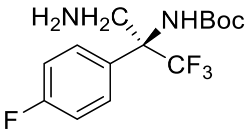

2.2 g, 68% yield (over two steps); **^{1}H NMR** (400 MHz, $CDCl_3$) δ 7.43-7.40 (m, 2H), 7.09-7.05 (m, 2H), 5.84 (s, 1H), 3.60 (d, *J* = 13.9 Hz, 1H), 3.23 (d, *J* = 13.5 Hz, 1H), 1.48 (s, 2H), 1.38 (s, 9H);

^{13}C NMR (101 MHz, $CDCl_3$) δ 162.45 (d, *J* = 247.8 Hz), 153.99, 128.22 (d, *J* = 8.2 Hz), 126.30, 125.88 (d, *J* = 287.5 Hz), 115.34 (d, *J* = 21.6 Hz), 80.63, 64.71 (q, *J* = 25.2 Hz), 46.90, 28.06; **^{19}F NMR** (376 MHz, $CDCl_3$) δ -72.07, -114.05; **HRMS** (ESI): calcd for [$C_{14}H_{19}F_4N_2O_2$] requires 323.13772; found 323.13723; $[\alpha]_D^{25}$ = 22.0 (*c* = 0.5, $CHCl_3$).

Compound **5c**

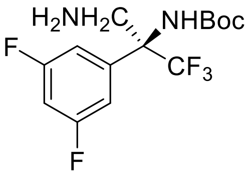

1.86 g, 55% yield (over two steps, after recrystallization); **^{1}H NMR** (400 MHz, $CDCl_3$) δ 6.93 (d, *J* = 7.4 Hz, 2H), 6.73 (t, *J* = 8.5 Hz, 1H), 5.96 (s, 1H), 3.51 (d, *J* = 13.8 Hz, 1H), 3.18 (d, *J* = 12.2 Hz, 1H), 2.31 (s, 2H), 1.31 (s, 9H); **^{13}C NMR** (101 MHz, $CDCl_3$) δ 162.93 (dd, *J* = 248.5, 12.8 Hz), 153.94, 140.01, 125.39 (q, *J* = 287.4 Hz), 109.91 (dd, *J* = 27.2, 1.3 Hz), 103.91 (t, *J* = 25.3 Hz), 81.16, 64.58 (q, *J* = 24.5 Hz), 46.60, 28.01; **^{19}F NMR** (376 MHz, $CDCl_3$) δ -71.72, -108.80; **HRMS** (ESI): calcd for [$C_{14}H_{18}F_5N_2O_2$] requires 341.12830; found 341.12764; enantiomeric excess: >99%, determined by HPLC (Chiralpak IF, hexane/i-PrOH 95/5, flow rate 1.0 mL/min; t_{major} = 5.3 min, t_{mjnor} = 6.5 min, λ = 254 nm); $[\alpha]_D^{25}$ = 23.8 (*c* = 0.5, $CHCl_3$).

Compound **5d**

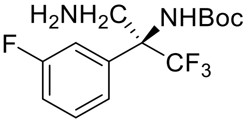

2.3 g, 70% yield (over two steps); **¹H NMR** (400 MHz, $CDCl_3$) δ 7.30-7.25 (m, 1H), 7.16-7.08 (m, 2H), 6.99-6.94 (m, 1H), 5.82 (s, 1H), 3.51 (d, *J* = 13.8 Hz, 1H), 3.15 (d, *J* = 13.3 Hz, 1H), 1.45 (s, 2H), 1.31 (s, 9H); **¹³C NMR** (101 MHz, $CDCl_3$) δ 162.74 (d, *J* = 245.7 Hz), 153.98, 138.84, 129.91 (d, *J* = 8.2 Hz), 125.77 (d, *J* = 287.5 Hz), 121.97, 115.21 (d, *J* = 21.1 Hz), 113.88 (dd, *J* = 23.9 Hz), 80.71, 64.86 (d, *J* = 25.8 Hz), 47.01, 28.04; **¹⁹F NMR** (376 MHz, $CDCl_3$) δ -72.33, -112.25; **HRMS** (ESI): calcd for [$C_{14}H_{19}F_4N_2O_2$] requires 323.13772; found 323.13833.

$[\alpha]_D^{25}$ = 19.9 (*c* = 0.5, $CHCl_3$).

Compound **5e**

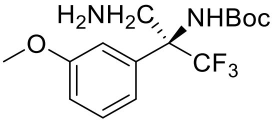

2.6 g, 77% yield (over two steps); **¹H NMR** (400 MHz, $CDCl_3$) δ 7.30 (t, *J* = 8.1 Hz, 1H), 7.01 (m, 2H), 6.88 (dd, *J* = 8.2, 2.2 Hz, 1H), 5.68 (s, 1H), 3.80 (s, 3H), 3.64 (d, *J* = 12.7 Hz, 1H), 3.28 (d, *J* = 13.3 Hz, 1H), 1.54 (s, 2H), 1.40 (s, 9H); **¹³C NMR** (101 MHz, $CDCl_3$) δ 159.66, 154.04, 137.59, 129.48, 125.93 (d, *J* = 287.5 Hz), 118.60, 113.32, 112.90, 80.52, 65.24 (q, *J* = 24.5 Hz), 55.22, 46.64, 28.10; **¹⁹F NMR** (376 MHz, $CDCl_3$) δ -72.09; **HRMS** (ESI): calcd for [$C_{15}H_{22}F_3N_2O_3$] requires 335.15770; found 335.15755; $[\alpha]_D^{25}$ = 24.1 (*c* = 0.5, $CHCl_3$).

Compound **5f**

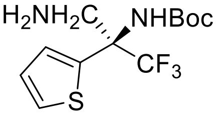

2.2 g, 71% yield (over two steps); **¹H NMR** (400 MHz, $CDCl_3$) δ 7.31 (d, *J* = 5.1 Hz, 1H), 7.13 (d, *J* = 3.1 Hz, 1H), 7.02-7.00 (m, 1H), 5.77 (s, 1H), 3.75 (d, *J* = 13.9 Hz, 1H), 3.42 (d, *J* = 14.0 Hz, 1H), 2.54 (s, 2H), 1.42 (s, 9H); **¹³C NMR** (101 MHz, $CDCl_3$) δ 153.90, 139.35, 127.16, 126.22, 125.82, 125.19 (q, *J* = 287.2 Hz), 81.04, 64.25 (q, *J* = 28.2 Hz), 46.26, 28.10.

^{19}F NMR (376 MHz, $CDCl_3$) δ -74.85; **HRMS** (ESI): calcd for [$C_{12}H_{18}F_3N_2O_2S$] requires 311.10356; found 311.10371; $[\alpha]_D^{25}$ = 8.1 (c = 0.5, $CHCl_3$).

Compound **6a**

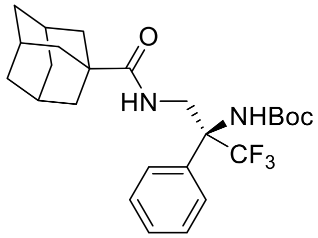

307.9 mg, 66% yield; **^{1}H NMR** (400 MHz, $CDCl_3$) δ 7.37-7.35 (m, 5H), 7.04 (s, 1H), 5.65 (s, 1H), 4.50 (s, 1H), 3.80 (dd, J = 14.3, 2.7 Hz, 1H), 1.96 (s, 3H), 1.69-1.60 (m, 12H), 1.45 (s, 9H); **^{13}C NMR** (101 MHz, $CDCl_3$) δ 178.21, 154.79, 133.38, 128.70, 128.32, 127.06, 125.12 (d, J = 286.4 Hz), 81.21, 65.86 (d, J = 25.5 Hz), 42.85, 40.57, 38.88, 36.46, 28.16, 28.05; **^{19}F NMR** (376 MHz, $CDCl_3$) δ -75.66; enantiomeric excess: 80%, determined by HPLC (Chiralpak AD-H and IC, hexane/i-PrOH 95/5, flow rate 1.0 mL/min; t_{major}= 18.6 min, t_{mjnor} = 21.3 min, λ = 254 nm); **HRMS** (ESI): calcd for [$C_{25}H_{34}F_3N_2O_3$] requires 467.25160; found 467.25122; $[\alpha]_D^{25}$ = -21.6 (c = 0.5, $CHCl_3$).

Compound **6b**

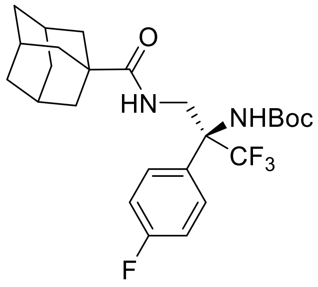

290.7 mg, 60% yield; **^{1}H NMR** (400 MHz, $CDCl_3$) δ 7.36-7.32 (m, 2H), 7.02-7.06 (m, 3H), 5.73 (s, 1H), 4.50 (s, 1H), 3.71 (dd, J = 14.3, 2.3 Hz, 1H), 1.95 (s, 3H), 1.71–1.59 (m, 12H), 1.43 (s, 9H); **^{13}C NMR** (101 MHz, $CDCl_3$) δ 178.28, 162.80 (d, J = 248.6 Hz), 154.78, 129.13 (d, J = 8.7 Hz), 128.29, 124.95 (q, J = 286.1 Hz), 115.27 (d, J = 21.7 Hz), 81.40, 65.54 (q, J = 25.9 Hz), 42.90, 40.57, 38.88, 36.42, 28.13, 28.01; **^{19}F NMR** (376 MHz, $CDCl_3$) δ -76.04, -113.24; **HRMS** (ESI): calcd for [$C_{25}H_{33}F_4N_2O_3$] requires 485.24218; found 485.24222; $[\alpha]_D^{25}$ = -27.2 (c = 0.5, $CHCl_3$).

Compound **6c**

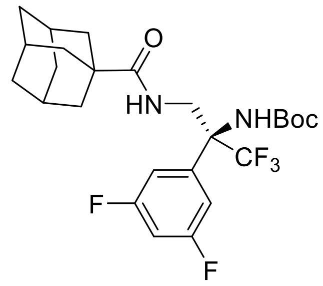

311.6 mg, 62% yield; ^{1}**H NMR** (500 MHz, CDCl$_3$) δ 6.93-6.92 (d, *J* = 7.2 Hz, 3H), 6.81-6.77 (m, 1H), 5.92 (s, 1H), 4.37 (s, 1H), 3.72 (dd, *J* = 14.5, 3.9 Hz, 1H), 1.97 (s, 3H), 1.70-1.61 (m, 12H), 1.43 (s, 9H); ^{13}**C NMR** (101 MHz, CDCl$_3$) δ 178.54, 162.80 (dd, *J* = 248.6, 12.7 Hz), 154.53, 137.73, 124.72 (q, *J* = 286.9 Hz), 110.54 (d, *J* = 27.3 Hz), 104.28 (t, *J* = 25.2 Hz), 81.64, 65.81 (q, *J* = 26.1 Hz), 43.03, 40.59, 38.87, 36.39, 28.09, 28.00; ^{19}**F NMR** (376 MHz, CDCl$_3$) δ -74.64, -108.74; **HRMS** (ESI): calcd for [$C_{25}H_{32}F_5N_2O_3$] requires 503.2328; found 485. 503.2321; enantiomeric excess: 99%, determined by HPLC (Chiralpak ADH and IC, hexane/i-PrOH 97/3, flow rate 1.0 mL/min; t_{mjnor}= 17.1 min, t_{major} = 20.6 min, λ = 254 nm); $[\alpha]_D^{25}$ = -14.0 (*c* = 0.5, CHCl$_3$).

Compound **6d**

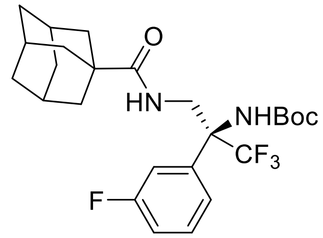

305.3 mg, 63% yield; ^{1}**H NMR** (400 MHz, CDCl$_3$) δ 7.37-7.31 (m, 1H), 7.18 (d, *J* = 7.9 Hz, 1H), 7.09-7.03 (m, 2H), 6.89 (s, 1H), 5.73 (s, 1H), 4.45 (s, 1H), 3.77 (dd, *J* = 14.4, 3.2 Hz, 1H), 2.79 (s, 1H), 1.97 (s, 3H), 1.67 (m, 12H), 1.41 (d, *J* = 30.1 Hz, 9H); ^{13}**C NMR** (101 MHz, CDCl$_3$) δ 178.41, 162.65 (d, *J* = 245.9 Hz), 154.69, 129.88 (d, *J* = 8.1 Hz), 128.33, 124.91 (d, *J* = 286.7 Hz), 122.82, 115.73 (d, *J* = 21.0 Hz), 114.52 (d, *J* = 24.6 Hz), 81.50, 65.74 (d, *J* = 26.2 Hz), 42.95, 40.60, 38.88, 36.43, 28.14, 28.03; ^{19}**F NMR** (376 MHz, CDCl$_3$) δ -75.21, -112.25; **HRMS** (ESI): calcd for [$C_{25}H_{33}F_4N_2O_3$] requires 485.24218; found 485.24238; $[\alpha]_D^{25}$ = -27.8 (*c* = 0.5, CHCl$_3$).

Compound **6e**

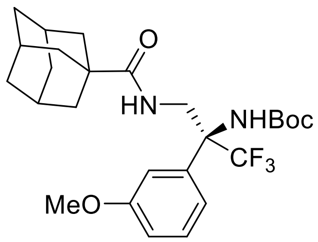

312.8 mg, 63% yield; **^{1}H NMR** (400 MHz, $CDCl_3$) δ 7.26-7.22 (m, 1H), 7.02-6.89 (m, 3H), 6.85 (d, *J* = 8.2 Hz, 1H), 5.63 (s, 1H), 4.43 (s, 1H), 3.77-3.74 (m, 4H), 1.93 (s, 3H), 1.66-1.57 (m, 12H), 1.42 (s, 9H); **^{13}C NMR** (101 MHz, $CDCl_3$) δ 178.21, 159.53, 154.75, 135.02, 129.28, 125.09 (d, *J* = 286.8 Hz), 119.21, 114.36, 113.01, 81.19, 65.91 (d, *J* = 25.4 Hz), 55.19, 42.77, 40.56, 38.87, 36.46, 28.17, 28.05; **^{19}F NMR** (376 MHz, $CDCl_3$) δ -75.40; **HRMS** (ESI): calcd for [$C_{26}H_{36}F_3N_2O_4$] requires 497.2622; found 497.2618; $[\alpha]_D^{25}$ = -16.5 (*c* = 0.5, $CHCl_3$).

Compound **6f**

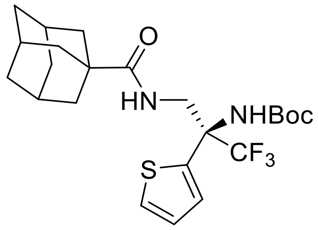

311.9 mg, 66% yield; **^{1}H NMR** (400 MHz, $CDCl_3$) δ 7.28 (s, 1H), 7.07 (d, *J* = 3.0 Hz, 1H), 6.99 (t, *J* = 4.3 Hz, 1H), 6.83 (s, 1H), 5.71 (s, 1H), 4.33 (dd, *J* = 14.8, 7.2 Hz, 1H), 3.94 (dd, *J* = 14.2, 4.6 Hz, 1H), 1.97 (s, 3H), 1.69 (m, 13H), 1.45 (s, 9H); **^{13}C NMR** (101 MHz, $CDCl_3$) δ 178.16, 154.31, 138.31, 127.38, 126.69, 125.71, 124.66 (q, *J* = 286.8 Hz), 81.31, 64.46 (q, *J* = 27.1 Hz), 42.77, 40.59, 38.91, 36.46, 28.12, 28.06; **^{19}F NMR** (376 MHz, $CDCl_3$) δ -76.50; **HRMS** (ESI): calcd for [$C_{23}H_{32}F_3N_2O_3S$] requires 473.20802; found 473.20829; $[\alpha]_D^{25}$ = -24.0 (*c* = 0.5, $CHCl_3$).

Compound **7a**

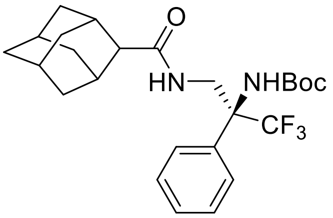

303.3 mg, 65% yield; **^{1}H NMR** (400 MHz, $CDCl_3$) δ 7.43-7.32 (m, 5H), 7.07 (s, 1H), 5.71 (s, 1H), 4.57 (s, 1H), 3.85 (dd, *J* = 14.2, 2.6 Hz, 1H), 2.36 (s, 1H), 2.23 (s, 1H), 2.10 (s, 1H), 1.91-1.55 (m, 12H), 1.44 (s, 9H); **^{13}C NMR** (101 MHz, $CDCl_3$) δ 174.62, 154.84, 133.45, 128.70, 128.44, 127.04, 125.12 (d, *J* = 286.5 Hz), 81.24, 65.92 (d, *J* = 25.8 Hz), 49.91, 42.91, 38.22, 38.19, 37.31, 33.53, 33.10, 32.80, 29.53, 28.13, 27.40, 27.25; **^{19}F NMR** (376 MHz, $CDCl_3$) δ -75.39; enantiomeric excess: 81%, determined by HPLC (Chiralpak AD-H and IC, hexane/i-PrOH 95/5, flow rate 1.0 mL/min; t_{mjnor}= 18.7 min, t_{major} = 20.2 min, λ = 254 nm); **HRMS** (ESI): calcd for [$C_{25}H_{34}F_3N_2O_3$] requires 467.25160; found 467.25130; $[\alpha]_D^{25}$ = -12.2 (*c* = 0.5, $CHCl_3$).

Compound **7b**

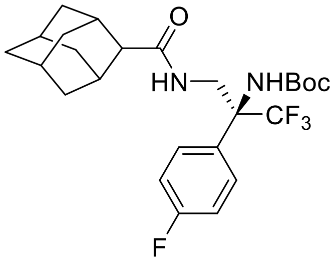

290.7 mg, 60% yield; **^{1}H NMR** (400 MHz, $CDCl_3$) δ 7.40-7.36 (m, 2H), 7.08-7.01 (m, 3H), 5.78 (s, 1H), 4.56 (s, 1H), 3.77 (dd, *J* = 14.4, 3.1 Hz, 1H), 2.34 (s, 1H), 2.21 (s, 1H), 2.09 (s, 1H), 1.96-1.50 (m, 12H), 1.43 (s, 9H); **^{13}C NMR** (101 MHz, $CDCl_3$) δ 174.64, 162.83 (d, *J* = 248.6 Hz), 154.74, 129.09 (d, *J* = 8.3 Hz), 128.39, 124.99 (q, *J* = 286.7 Hz), 115.36 (d, *J* = 21.7 Hz), 81.36, 65.67 (q, *J* = 25.7 Hz), 49.95, 43.00, 38.22, 38.19, 37.28, 33.13, 32.84, 29.54, 28.09, 27.40, 27.24; **^{19}F NMR** (376 MHz, $CDCl_3$) δ -75.58, -113.36; **HRMS** (ESI): calcd for [$C_{25}H_{33}F_4N_2O_3$] requires 485.24218; found 485.24222; $[\alpha]_D^{25}$ = -25.0 (*c* = 0.5, $CHCl_3$).

Compound **7c**

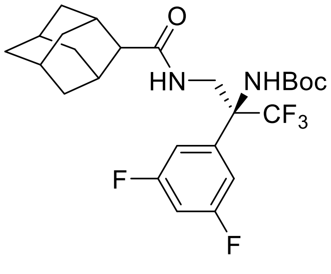

316.6 mg, 63% yield; **^{1}H NMR** (500 MHz, $CDCl_3$) δ 6.96-6.95 (m, 3H), 6.79-6.75 (m,

1H), 6.05 (s, 1H), 4.35 (s, 1H), 3.78 (dd, *J* = 14.5, 4.3 Hz, 1H), 2.38 (s, 1H), 2.21 (s, 1H), 2.11 (s, 1H), 1.55-1.85 (m, 12H), 1.41 (s, 9H); **^{13}C NMR** (101 MHz, $CDCl_3$) δ 175.13, 162.87 (dd, *J* = 248.8, 12.7 Hz), 154.46, 138.08, 124.77 (q, *J* = 287.3 Hz), 110.46 (d, *J* = 27.0 Hz), 104.18 (t, *J* = 25.2 Hz), 81.47, 65.91 (q, *J* = 25.7 Hz), 49.93, 43.34, 38.20, 38.17, 37.23, 33.06, 32.89, 29.66, 28.03, 27.38, 27.28; **^{19}F NMR** (376 MHz, $CDCl_3$) δ -73.92, -108.68; enantiomeric excess: 99%, determined by HPLC (Chiralpak AD-H, hexane/i-PrOH 95/5, flow rate 1.0 mL/min; t_{mjnor}= 6.9 min, t_{major} = 7.8 min, λ = 254 nm); **HRMS** (ESI): calcd for [$C_{25}H_{32}F_5N_2O_3$] requires 503.2328; found 503.2321; $[\alpha]_D^{25}$ = -9.6 (*c* = 1.0, $CHCl_3$).

Compound **7d**

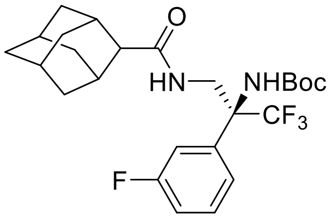

305.3 mg, 63% yield; **^{1}H NMR** (400 MHz, $CDCl_3$) δ 7.37-7.31 (m, 1H), 7.22 (d, *J* = 7.9 Hz, 1H), 7.14 (d, *J* = 10.4 Hz, 1H), 7.06-6.94 (m, 2H), 5.79 (s, 1H), 4.51 (s, 1H), 3.83 (dd, *J* = 14.4, 3.0 Hz, 1H), 2.38 (s, 1H), 2.23 (s, 1H), 2.11 (s, 1H), 1.85-1.68 (m, 12H), 1.44 (s, 9H); **^{13}C NMR** (101 MHz, $CDCl_3$) δ 174.84, 162.73 (d, *J* = 246.2 Hz), 154.68, 129.98 (d, *J* = 8.2 Hz), 128.43, 124.93 (d, *J* = 286.7 Hz), 122.79, 115.70 (d, *J* = 21.1 Hz), 114.50 (d, *J* = 23.7 Hz), 81.48, 65.82 (d, *J* = 25.8 Hz), 49.98, 38.23, 38.20, 37.28, 33.52, 33.10, 32.86, 29.60, 28.10, 27.41, 27.28; **^{19}F NMR** (376 MHz, $CDCl_3$) δ -74.87, -112.13; **HRMS** (ESI): calcd for [$C_{25}H_{33}F_4N_2O_3$] requires 485.24218; found 485.24224;

$[\alpha]_D^{25}$ = -14.4 (*c* = 0.5, $CHCl_3$).

Compound **7e**

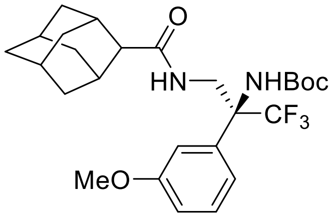

307.9 mg, 62% yield; **^{1}H NMR** (400 MHz, $CDCl_3$) δ 7.24-7.22 (m, 1H), 7.01-6.93 (m, 3H), 6.83 (dd, *J* = 8.1, 1.8 Hz, 1H), 5.67 (s, 1H), 4.50 (s, 1H), 3.81 (dd, *J* = 14.6, 3.3 Hz, 1H), 3.75 (s, 3H), 2.32 (s, 1H), 2.19 (s, 1H), 2.07 (s, 1H), 1.81-1.47 (m, 12H), 1.41 (s, 9H); **^{13}C NMR** (101 MHz, $CDCl_3$) δ 174.52, 159.63, 154.72, 135.22, 129.39, 125.12 (d, *J* = 286.8 Hz), 119.19, 114.13, 113.19, 81.14, 66.01 (d, *J* = 25.8 Hz), 55.20, 49.99, 42.85, 38.27, 38.23, 37.33, 33.07, 32.85, 29.61, 29.58, 28.14, 27.45, 27.33; **^{19}F NMR** (376 MHz, $CDCl_3$) δ -74.97; **HRMS** (ESI): calcd for [$C_{26}H_{36}F_3N_2O_4$] requires 497.2622; found 497.2614; $[\alpha]_D^{25}$ = -16.5 (*c* = 0.5, $CHCl_3$).

Compound **7f**

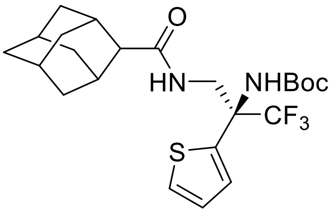

288.3 mg, 61% yield; **^{1}H NMR** (400 MHz, $CDCl_3$) δ 7.28 (d, *J* = 5.1 Hz, 1H), 7.12 (d, *J* = 3.1 Hz, 1H), 7.00 (t, *J* = 4.4 Hz, 1H), 6.87 (s, 1H), 5.76 (s, 1H), 4.39 (dd, *J* = 13.8, 7.5 Hz, 1H), 4.00 (dd, *J* = 14.3, 4.8 Hz, 1H), 2.38 (s, 1H), 2.24 (s, 1H), 2.14 (s, 1H), 1.74 (m, 12H), 1.44 (s, 9H); **^{13}C NMR** (101 MHz, $CDCl_3$) δ 174.58, 154.27, 138.39, 127.49, 126.77, 125.72, 124.67 (q, *J* = 287.0 Hz), 81.28, 64.50 (q, *J* = 27.1 Hz), 49.99, 42.91, 38.27, 38.25, 37.33, 33.11, 32.93, 29.70, 29.62, 28.10, 27.44, 27.33; **^{19}F NMR** (376 MHz, $CDCl_3$) δ -76.24; **HRMS** (ESI): calcd for [$C_{23}H_{32}F_3N_2O_3S$] requires 473.20802; found 473.20787; $[\alpha]_D^{25}$ = -15.0 (*c* = 1.0, $CHCl_3$).

Compound **8a**

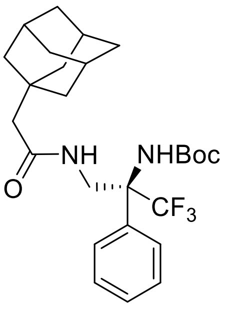

336.4 mg, 70% yield; **^{1}H NMR** (400 MHz, $CDCl_3$) δ 7.44-7.33 (m, 5H), 6.62 (s, 1H), 5.76 (s, 1H), 4.40 (s, 1H), 3.83 (dd, *J* = 14.4, 3.8 Hz, 1H), 1.90-1.86 (m, 5H), 1.71-1.50

(m, 12H), 1.44 (s, 9H); **^{13}C NMR** (101 MHz, $CDCl_3$) δ 171.61, 154.63, 133.65, 128.71, 128.42, 126.95, 125.17 (d, *J* = 286.9 Hz), 81.08, 65.88 (d, *J* = 25.8 Hz), 51.69, 43.41, 42.35, 36.67, 32.70, 28.61, 28.14; **^{19}F NMR** (376 MHz, $CDCl_3$) δ -74.25; enantiomeric excess: 79%, determined by HPLC (Chiralpak IF, hexane/i-PrOH 90/10, flow rate 1.0 mL/min; t_{mjnor}= 4.3 min, t_{major} = 5.0 min, λ = 254 nm); **HRMS** (ESI): calcd for [$C_{26}H_{36}F_3N_2O_3$] requires 481.26725; found 481.26725; $[\alpha]_D^{25}$ = -13.7 (*c* = 0.5, $CHCl_3$).

Compound **8b**

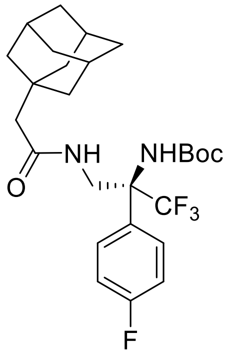

324.1 mg, 65% yield; **^{1}H NMR** (400 MHz, $CDCl_3$) δ 7.37-7.41 (m, 2H), 7.05 (t, *J* = 8.6 Hz, 2H), 6.71 (s, 1H), 5.87 (s, 1H), 4.37 (s, 1H), 3.75 (dd, *J* = 14.5, 3.6 Hz, 1H), 1.90 (s, 3H), 1.85 (s, 2H), 1.68-1.48 (m, 12H), 1.42 (s, 9H); **^{13}C NMR** (101 MHz, $CDCl_3$) δ 171.76, 162.80 (d, *J* = 248.5 Hz), 154.59, 128.99 (d, *J* = 8.1 Hz), 128.41, 125.03 (q, *J* = 287.0 Hz), 115.39 (d, *J* = 21.7 Hz), 81.25, 65.62 (q, *J* = 26.0 Hz), 51.61, 43.48, 42.36, 36.64, 32.70, 28.58, 28.11; **^{19}F NMR** (376 MHz, $CDCl_3$) δ -74.46, -113.28; **HRMS** (ESI): calcd for [$C_{26}H_{35}F_4N_2O_3$] requires 499.25783; found 499.25761; $[\alpha]_D^{25}$ = -16.5 (*c* = 0.5, $CHCl_3$).

Compound **8c**

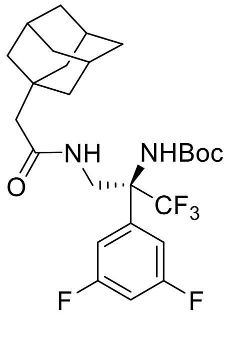

340.9 mg, 66% yield; **^{1}H NMR** (500 MHz, $CDCl_3$) δ 6.96 (d, *J* = 7.0 Hz, 2H), 6.79-6.75 (m, 2H), 6.22 (s, 1H), 4.12 (s, 1H), 3.75 (dd, *J* = 14.5, 4.7 Hz, 1H), 1.91-1.88 (m,

5H), 1.67-1.51 (m, 12H), 1.41 (s, 9H); **^{13}C NMR** (101 MHz, $CDCl_3$) δ 172.40, 162.87 (dd, *J* = 248.7, 12.7 Hz), 154.30, 138.24, 124.82 (q, *J* = 287.7 Hz), 110.35 (d, *J* = 27.0 Hz), 104.18 (t, *J* = 25.1 Hz), 81.32, 65.94 (q, *J* = 26.2 Hz), 51.38, 44.04, 42.41, 36.61, 32.77, 28.57, 28.05; **^{19}F NMR** (376 MHz, $CDCl_3$) δ -72.45, -108.65; **HRMS** (ESI): calcd for [$C_{26}H_{34}F_5N_2O_3$] requires 517.2484; found 517.2481. enantiomeric excess: 97%, determined by HPLC (Chiralpak AD-H, hexane/i-PrOH 95/5, flow rate 1.0 mL/min; t_{mjnor}= 6.9 min, t_{major} = 7.9 min, λ = 254 nm); $[\alpha]_D^{25}$ = -1.0 (*c* = 1.0, $CHCl_3$).

Compound **8d**

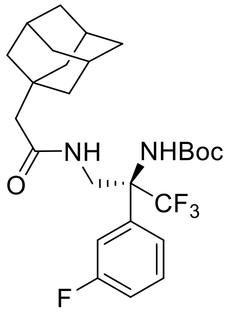

324.1 mg, 65% yield; **^{1}H NMR** (400 MHz, $CDCl_3$) δ 7.35 (q, *J* = 8.0 Hz, 1H), 7.22 (d, *J* = 7.9 Hz, 1H), 7.14 (d, *J* = 10.4 Hz, 1H), 7.05 (m, 1H), 5.90 (s, 1H), 3.81 (dd, *J* = 14.4, 3.8 Hz, 1H), 1.89 (m, 5H), 1.66 (d, *J* = 12.5 Hz, 5H), 1.59 (s, 2H), 1.50 (s, 5H), 1.43 (s, 9H);

^{13}C NMR (101 MHz, $CDCl_3$) δ 171.99, 162.72 (d, *J* = 246.1 Hz), 154.55, 129.98 (d, *J* = 8.1 Hz), 128.44, 127.13, 124.95 (q, *J* = 287.1 Hz), 122.68, 115.74 (d, *J* = 20.9 Hz), 114.46 (d, *J* = 24.2 Hz), 81.39, 65.75 (q, *J* = 25.5 Hz), 51.61, 48.54, 43.58, 42.37, 42.31, 36.71, 36.64, 32.74, 28.61, 28.59, 28.10; **HRMS** (ESI): calcd for [$C_{26}H_{35}F_4N_2O_3$] requires 499.25783; found 499.25693; **^{19}F NMR** (376 MHz, $CDCl_3$) δ -73.80, -112.09; $[\alpha]_D^{25}$ = -6.2 (*c* = 0.5, $CHCl_3$).

Compound **8e**

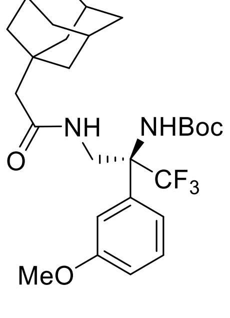

352.3 mg, 69% yield; **^{1}H NMR** (400 MHz, $CDCl_3$) δ 7.21 (d, J = 8.6 Hz, 1H), 6.95-6.91 (m, 2H), 6.82-6.80 (m, 1H), 6.58 (s, 1H), 5.71 (s, 1H), 4.32 (dd, J = 12.5, 6.2 Hz, 1H), 3.77-3.72 (m, 4H), 1.83-1.79 (m, 5H), 1.61-1.42 (m, 12H), 1.38 (s, 9H); **^{13}C NMR** (101 MHz, $CDCl_3$) δ 171.62, 159.61, 154.61, 135.21, 129.38, 125.12 (q, J = 286.9 Hz), 119.13, 114.20, 113.13, 81.08, 65.93 (q, J = 25.4 Hz), 55.19, 51.72, 43.28, 42.34, 36.65, 32.70, 28.61, 28.15; **^{19}F NMR** (376 MHz, $CDCl_3$) δ -74.12; **HRMS** (ESI): calcd for [$C_{27}H_{38}F_3N_2O_4$] requires 511.2778; found 511.2772; $[\alpha]_D^{25}$ = -8.6 (c = 0.5, $CHCl_3$).

Compound **8f**

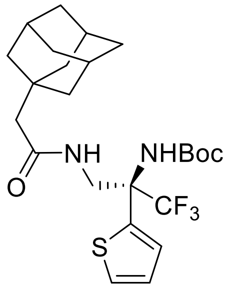

321.1 mg, 66% yield; **^{1}H NMR** (400 MHz, $CDCl_3$) δ 7.29 (d, J = 5.1 Hz, 1H), 7.13 (d, J = 3.0 Hz, 1H), 7.01 (t, J = 4.3 Hz, 1H), 6.47 (s, 1H), 5.81 (s, 1H), 4.28 (dd, J = 14.1, 7.6 Hz, 1H), 3.96 (dd, J = 14.3, 4.9 Hz, 1H), 1.90 (d, J = 10.7 Hz, 5H), 1.62 (m, 7H), 1.51 (s, 5H), 1.45 (s, 9H); **^{13}C NMR** (101 MHz, $CDCl_3$) δ 171.59, 154.15, 138.34, 127.43, 126.77, 125.73, 124.67 (q, J = 287.0 Hz), 81.22, 64.44 (q, J = 27.1 Hz), 51.71, 43.33, 42.35, 36.67, 32.68, 28.61, 28.12; **^{19}F NMR** (376 MHz, $CDCl_3$) δ -75.58; **HRMS** (ESI): calcd for [$C_{24}H_{34}F_3N_2O_3S$] requires 487.22367; found 487.22341; $[\alpha]_D^{25}$ = -9.7 (c = 0.5, $CHCl_3$).

Compound **9a**

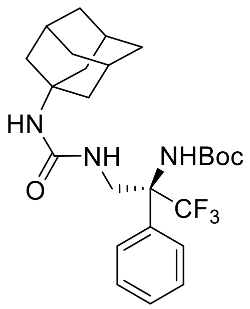

399.7 mg, 83% yield; **^{1}H NMR** (400 MHz, $CDCl_3$) δ 7.43-7.30 (m, 5H), 6.17 (s, 1H), 5.34 (s, 1H), 4.34 (s, 1H), 4.13 (s, 1H), 3.72 (d, J = 12.0 Hz, 1H), 2.03 (s, 3H), 1.88 (s,

6H), 1.64 (s, 6H), 1.41 (s, 9H); **^{13}C NMR** (101 MHz, $CDCl_3$) δ 157.39, 154.65, 128.29, 126.73, 125.49 (q, *J* = 286.7 Hz), 80.56, 66.28 (q, *J* = 25.1 Hz), 50.93, 45.31, 42.19, 36.33, 29.53, 28.14; **^{19}F NMR** (376 MHz, $CDCl_3$) δ -72.48; **HRMS** (ESI): calcd for [$C_{25}H_{35}F_3N_3O_3$] requires 482.26250; found 482.26178; enantiomeric excess: 78%, determined by HPLC (Chiralpak IF, hexane/i-PrOH 90/10, flow rate 1.0 mL/min; t_{mjnor}= 3.9 min, t_{major} = 5.3 min, λ = 254 nm); $[\alpha]_D^{25}$ = -6.9 (*c* = 0.5, $CHCl_3$).

Compound **9b**

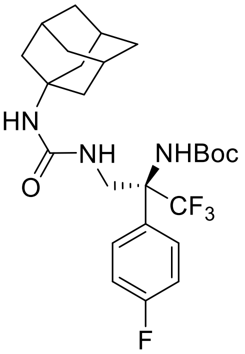

379.7 mg, 76% yield; **^{1}H NMR** (400 MHz, $CDCl_3$) δ 7.39-7.36 (m, 2H), 7.02 (t, *J* = 8.5 Hz, 2H), 6.44 (s, 1H), 5.49 (s, 1H), 4.58 (s, 1H), 3.99 (s, 1H), 3.60 (dd, *J* = 14.5, 4.1 Hz, 1H), 2.02 (s, 3H), 1.86 (s, 6H), 1.63 (s, 6H), 1.38 (s, 9H); **^{13}C NMR** (101 MHz, $CDCl_3$) δ 162.50 (d, *J* = 248.1 Hz), 157.55, 154.57, 128.62 (d, *J* = 7.0 Hz), 128.28, 125.40 (q, *J* = 288.5 Hz), 115.18 (d, *J* = 21.5 Hz), 80.68, 65.98 (q, *J* = 25.6 Hz), 50.91, 45.52, 42.16, 36.30, 29.50, 28.11; **^{19}F NMR** (376 MHz, $CDCl_3$) δ -72.31, -114.02; **HRMS** (ESI): calcd for [$C_{25}H_{34}F_4N_3O_3$] requires 500.25308; found 500.25312.
$[\alpha]_D^{25}$ = -11.2 (*c* = 0.5, $CHCl_3$).

Compound **9c**

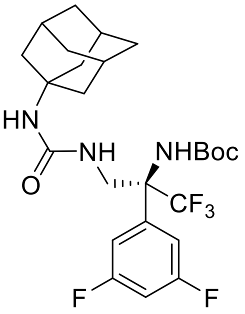

403.7 mg, 78% yield; **^{1}H NMR** (500 MHz, $CDCl_3$) δ 6.95-6.87 (t, *J* = 19.7 Hz, 3H), 6.77-6.73 (m, 1H), 5.58 (s, 1H), 4.82 (s, 1H), 3.84 (s, 1H), 3.55 (d, *J* = 8.1 Hz, 1H),

2.01 (s, 3H), 1.85 (s, 6H), 1.62 (s, 6H), 1.40 (s, 9H); **^{13}C NMR** (101 MHz, $CDCl_3$) δ 162.80 (dd, *J* = 248.1, 12.7 Hz), 157.91, 154.43, 139.01, 125.15 (q, *J* = 287.3 Hz), 109.94 (d, *J* = 26.0 Hz), 103.67 (t, *J* = 23.6 Hz), 80.81, 66.31 (q, *J* = 26.9 Hz), 50.98, 45.86, 42.06, 36.29, 29.48, 28.05; **^{19}F NMR** (376 MHz, $CDCl_3$) δ -70.41, -109.00; **HRMS** (ESI): calcd for [$C_{25}H_{33}F_5N_3O_3$] requires 518.2437; found 518.2430; enantiomeric excess: 99%, determined by HPLC (Chiralpak IF and IC, hexane/i-PrOH 96/4, flow rate 1.0 mL/min; t_{mjnor}= 12.9 min, t_{major} = 16.0 min, λ = 254 nm); $[\alpha]_D^{25}$ = -2.9 (*c* = 0.5, $CHCl_3$).

Compound **9d**

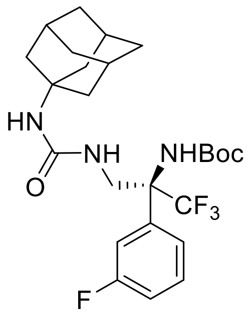

394.6 mg, 79% yield; **^{1}H NMR** (400 MHz, $CDCl_3$) δ 7.37-7.32 (m, 1H), 7.22 (d, *J* = 7.7 Hz, 1H), 7.15 (d, *J* = 10.7 Hz, 1H), 7.04 (t, *J* = 8.0 Hz, 1H), 6.16 (s, 1H), 5.39 (s, 1H), 4.16 (s, 2H), 3.73 (d, *J* = 14.3 Hz, 1H), 2.05 (s, 3H), 1.88 (s, 6H), 1.65 (s, 6H), 1.42 (s, 9H); **^{13}C NMR** (101 MHz, $CDCl_3$) δ 163.94, 154.57, 129.89 (d, *J* = 8.2 Hz), 125.15 (q, *J* = 287.3 Hz), 122.57, 115.42 (d, *J* = 19.9 Hz), 114.35 (d, *J* = 24.4 Hz), 109.99, 81.02, 66.00 (q, *J* = 26.8 Hz), 51.16, 42.14, 36.27, 29.65, 29.51, 28.14; **^{19}F NMR** (376 MHz, $CDCl_3$) δ -72.95, -112.34; **HRMS** (ESI): calcd for [$C_{25}H_{34}F_4N_3O_3$] requires 500.25308; found 500.25302; $[\alpha]_D^{25}$ = -1.5 (*c* = 0.5, $CHCl_3$).

Compound **9e**

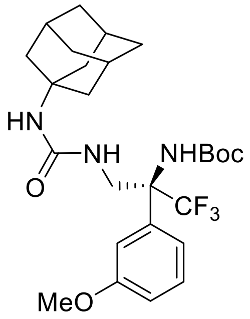

399.0 mg, 78% yield; **^{1}H NMR** (400 MHz, $CDCl_3$) δ 7.24 (dd, *J* = 9.5, 6.5 Hz, 1H),

6.96 (m, 2H), 6.83 (d, *J* = 7.1 Hz, 1H), 6.21 (s, 1H), 5.32 (s, 1H), 4.38 (s, 1H), 4.05 (s, 1H), 3.75 (s, 3H), 3.67 (dd, *J* = 14.4, 3.1 Hz, 1H), 2.00 (s, 3H), 1.85 (s, 6H), 1.61 (s, 6H), 1.38 (s, 9H); ^{13}**C NMR** (101 MHz, $CDCl_3$) δ 159.56, 157.29, 154.60, 136.20, 129.28, 125.41 (d, *J* = 287.6 Hz), 118.97, 113.97, 112.80, 80.60, 66.30 (d, *J* = 25.5 Hz), 55.19, 50.97, 45.18, 42.19, 36.32, 29.52, 28.16; ^{19}F NMR (376 MHz, $CDCl_3$) δ -72.45.

HRMS (ESI): calcd for [$C_{26}H_{37}F_3N_3O_4$] requires 512.2731; found 512.2725.

$[\alpha]_D^{25}$ = -2.4 (*c* = 0.5, $CHCl_3$).

Compound **9f**

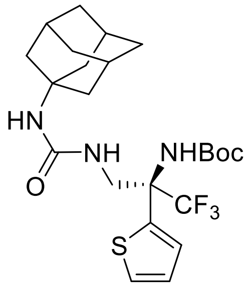

390.1 mg, 80% yield; ^{1}**H NMR** (400 MHz, $CDCl_3$) δ 7.27 (s, 1H), 7.07 (d, *J* = 3.1 Hz, 1H), 6.98 (m, 1H), 6.37 (s, 1H), 5.31 (s, 1H), 4.54 (s, 1H), 3.88 (td, *J* = 14.4, 5.7 Hz, 2H), 2.03 (s, 3H), 1.89 (s, 6H), 1.64 (s, 6H), 1.40 (s, 9H); ^{13}**C NMR** (101 MHz, $CDCl_3$) δ 157.37, 154.23, 138.99, 127.11, 126.14, 125.43, 124.94 (q, *J* = 287.7 Hz), 80.69, 64.66 (q, *J* = 27.0 Hz), 50.96, 45.37, 42.19, 36.34, 29.52, 28.16; ^{19}**F NMR** (376 MHz, $CDCl_3$) δ -73.97.

HRMS (ESI): calcd for [$C_{23}H_{33}F_3N_3O_3S$] requires 488.22909; found 488.22921.

$[\alpha]_D^{25}$ = -1.9 (*c* = 0.5, $CHCl_3$).

Preparation of the catalysts[1-3]

The catalysts used in this reaction were prepared according to the previous references, which included (thio)urea ammonium salts and amide ammonium salts.

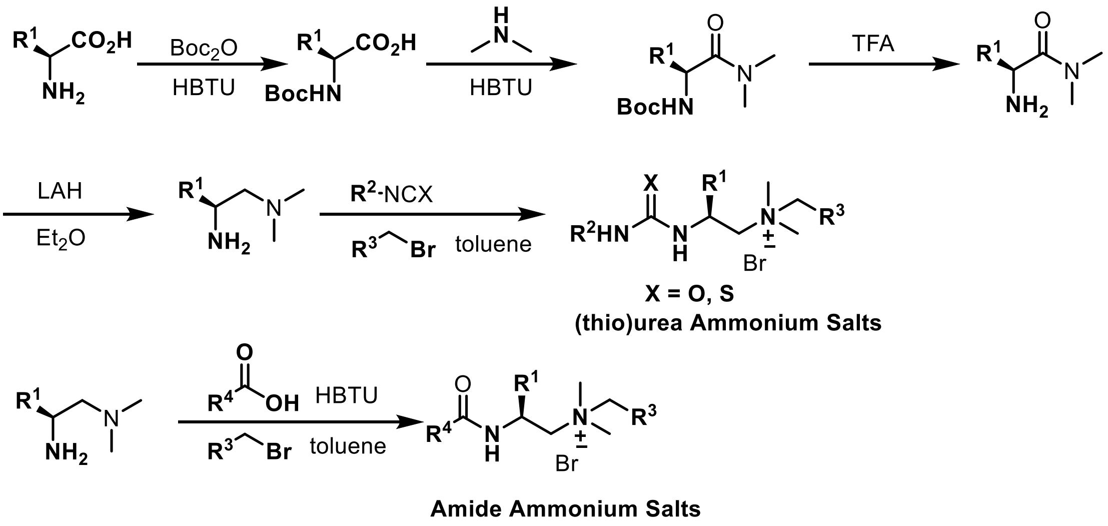

References

[1] H.-Y. Wang, J.-X. Zhang, D.-D. Cao and G. Zhao, *ACS Catal.*, **2013**, *3*, 2218-2221.

[2] H.-Y. Wang, Z. Chai and G. Zhao, *Tetrahedron*, **2013**, *69*, 5104-5111.

[3] J.-X. Zhang, H.-Y. Wang, Q.-W. Jin, C.-W. Zheng, G. Zhao and Y.-J. Shang, *Org. Lett.*, **2016**, *18*, 4774-4777.

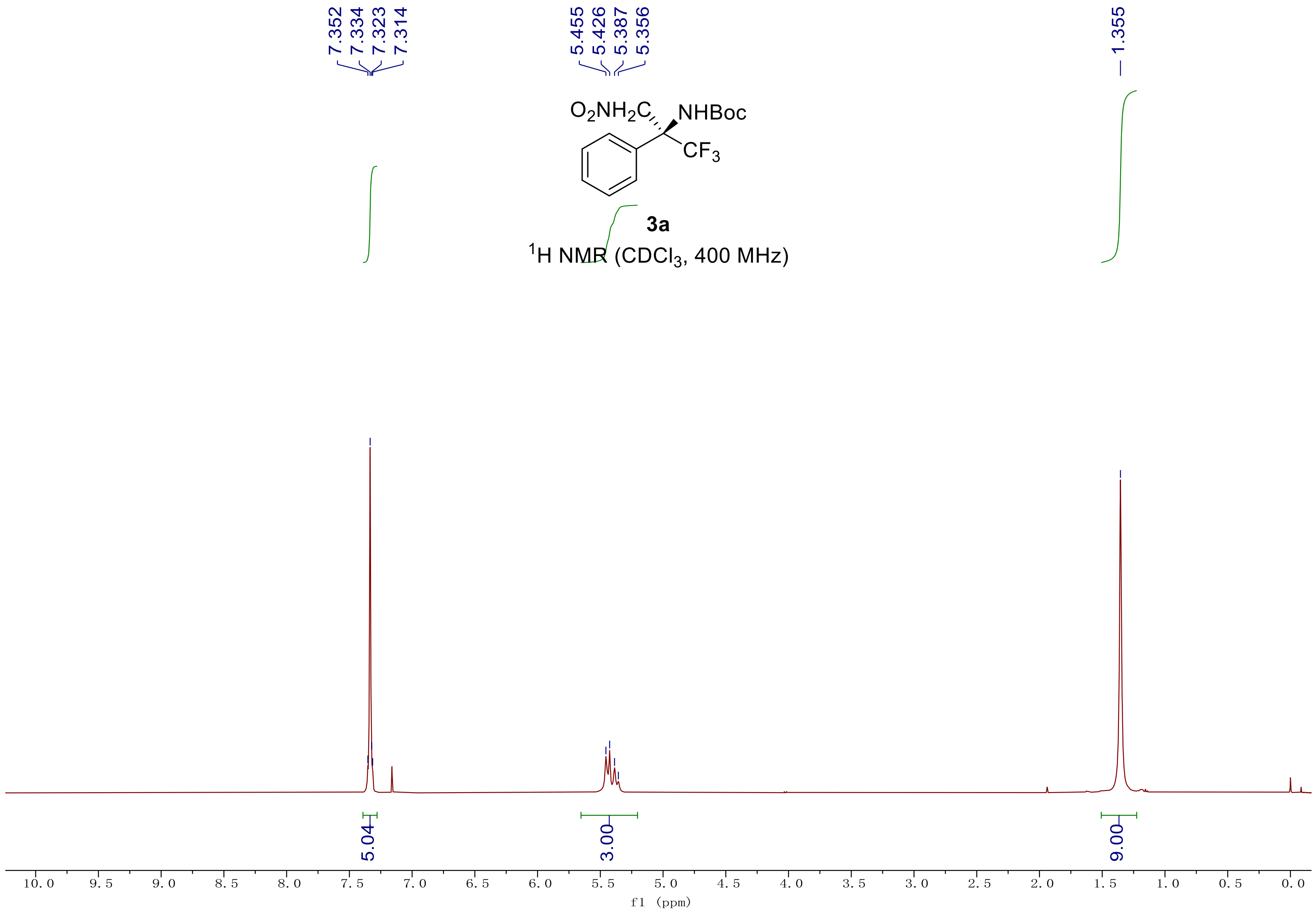
7.352
7.334
7.323
7.314
5.455
5.426
5.387
5.356
1.355
O2NH2C
NHBoc
CF3
3a
1H NMR (CDCl3, 400 MHz)
5.04
3.00
9.00
10.0 9.5 9.0 8.5 8.0 7.5 7.0 6.5 6.0 5.5 5.0 4.5 4.0 3.5 3.0 2.5 2.0 1.5 1.0 0.5 0.0
f1 (ppm)

O_2NH_2C NHBoc CF_3 (structure)

3a

^{13}C NMR ($CDCl_3$, 101 MHz)

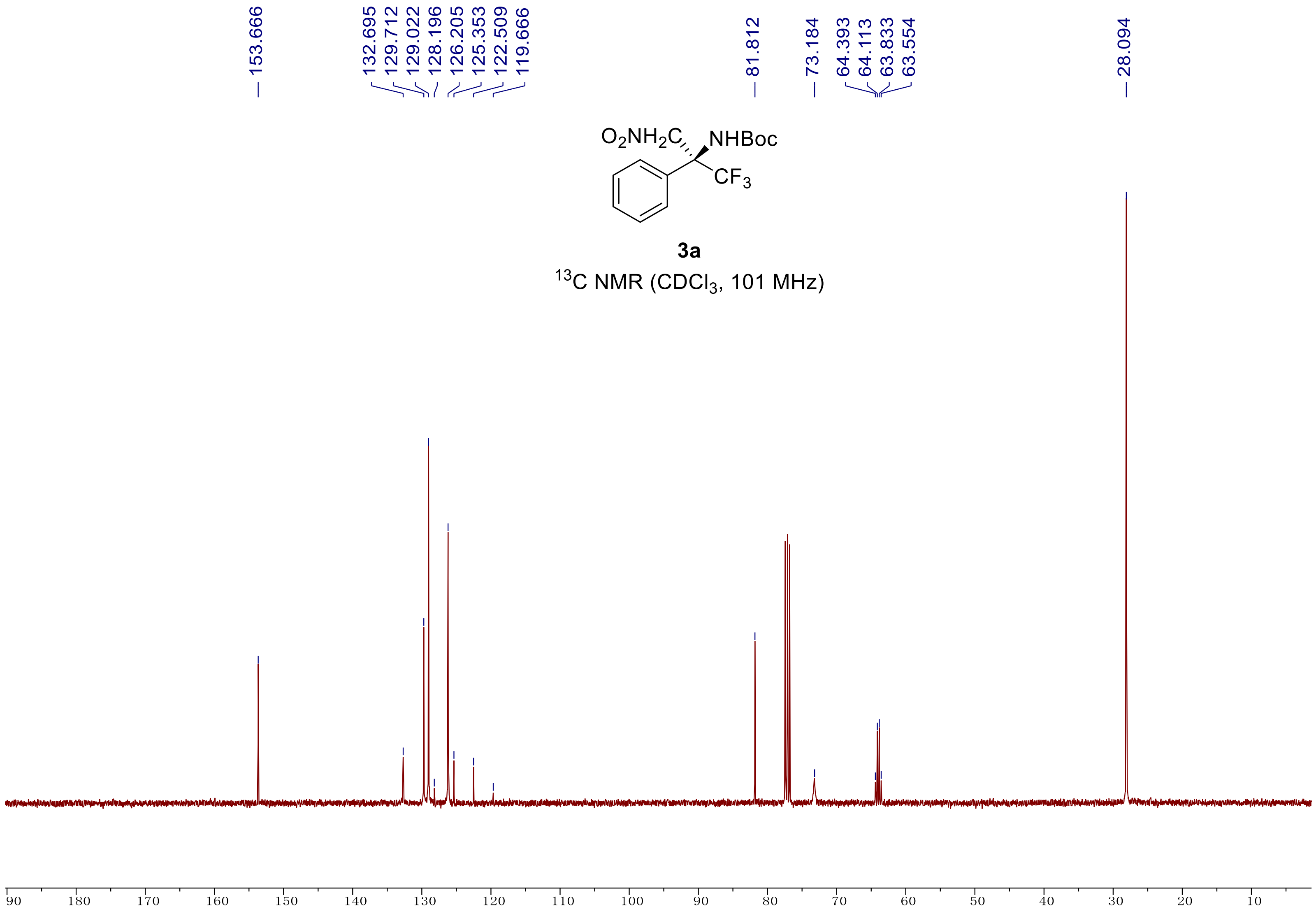

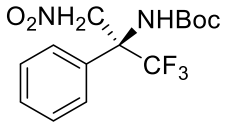

3a

^{19}F NMR ($CDCl_3$, 376 MHz)

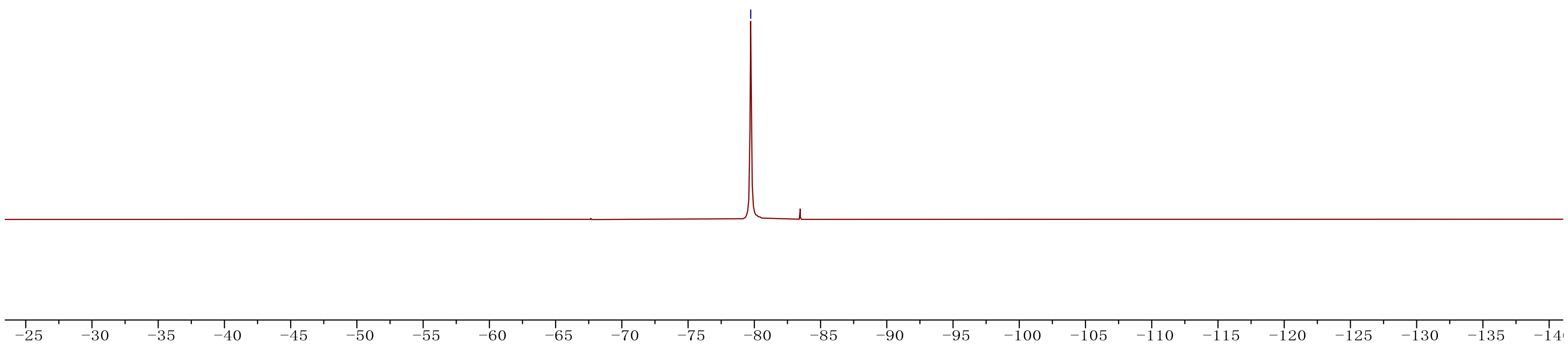

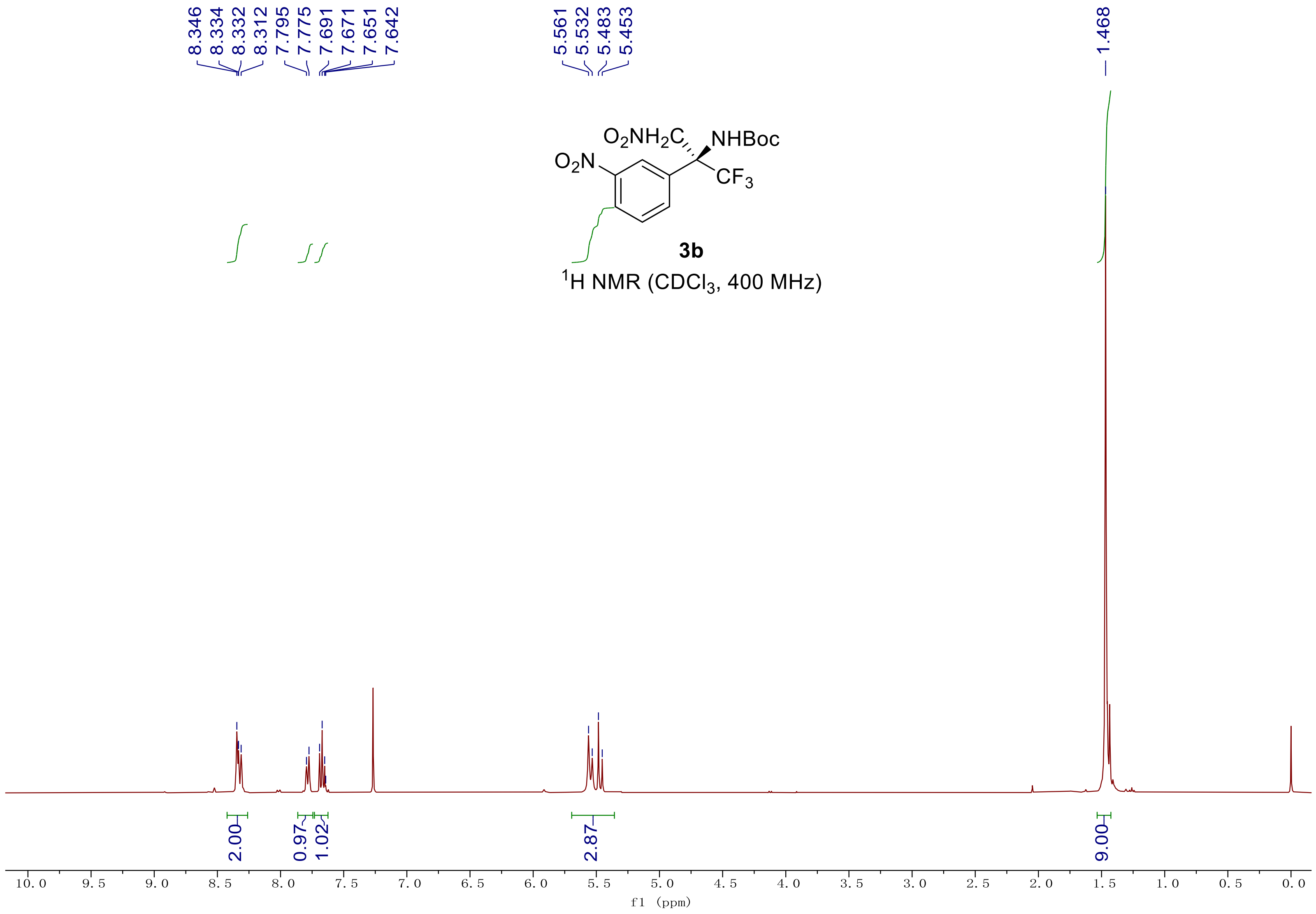
8.346
8.334
8.332
8.312
7.795
7.775
7.691
7.671
7.651
7.642
5.561
5.532
5.483
5.453
1.468
O_2NH_2C
NHBoc
O_2N
CF_3
3b
^1H NMR ($CDCl_3$, 400 MHz)
2.00
0.97
1.02
2.87
9.00
10.0
9.5
9.0
8.5
8.0
7.5
7.0
6.5
6.0
5.5
5.0
4.5
4.0
3.5
3.0
2.5
2.0
1.5
1.0
0.5
0.0
f1 (ppm)

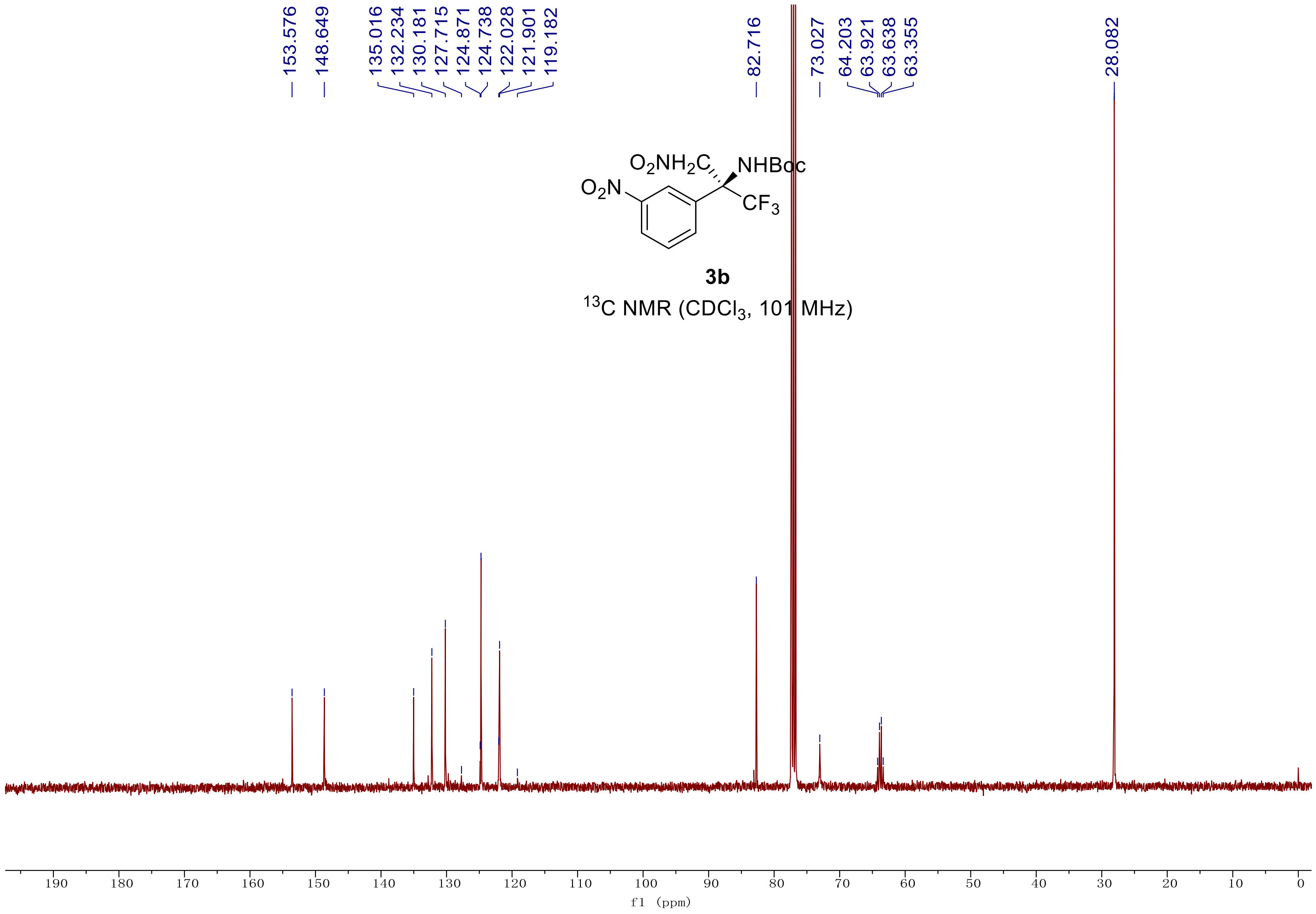

153.576
148.649
135.016
132.234
130.181
127.715
124.871
124.738
122.028
121.901
119.182
82.716
73.027
64.203
63.921
63.638
63.355
28.082
O2NH2C
NHBoc
O2N
CF3
3b
13C NMR (CDCl3, 101 MHz)
f1 (ppm)

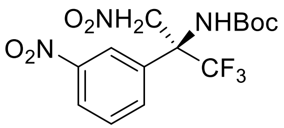

3b

^{19}F NMR ($CDCl_3$, 376 MHz)

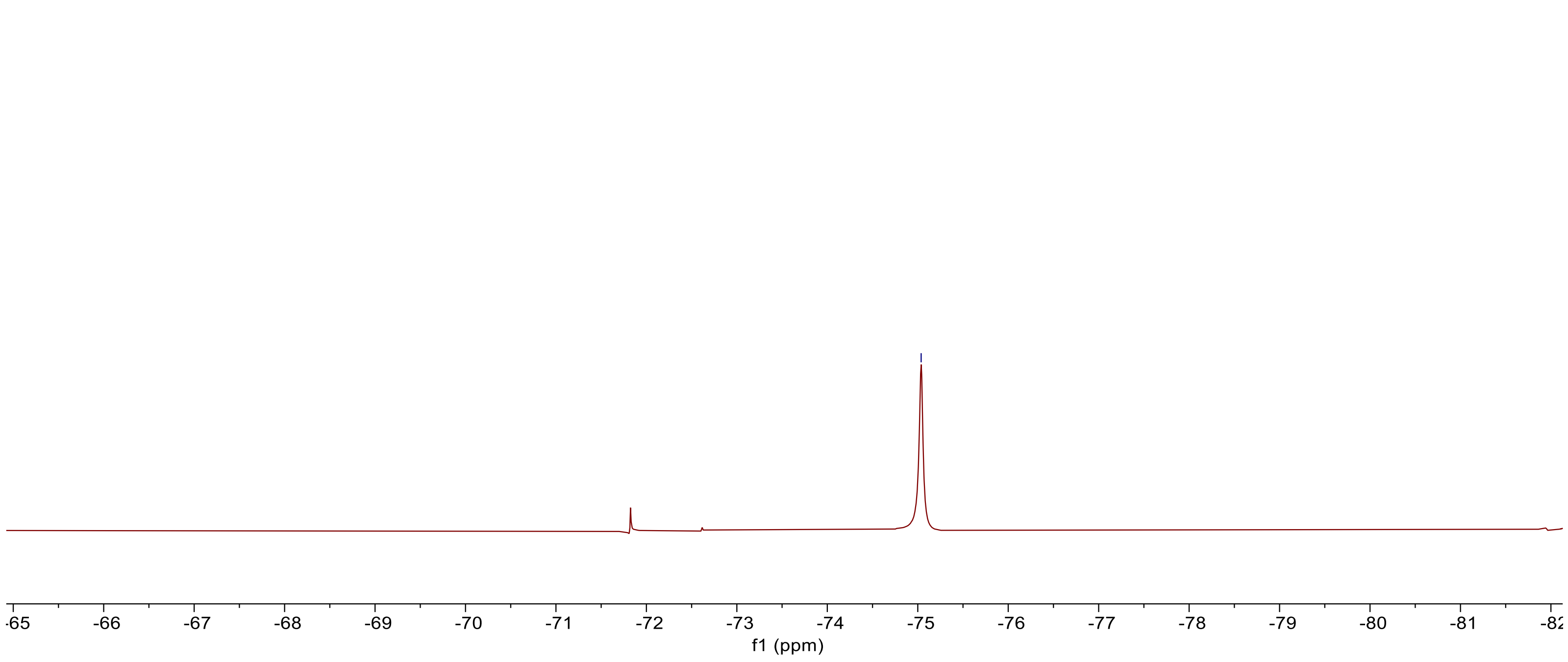

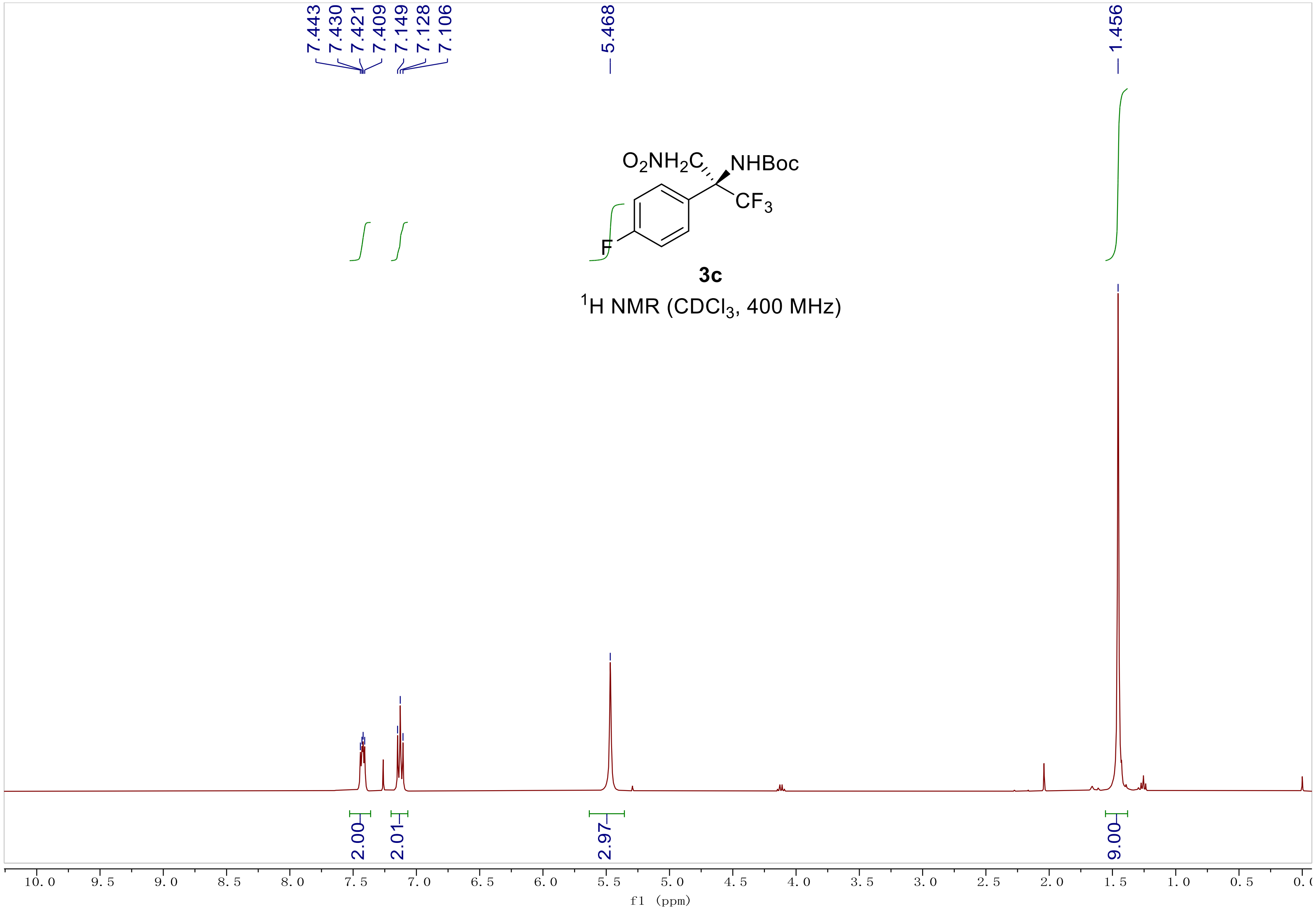
7.443
7.430
7.421
7.409
7.149
7.128
7.106
5.468
1.456
O2NH2C
NHBoc
CF3
F
3c
1H NMR (CDCl3, 400 MHz)
2.00
2.01
2.97
9.00
10.0 9.5 9.0 8.5 8.0 7.5 7.0 6.5 6.0 5.5 5.0 4.5 4.0 3.5 3.0 2.5 2.0 1.5 1.0 0.5
f1 (ppm)

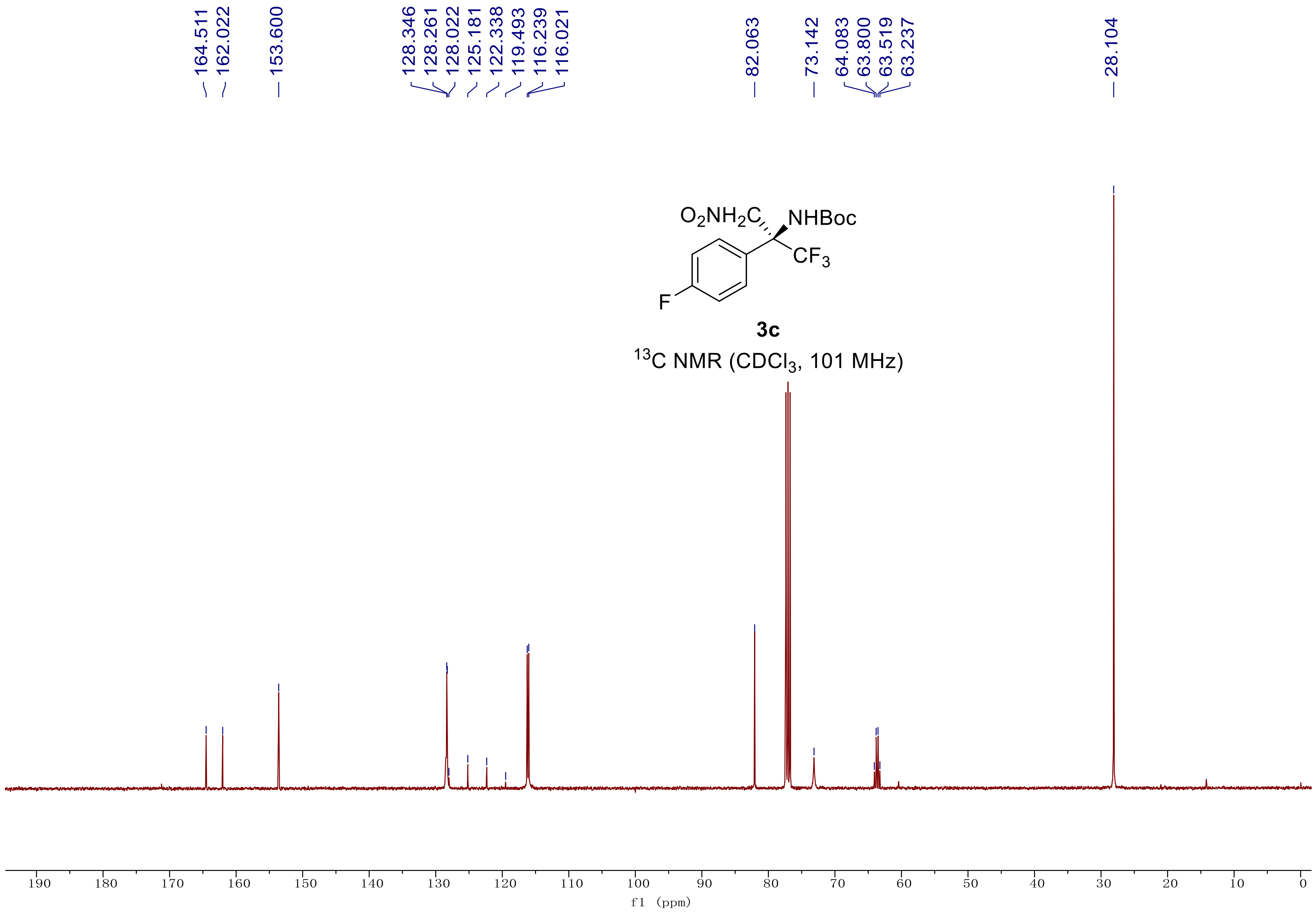
164.511
162.022
153.600
128.346
128.261
128.022
125.181
122.338
119.493
116.239
116.021
82.063
73.142
64.083
63.800
63.519
63.237
28.104
O2NH2C
NHBoc
CF3
F
3c
13C NMR (CDCl3, 101 MHz)
190 180 170 160 150 140 130 120 110 100 90 80 70 60 50 40 30 20 10 0
f1 (ppm)

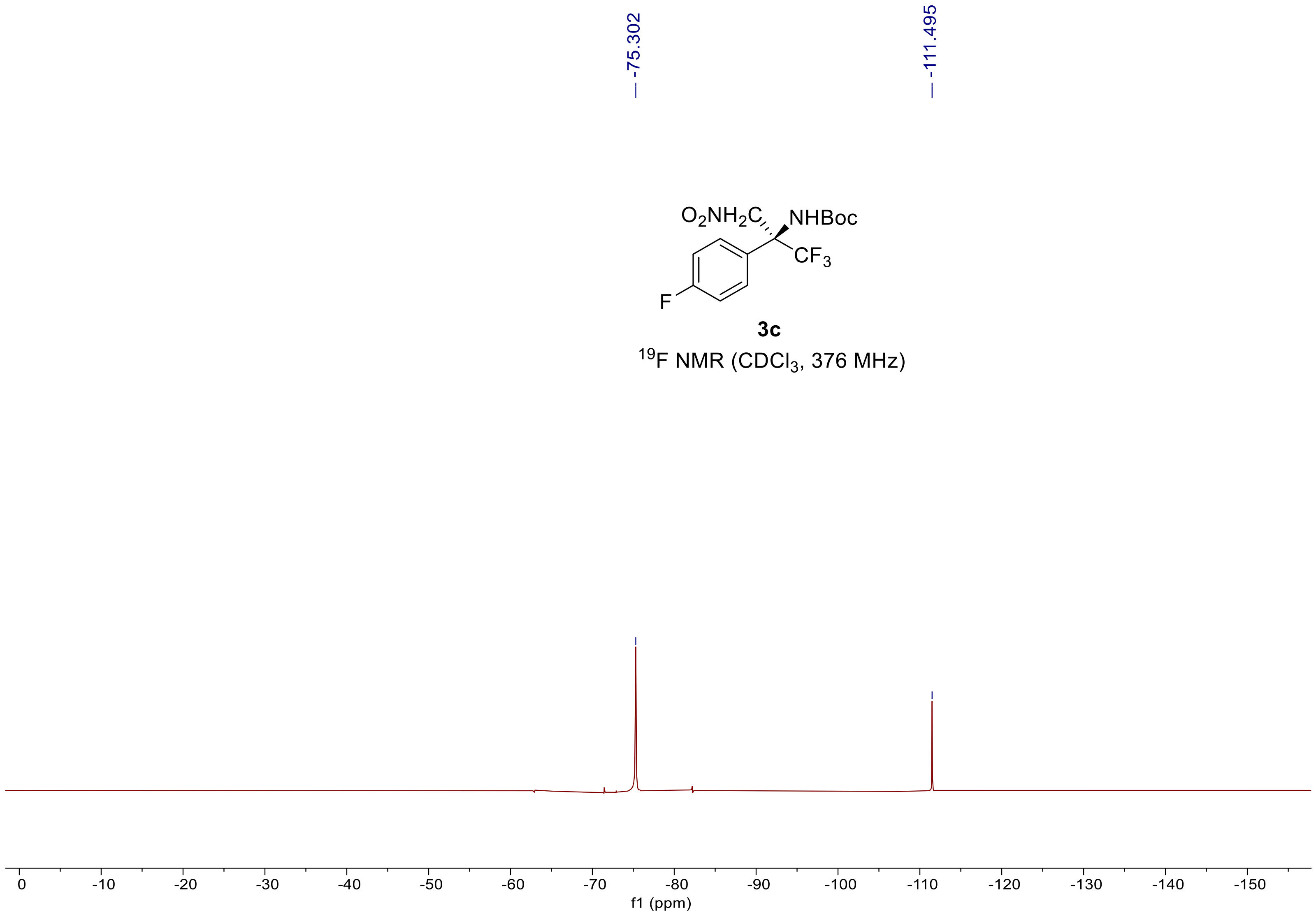

3c

^{19}F NMR ($CDCl_3$, 376 MHz)

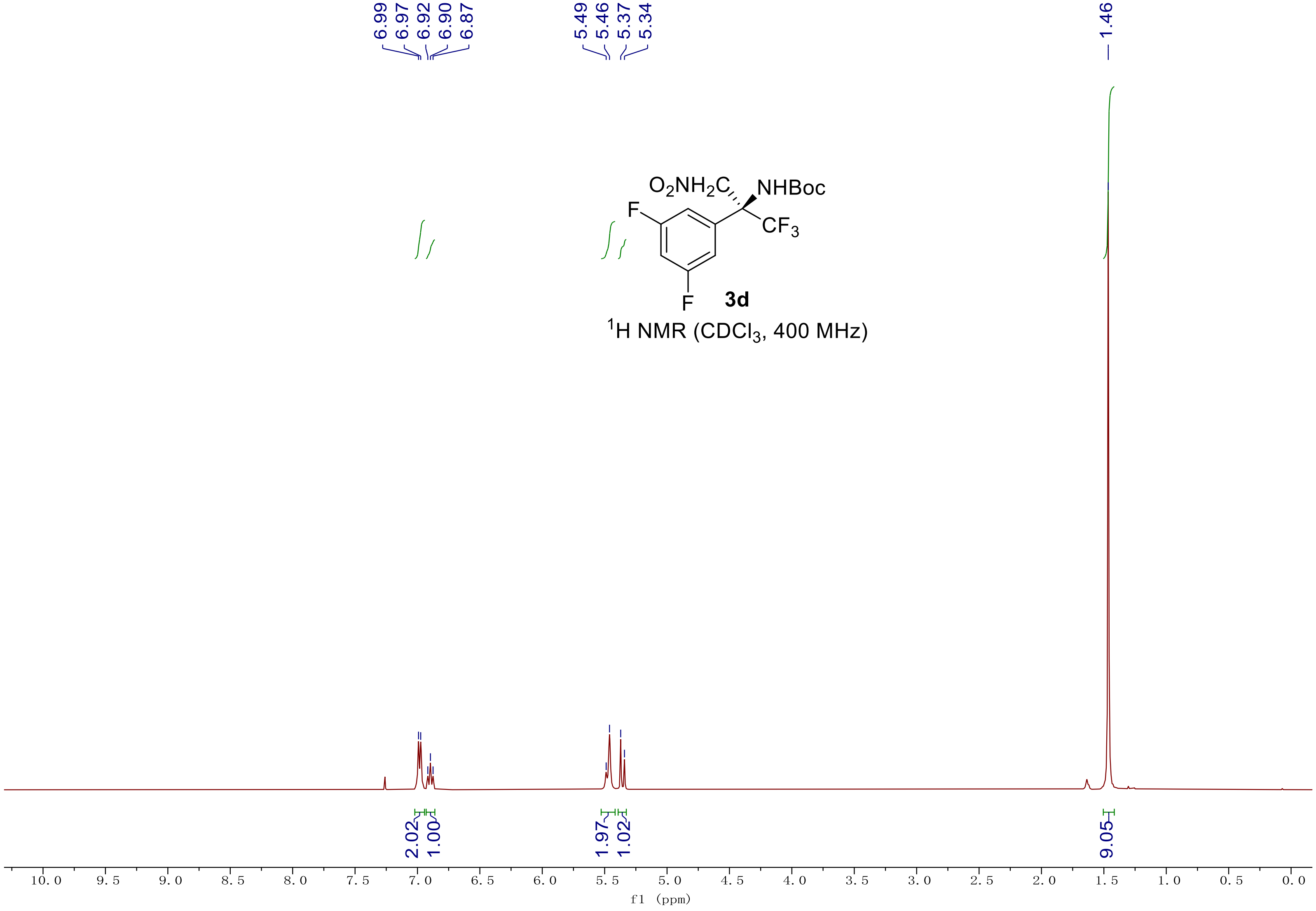
6.99
6.97
6.92
6.90
6.87
5.49
5.46
5.37
5.34
1.46
O_2NH_2C
NHBoc
F
CF_3
F
3d
^1H NMR ($CDCl_3$, 400 MHz)
2.02
1.00
1.97
1.02
9.05
10.0 9.5 9.0 8.5 8.0 7.5 7.0 6.5 6.0 5.5 5.0 4.5 4.0 3.5 3.0 2.5 2.0 1.5 1.0 0.5 0.0
f1 (ppm)

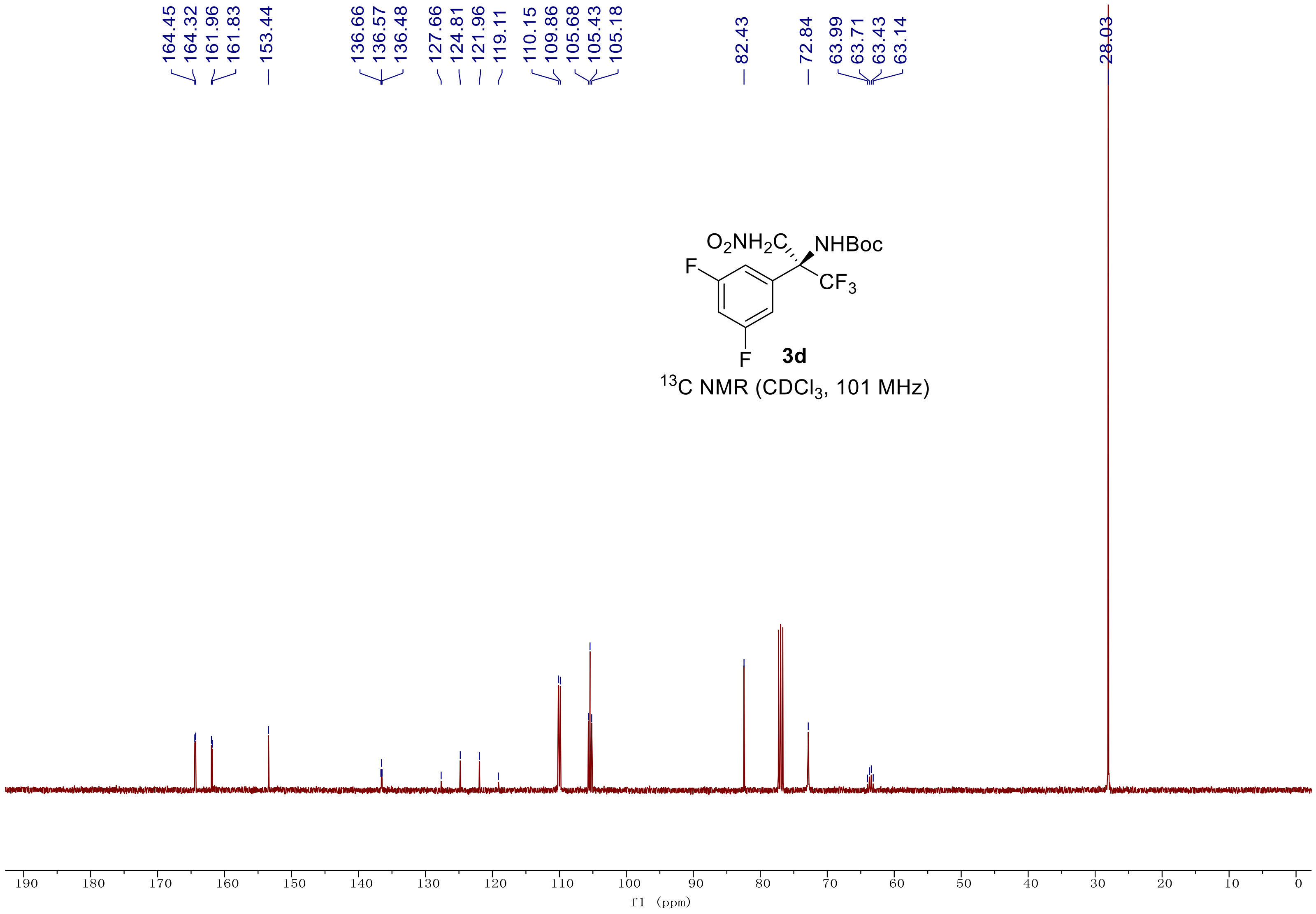
164.45
164.32
161.96
161.83
153.44
136.66
136.57
136.48
127.66
124.81
121.96
119.11
110.15
109.86
105.68
105.43
105.18
82.43
72.84
63.99
63.71
63.43
63.14
28.03
O_2NH_2C
NHBoc
F
CF_3
F
3d
^{13}C NMR ($CDCl_3$, 101 MHz)
190 180 170 160 150 140 130 120 110 100 90 80 70 60 50 40 30 20 10 0
f1 (ppm)

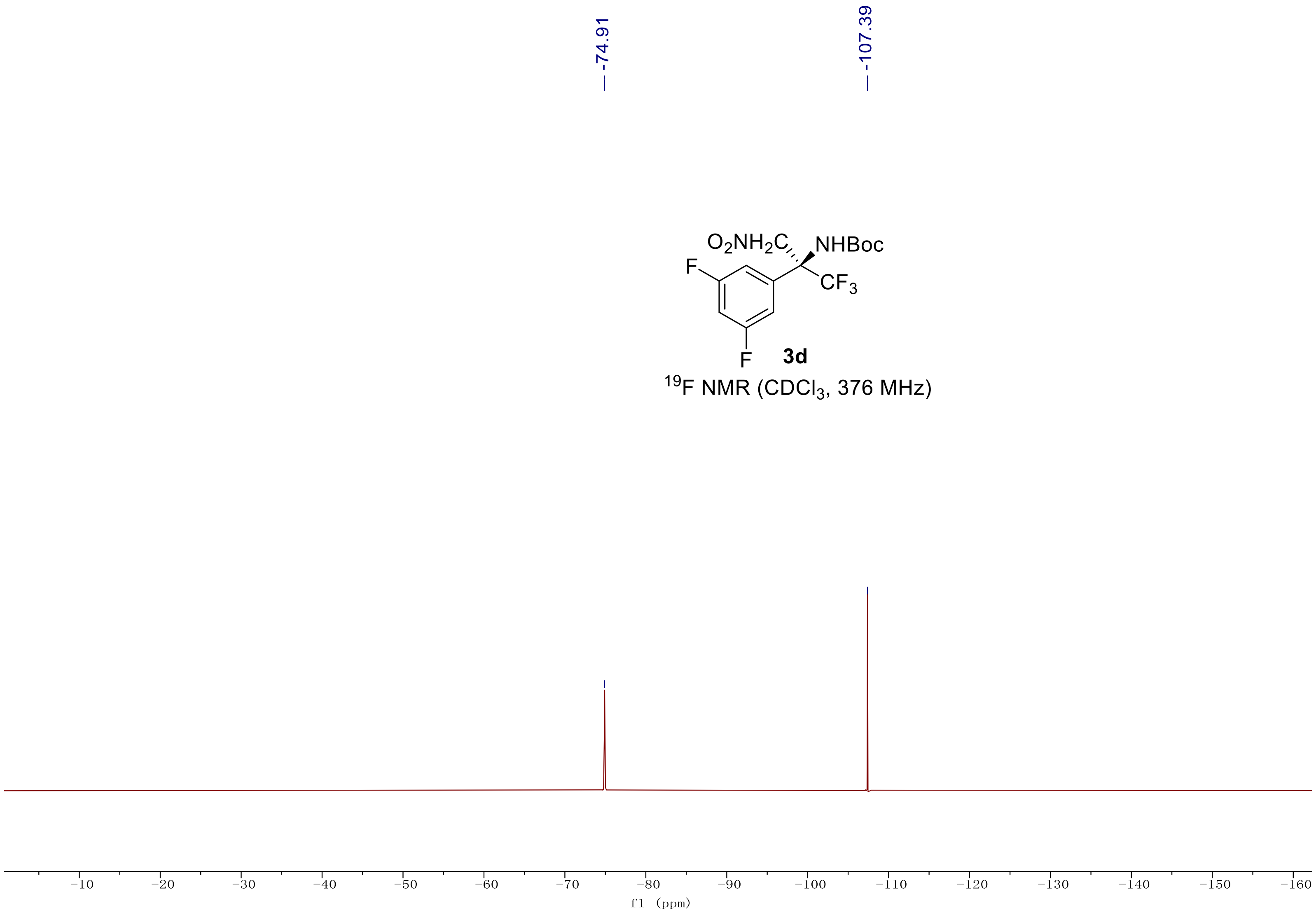

3d

^{19}F NMR ($CDCl_3$, 376 MHz)

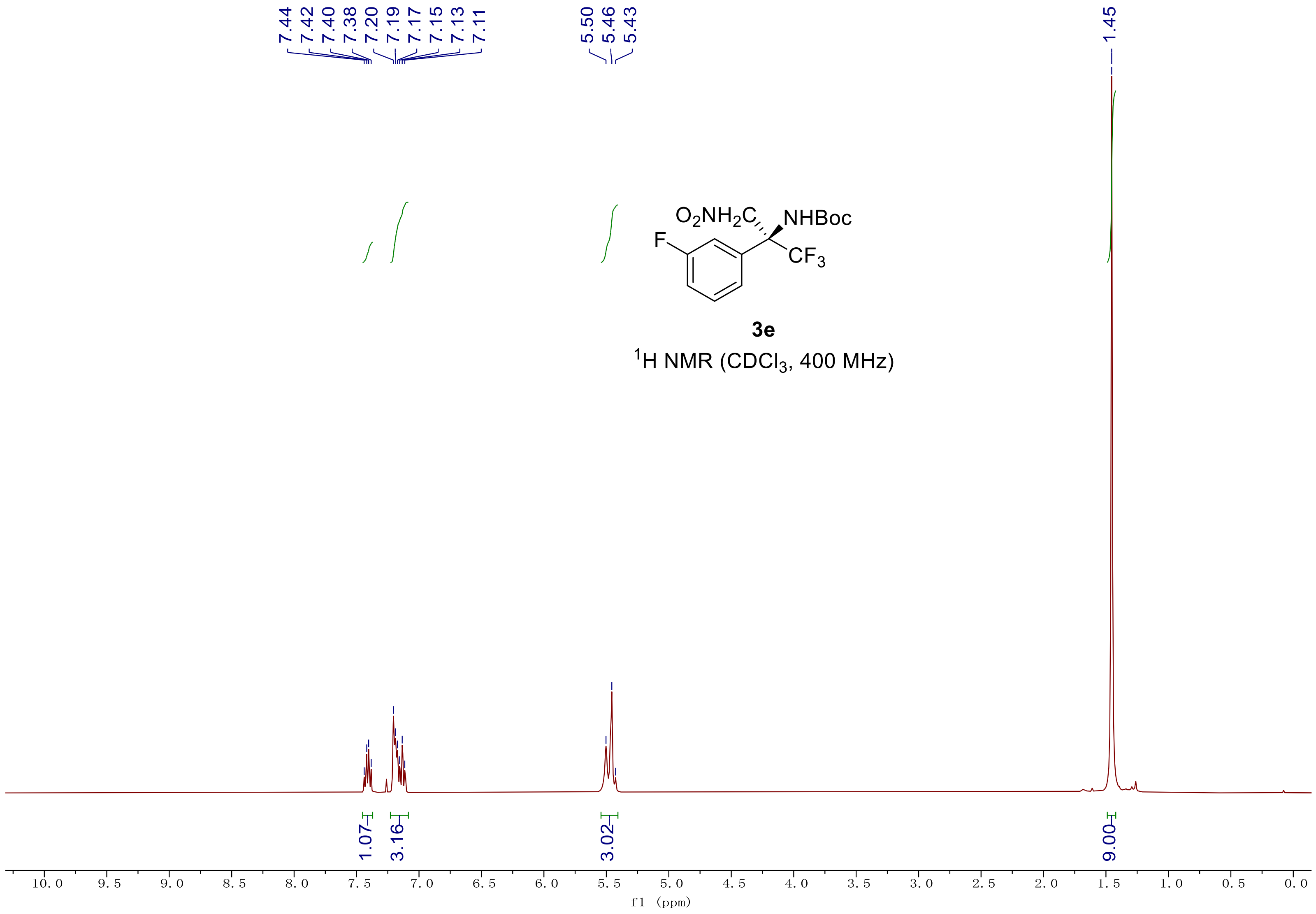
7.44
7.42
7.40
7.38
7.20
7.19
7.17
7.15
7.13
7.11
5.50
5.46
5.43
1.45
O_2NH_2C
NHBoc
F
CF_3
3e
^1H NMR ($CDCl_3$, 400 MHz)
1.07
3.16
3.02
9.00
10.0
9.5
9.0
8.5
8.0
7.5
7.0
6.5
6.0
5.5
5.0
4.5
4.0
3.5
3.0
2.5
2.0
1.5
1.0
0.5
0.0
f1 (ppm)

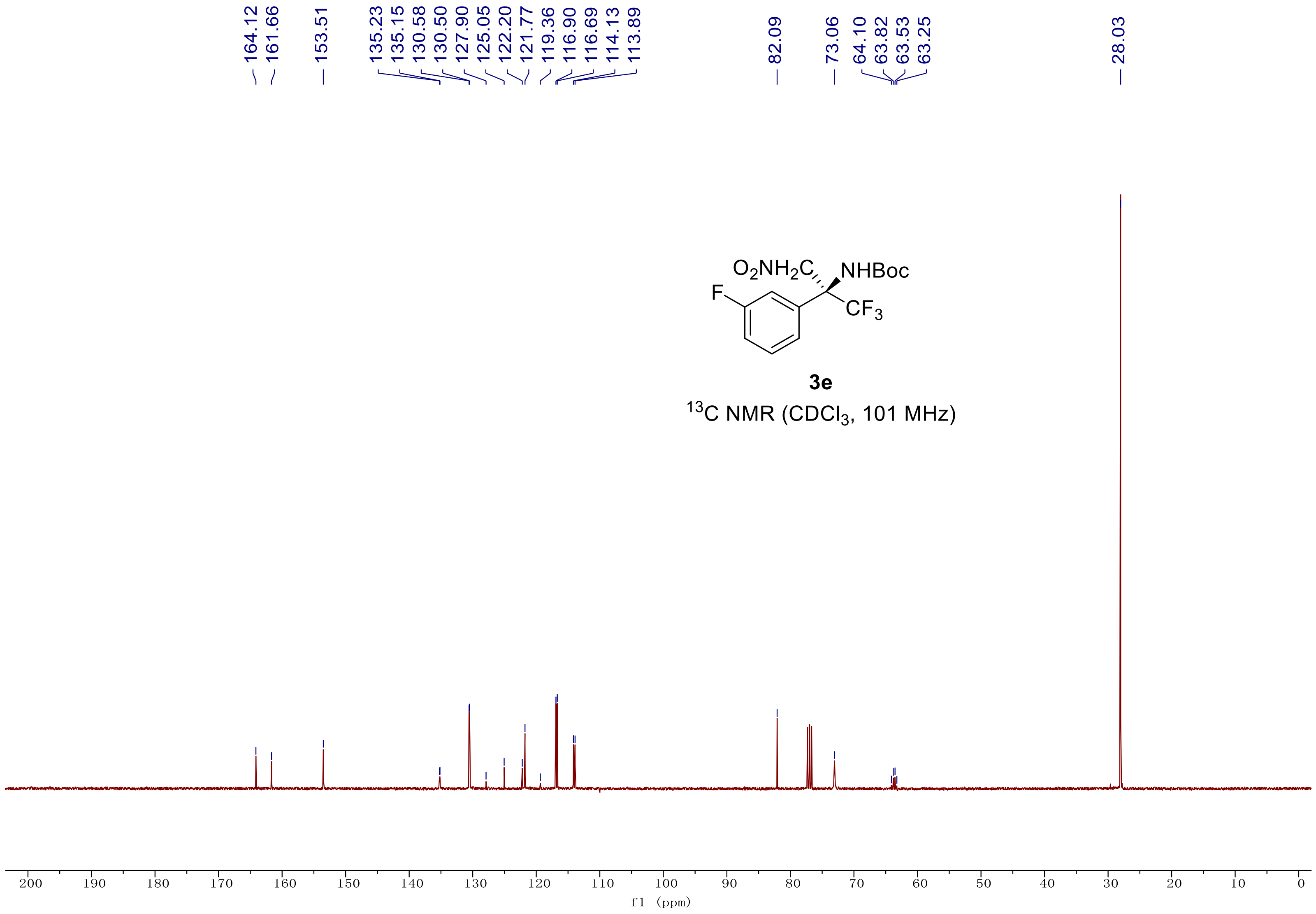
164.12
161.66
153.51
135.23
135.15
130.58
130.50
127.90
125.05
122.20
121.77
119.36
116.90
116.69
114.13
113.89
82.09
73.06
64.10
63.82
63.53
63.25
28.03
O2NH2C
NHBoc
F
CF3
3e
13C NMR (CDCl3, 101 MHz)
200 190 180 170 160 150 140 130 120 110 100 90 80 70 60 50 40 30 20 10 0
f1 (ppm)

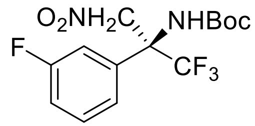

3e

^{19}F NMR ($CDCl_3$, 376 MHz)

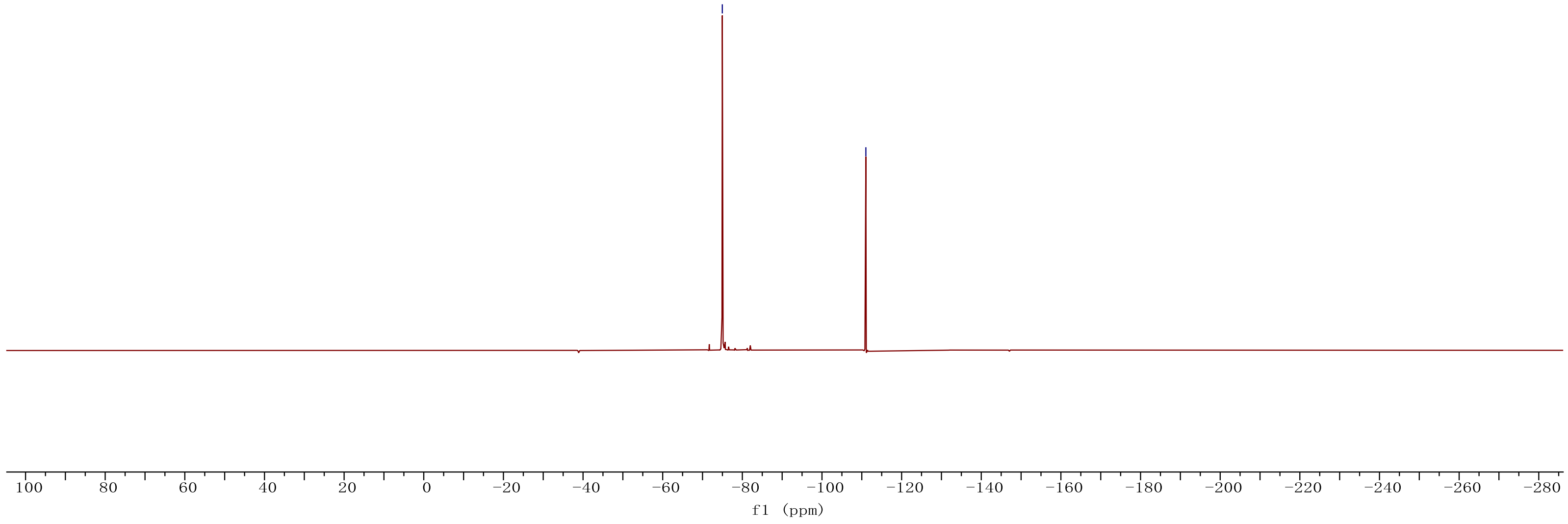

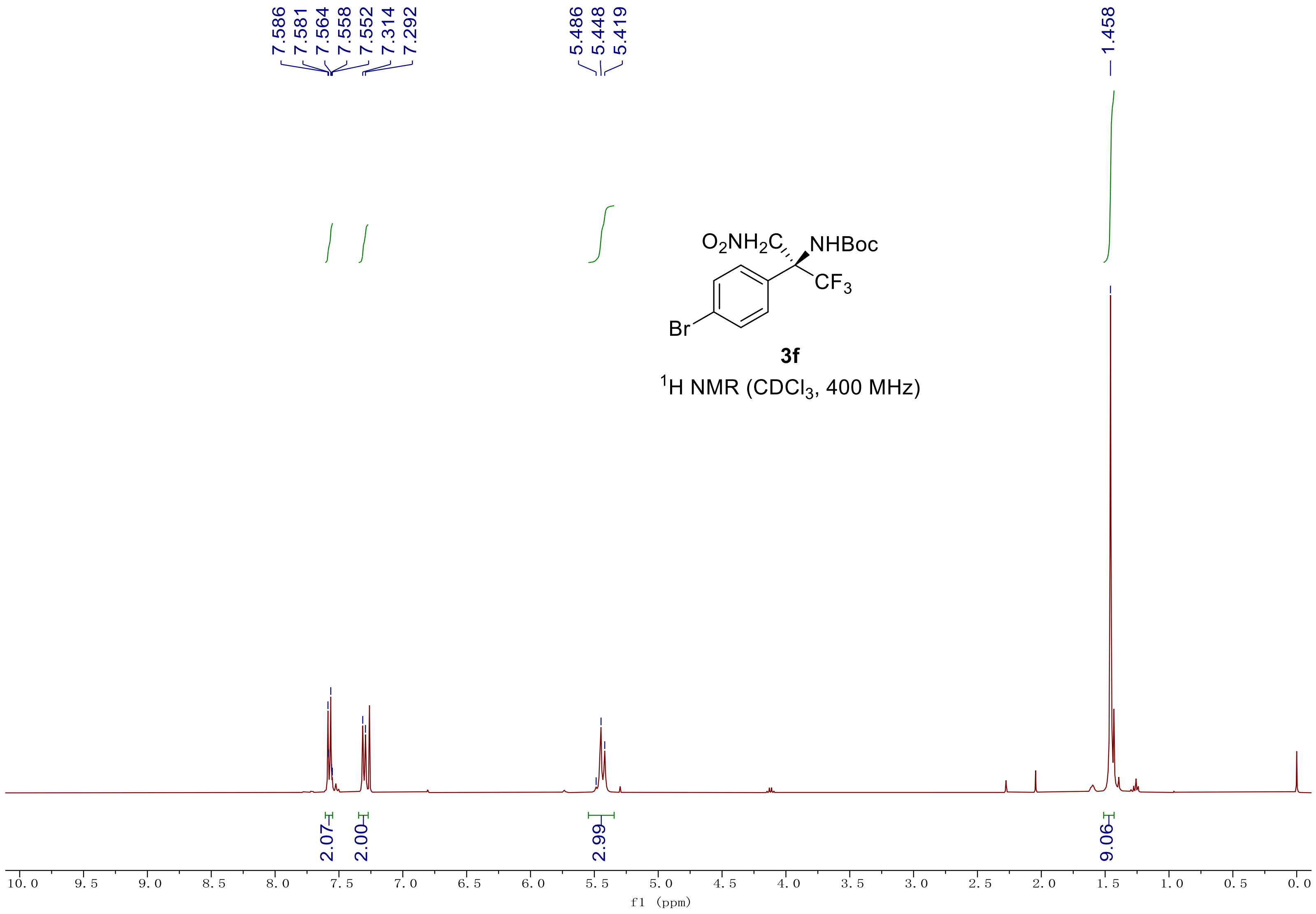

7.586
7.581
7.564
7.558
7.552
7.314
7.292
5.486
5.448
5.419
1.458
O2NH2C
NHBoc
CF3
Br
3f
1H NMR (CDCl3, 400 MHz)
2.07
2.00
2.99
9.06
10.0 9.5 9.0 8.5 8.0 7.5 7.0 6.5 6.0 5.5 5.0 4.5 4.0 3.5 3.0 2.5 2.0 1.5 1.0 0.5 0.0
f1 (ppm)

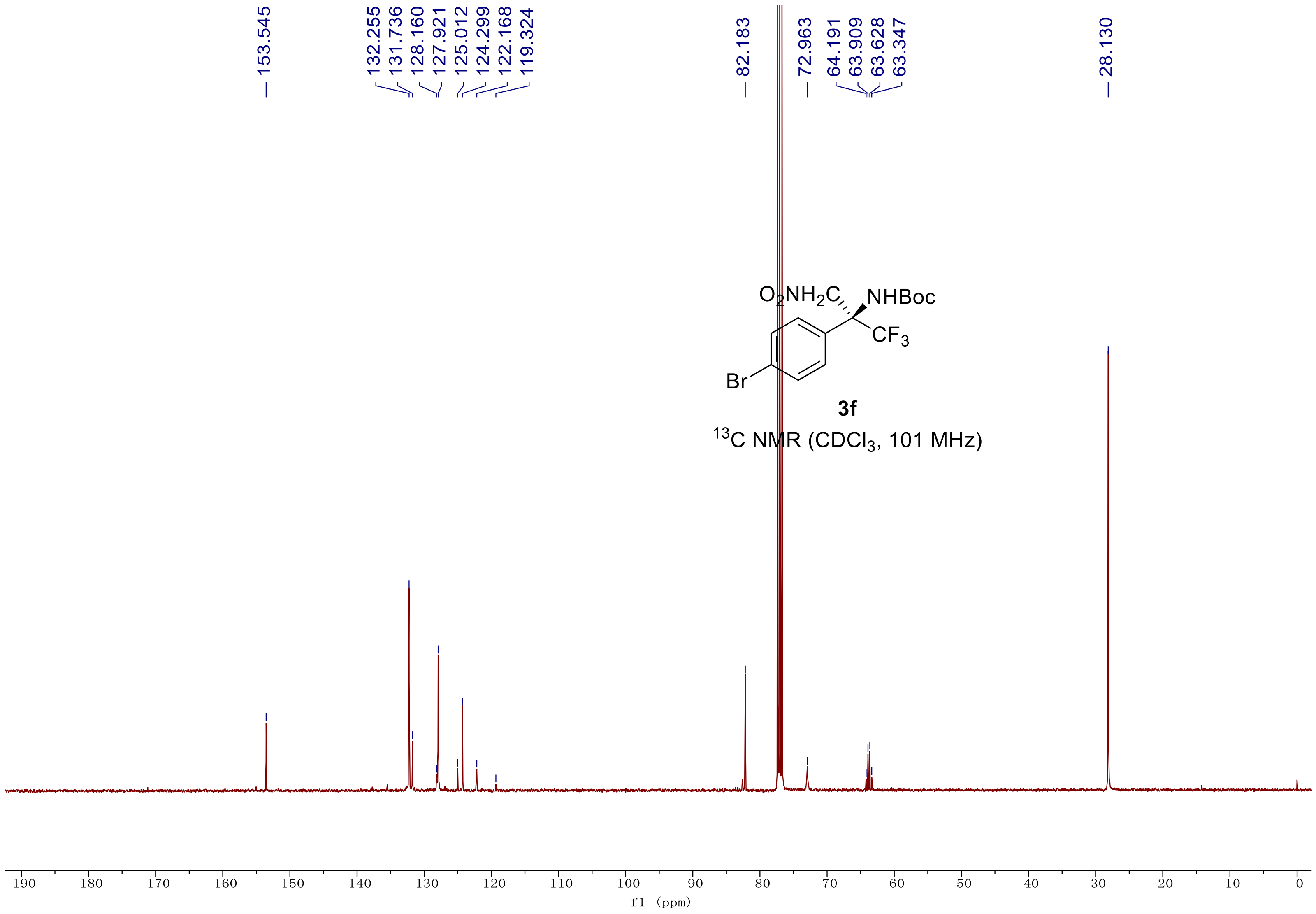
153.545
132.255
131.736
128.160
127.921
125.012
124.299
122.168
119.324
82.183
72.963
64.191
63.909
63.628
63.347
28.130
O2NH2C
NHBoc
CF3
Br
3f
13C NMR (CDCl3, 101 MHz)
190 180 170 160 150 140 130 120 110 100 90 80 70 60 50 40 30 20 10 0
f1 (ppm)

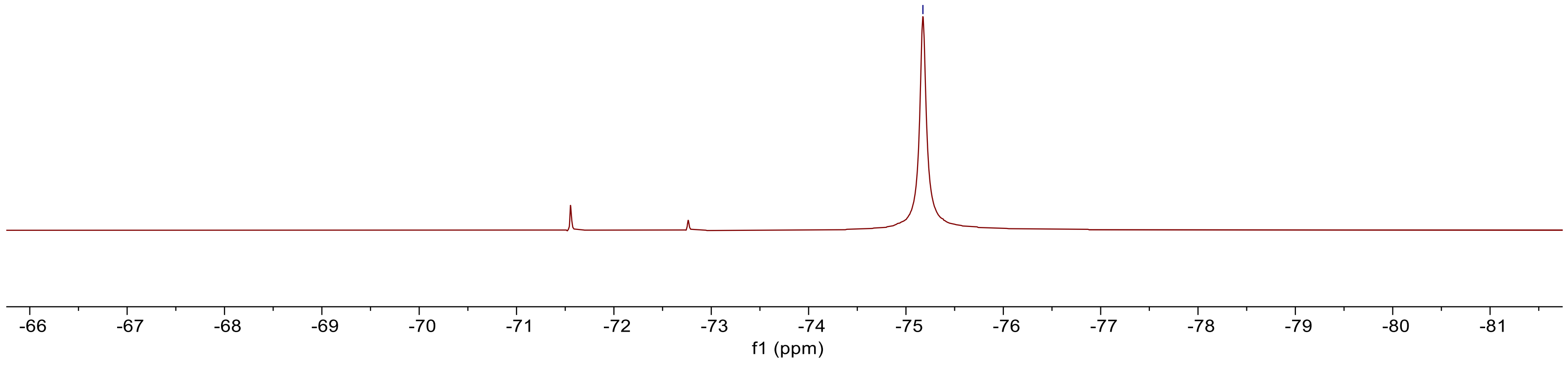

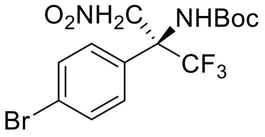

3f

^{19}F NMR ($CDCl_3$, 376 MHz)

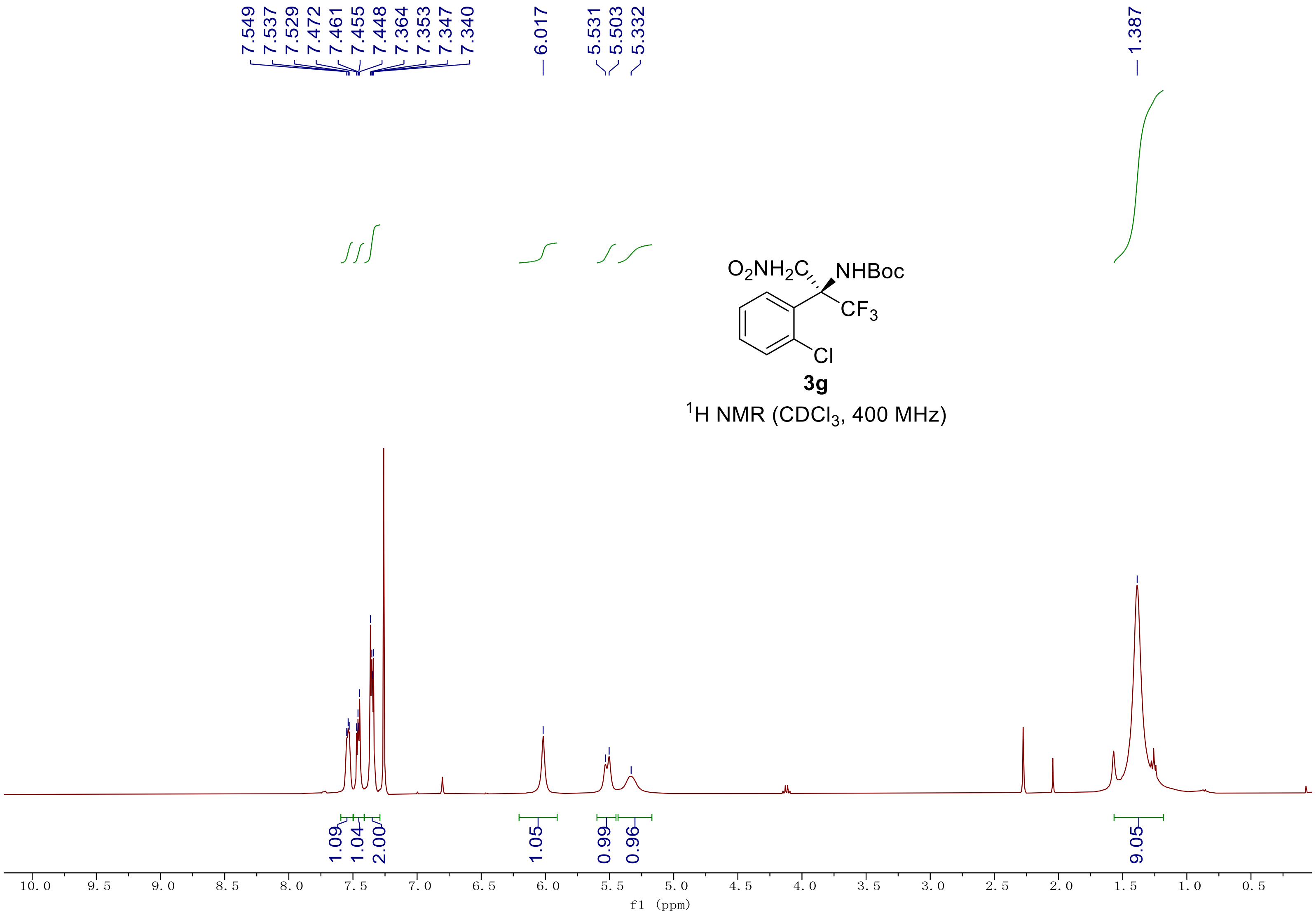

3g

^{1}H NMR ($CDCl_3$, 400 MHz)

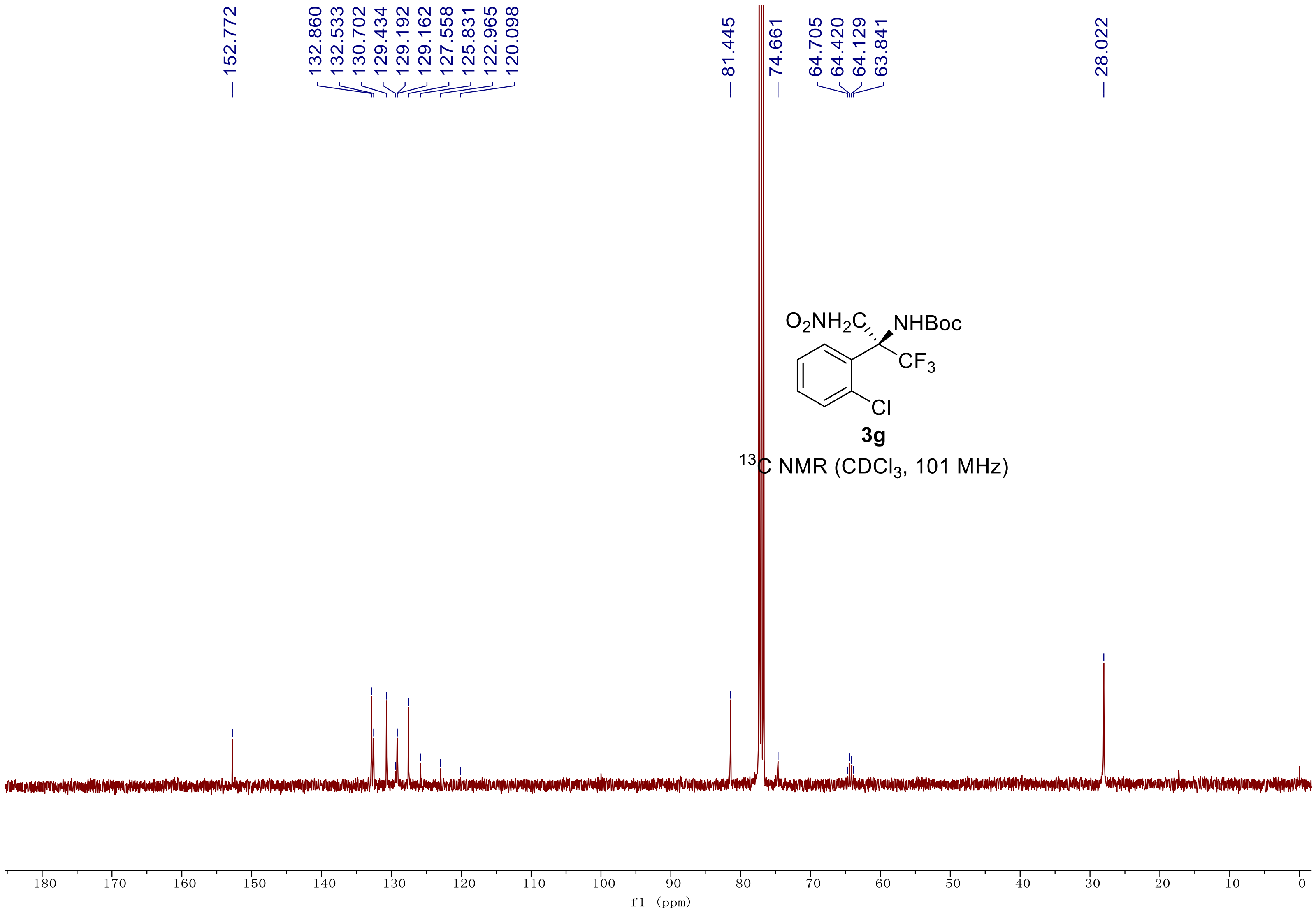
152.772
132.860
132.533
130.702
129.434
129.192
129.162
127.558
125.831
122.965
120.098
81.445
74.661
64.705
64.420
64.129
63.841
28.022
O2NH2C
NHBoc
CF3
Cl
3g
13C NMR (CDCl3, 101 MHz)
180 170 160 150 140 130 120 110 100 90 80 70 60 50 40 30 20 10 0
f1 (ppm)

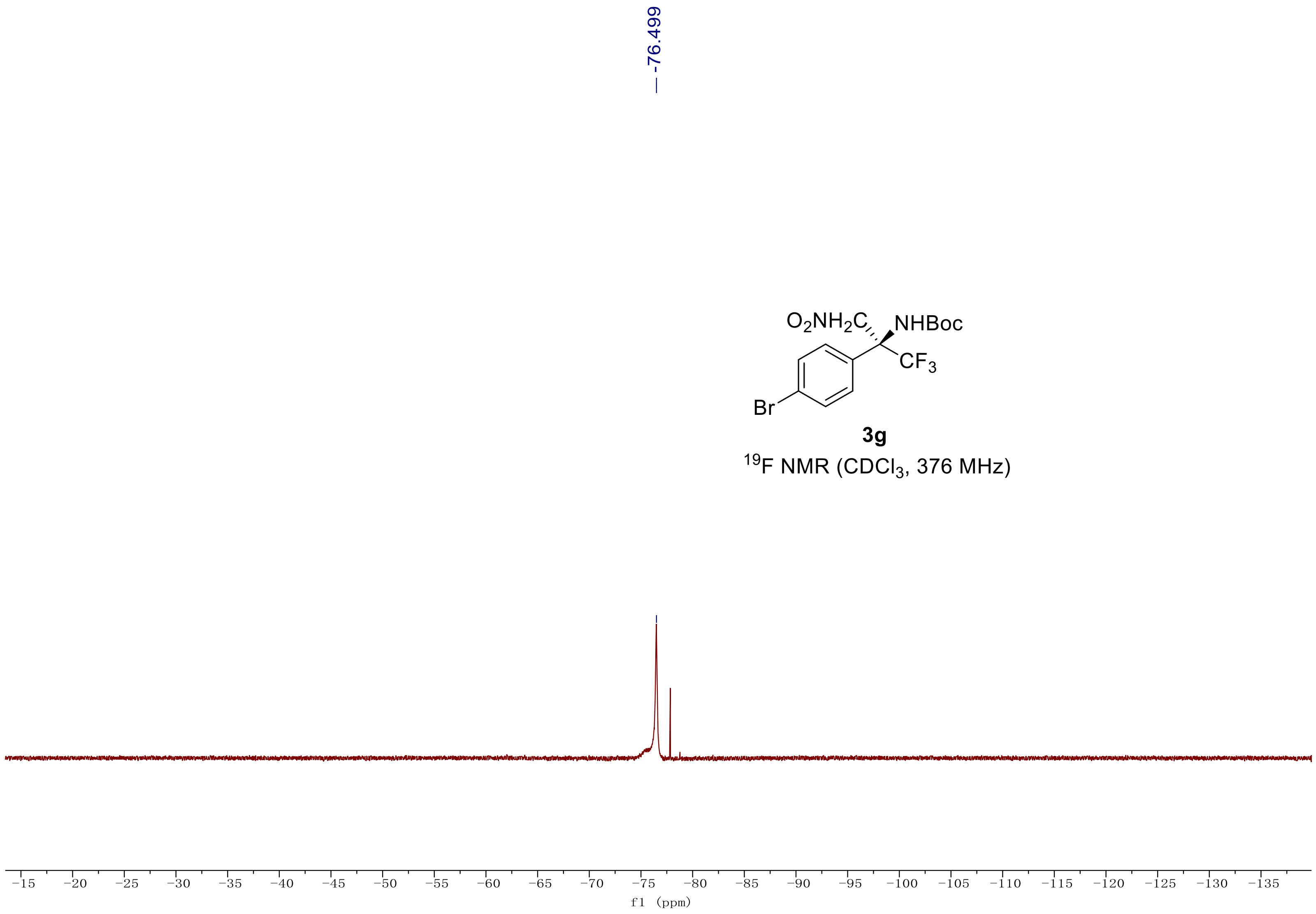

3g

^{19}F NMR ($CDCl_3$, 376 MHz)

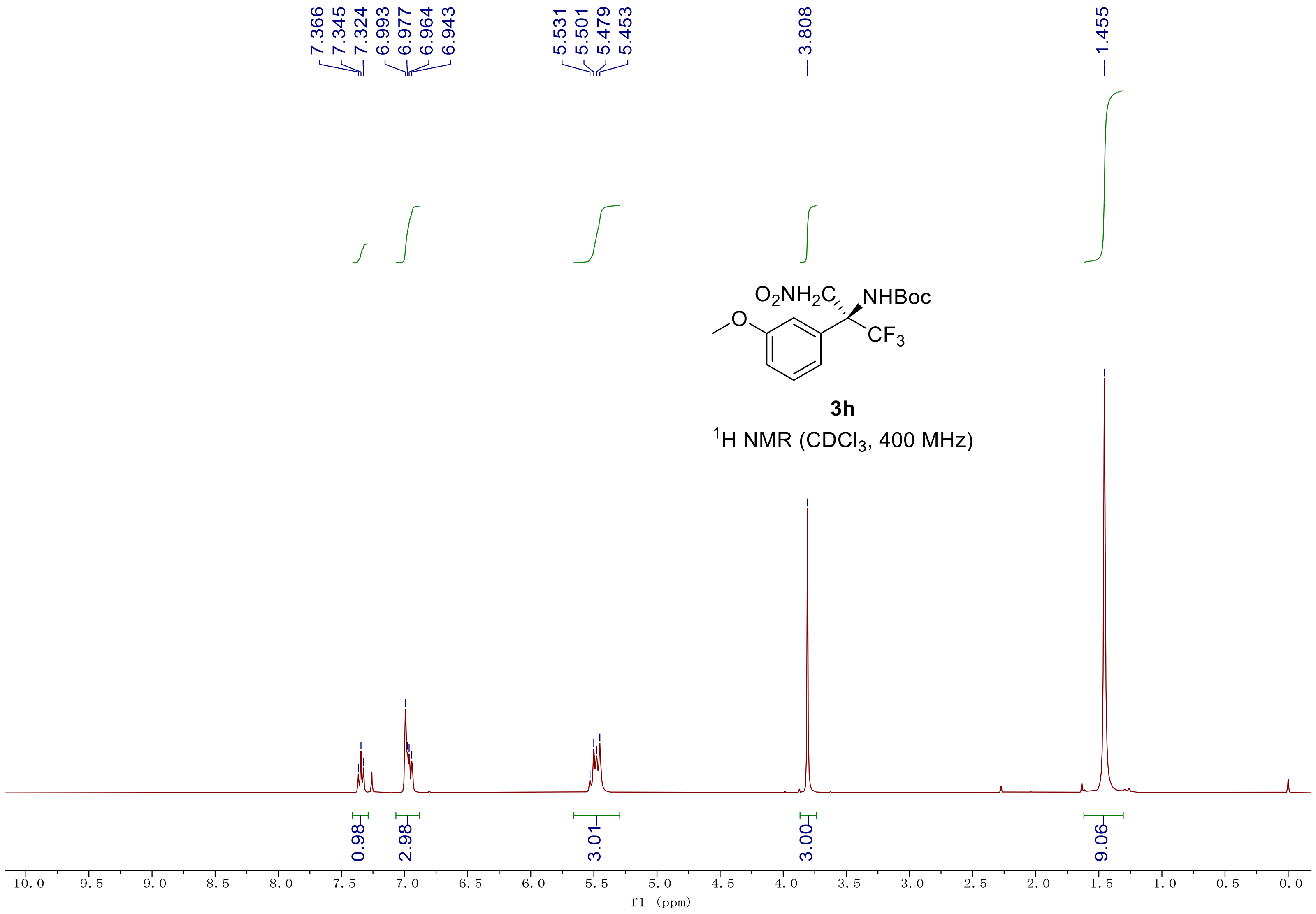

7.366
7.345
7.324
6.993
6.977
6.964
6.943
5.531
5.501
5.479
5.453
3.808
1.455
O2NH2C
NHBoc
CF3
O
3h
1H NMR (CDCl3, 400 MHz)
0.98
2.98
3.01
3.00
9.06
10.0
9.5
9.0
8.5
8.0
7.5
7.0
6.5
6.0
5.5
5.0
4.5
4.0
3.5
3.0
2.5
2.0
1.5
1.0
0.5
0.0
f1 (ppm)

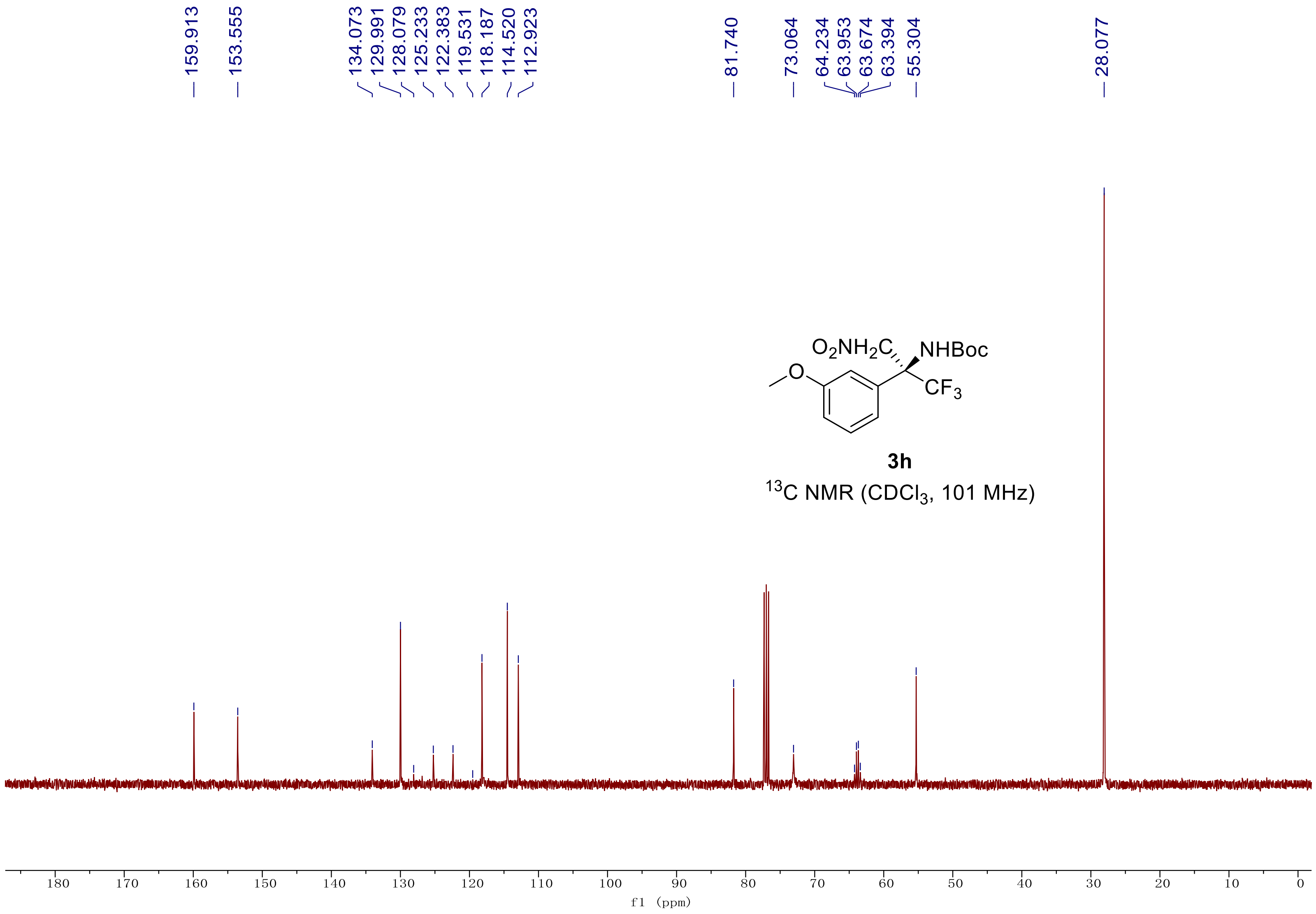
159.913
153.555
134.073
129.991
128.079
125.233
122.383
119.531
118.187
114.520
112.923
81.740
73.064
64.234
63.953
63.674
63.394
55.304
28.077
O2NH2C
NHBoc
O
CF3
3h
13C NMR (CDCl3, 101 MHz)
180 170 160 150 140 130 120 110 100 90 80 70 60 50 40 30 20 10 0
f1 (ppm)

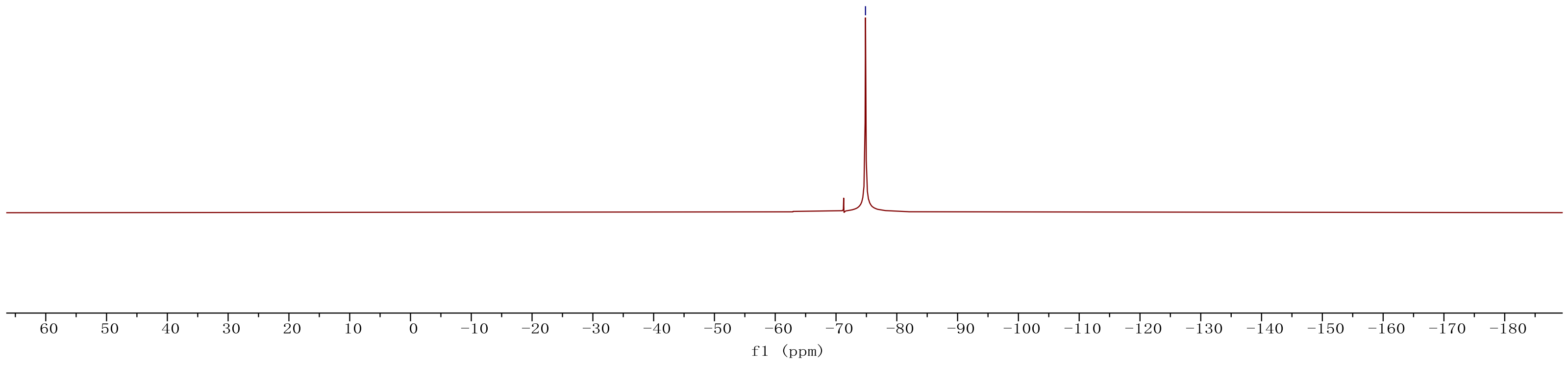

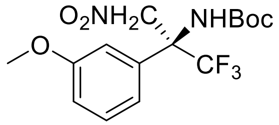

3h

^{19}F NMR ($CDCl_3$, 376 MHz)

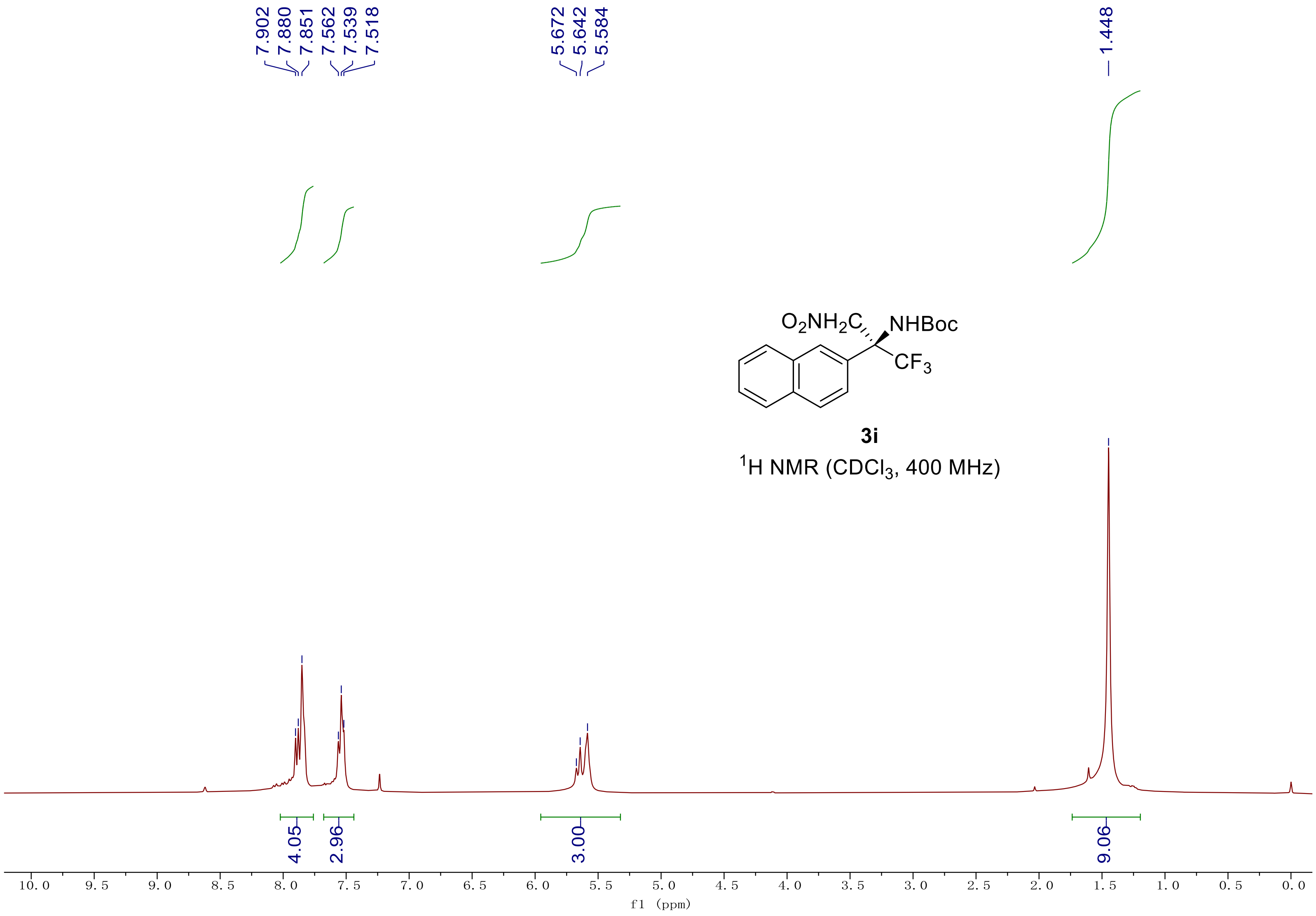

7.902
7.880
7.851
7.562
7.539
7.518
5.672
5.642
5.584
1.448
O_2NH_2C
NHBoc
CF_3
3i
^{1}H NMR ($CDCl_3$, 400 MHz)
4.05
2.96
3.00
9.06
10.0 9.5 9.0 8.5 8.0 7.5 7.0 6.5 6.0 5.5 5.0 4.5 4.0 3.5 3.0 2.5 2.0 1.5 1.0 0.5 0.0
f1 (ppm)

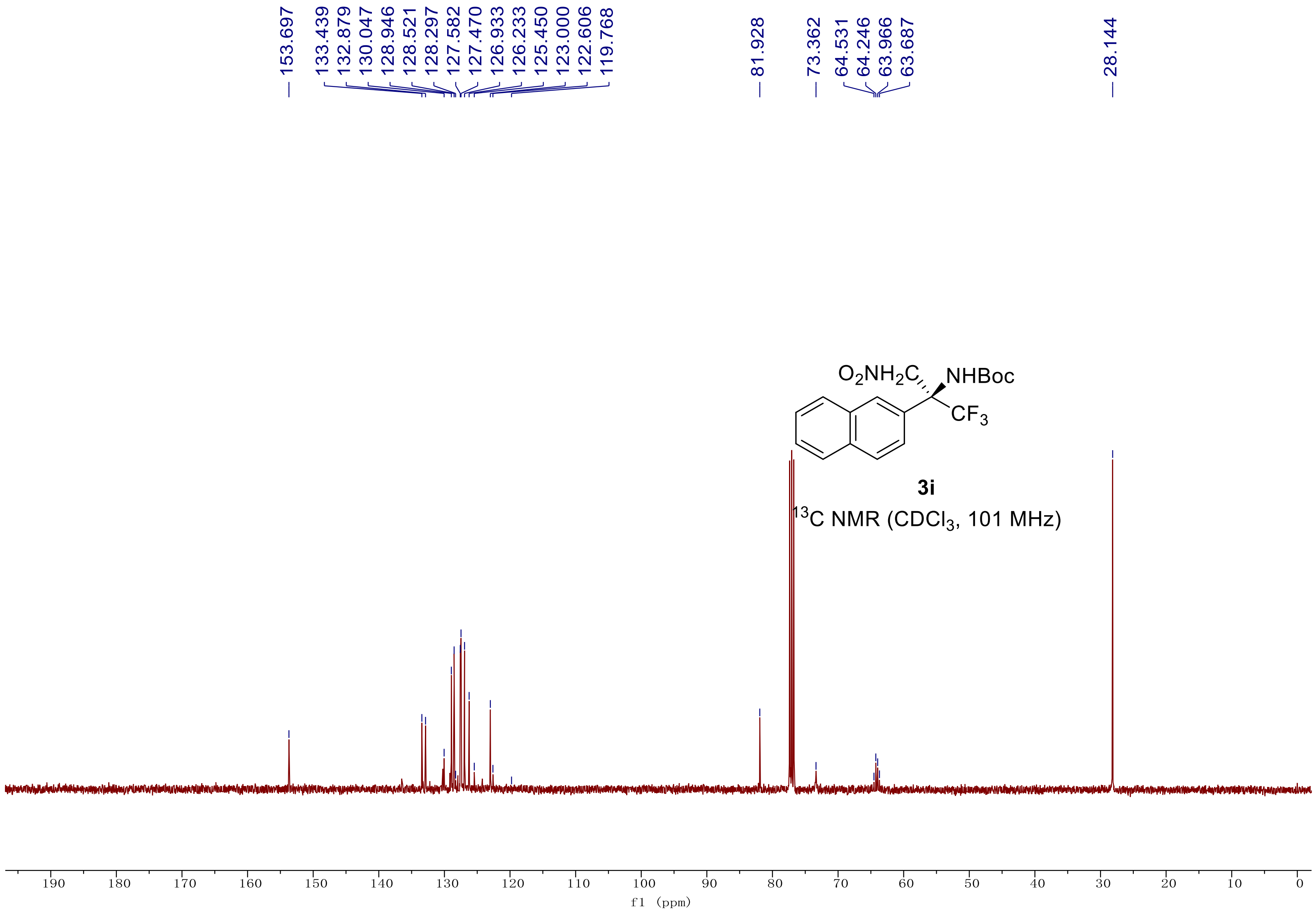
153.697
133.439
132.879
130.047
128.946
128.521
128.297
127.582
127.470
126.933
126.233
125.450
123.000
122.606
119.768
81.928
73.362
64.531
64.246
63.966
63.687
28.144
O_2NH_2C
NHBoc
CF_3
3i
^{13}C NMR ($CDCl_3$, 101 MHz)
190 180 170 160 150 140 130 120 110 100 90 80 70 60 50 40 30 20 10 0
f1 (ppm)

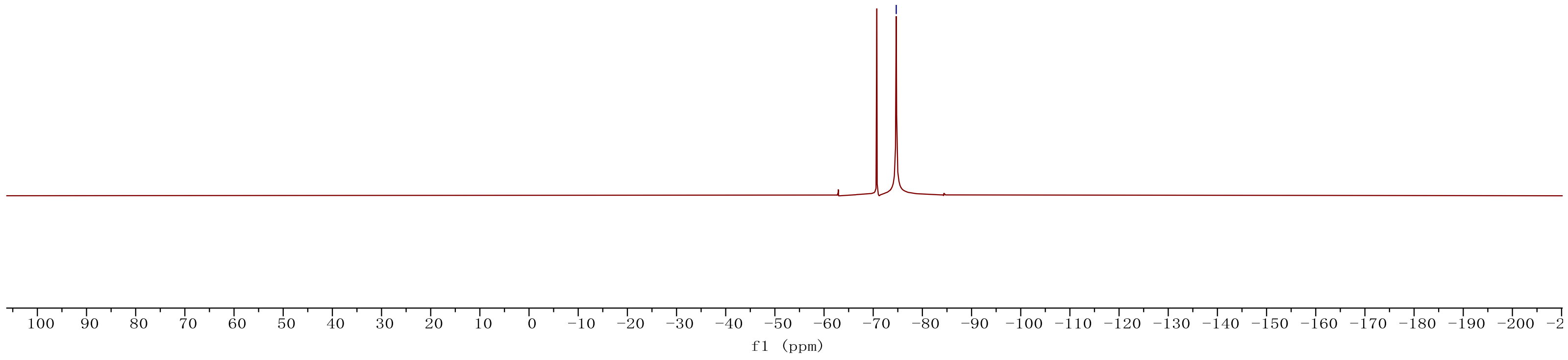

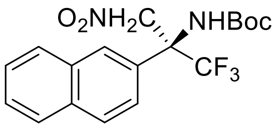

3i

^{19}F NMR ($CDCl_3$, 376 MHz)

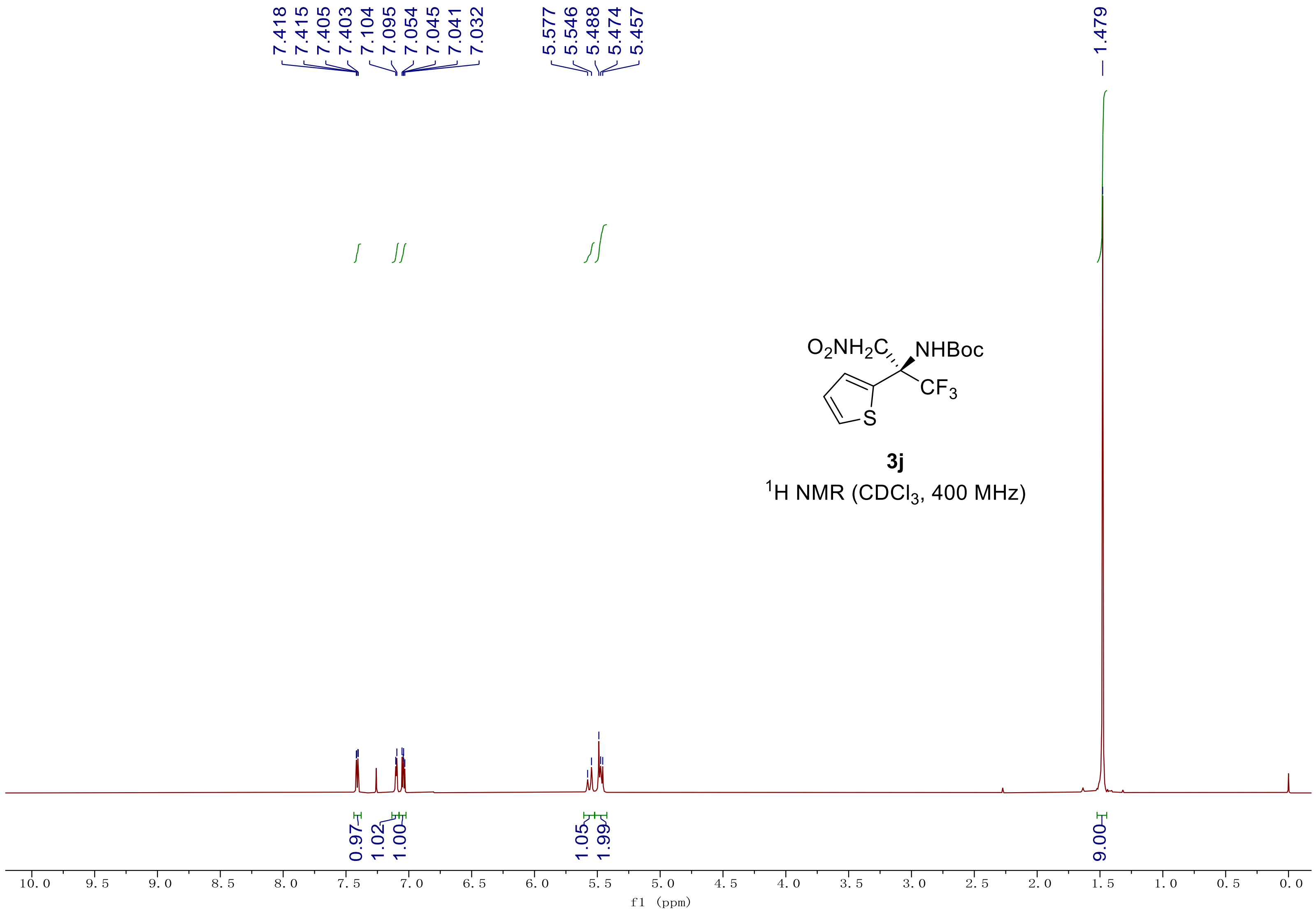

7.418
7.415
7.405
7.403
7.104
7.095
7.054
7.045
7.041
7.032
5.577
5.546
5.488
5.474
5.457
1.479
O2NH2C
NHBoc
CF3
S
3j
1H NMR (CDCl3, 400 MHz)
0.97
1.02
1.00
1.05
1.99
9.00
10.0 9.5 9.0 8.5 8.0 7.5 7.0 6.5 6.0 5.5 5.0 4.5 4.0 3.5 3.0 2.5 2.0 1.5 1.0 0.5 0.0
f1 (ppm)

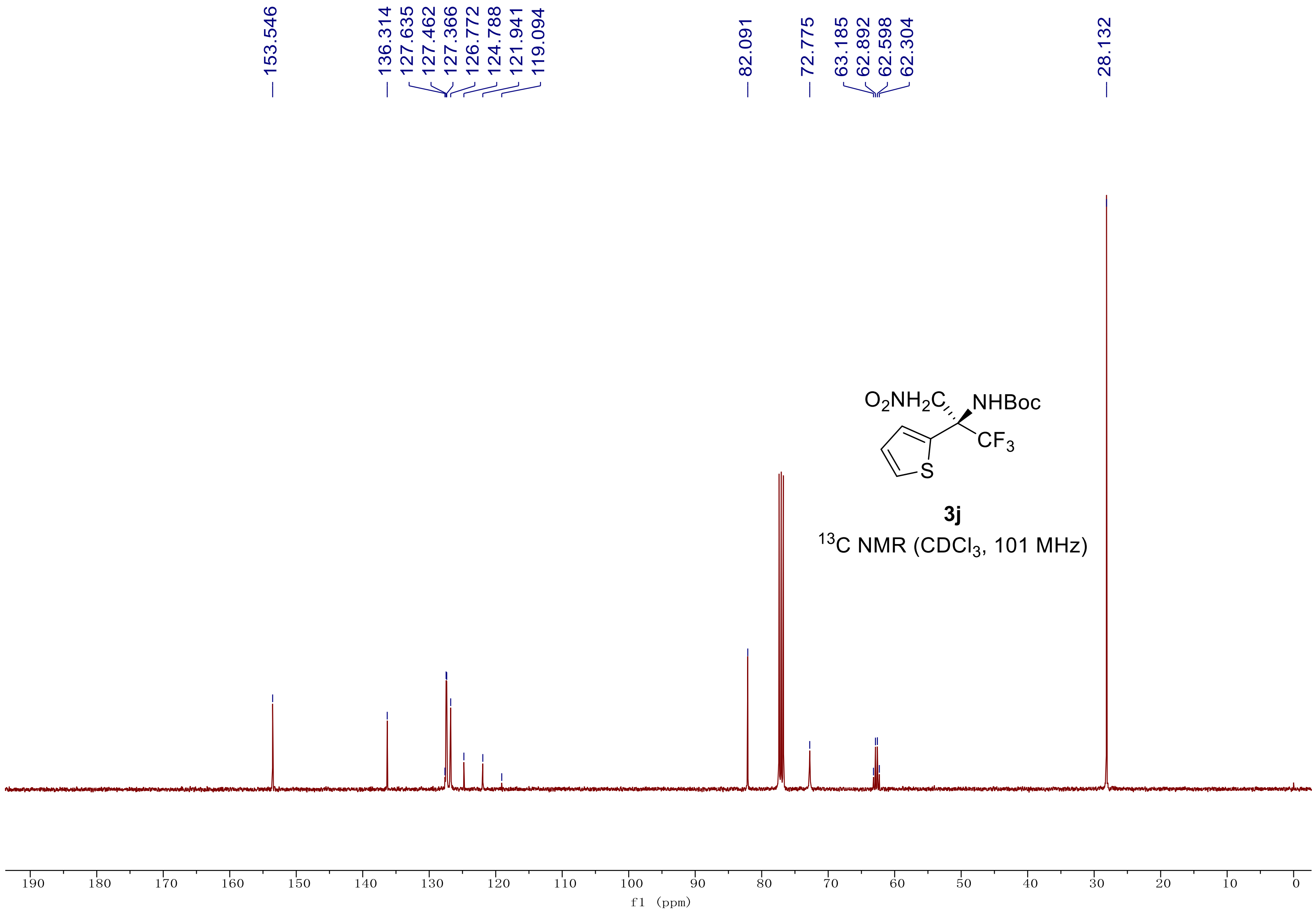
153.546
136.314
127.635
127.462
127.366
126.772
124.788
121.941
119.094
82.091
72.775
63.185
62.892
62.598
62.304
28.132
O2NH2C
NHBoc
CF3
S
3j
13C NMR (CDCl3, 101 MHz)
190 180 170 160 150 140 130 120 110 100 90 80 70 60 50 40 30 20 10 0
f1 (ppm)

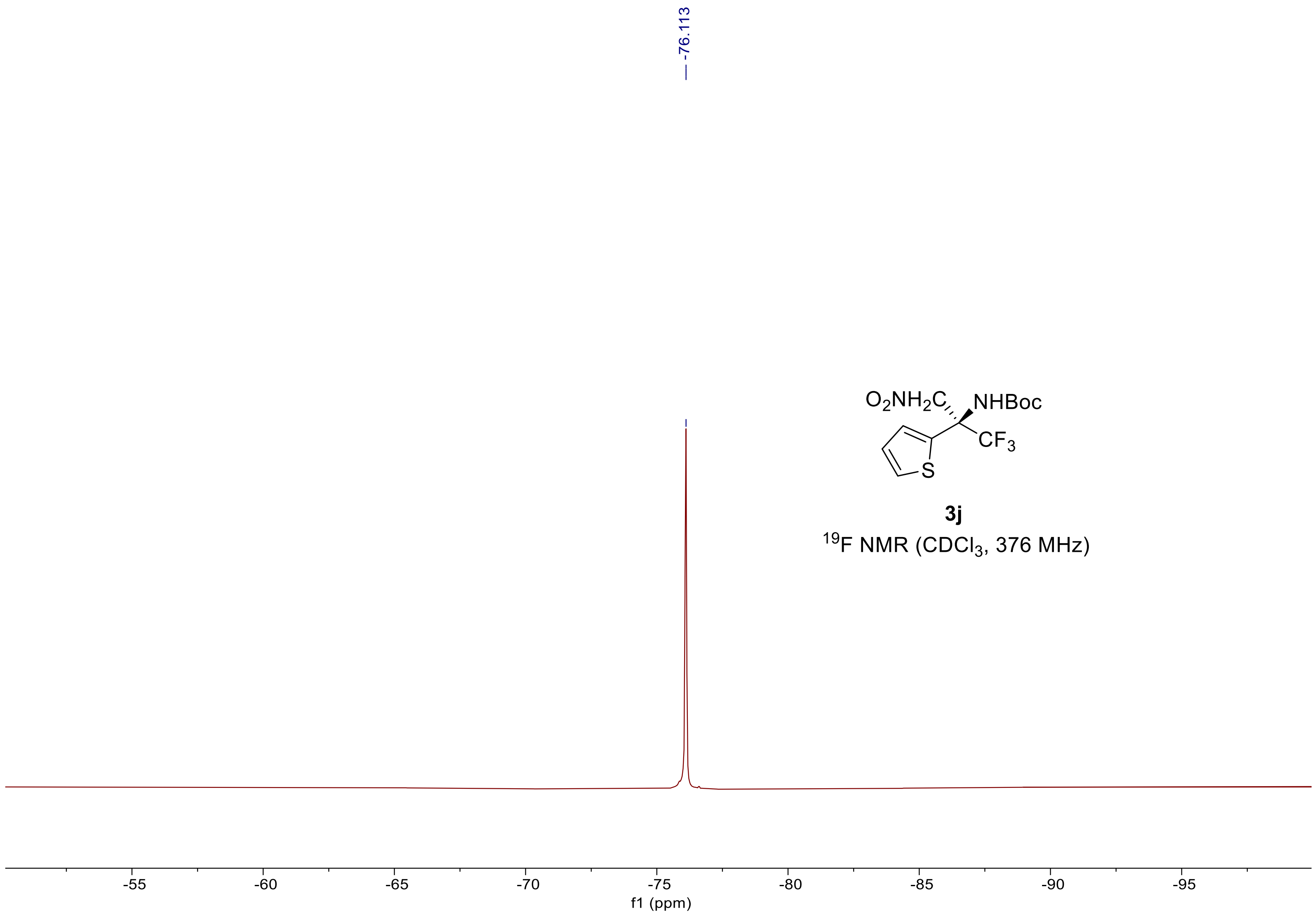

3j

^{19}F NMR ($CDCl_3$, 376 MHz)

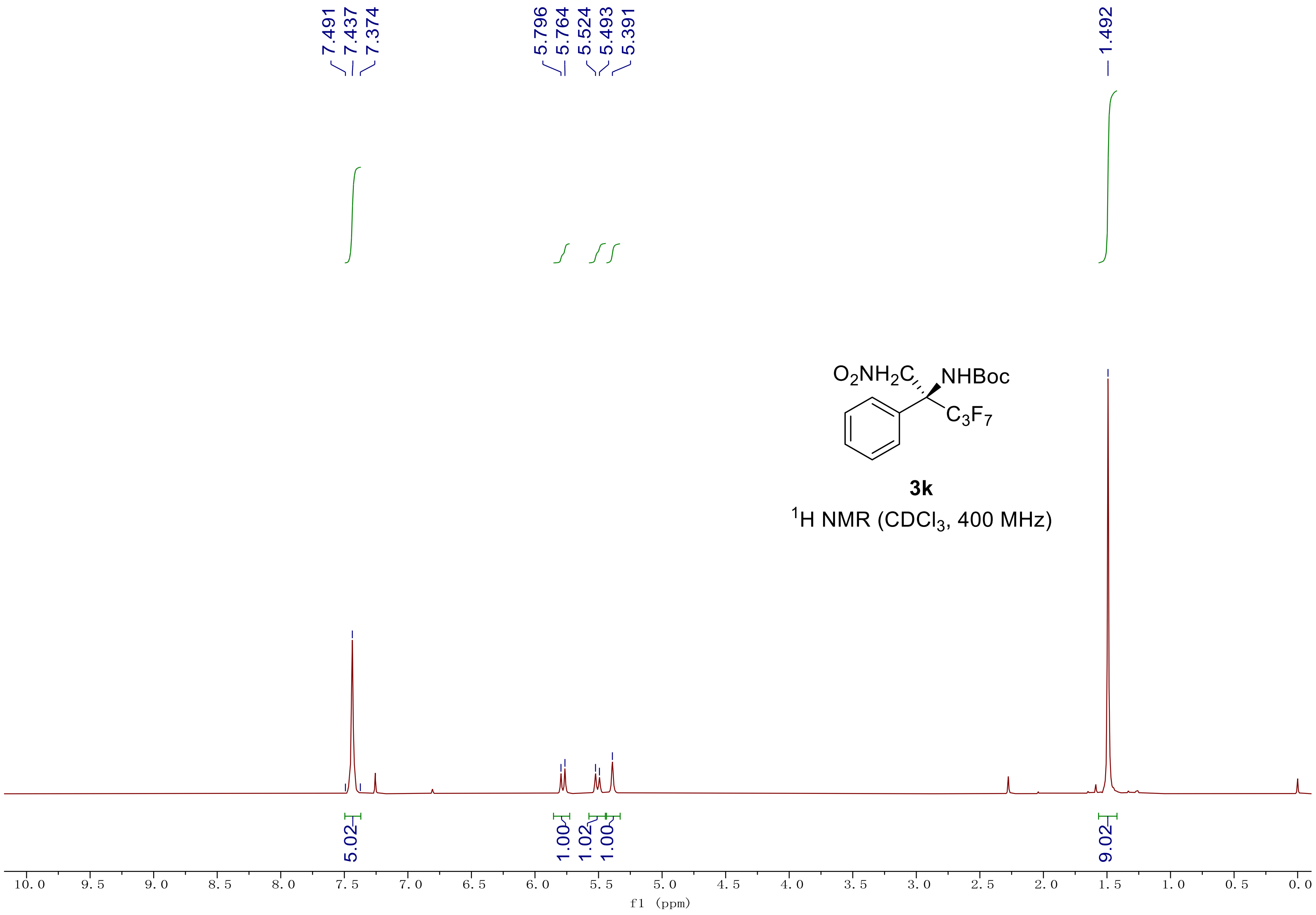

3k

^1H NMR ($CDCl_3$, 400 MHz)

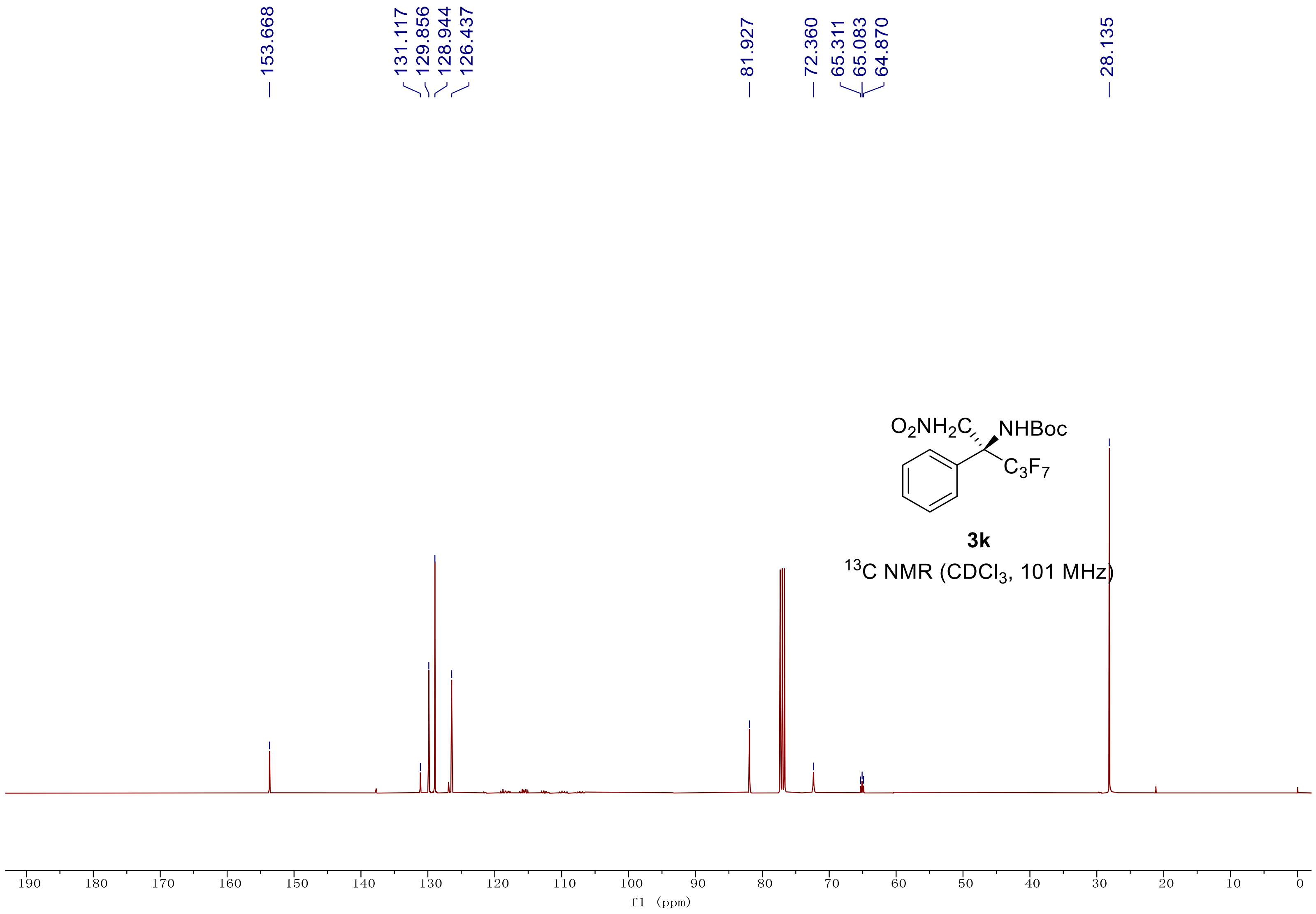

3k

^{13}C NMR (CDCl$_3$, 101 MHz)

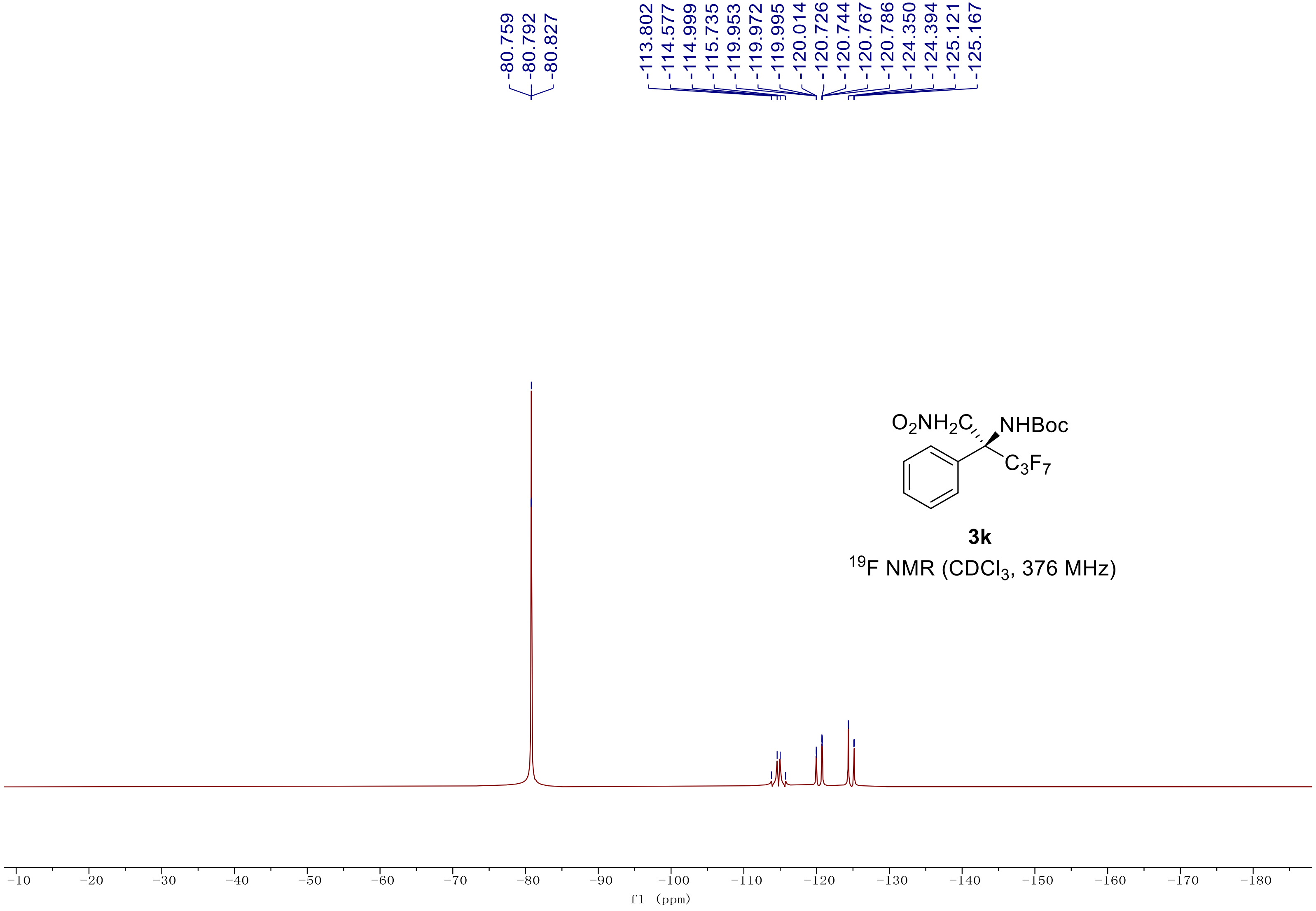

3k

^{19}F NMR ($CDCl_3$, 376 MHz)

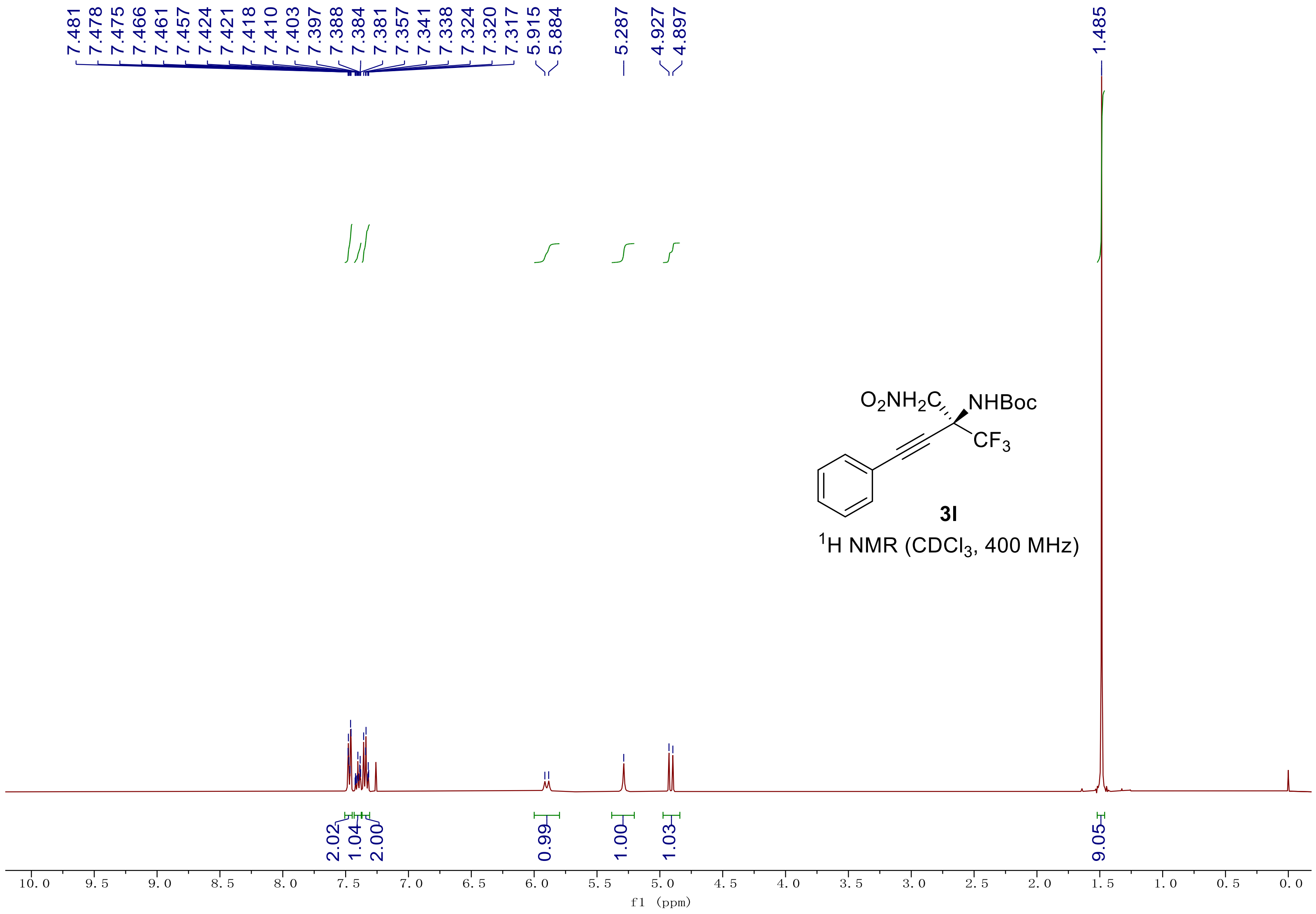
7.481
7.478
7.475
7.466
7.461
7.457
7.424
7.421
7.418
7.410
7.403
7.397
7.388
7.384
7.381
7.357
7.341
7.338
7.324
7.320
7.317
5.915
5.884
5.287
4.927
4.897
1.485
O_2NH_2C
NHBoc
CF_3
3l
^1H NMR ($CDCl_3$, 400 MHz)
2.02
1.04
2.00
0.99
1.00
1.03
9.05
f1 (ppm)

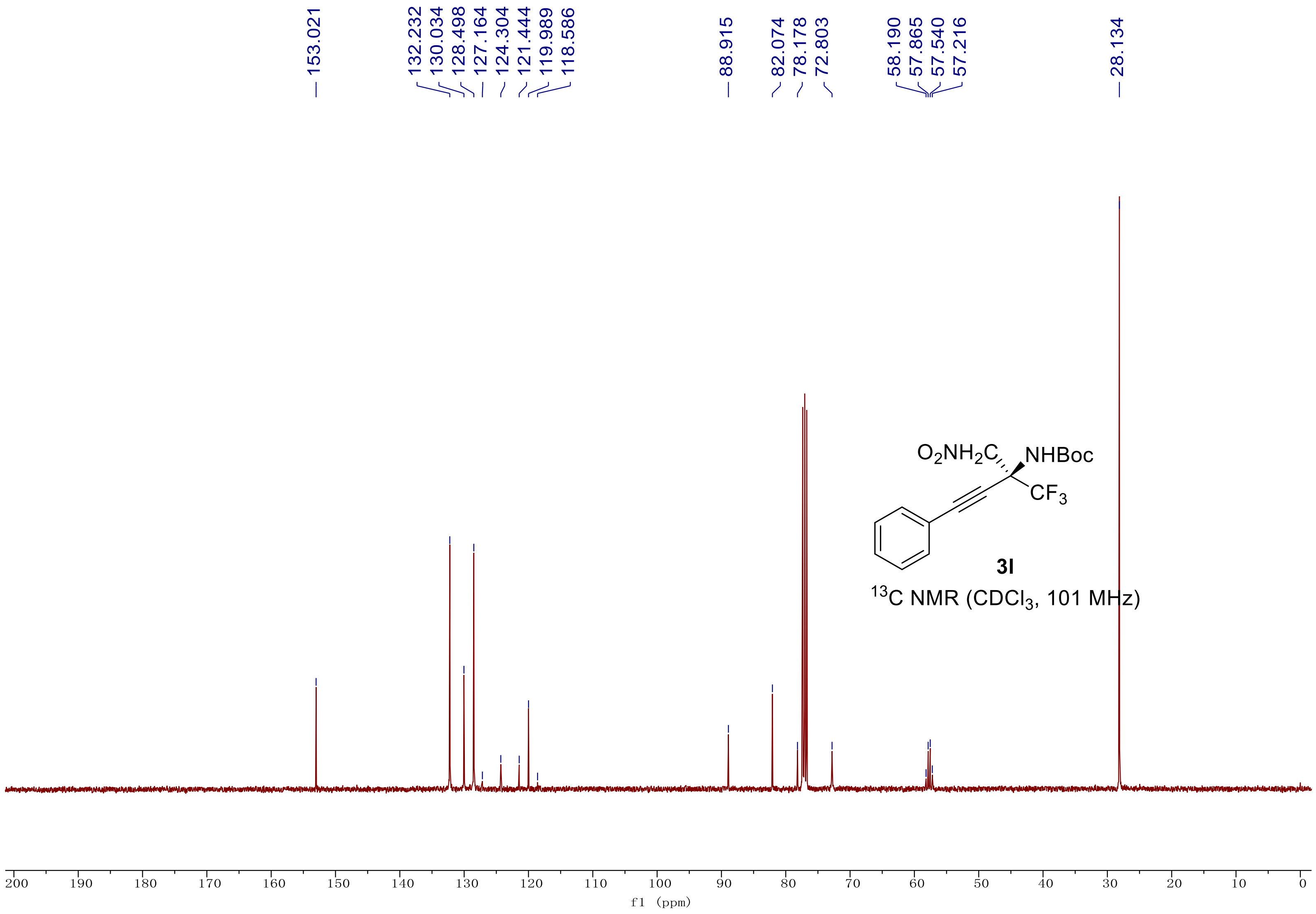

3l

^{13}C NMR ($CDCl_3$, 101 MHz)

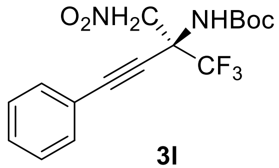

3l

^{19}F NMR ($CDCl_3$, 376 MHz)

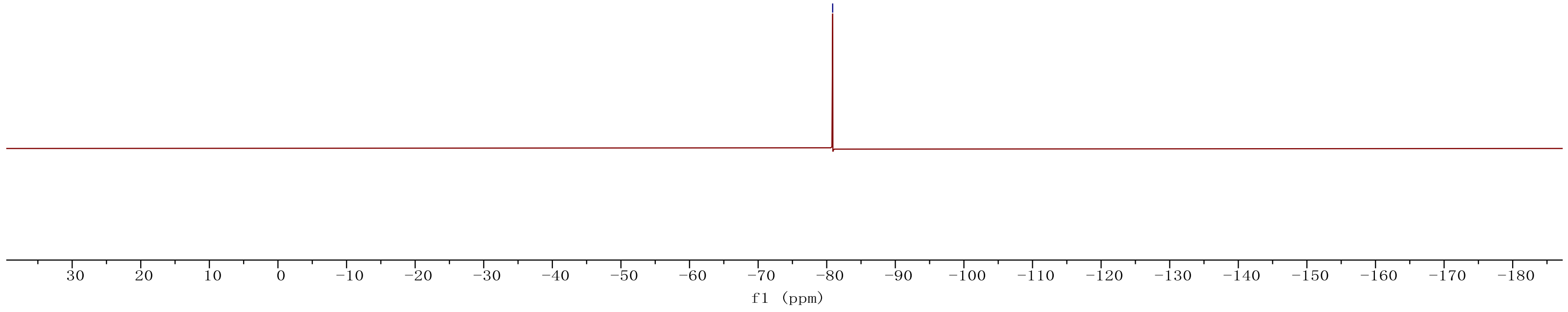

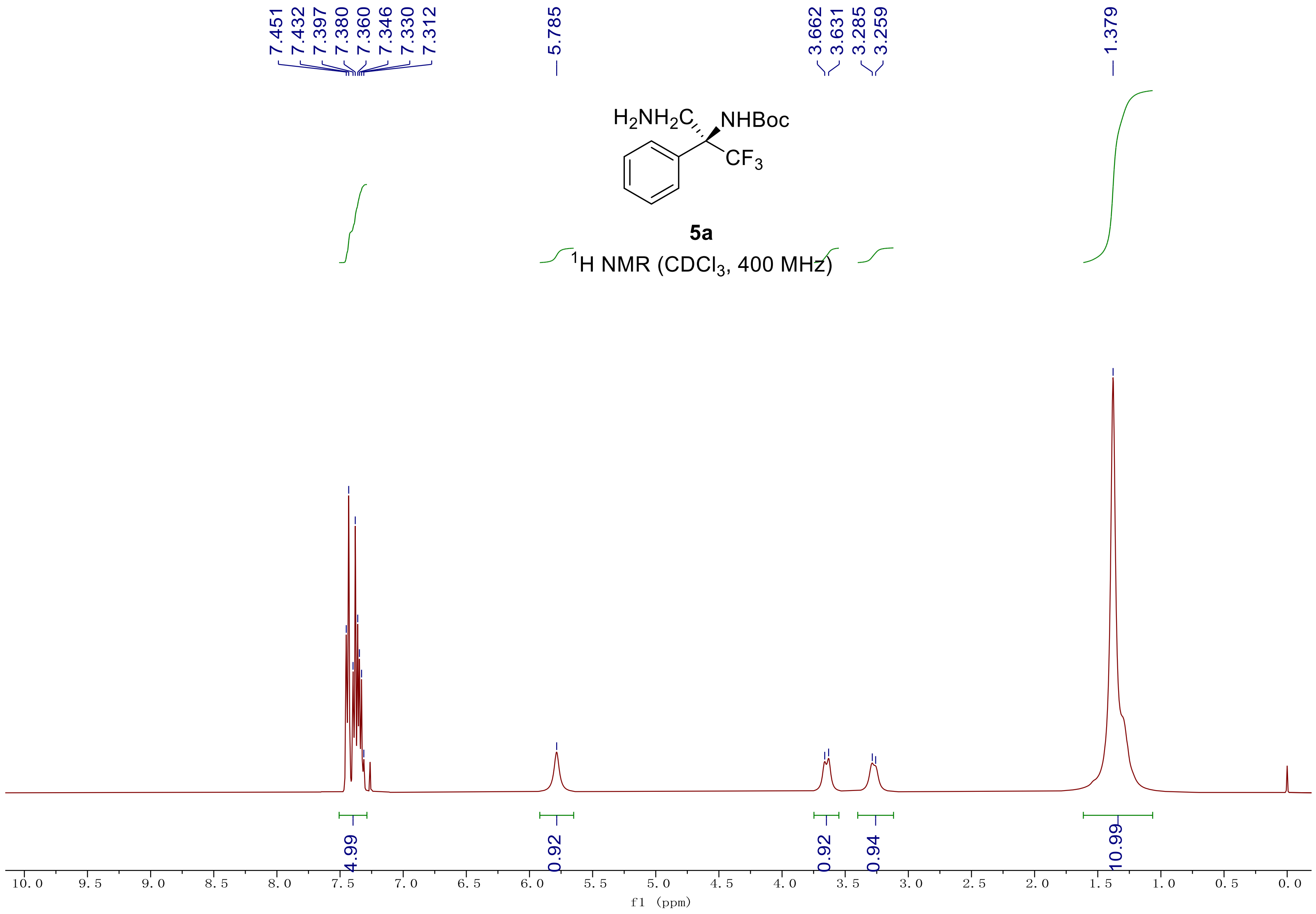
7.451
7.432
7.397
7.380
7.360
7.346
7.330
7.312
5.785
3.662
3.631
3.285
3.259
1.379
H2NH2C
NHBoc
CF3
5a
1H NMR (CDCl3, 400 MHz)
4.99
0.92
0.92
0.94
10.99
10.0 9.5 9.0 8.5 8.0 7.5 7.0 6.5 6.0 5.5 5.0 4.5 4.0 3.5 3.0 2.5 2.0 1.5 1.0 0.5 0.0
f1 (ppm)

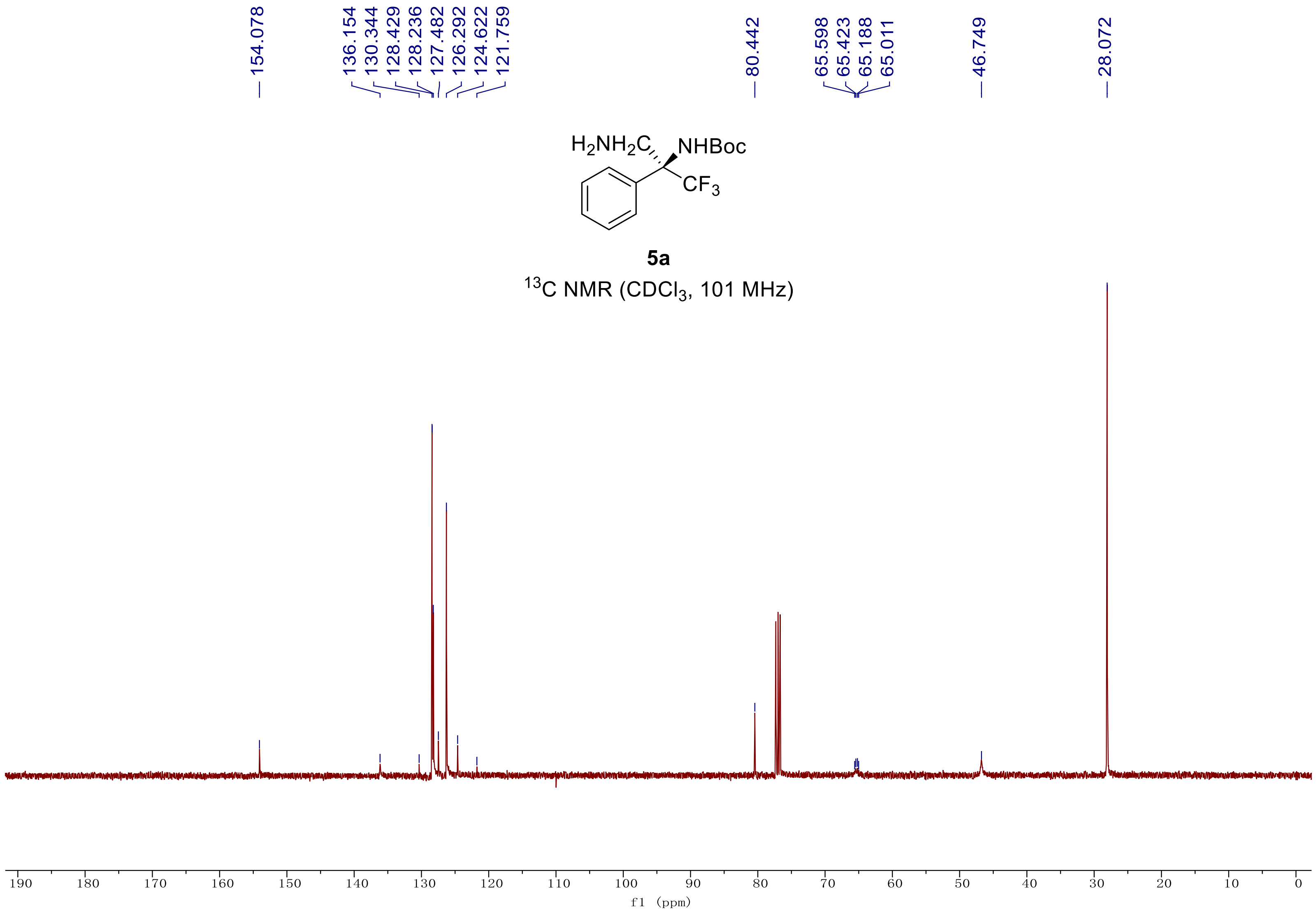
154.078
136.154
130.344
128.429
128.236
127.482
126.292
124.622
121.759
80.442
65.598
65.423
65.188
65.011
46.749
28.072
H2NH2C
NHBoc
CF3
5a
13C NMR (CDCl3, 101 MHz)
190 180 170 160 150 140 130 120 110 100 90 80 70 60 50 40 30 20 10 0
f1 (ppm)

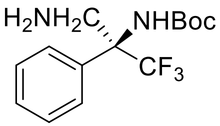

5a

^{19}F NMR ($CDCl_3$, 376 MHz)

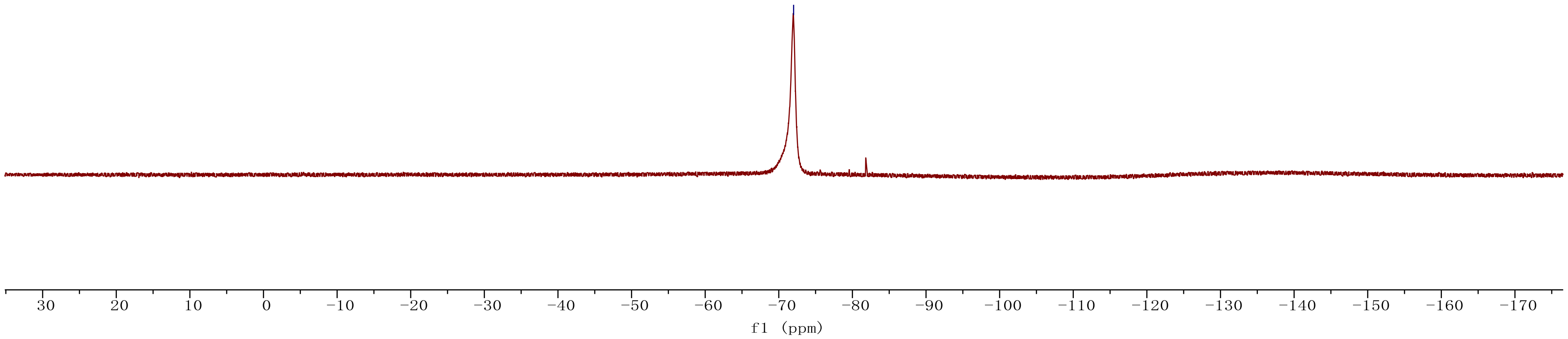

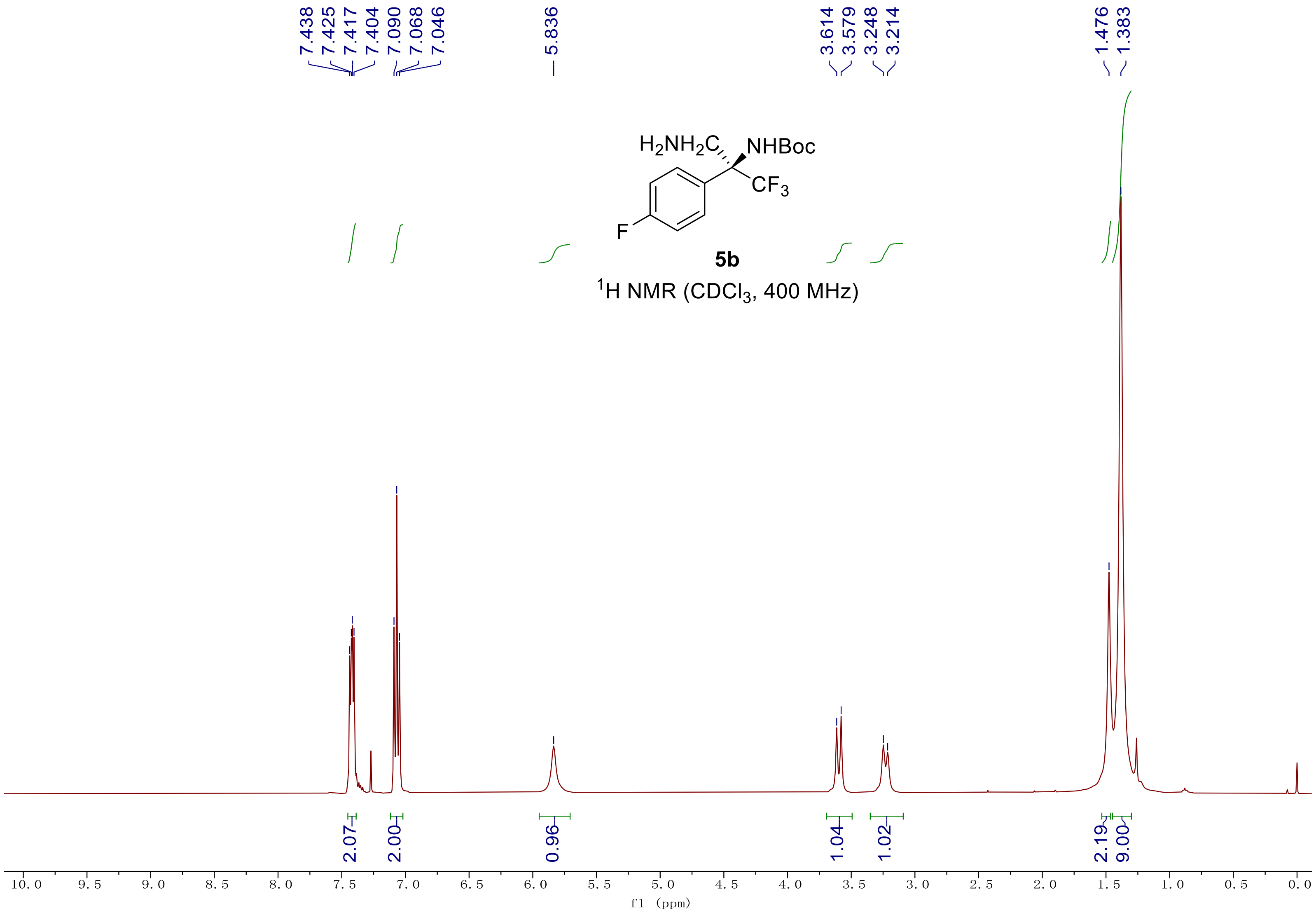

7.438
7.425
7.417
7.404
7.090
7.068
7.046
5.836
3.614
3.579
3.248
3.214
1.476
1.383
H2NH2C
NHBoc
CF3
F
5b
1H NMR (CDCl3, 400 MHz)
2.07
2.00
0.96
1.04
1.02
2.19
9.00
10.0
9.5
9.0
8.5
8.0
7.5
7.0
6.5
6.0
5.5
5.0
4.5
4.0
3.5
3.0
2.5
2.0
1.5
1.0
0.5
0.0
f1 (ppm)

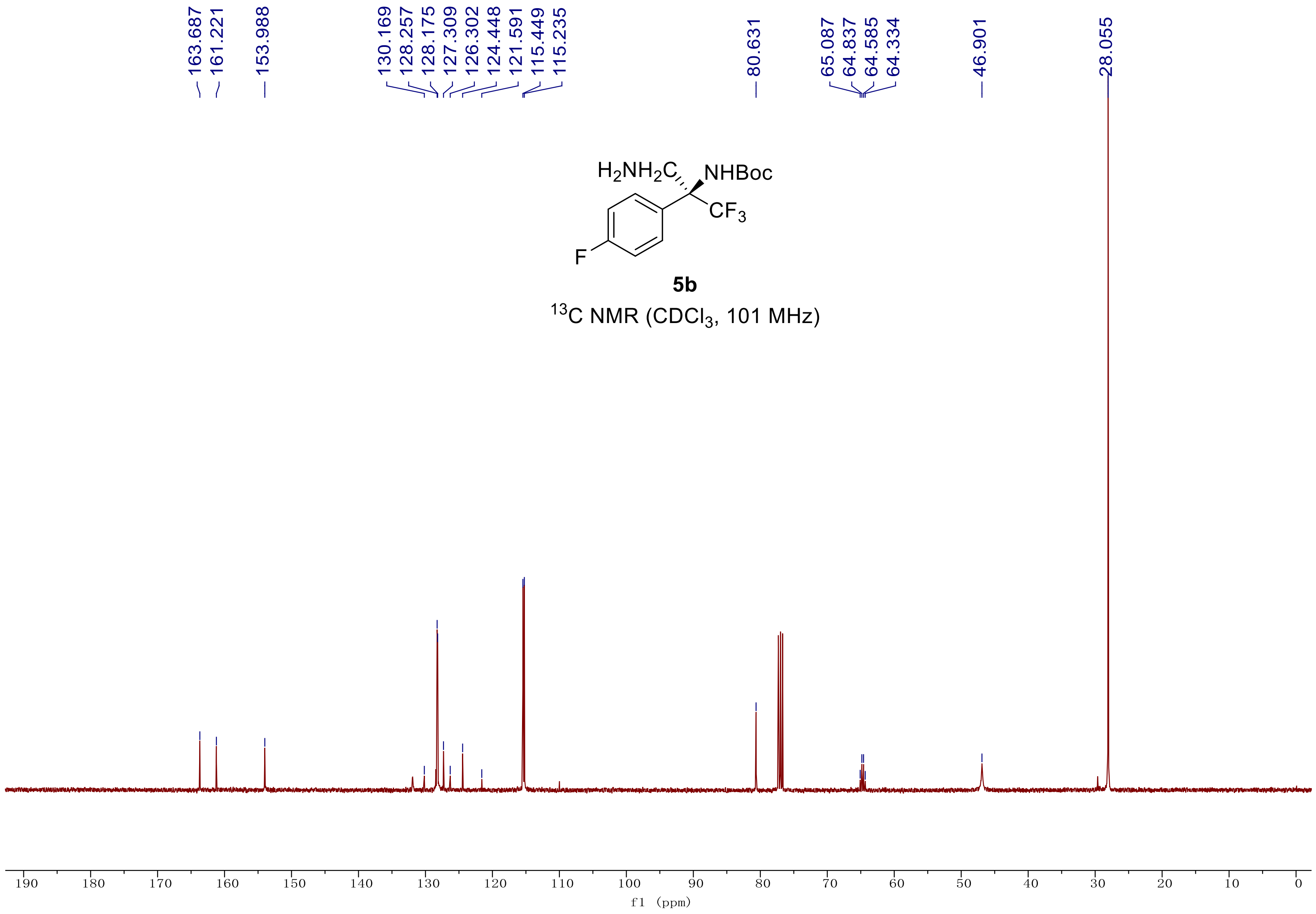

163.687
161.221
153.988
130.169
128.257
128.175
127.309
126.302
124.448
121.591
115.449
115.235
80.631
65.087
64.837
64.585
64.334
46.901
28.055
H2NH2C
NHBoc
CF3
F
5b
13C NMR (CDCl3, 101 MHz)
190 180 170 160 150 140 130 120 110 100 90 80 70 60 50 40 30 20 10 0
f1 (ppm)

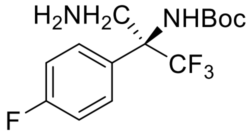

5b

^{19}F NMR ($CDCl_3$, 376 MHz)

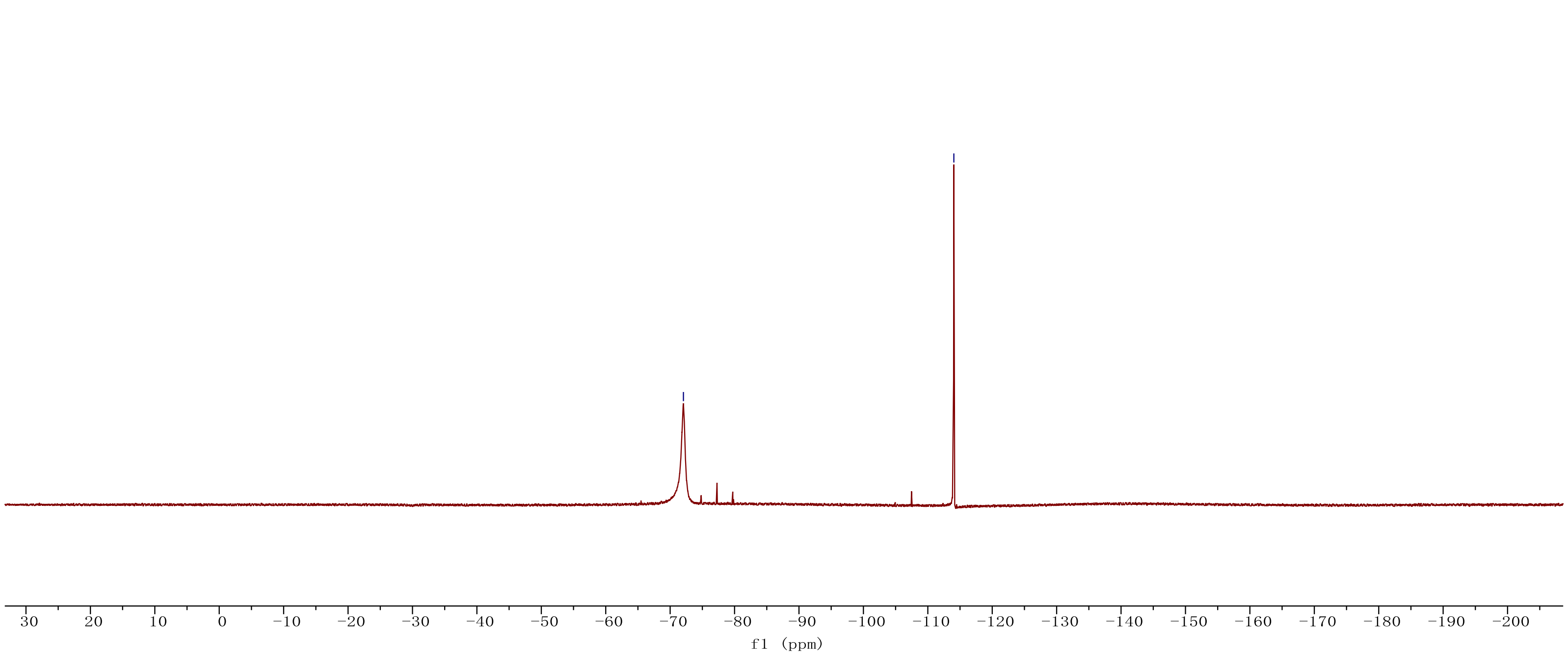

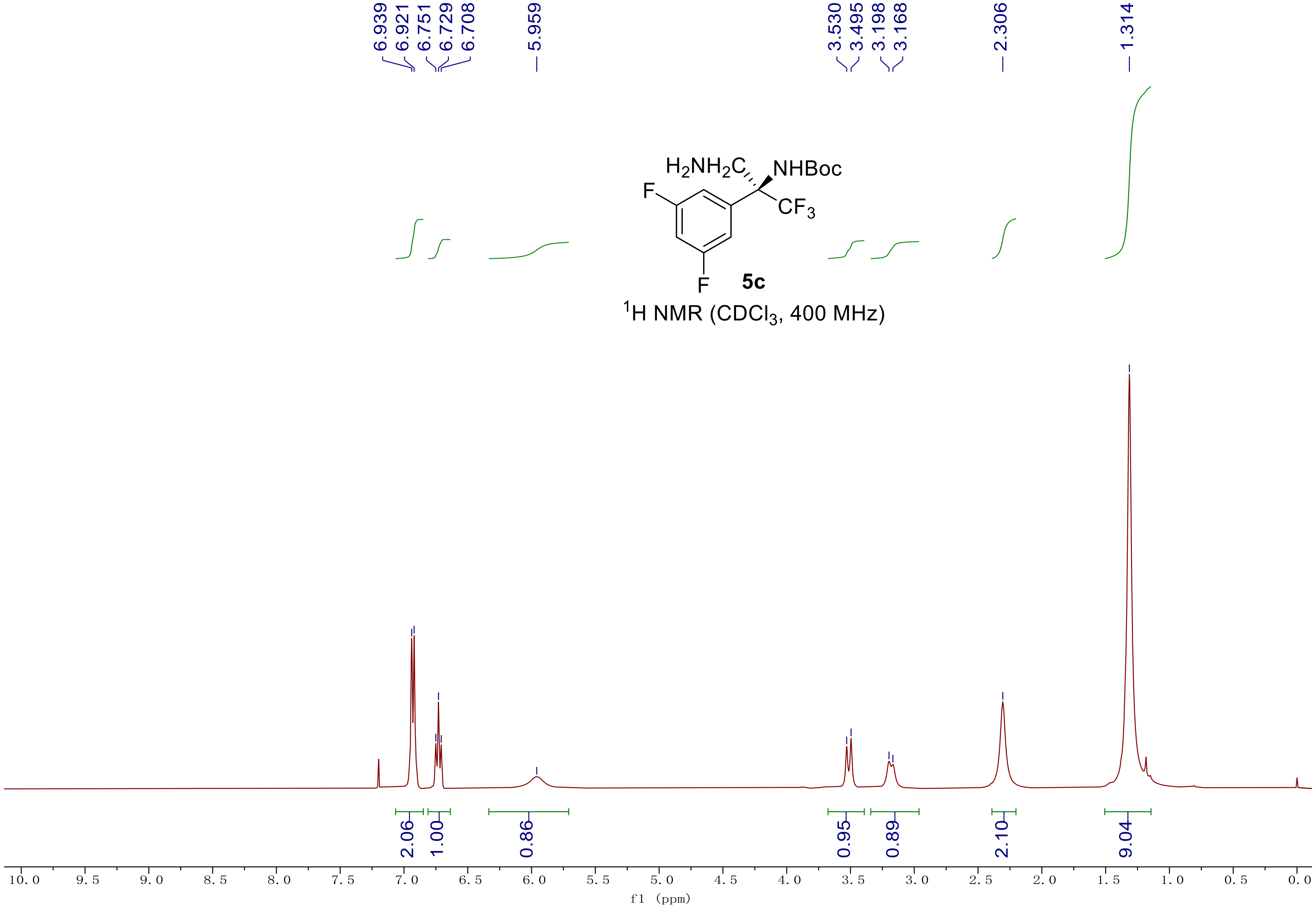
6.939
6.921
6.751
6.729
6.708
5.959
3.530
3.495
3.198
3.168
2.306
1.314
H2NH2C
NHBoc
F
CF3
F
5c
1H NMR (CDCl3, 400 MHz)
2.06
1.00
0.86
0.95
0.89
2.10
9.04
10.0
9.5
9.0
8.5
8.0
7.5
7.0
6.5
6.0
5.5
5.0
4.5
4.0
3.5
3.0
2.5
2.0
1.5
1.0
0.5
0.0
f1 (ppm)

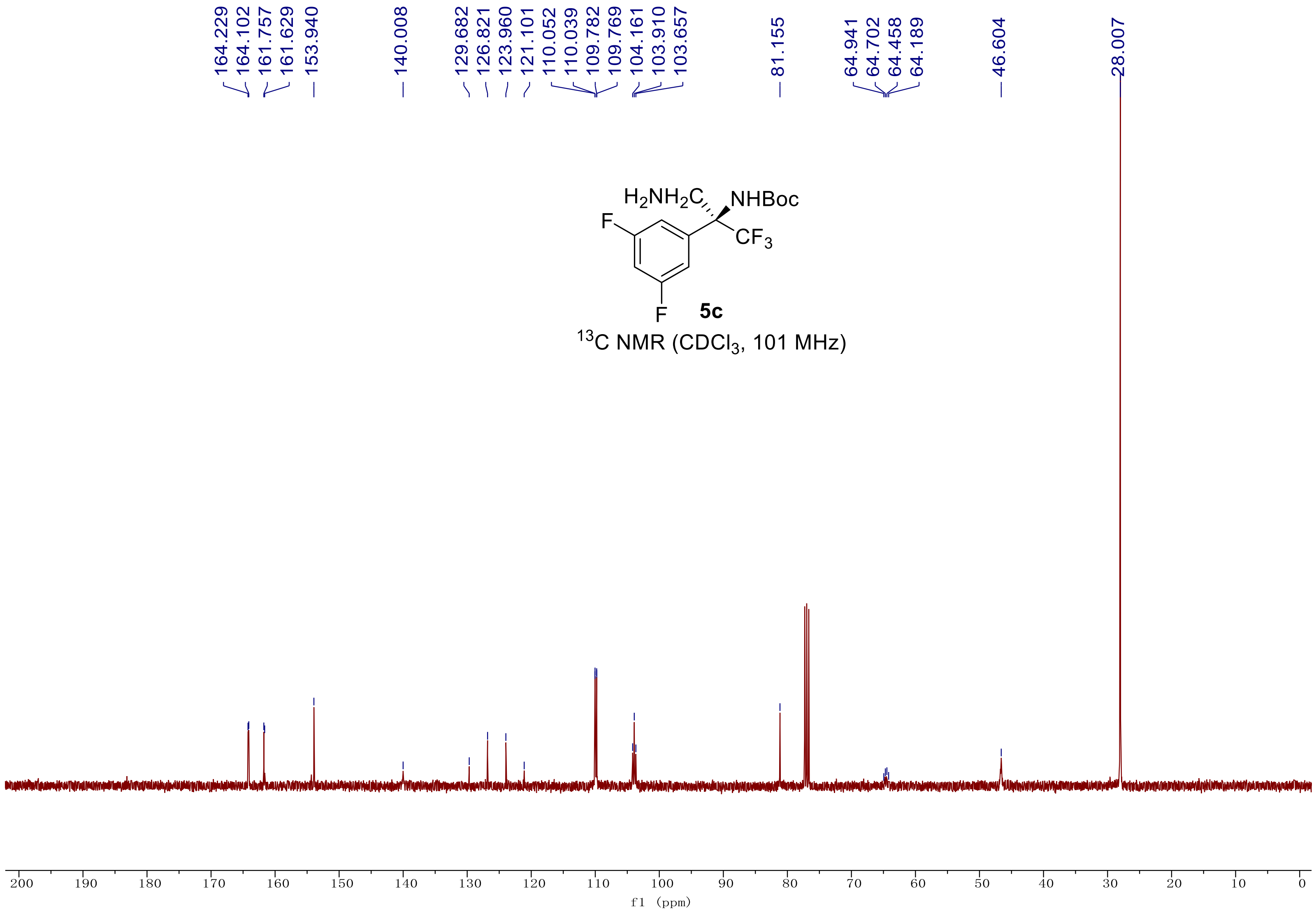
164.229
164.102
161.757
161.629
153.940
140.008
129.682
126.821
123.960
121.101
110.052
110.039
109.782
109.769
104.161
103.910
103.657
81.155
64.941
64.702
64.458
64.189
46.604
28.007
H_2NH_2C
NHBoc
F
CF_3
F
5c
^{13}C NMR ($CDCl_3$, 101 MHz)
200 190 180 170 160 150 140 130 120 110 100 90 80 70 60 50 40 30 20 10 0
f1 (ppm)

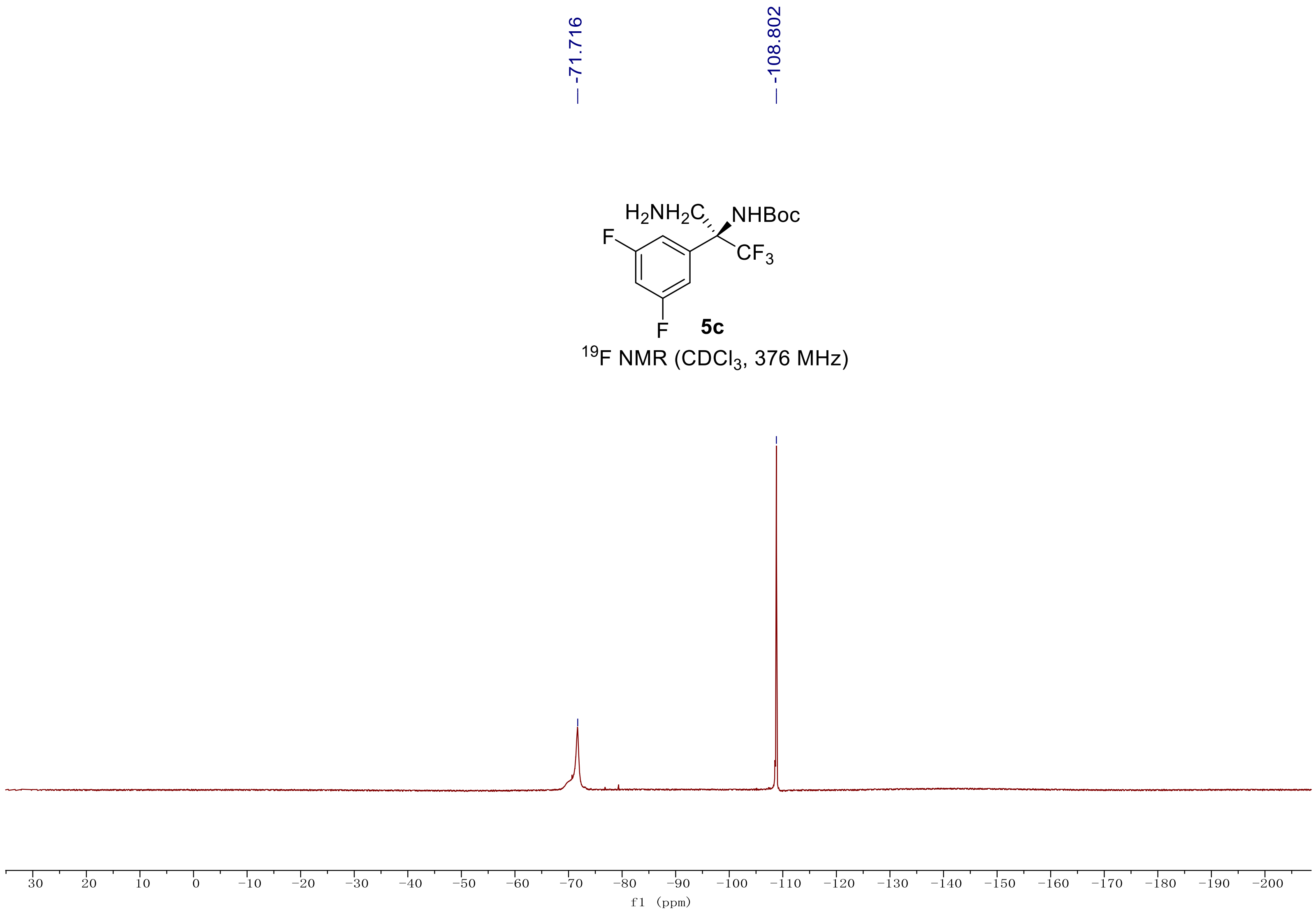

-71.716
-108.802
H2NH2C
NHBoc
F
CF3
F
5c
19F NMR (CDCl3, 376 MHz)
30 20 10 0 −10 −20 −30 −40 −50 −60 −70 −80 −90 −100 −110 −120 −130 −140 −150 −160 −170 −180 −190 −200
f1 (ppm)

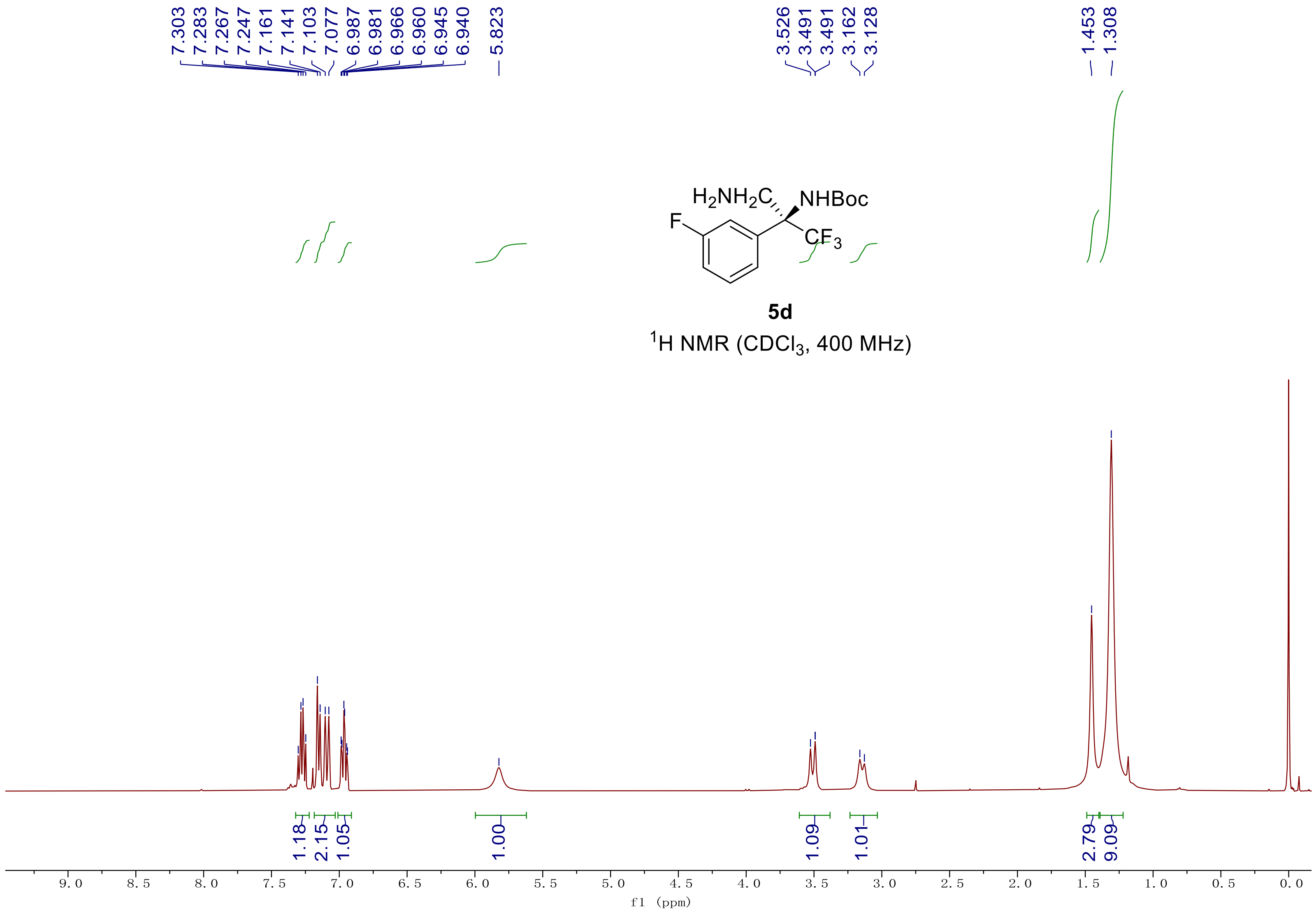
7.303
7.283
7.267
7.247
7.161
7.141
7.103
7.077
6.987
6.981
6.966
6.960
6.945
6.940
5.823
3.526
3.491
3.491
3.162
3.128
1.453
1.308
H2NH2C
NHBoc
F
CF3
5d
1H NMR (CDCl3, 400 MHz)
1.18
2.15
1.05
1.00
1.09
1.01
2.79
9.09
9.0
8.5
8.0
7.5
7.0
6.5
6.0
5.5
5.0
4.5
4.0
3.5
3.0
2.5
2.0
1.5
1.0
0.5
0.0
f1 (ppm)

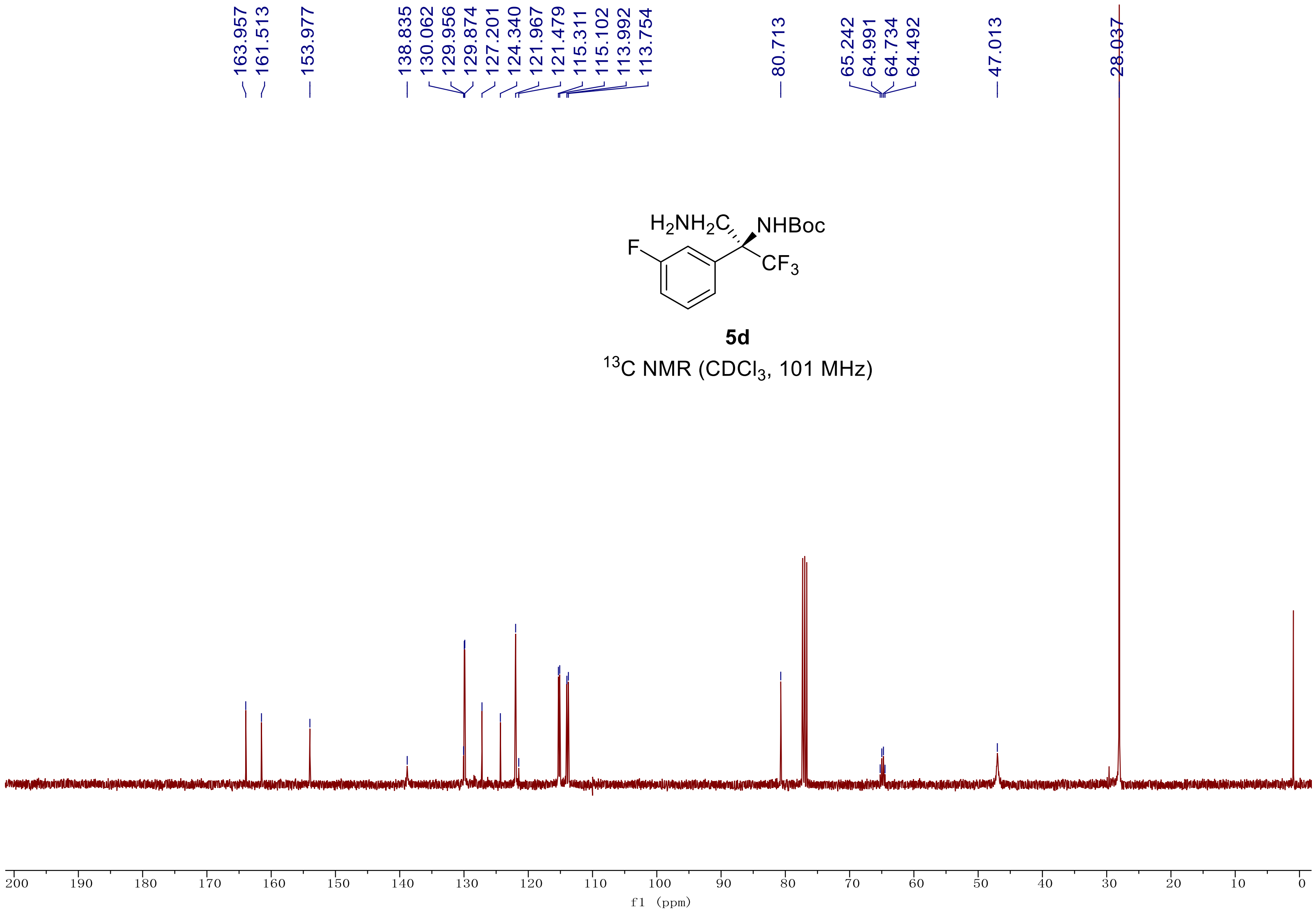

5d

^{13}C NMR ($CDCl_3$, 101 MHz)

-72.334
-112.254

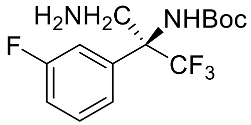

5d

^{19}F NMR ($CDCl_3$, 376 MHz)

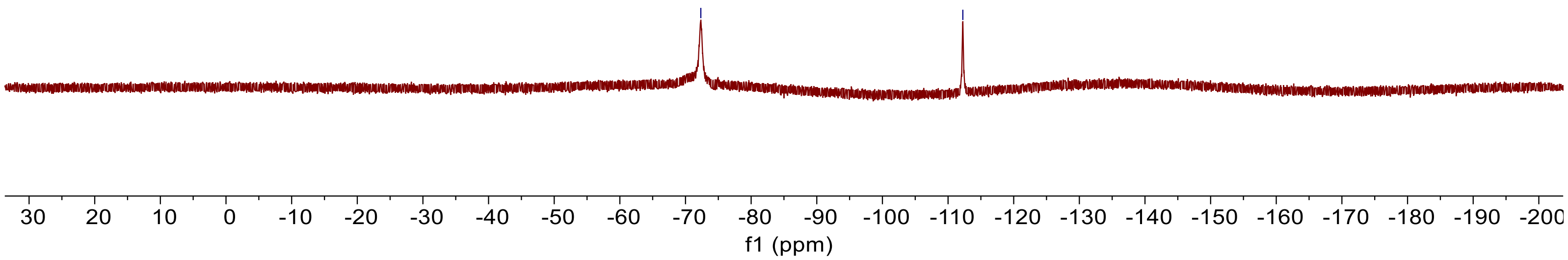

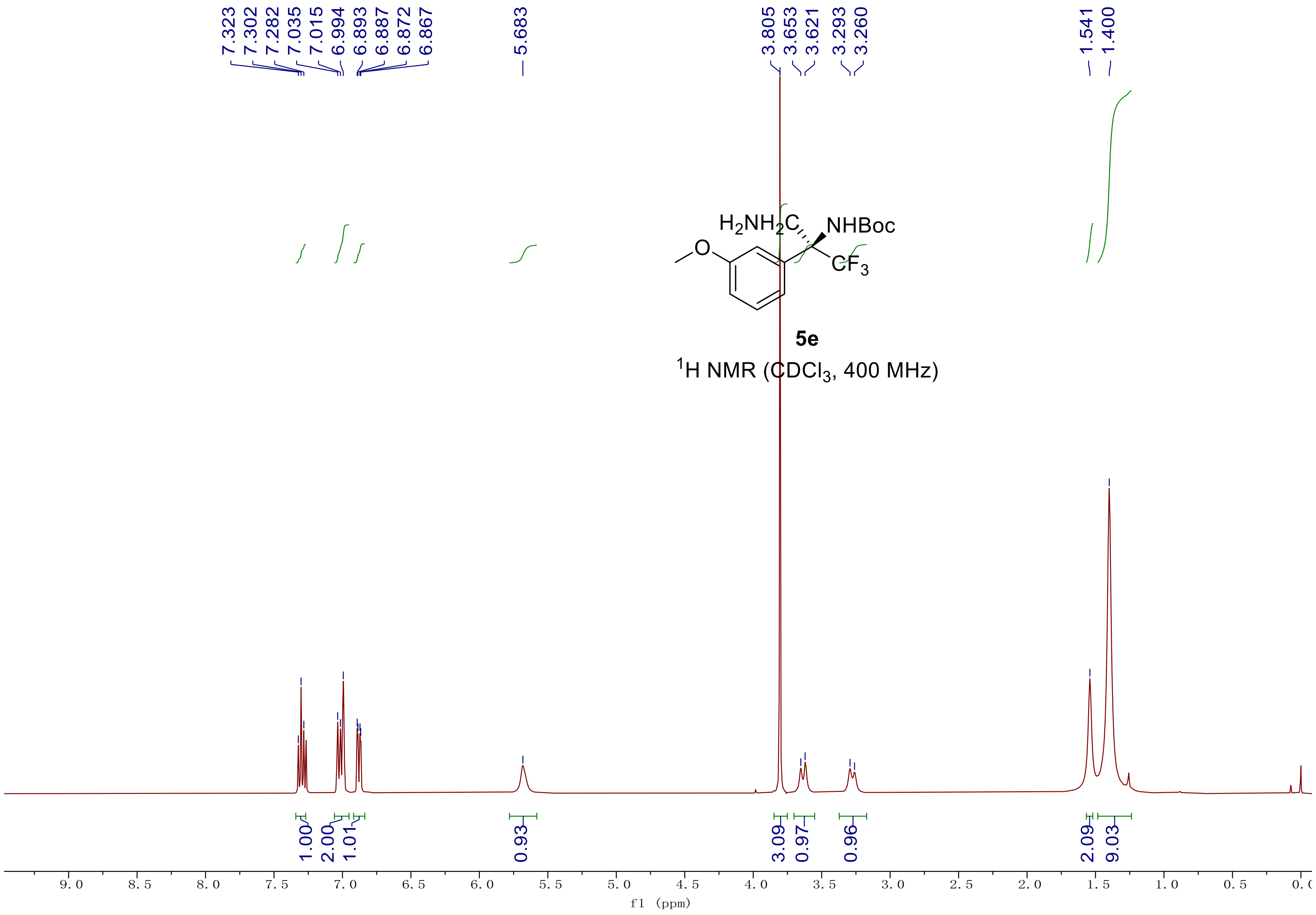
7.323
7.302
7.282
7.035
7.015
6.994
6.893
6.887
6.872
6.867
5.683
3.805
3.653
3.621
3.293
3.260
1.541
1.400
H2NH2C
NHBoc
O
CF3
5e
1H NMR (CDCl3, 400 MHz)
1.00
2.00
1.01
0.93
3.09
0.97
0.96
2.09
9.03
9.0
8.5
8.0
7.5
7.0
6.5
6.0
5.5
5.0
4.5
4.0
3.5
3.0
2.5
2.0
1.5
1.0
0.5
f1 (ppm)

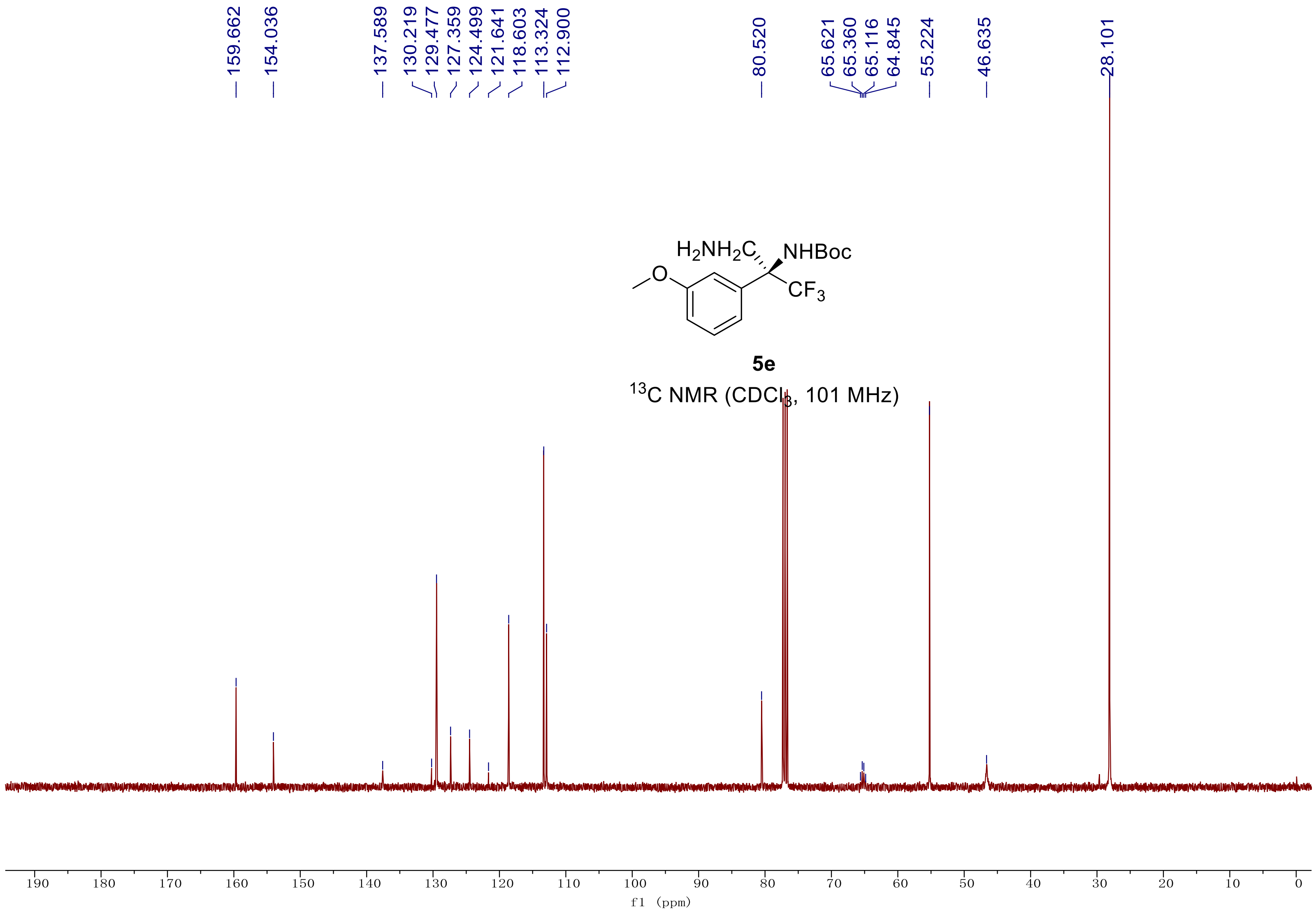
159.662
154.036
137.589
130.219
129.477
127.359
124.499
121.641
118.603
113.324
112.900
80.520
65.621
65.360
65.116
64.845
55.224
46.635
28.101
H2NH2C
NHBoc
O
CF3
5e
13C NMR (CDCl3, 101 MHz)
190 180 170 160 150 140 130 120 110 100 90 80 70 60 50 40 30 20 10 0
f1 (ppm)

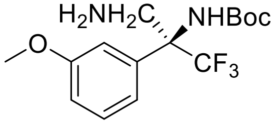

5e

^{19}F NMR ($CDCl_3$, 376 MHz)

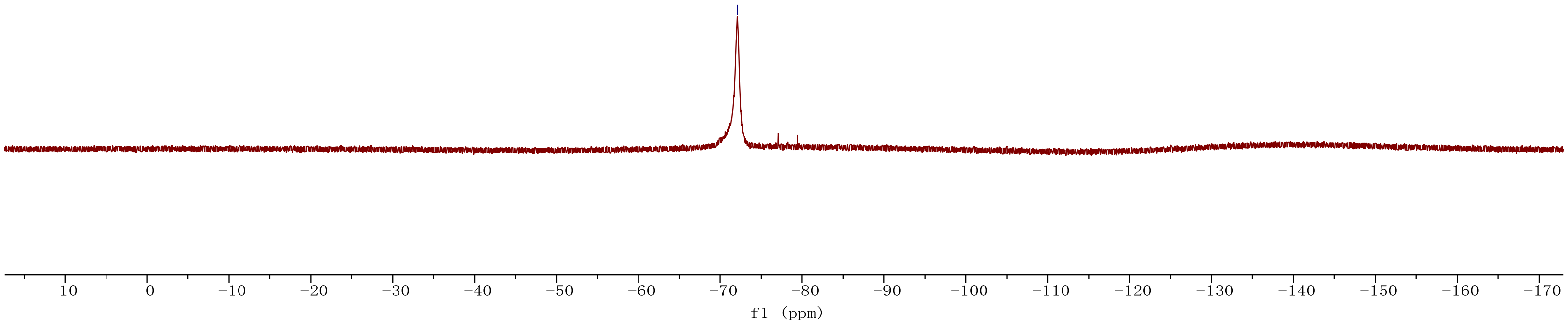

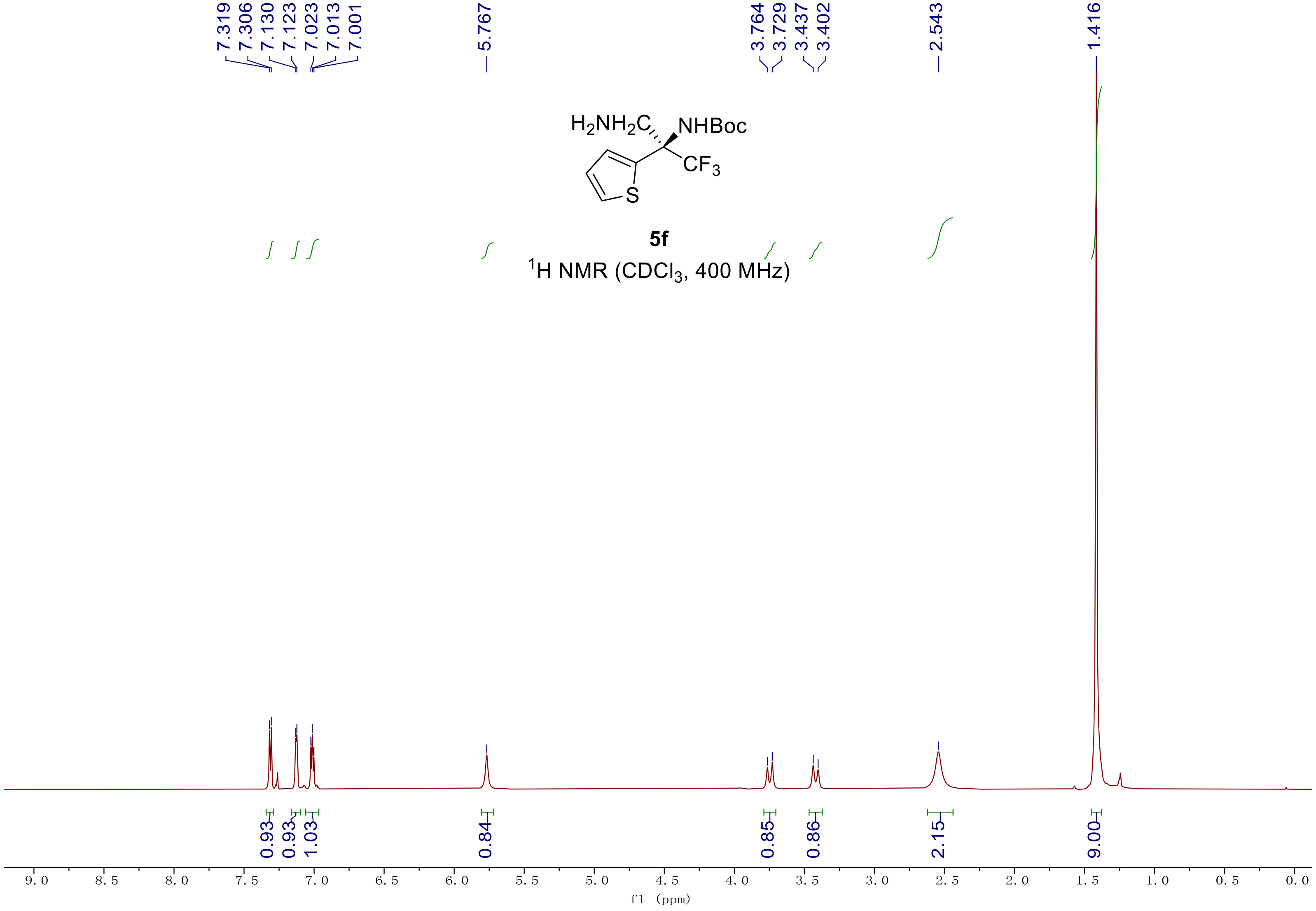
7.319
7.306
7.130
7.123
7.023
7.013
7.001
5.767
3.764
3.729
3.437
3.402
2.543
1.416
H2NH2C
NHBoc
CF3
S
5f
1H NMR (CDCl3, 400 MHz)
0.93
0.93
1.03
0.84
0.85
0.86
2.15
9.00
9.0
8.5
8.0
7.5
7.0
6.5
6.0
5.5
5.0
4.5
4.0
3.5
3.0
2.5
2.0
1.5
1.0
0.5
0.0
f1 (ppm)

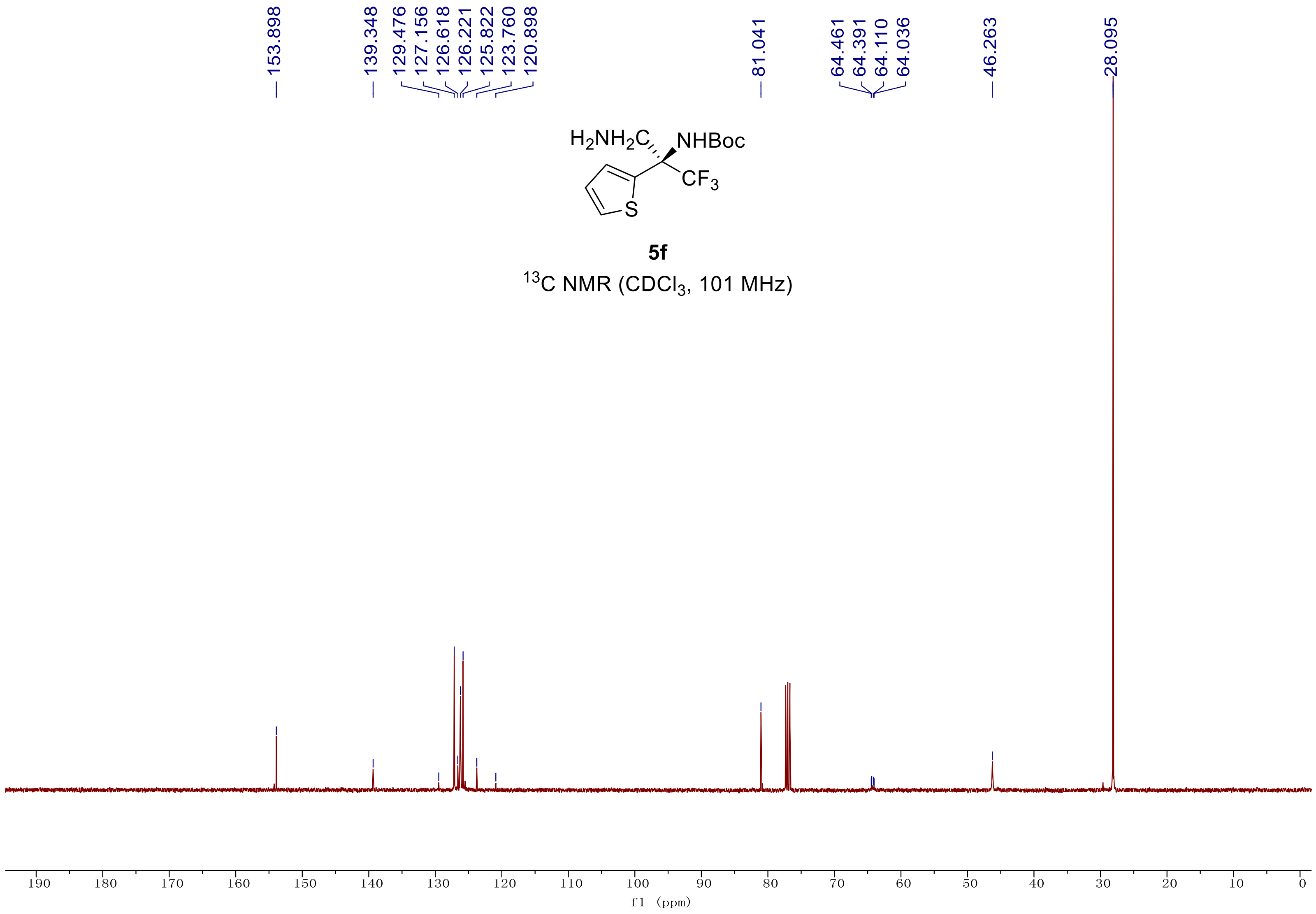

5f

^{13}C NMR ($CDCl_3$, 101 MHz)

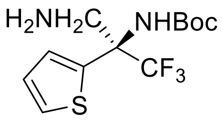

5f

^{19}F NMR (CDCl$_3$, 376 MHz)

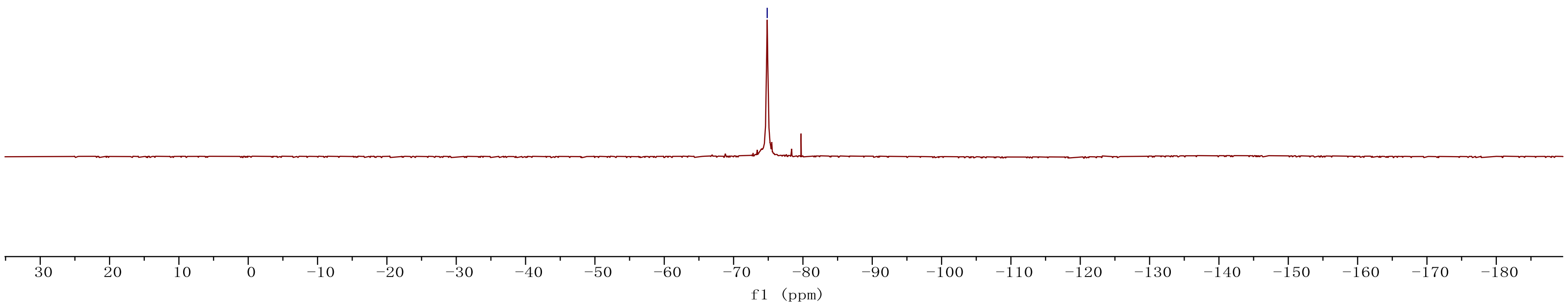

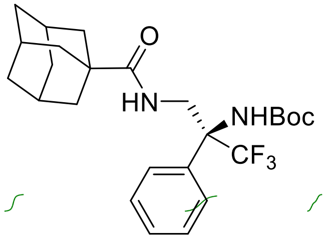

6a

^{1}H NMR (CDCl3, 400 MHz)

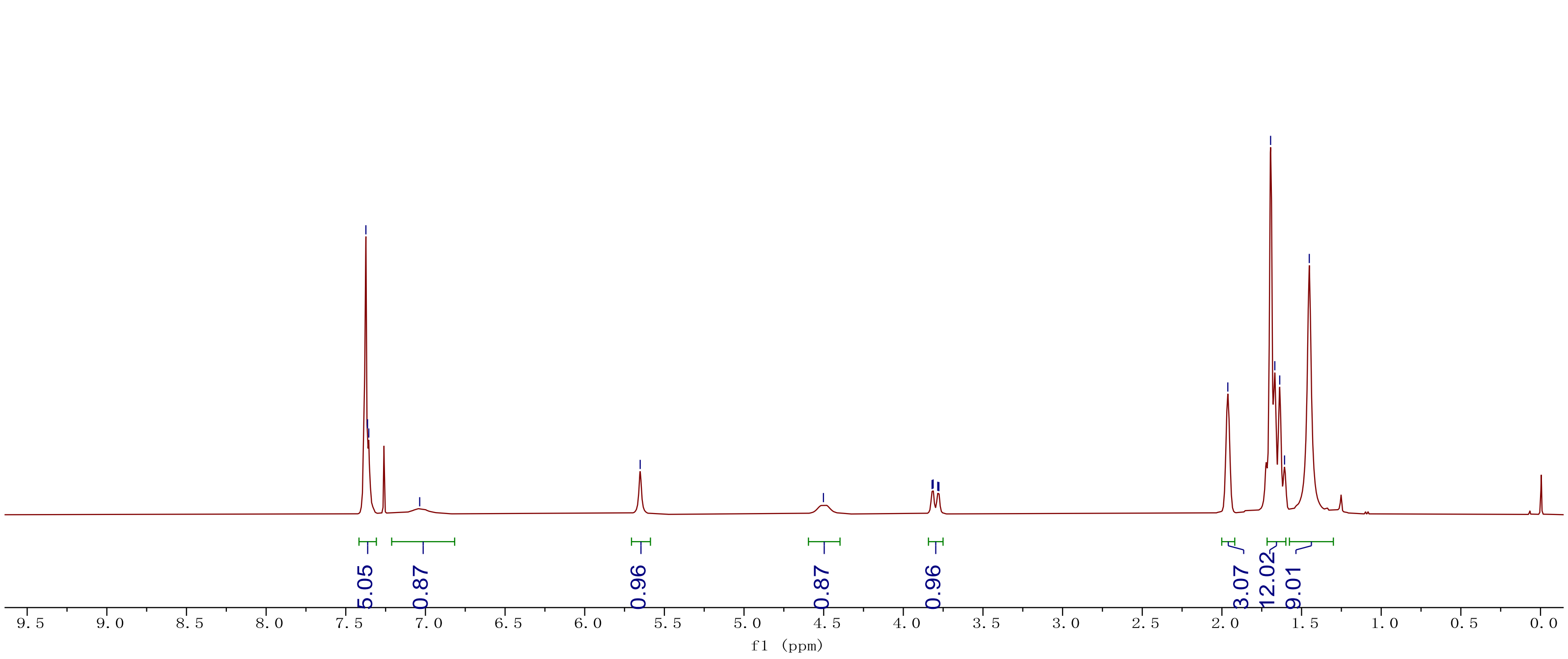

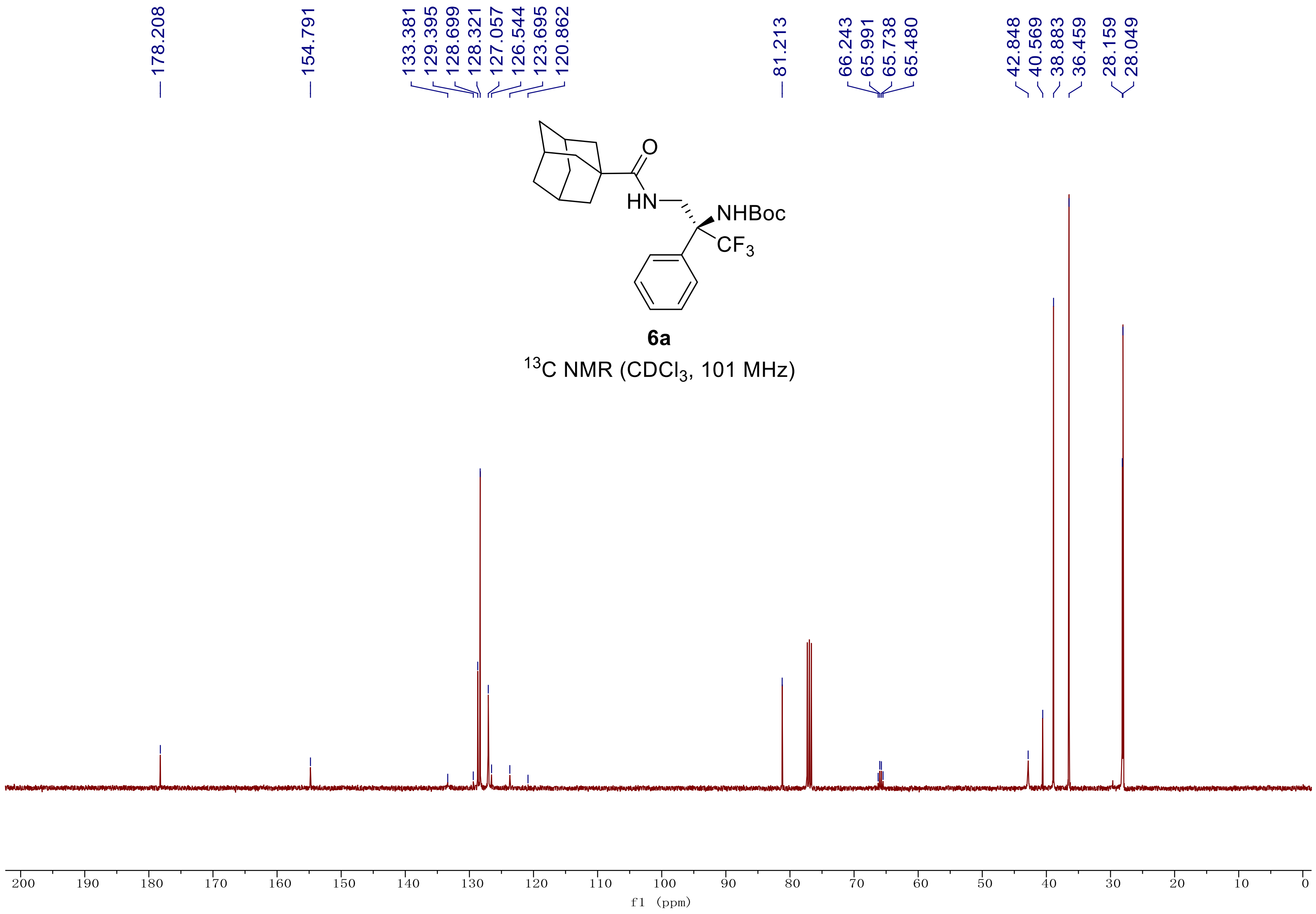

6a

^{13}C NMR ($CDCl_3$, 101 MHz)

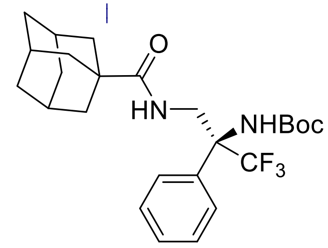

6a

^{19}F NMR ($CDCl_3$, 376 MHz)

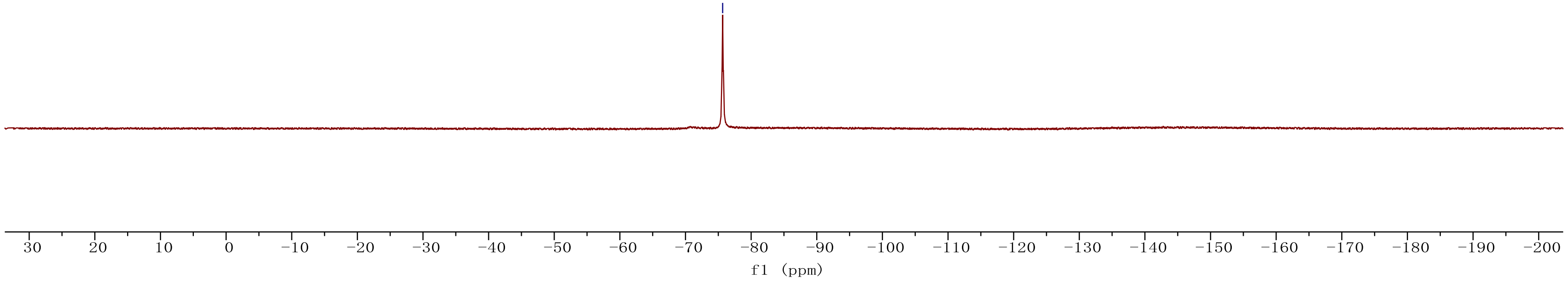

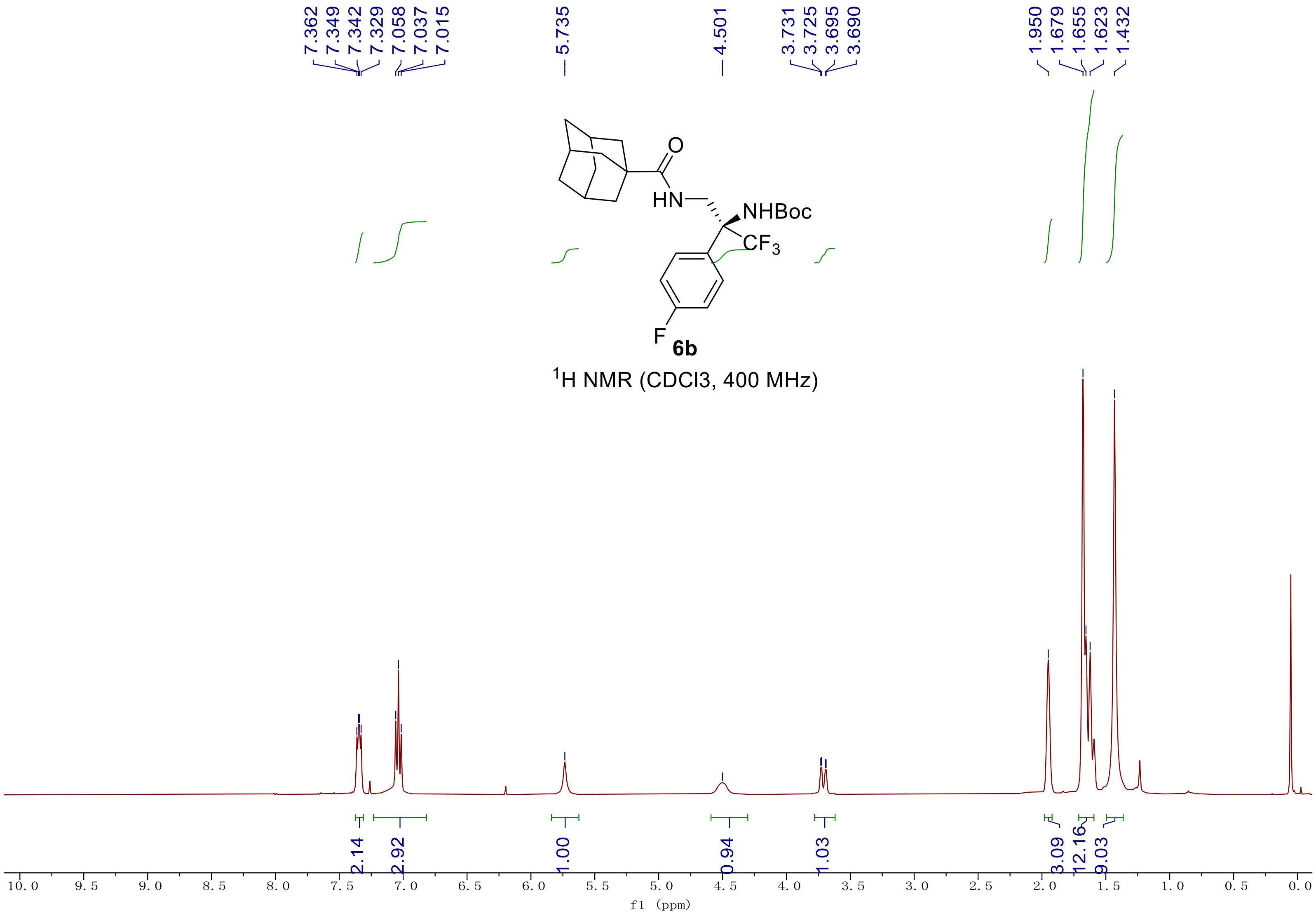
7.362
7.349
7.342
7.329
7.058
7.037
7.015
5.735
4.501
3.731
3.725
3.695
3.690
1.950
1.679
1.655
1.623
1.432
O
HN
NHBoc
CF_3
F
6b
^{1}H NMR (CDCl3, 400 MHz)
2.14
2.92
1.00
0.94
1.03
3.09
12.16
9.03
10.0 9.5 9.0 8.5 8.0 7.5 7.0 6.5 6.0 5.5 5.0 4.5 4.0 3.5 3.0 2.5 2.0 1.5 1.0 0.5 0.0
f1 (ppm)

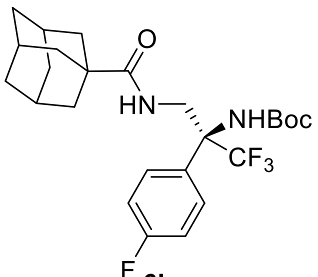

6b

^{13}C NMR ($CDCl_3$, 101 MHz)

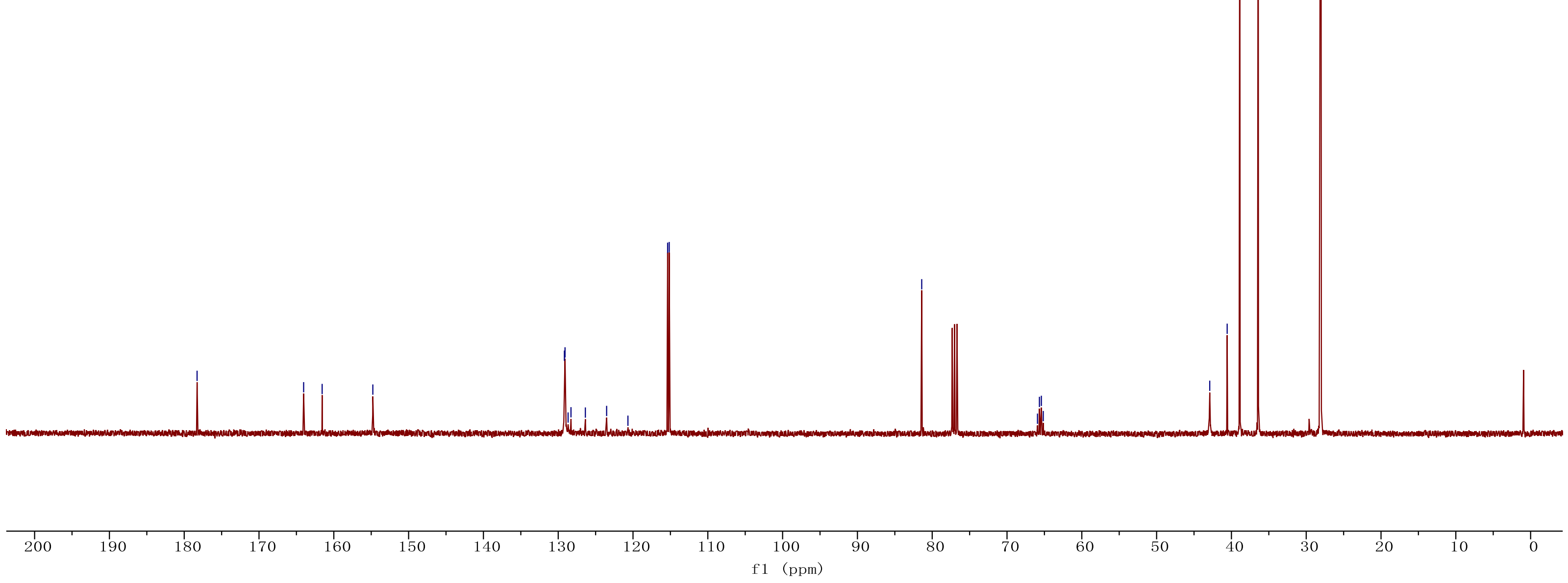

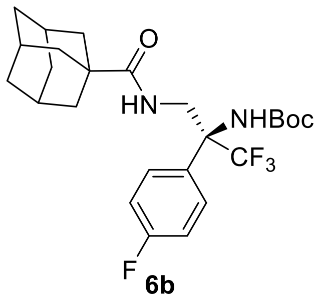

6b

^{19}F NMR ($CDCl_3$, 376 MHz)

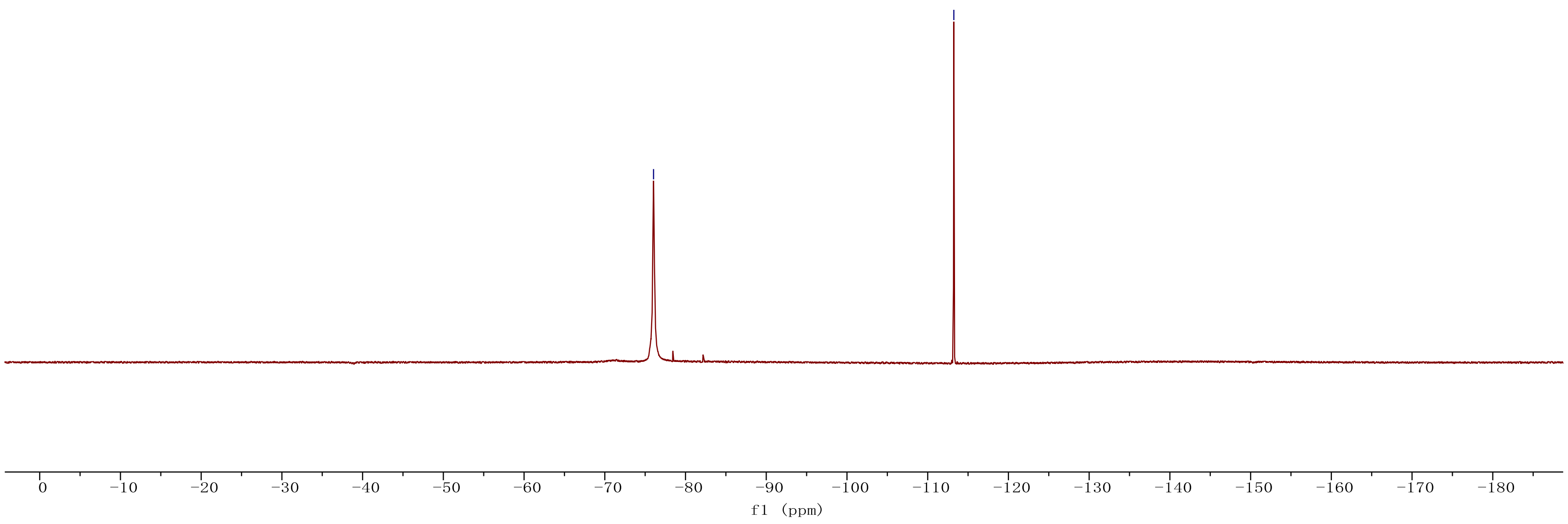

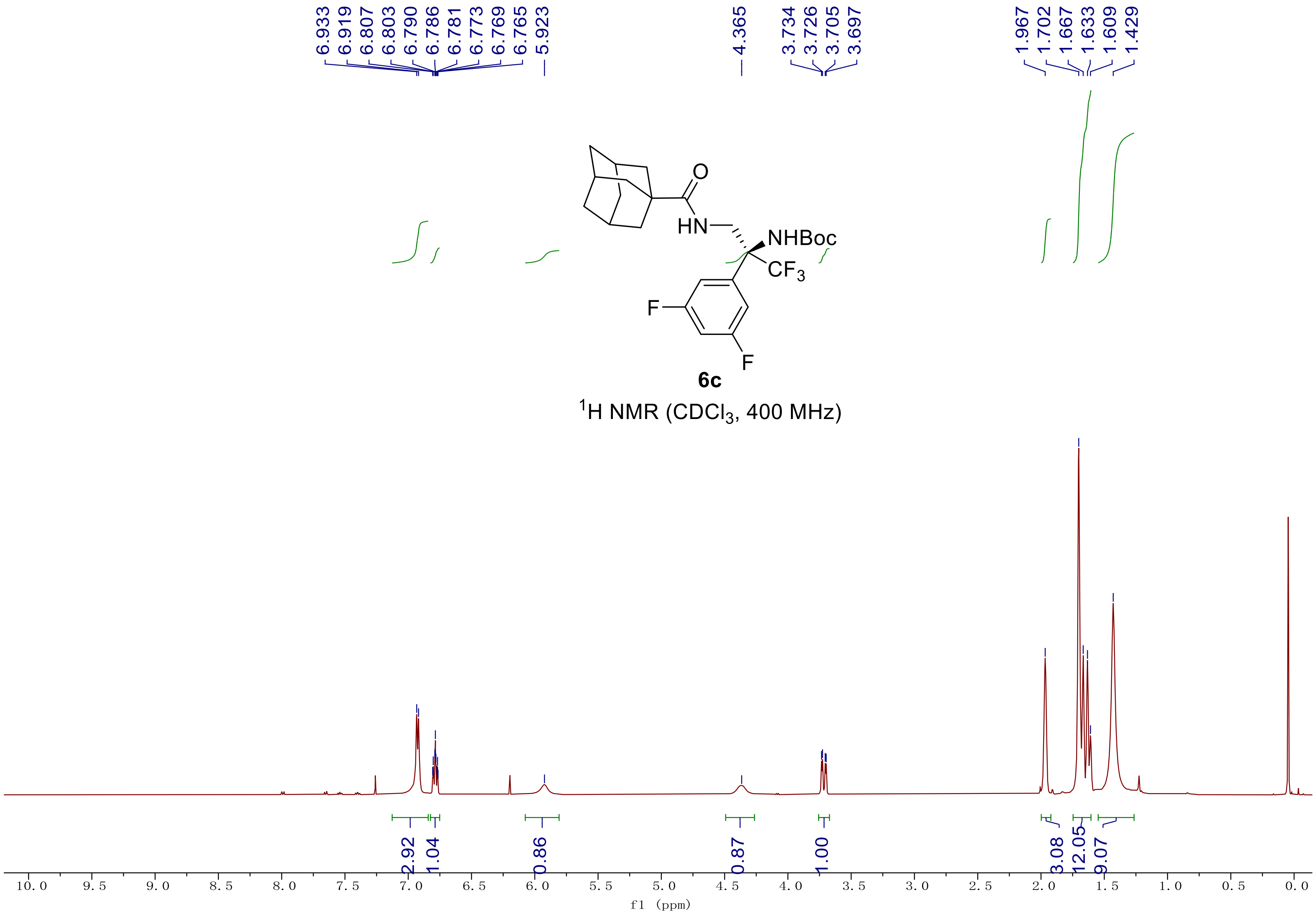

6c

^{1}H NMR ($CDCl_3$, 400 MHz)

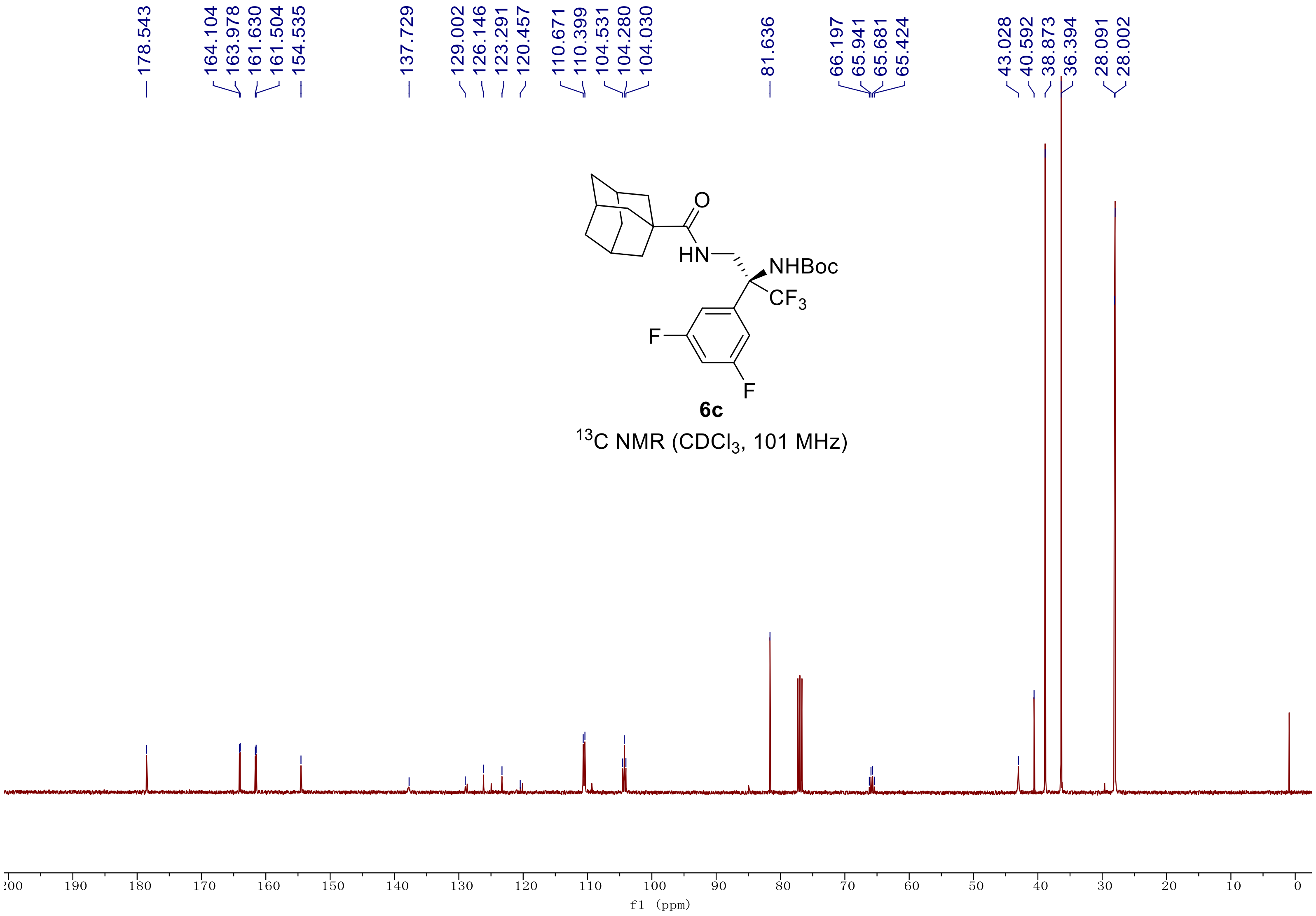

6c

^{13}C NMR ($CDCl_3$, 101 MHz)

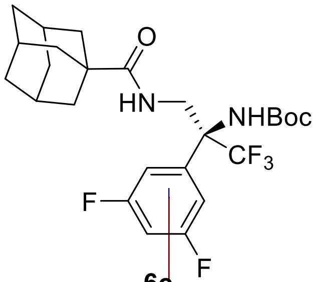

6c

^{19}F NMR ($CDCl_3$, 376 MHz)

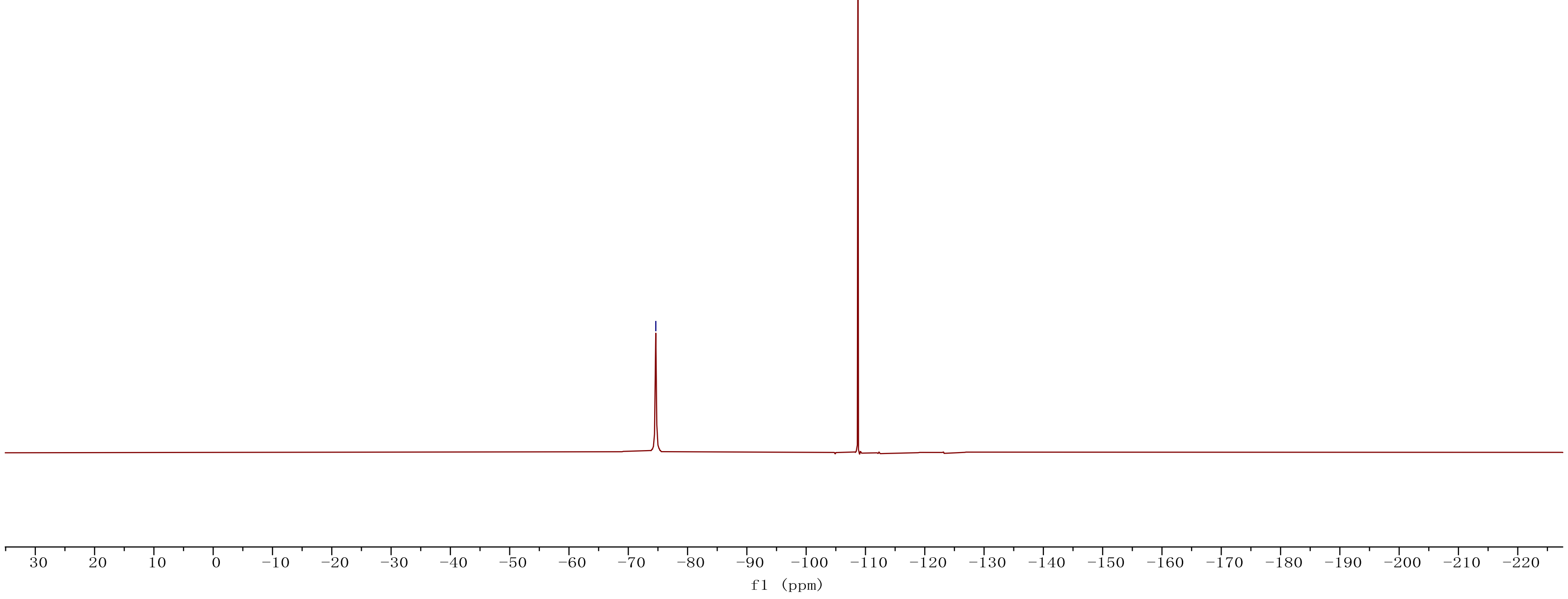

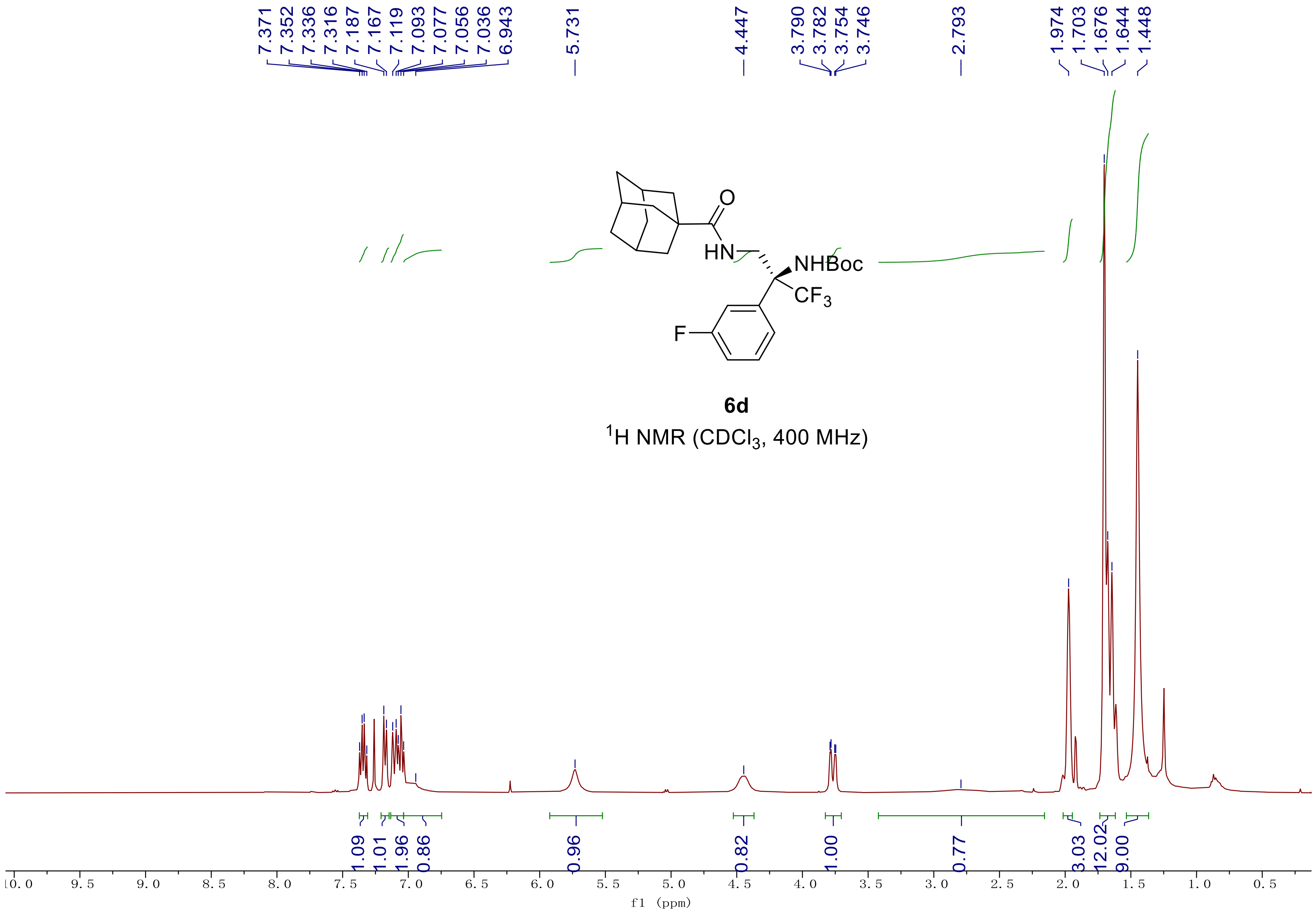

6d

^{1}H NMR ($CDCl_3$, 400 MHz)

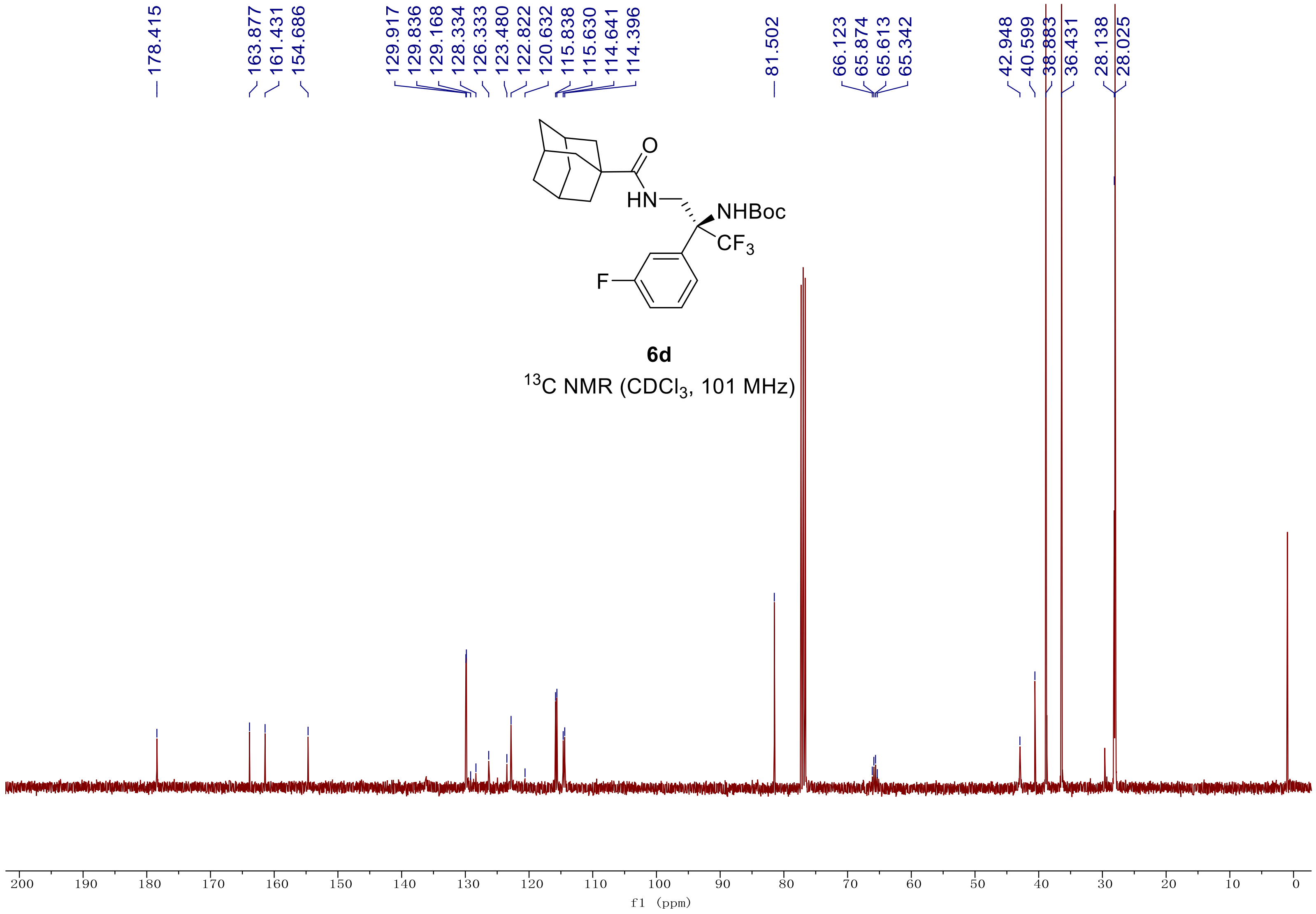

6d

^{13}C NMR ($CDCl_3$, 101 MHz)

-75.206
-112.250

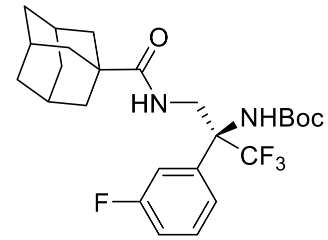

6d

^{19}F NMR ($CDCl_3$, 376 MHz)

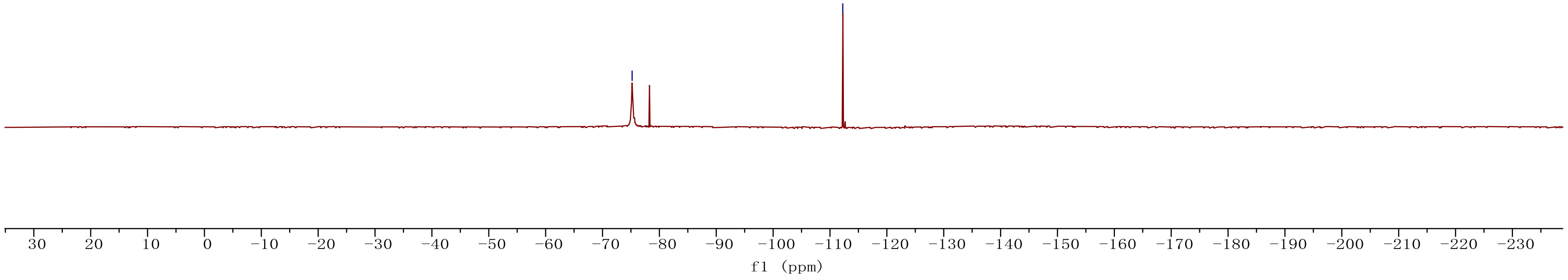

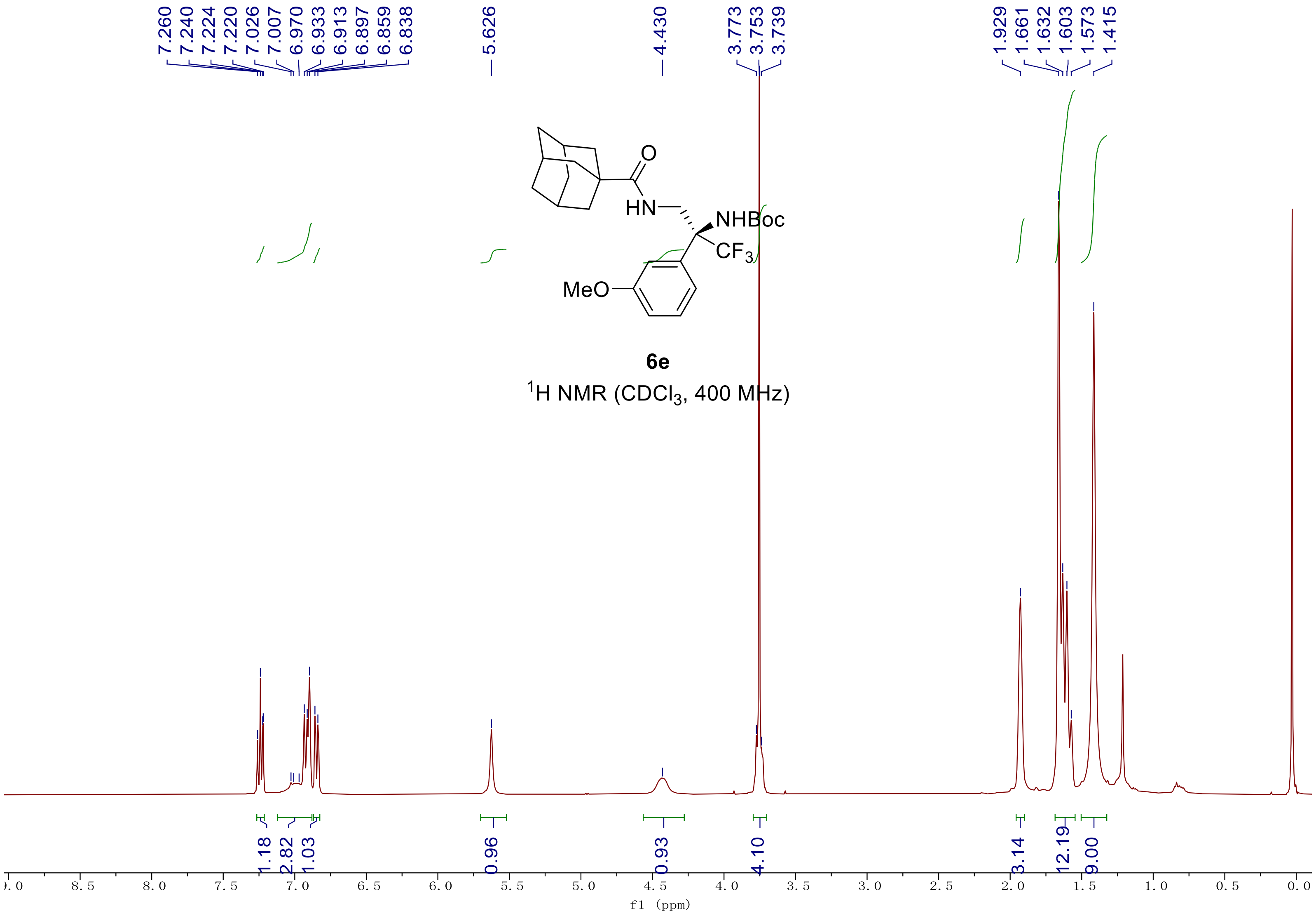
7.260
7.240
7.224
7.220
7.026
7.007
6.970
6.933
6.913
6.897
6.859
6.838
5.626
4.430
3.773
3.753
3.739
1.929
1.661
1.632
1.603
1.573
1.415
O
HN
NHBoc
CF3
MeO
6e
1H NMR (CDCl3, 400 MHz)
1.18
2.82
1.03
0.96
0.93
4.10
3.14
12.19
9.00
f1 (ppm)

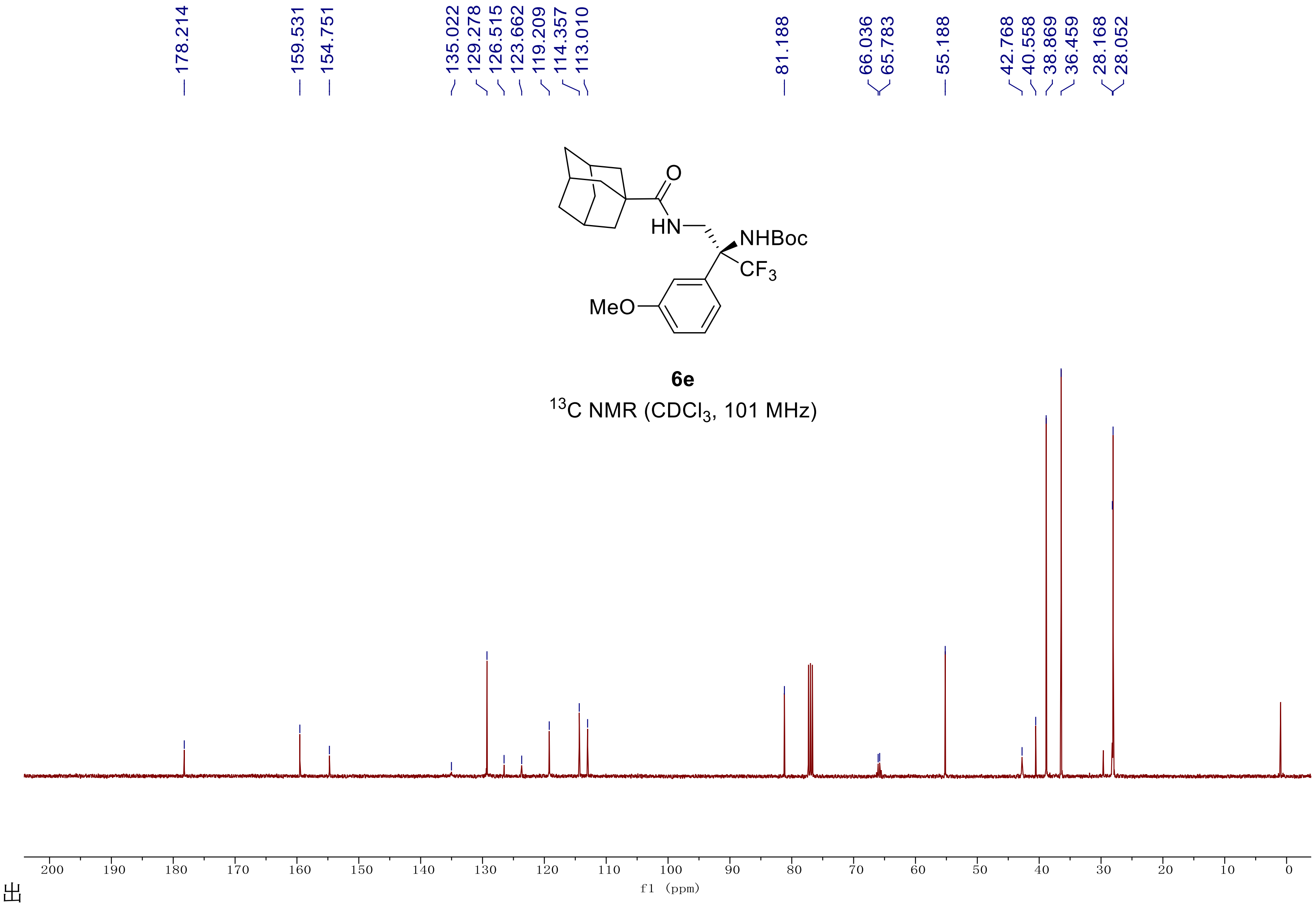

6e

^{13}C NMR ($CDCl_3$, 101 MHz)

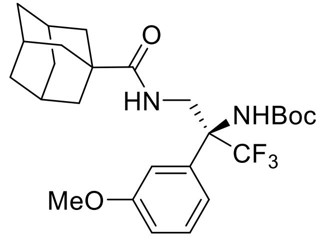

6e

^{19}F NMR ($CDCl_3$, 376 MHz)

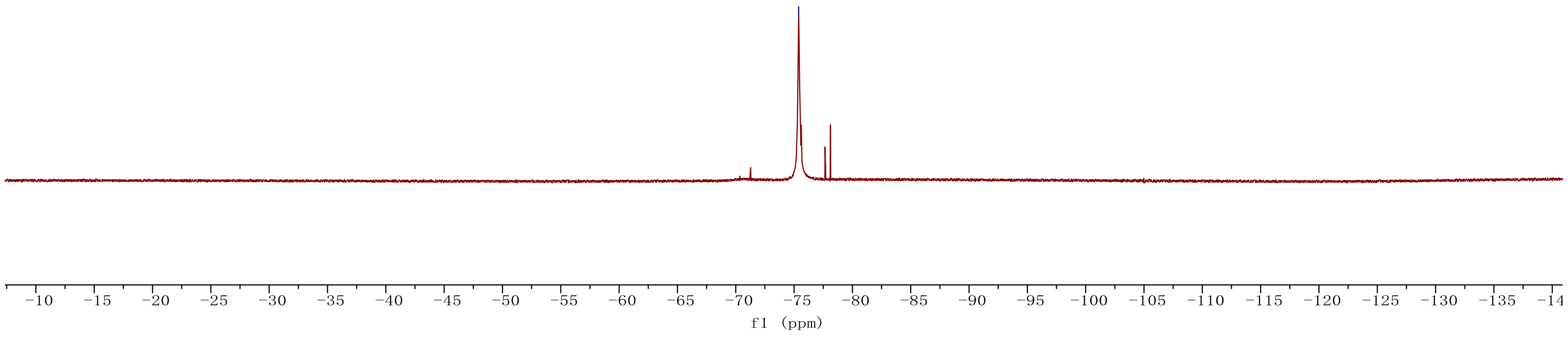

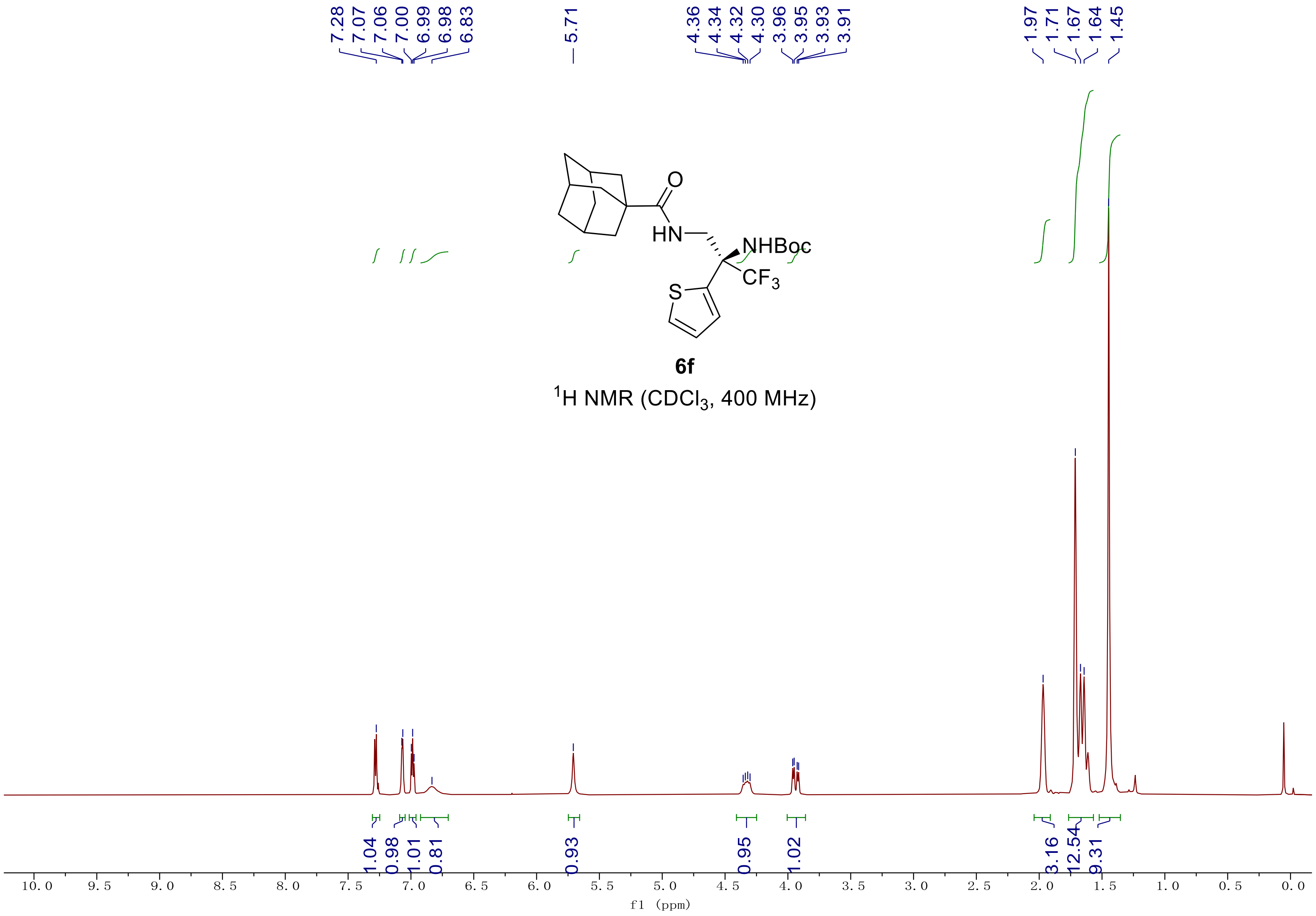

6f

^{1}H NMR ($CDCl_3$, 400 MHz)

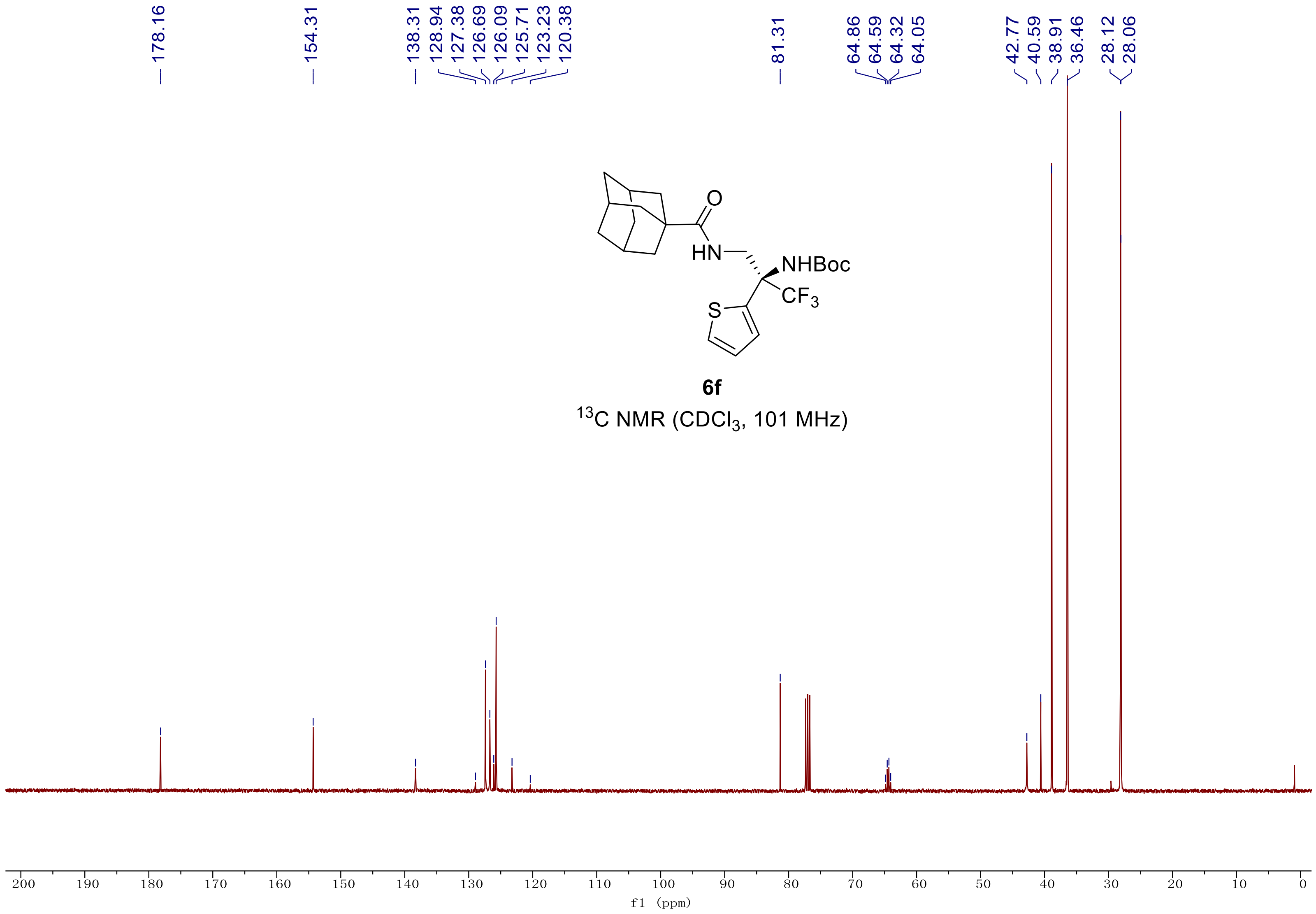

6f

^{13}C NMR ($CDCl_3$, 101 MHz)

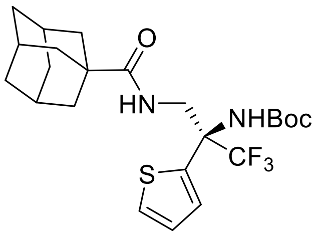

6f

^{19}F NMR ($CDCl_3$, 376 MHz)

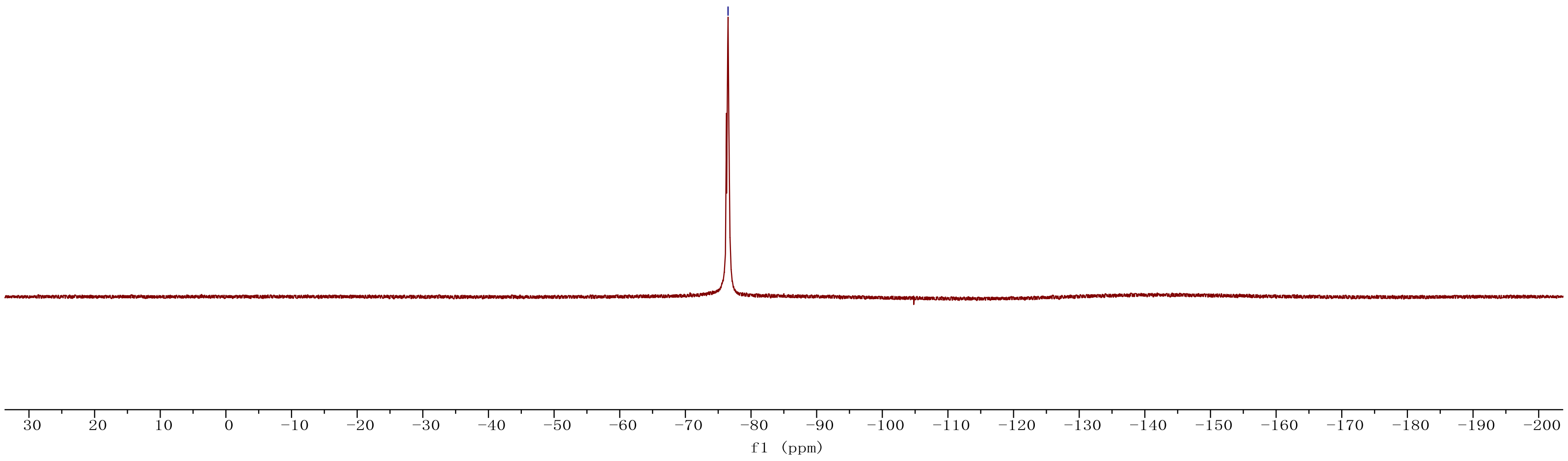

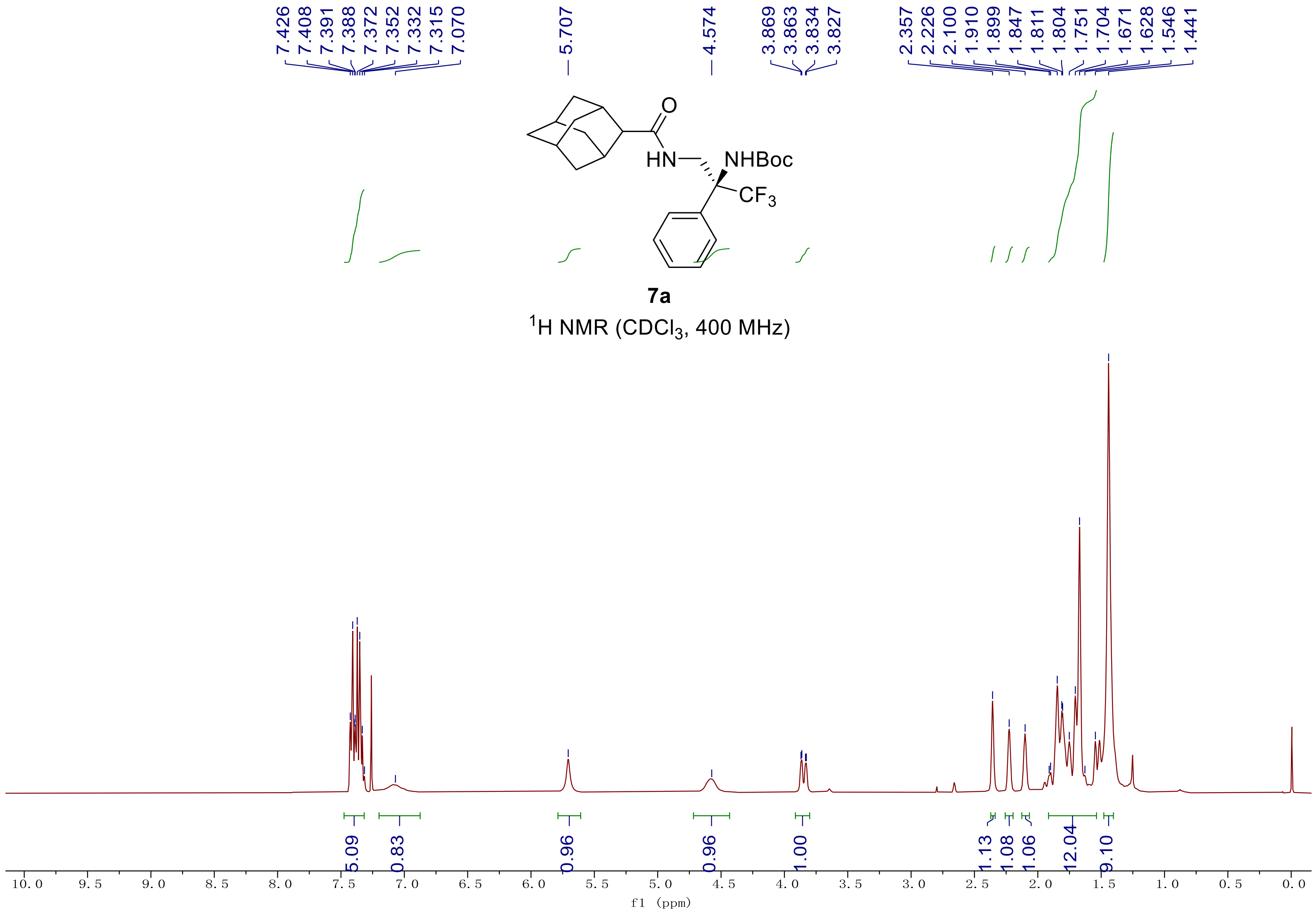

7a

^{1}H NMR ($CDCl_3$, 400 MHz)

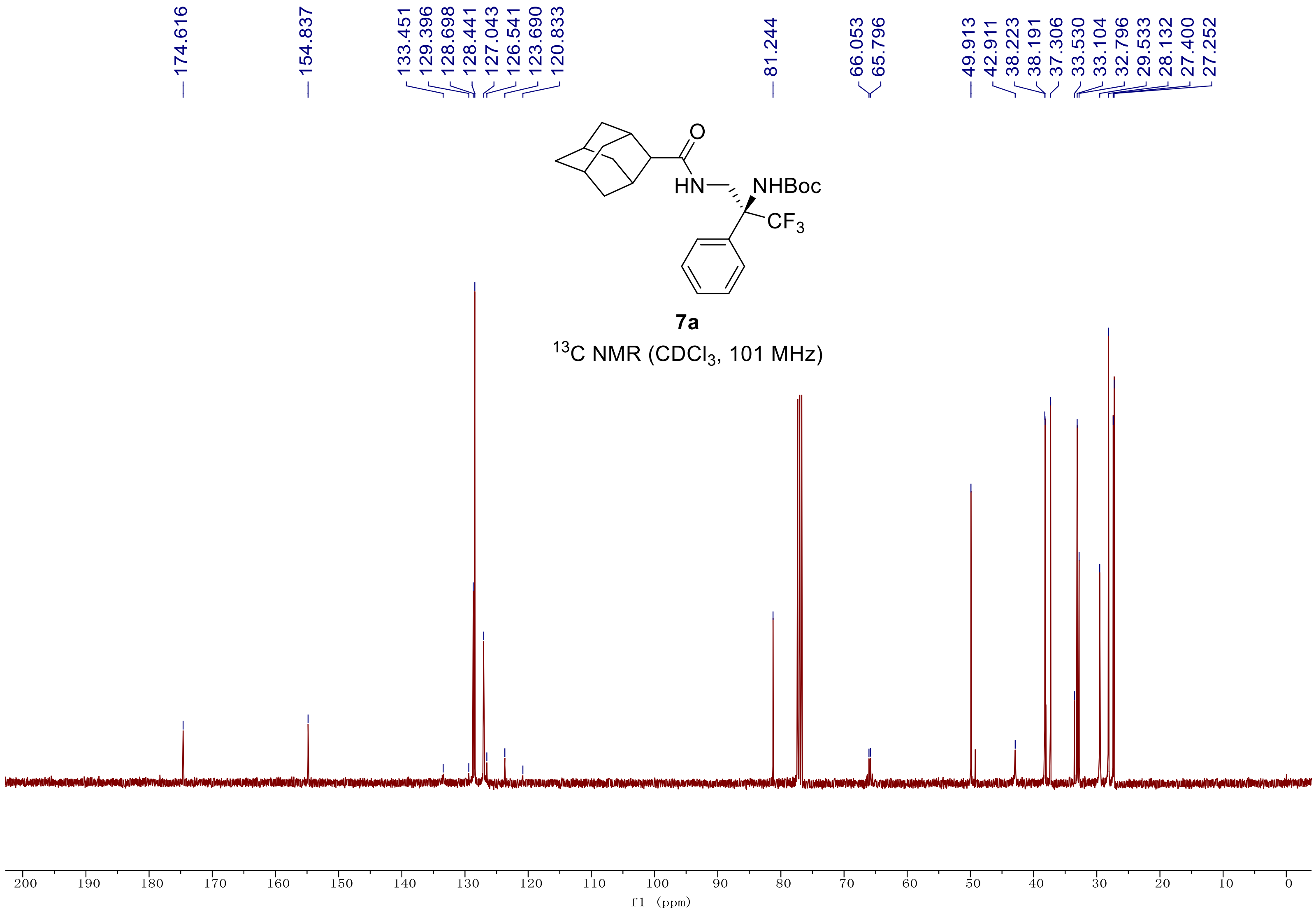
174.616
154.837
133.451
129.396
128.698
128.441
127.043
126.541
123.690
120.833
81.244
66.053
65.796
49.913
42.911
38.223
38.191
37.306
33.530
33.104
32.796
29.533
28.132
27.400
27.252
O
HN
NHBoc
CF_3
7a
^{13}C NMR ($CDCl_3$, 101 MHz)
200 190 180 170 160 150 140 130 120 110 100 90 80 70 60 50 40 30 20 10 0
f1 (ppm)

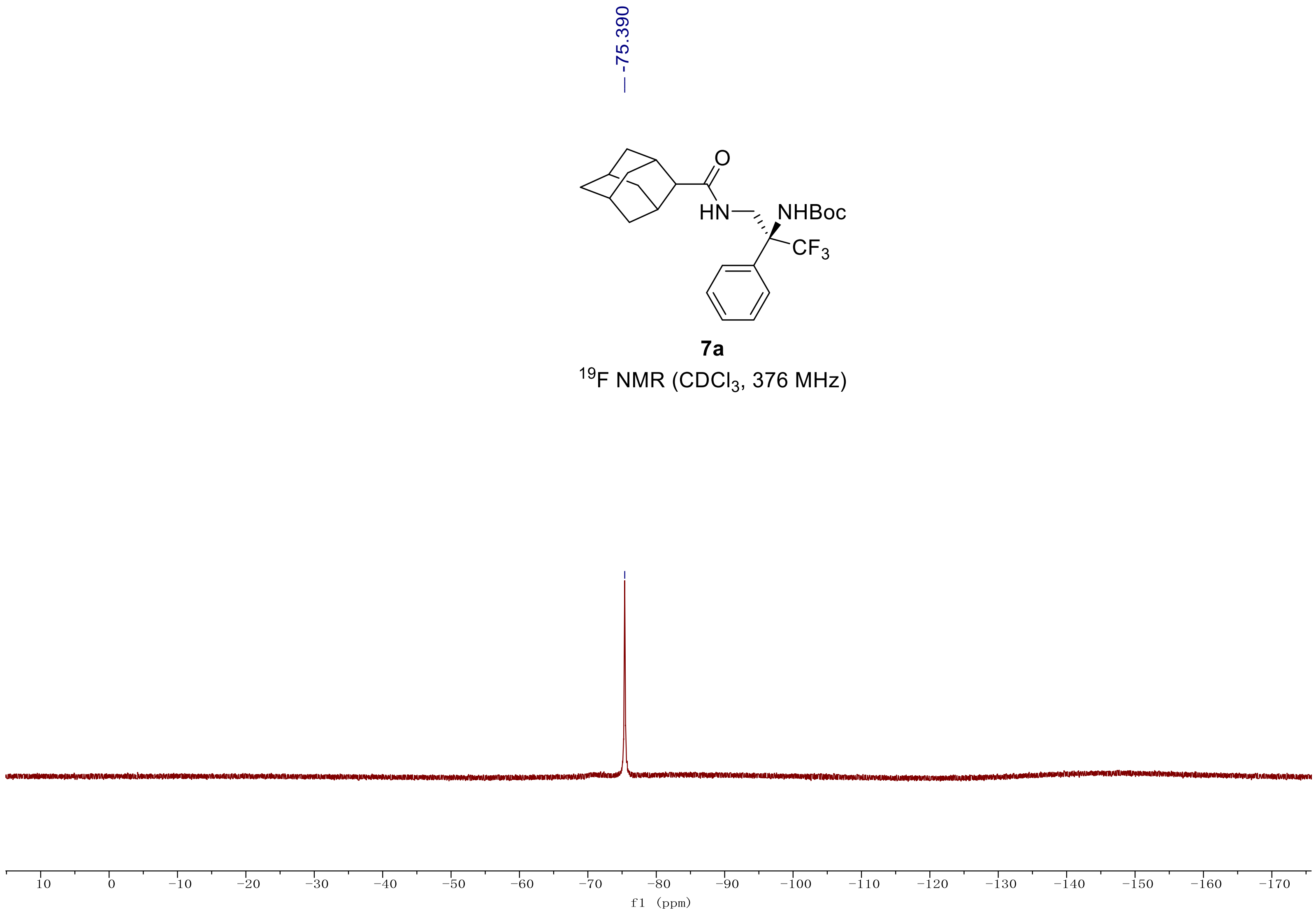

7a

^{19}F NMR ($CDCl_3$, 376 MHz)

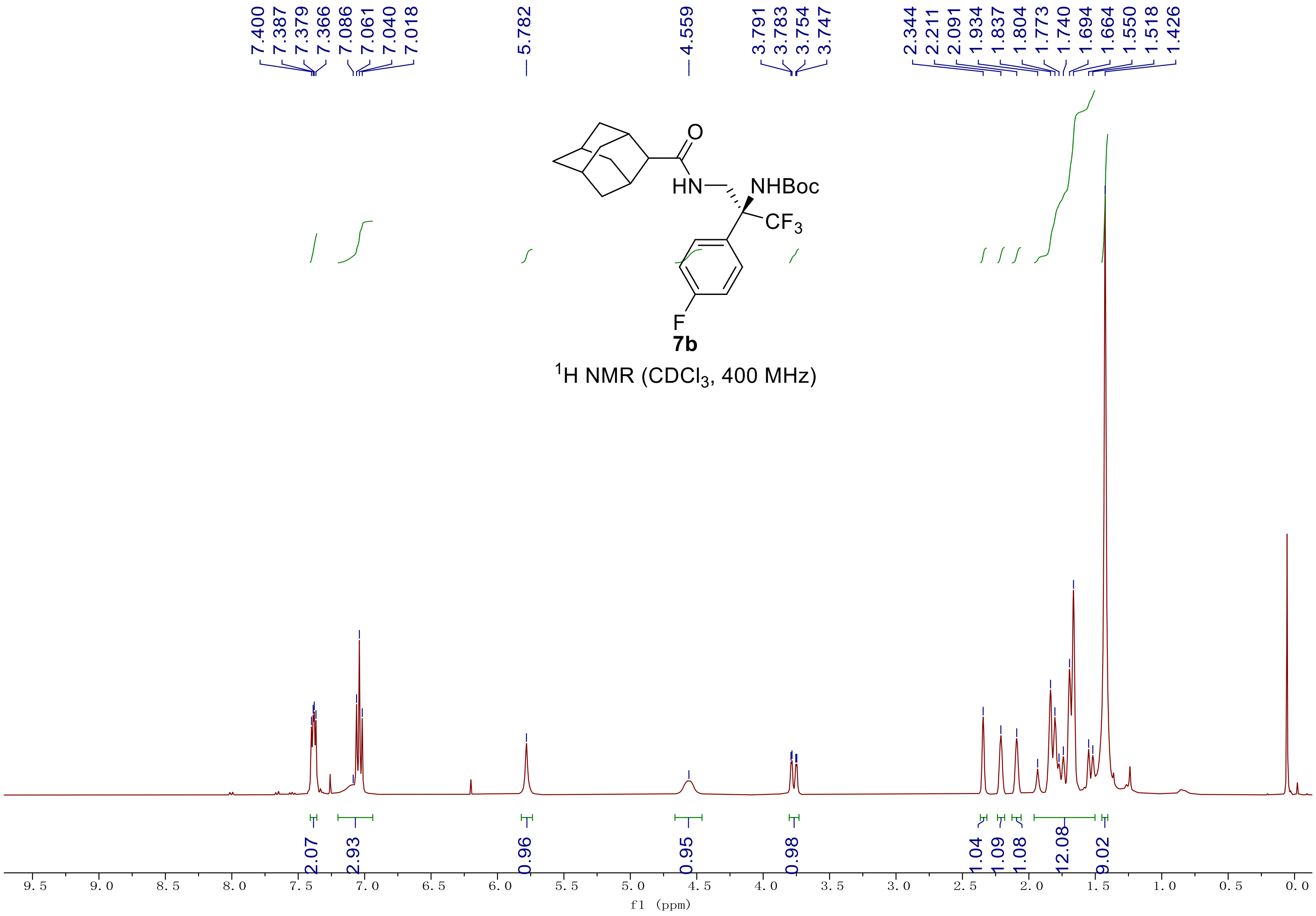
7.400
7.387
7.379
7.366
7.086
7.061
7.040
7.018
5.782
4.559
3.791
3.783
3.754
3.747
2.344
2.211
2.091
1.934
1.837
1.804
1.773
1.740
1.694
1.664
1.550
1.518
1.426
O
HN
NHBoc
CF3
F
7b
1H NMR (CDCl3, 400 MHz)
2.07
2.93
0.96
0.95
0.98
1.04
1.09
1.08
12.08
9.02
9.5 9.0 8.5 8.0 7.5 7.0 6.5 6.0 5.5 5.0 4.5 4.0 3.5 3.0 2.5 2.0 1.5 1.0 0.5 0.0
f1 (ppm)

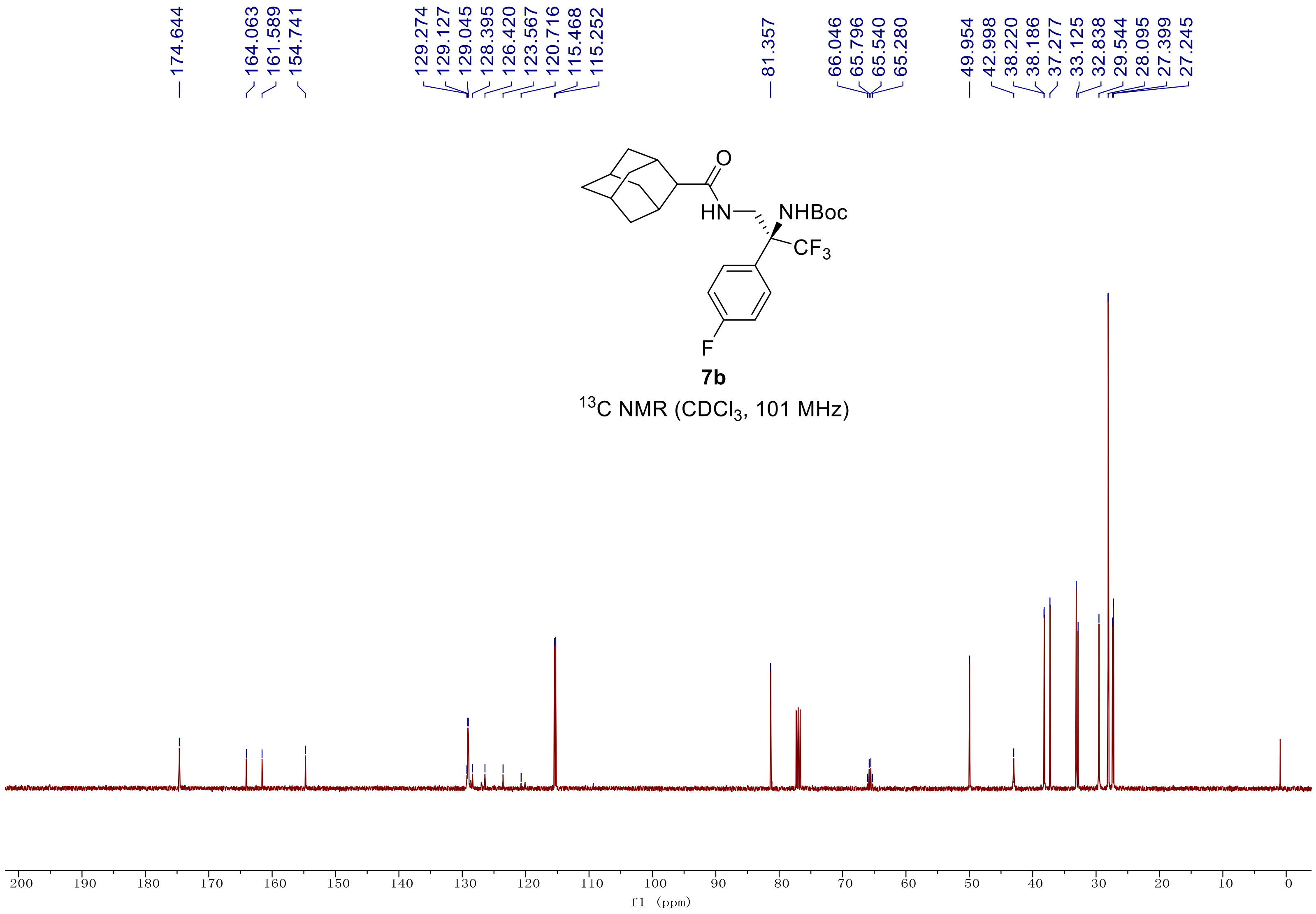

7b

^{13}C NMR ($CDCl_3$, 101 MHz)

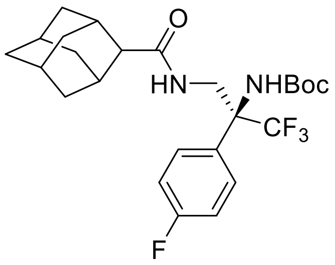

7b

^{19}F NMR (CDCl$_3$, 376 MHz)

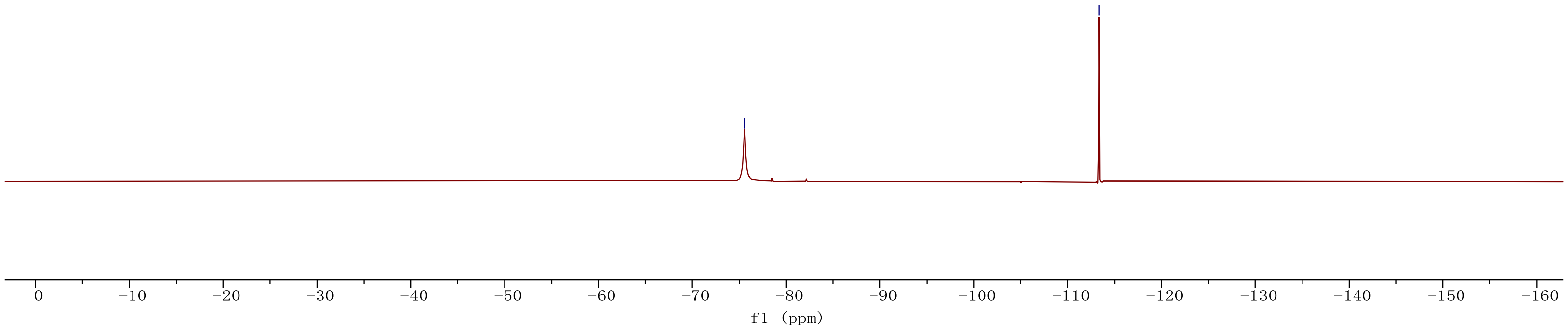

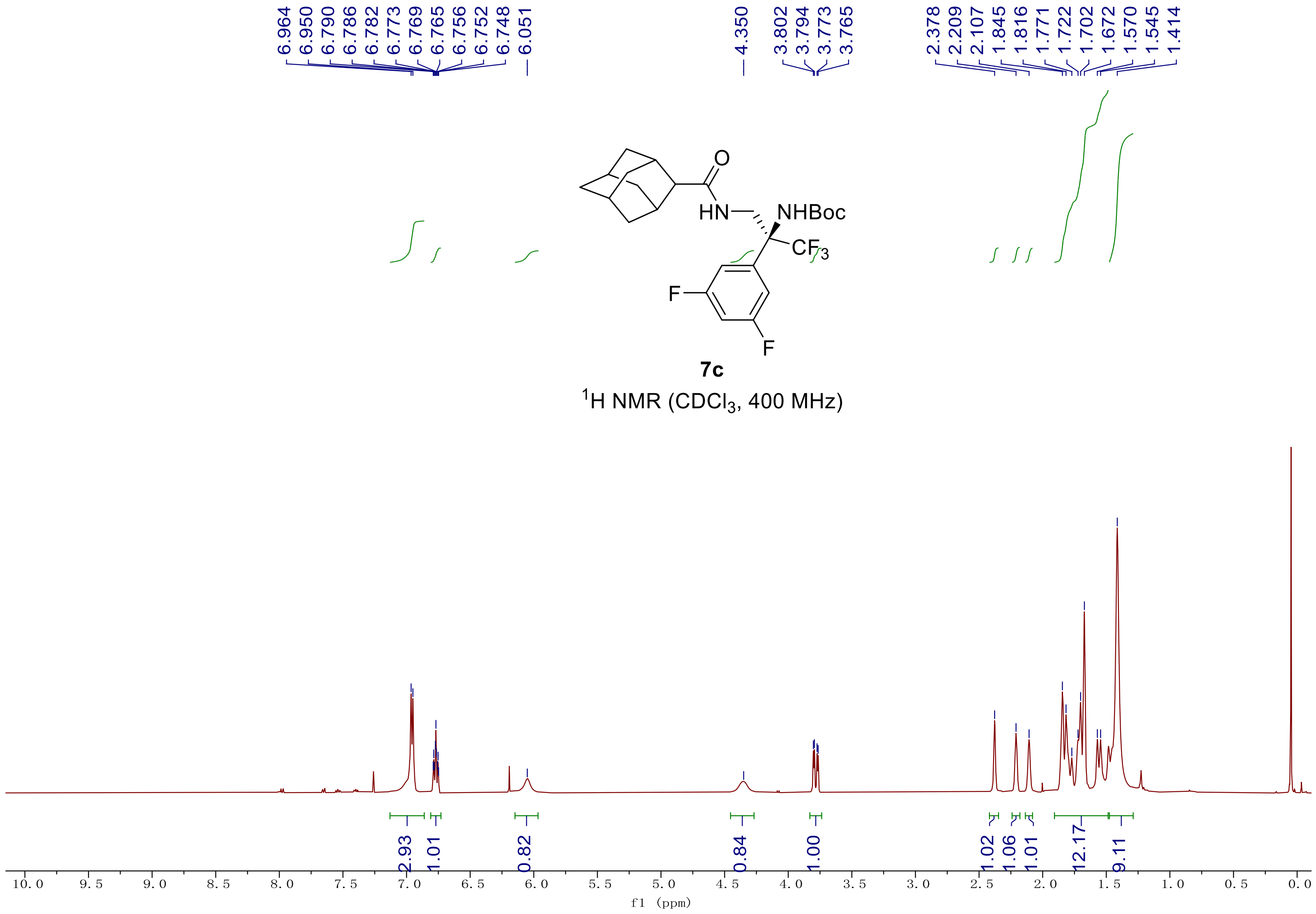

7c

^{1}H NMR ($CDCl_3$, 400 MHz)

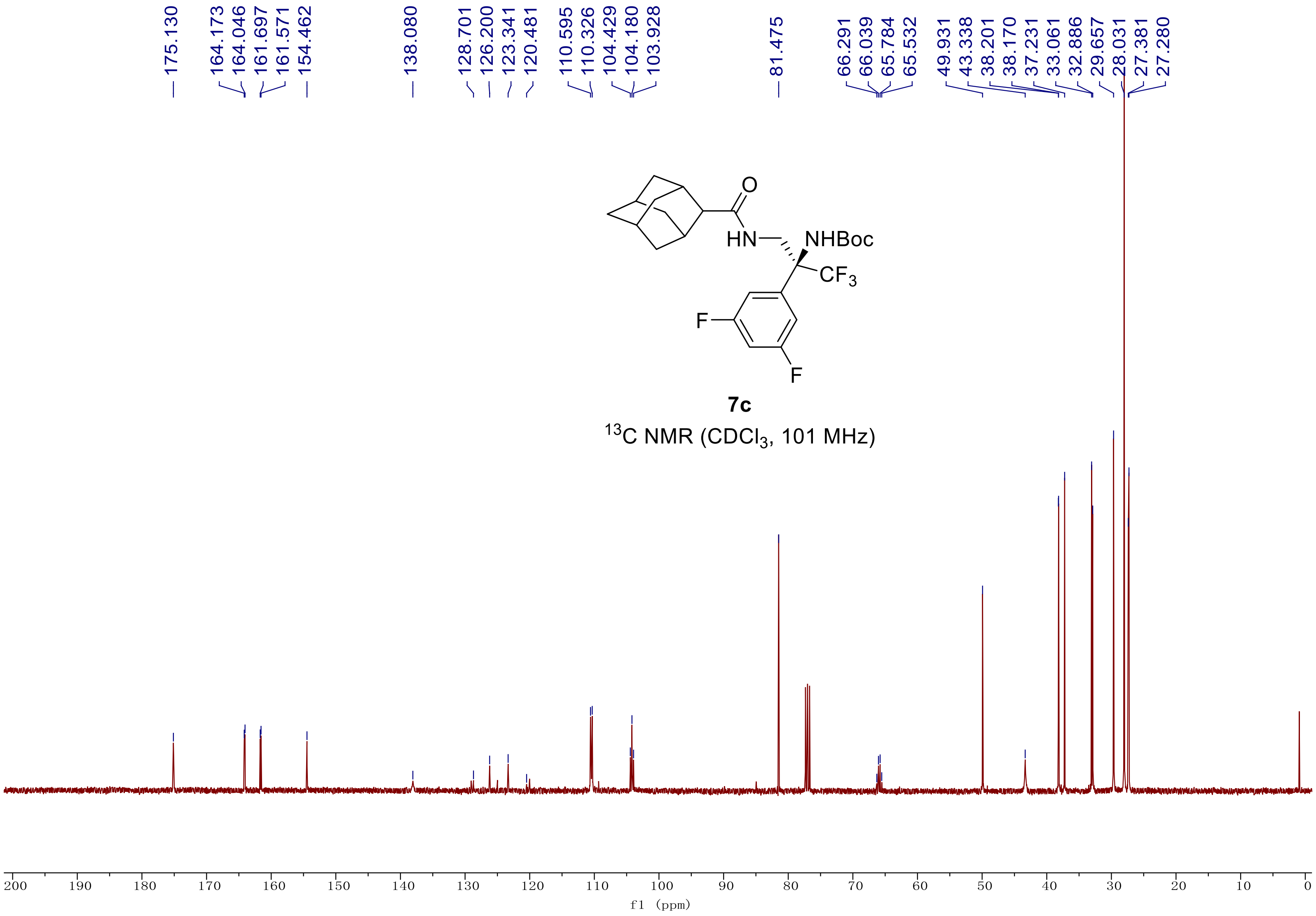

7c

^{13}C NMR ($CDCl_3$, 101 MHz)

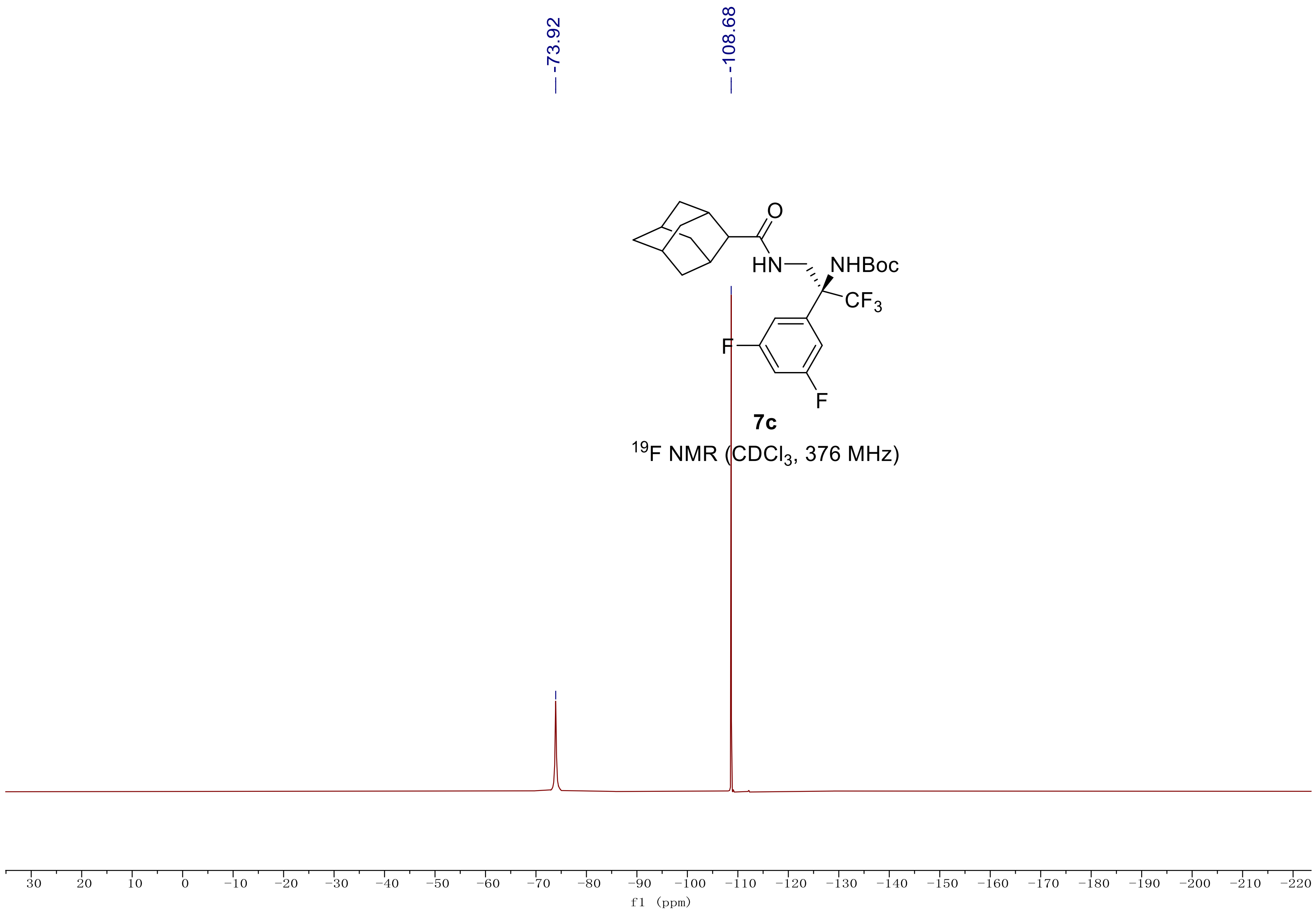
-73.92
-108.68
O
HN
NHBoc
CF3
F
F
7c
19F NMR (CDCl3, 376 MHz)
30 20 10 0 −10 −20 −30 −40 −50 −60 −70 −80 −90 −100 −110 −120 −130 −140 −150 −160 −170 −180 −190 −200 −210 −220
f1 (ppm)

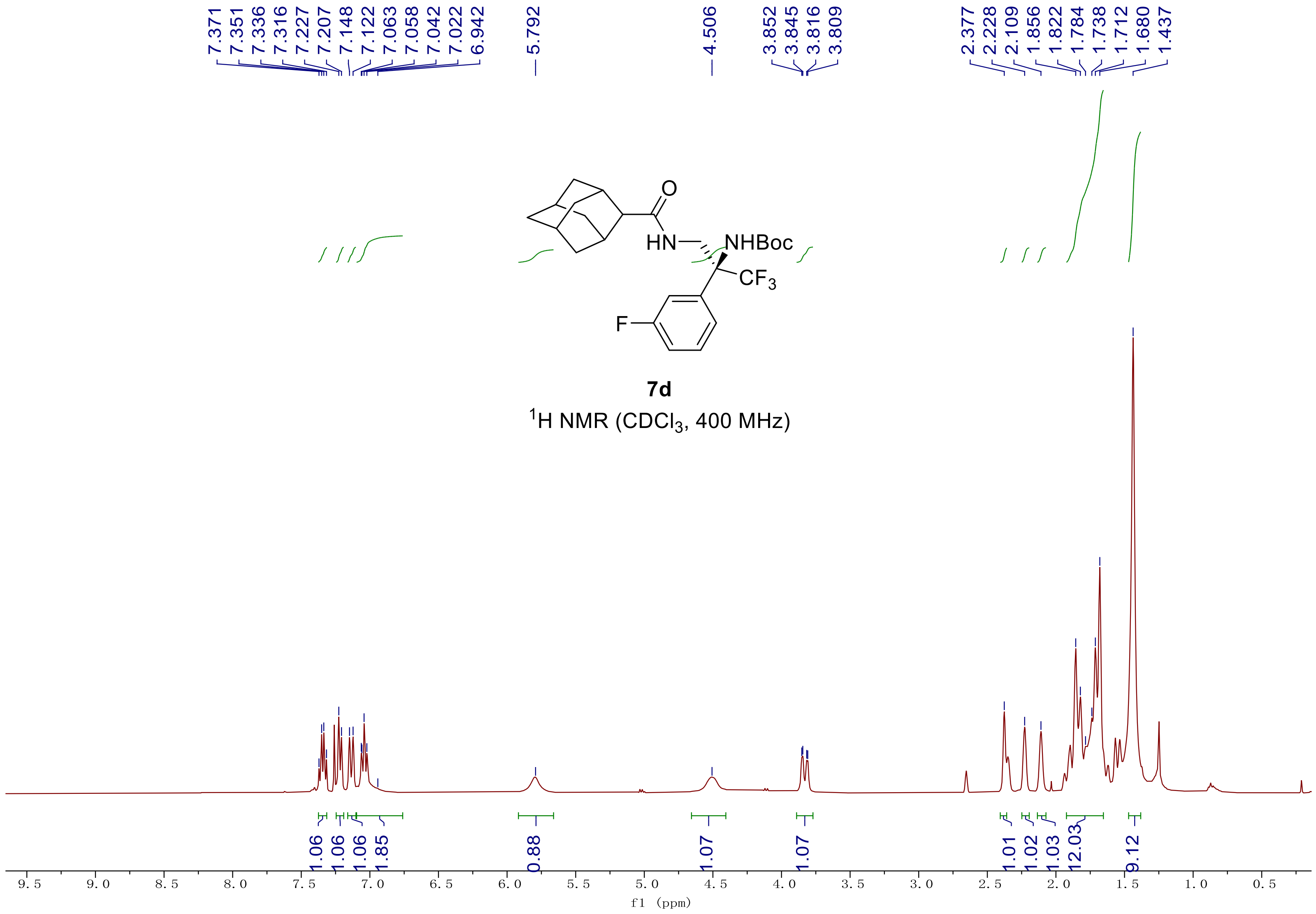
7.371 7.351 7.336 7.316 7.227 7.207 7.148 7.122 7.063 7.058 7.042 7.022 6.942
5.792
4.506
3.852 3.845 3.816 3.809
2.377 2.228 2.109 1.856 1.822 1.784 1.738 1.712 1.680 1.437
O
HN
NHBoc
CF3
F
7d
1H NMR (CDCl3, 400 MHz)
1.06 1.06 1.06 1.85 0.88 1.07 1.07 1.01 1.02 1.03 12.03 9.12
9.5 9.0 8.5 8.0 7.5 7.0 6.5 6.0 5.5 5.0 4.5 4.0 3.5 3.0 2.5 2.0 1.5 1.0 0.5
f1 (ppm)

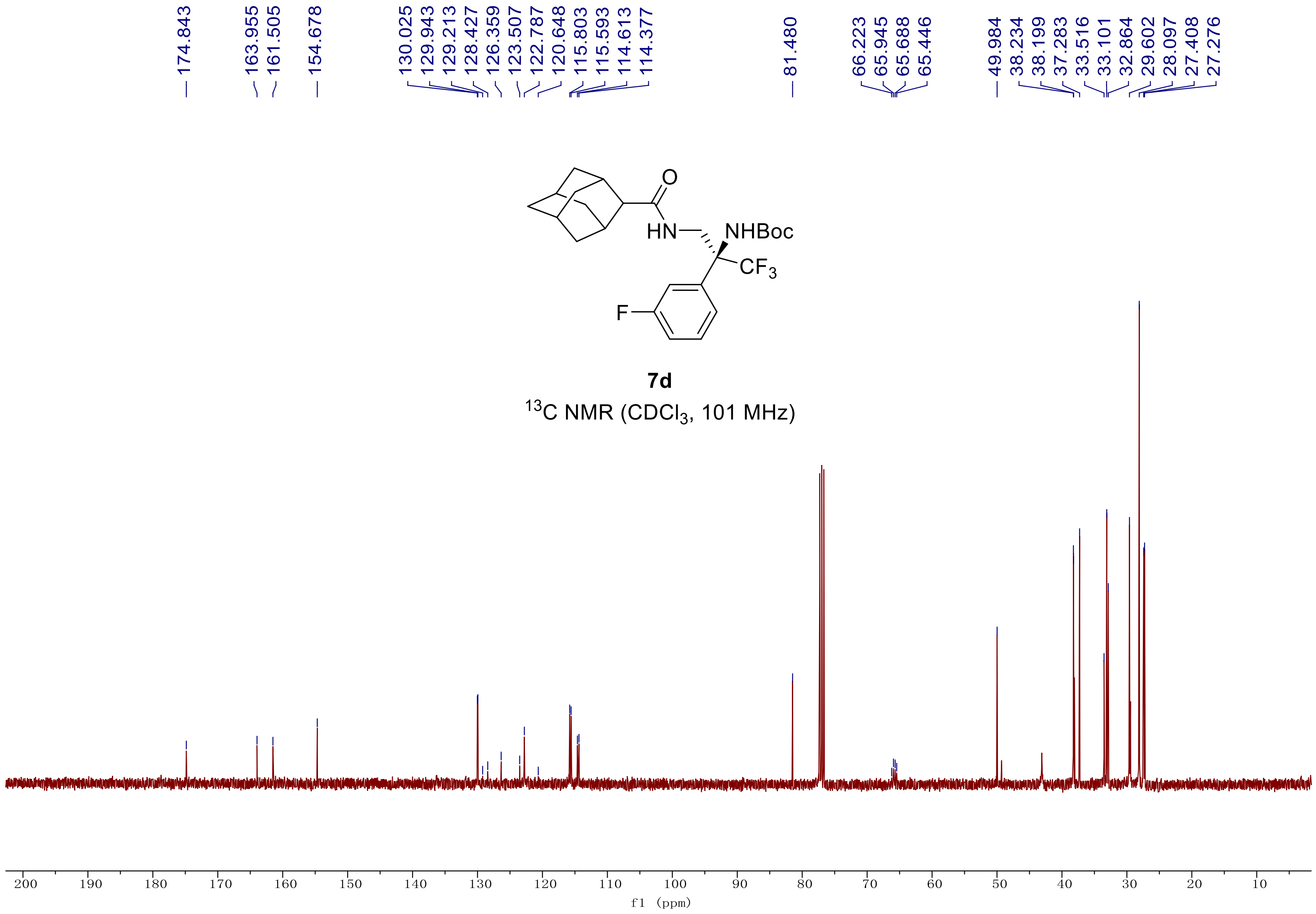

7d

^{13}C NMR ($CDCl_3$, 101 MHz)

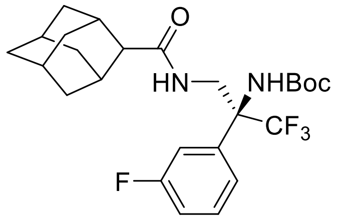

7d

^{19}F NMR ($CDCl_3$, 376 MHz)

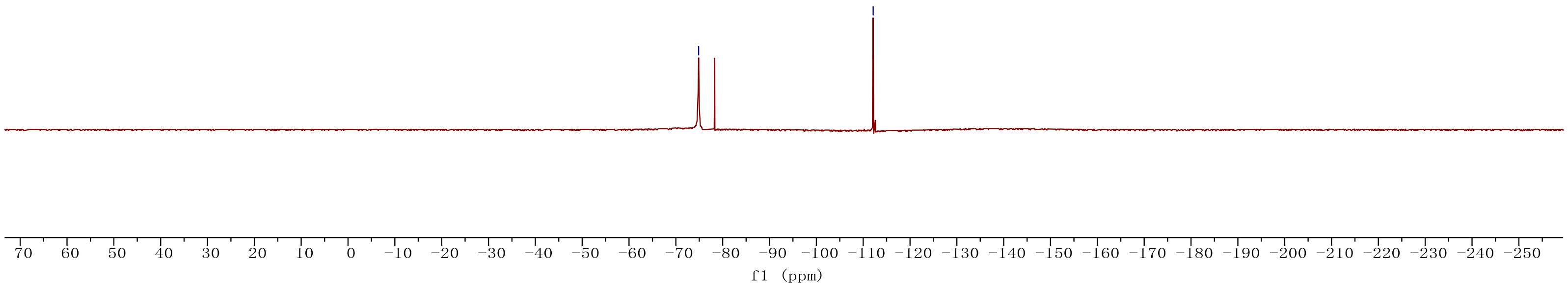

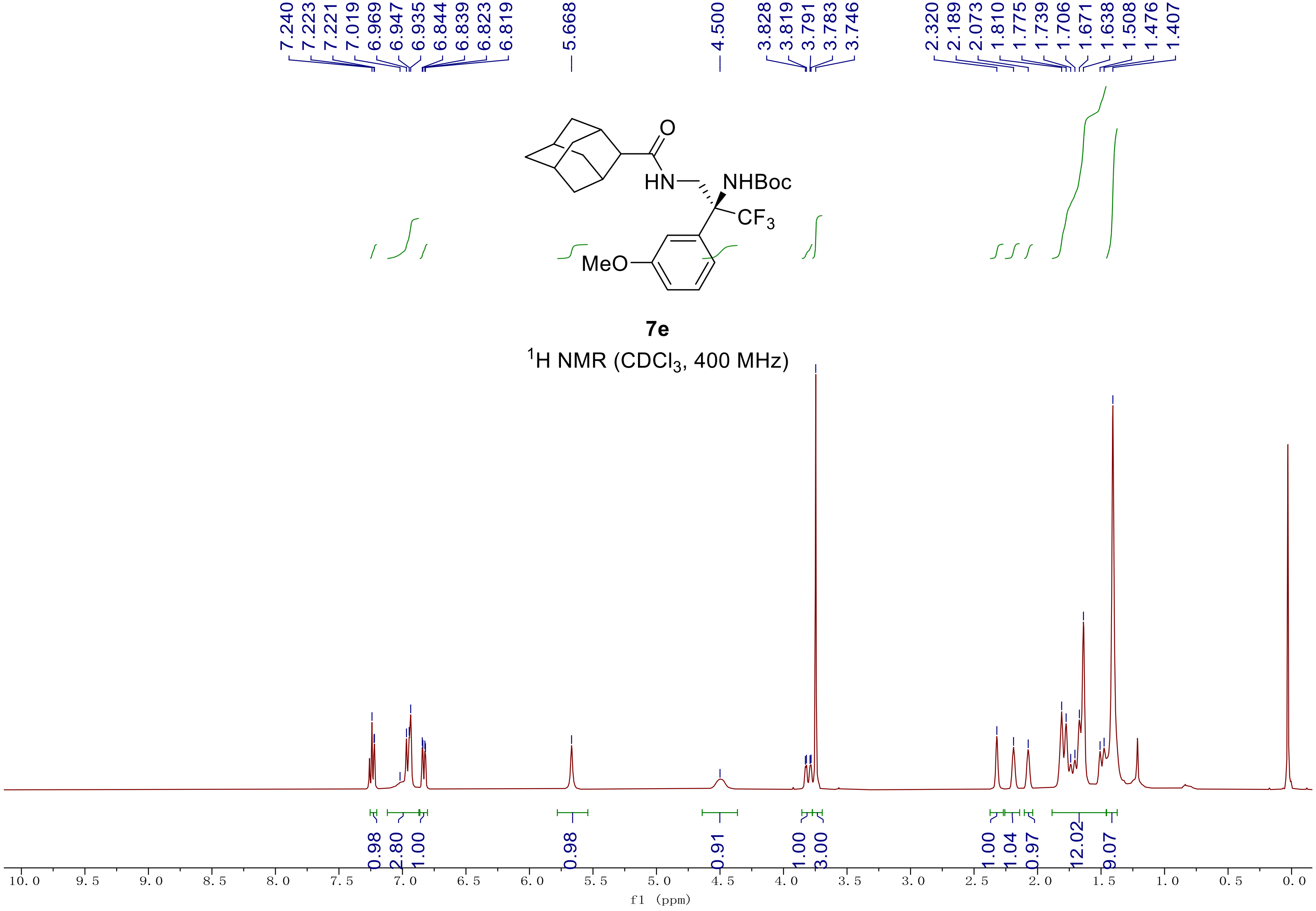

7.240
7.223
7.221
7.019
6.969
6.947
6.935
6.844
6.839
6.823
6.819
5.668
4.500
3.828
3.819
3.791
3.783
3.746
2.320
2.189
2.073
1.810
1.775
1.739
1.706
1.671
1.638
1.508
1.476
1.407
NHBoc
HN
O
CF3
MeO
7e
1H NMR (CDCl3, 400 MHz)
0.98
2.80
1.00
0.98
0.91
1.00
3.00
1.00
1.04
0.97
12.02
9.07
10.0
9.5
9.0
8.5
8.0
7.5
7.0
6.5
6.0
5.5
5.0
4.5
4.0
3.5
3.0
2.5
2.0
1.5
1.0
0.5
0.0
f1 (ppm)

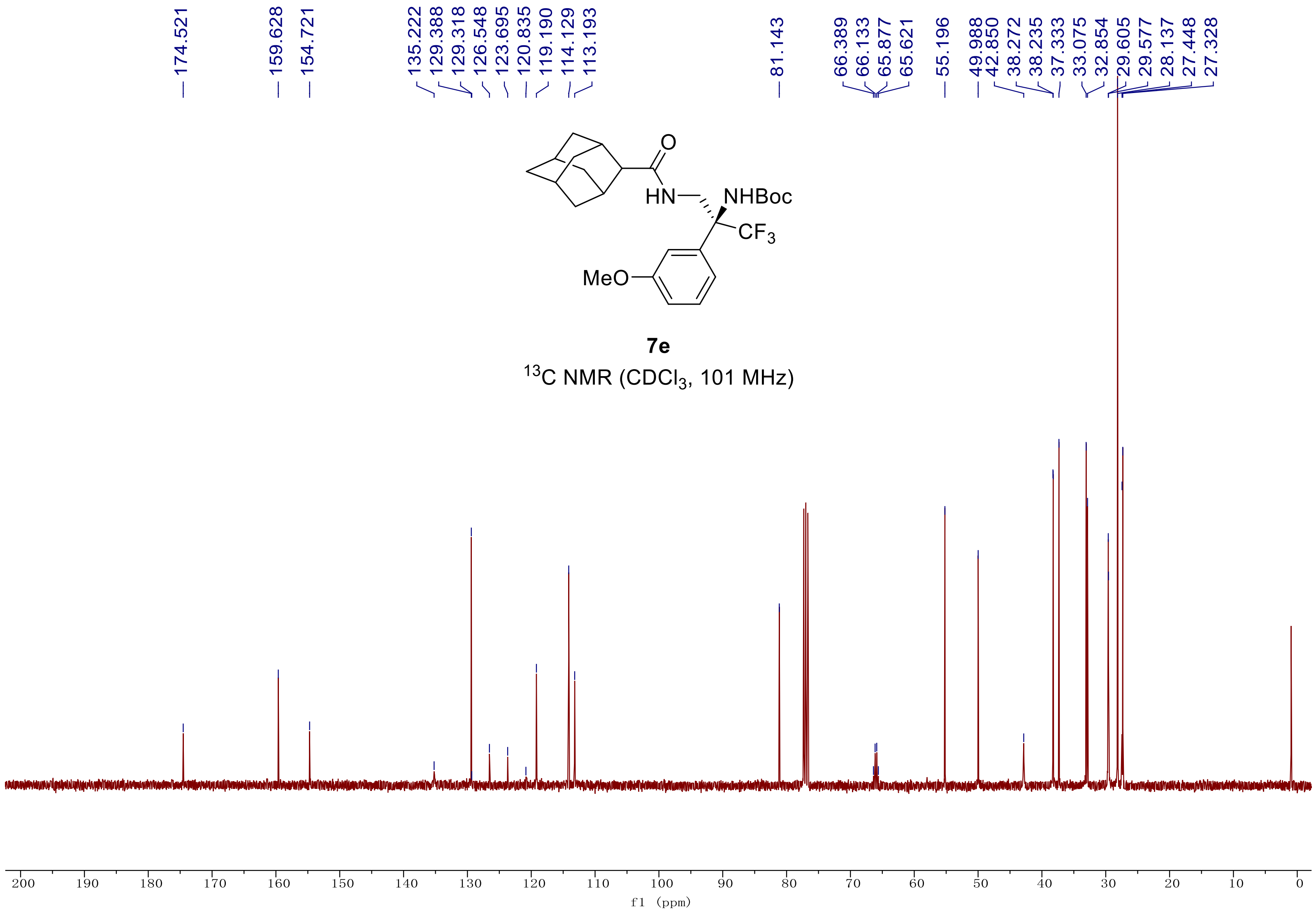

7e

^{13}C NMR ($CDCl_3$, 101 MHz)

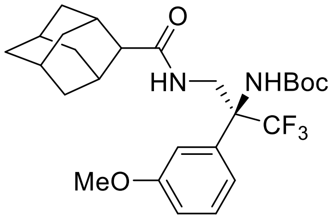

7e

^{19}F NMR ($CDCl_3$, 376 MHz)

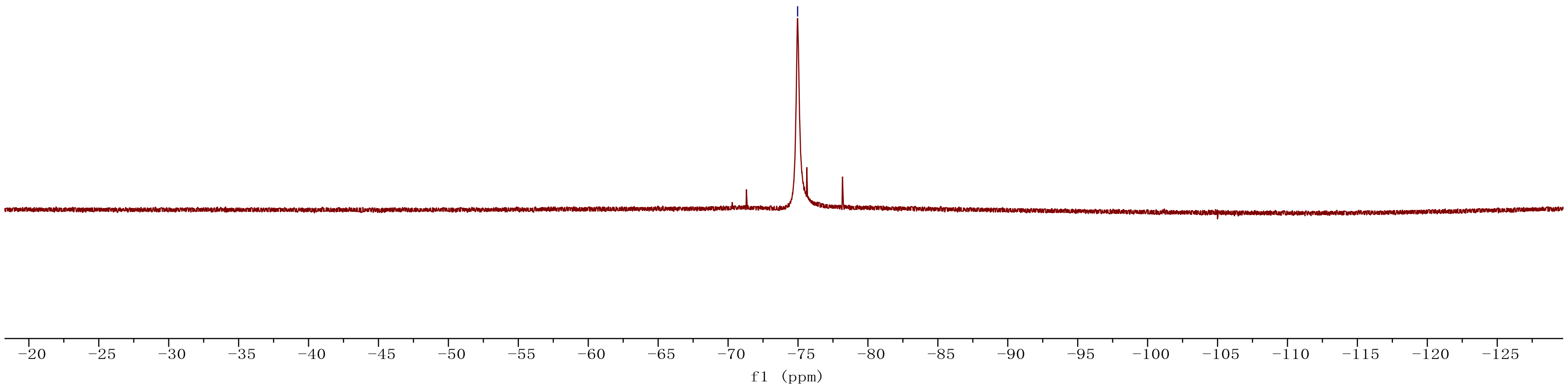

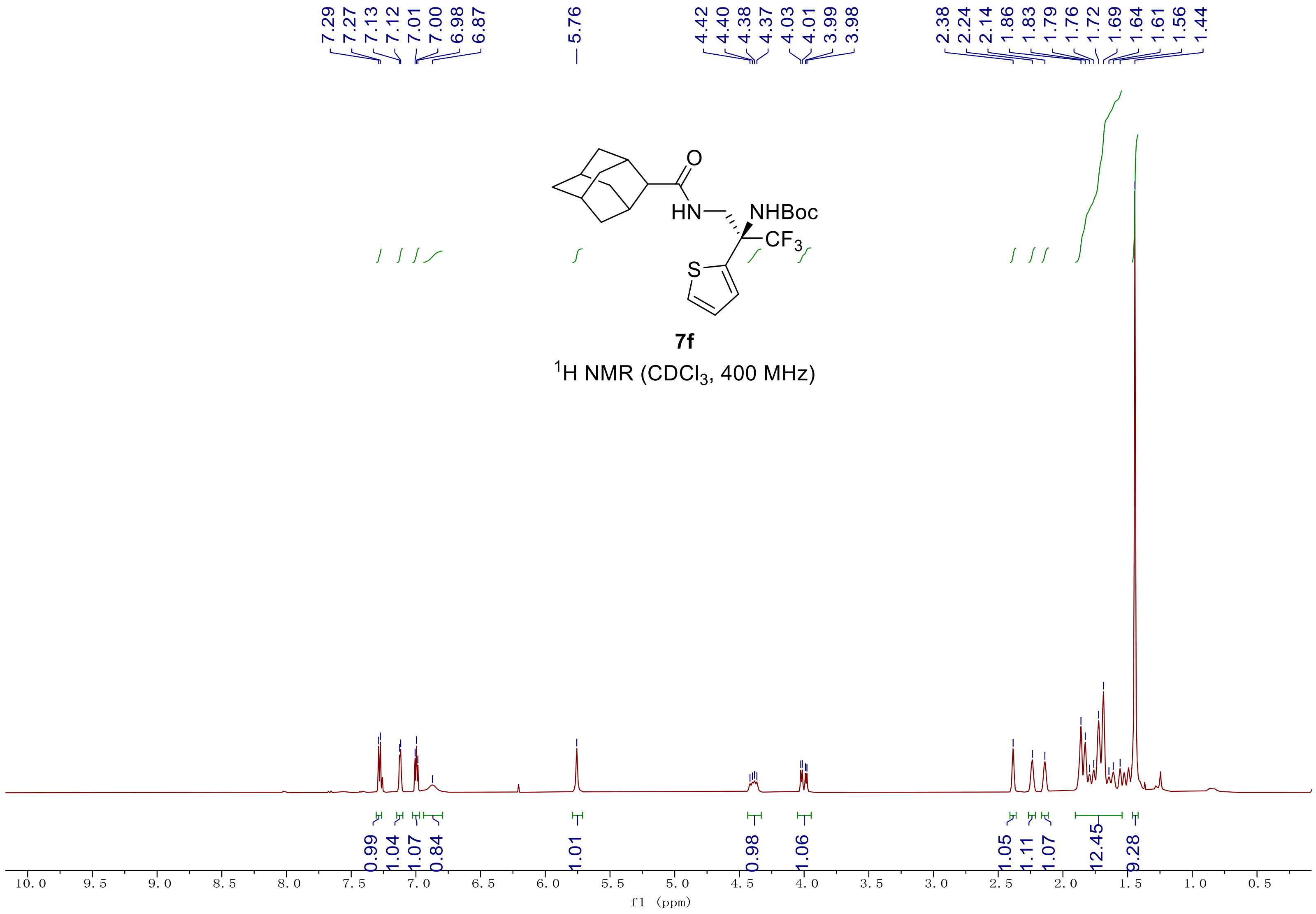

7f

^{1}H NMR ($CDCl_3$, 400 MHz)

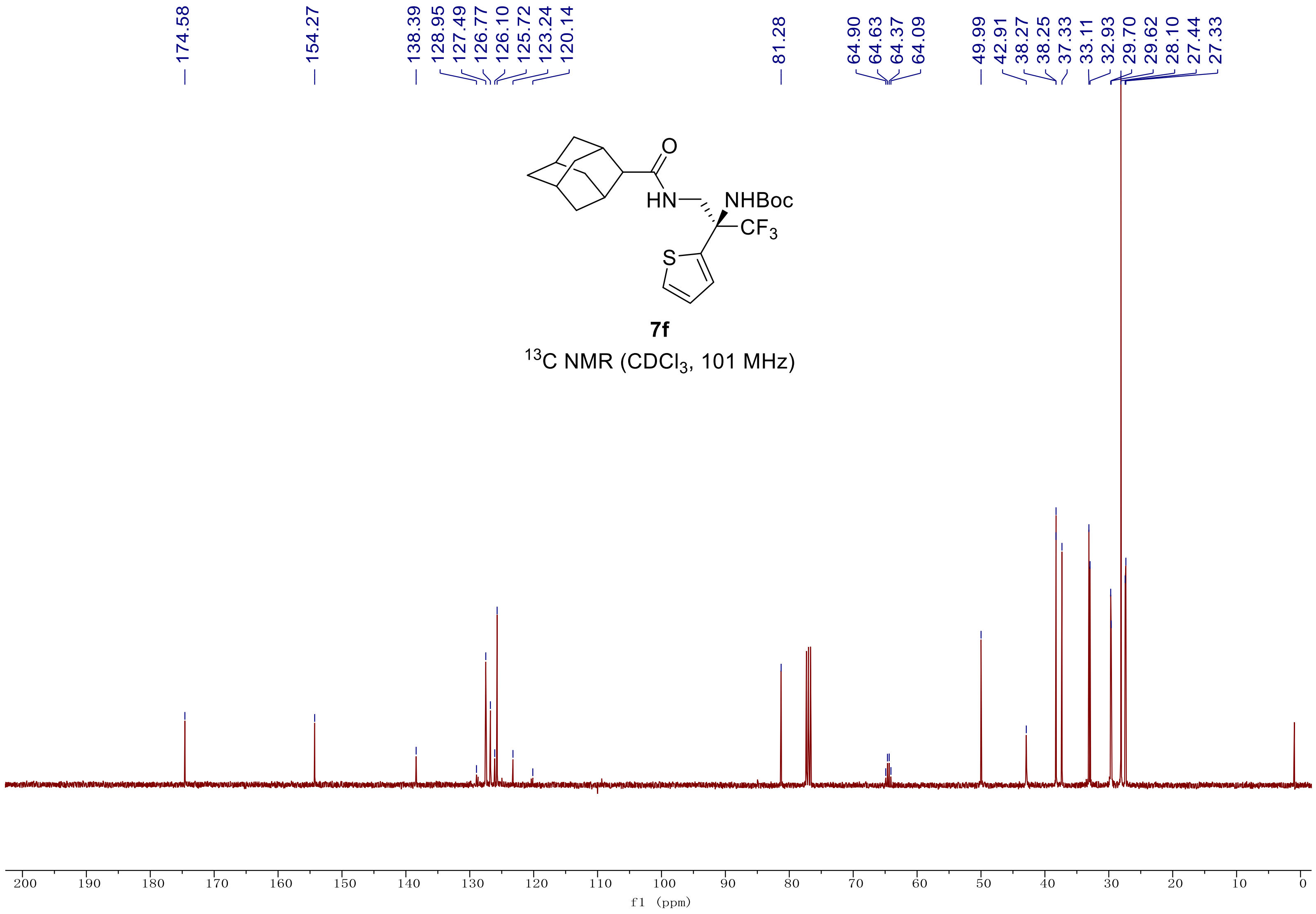
174.58
154.27
138.39
128.95
127.49
126.77
126.10
125.72
123.24
120.14
81.28
64.90
64.63
64.37
64.09
49.99
42.91
38.27
38.25
37.33
33.11
32.93
29.70
29.62
28.10
27.44
27.33
O
HN
NHBoc
CF_3
S
7f
^{13}C NMR ($CDCl_3$, 101 MHz)
200 190 180 170 160 150 140 130 120 110 100 90 80 70 60 50 40 30 20 10 0
f1 (ppm)

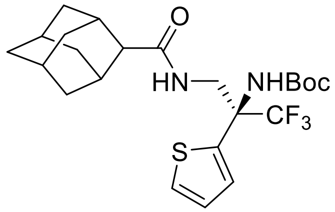

7f

^{19}F NMR ($CDCl_3$, 376 MHz)

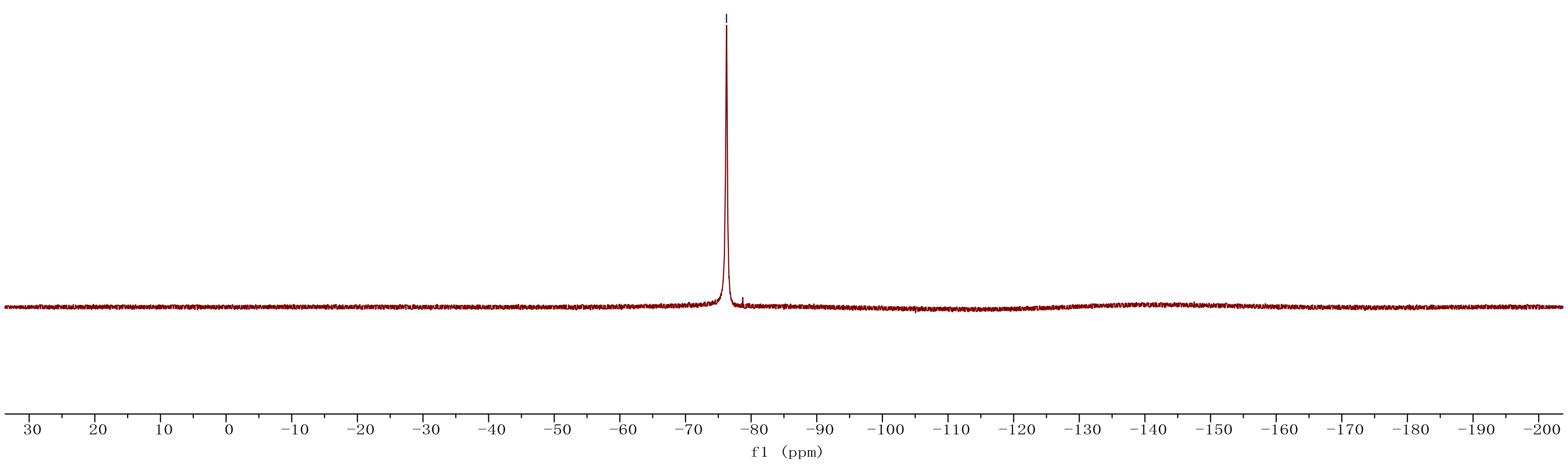

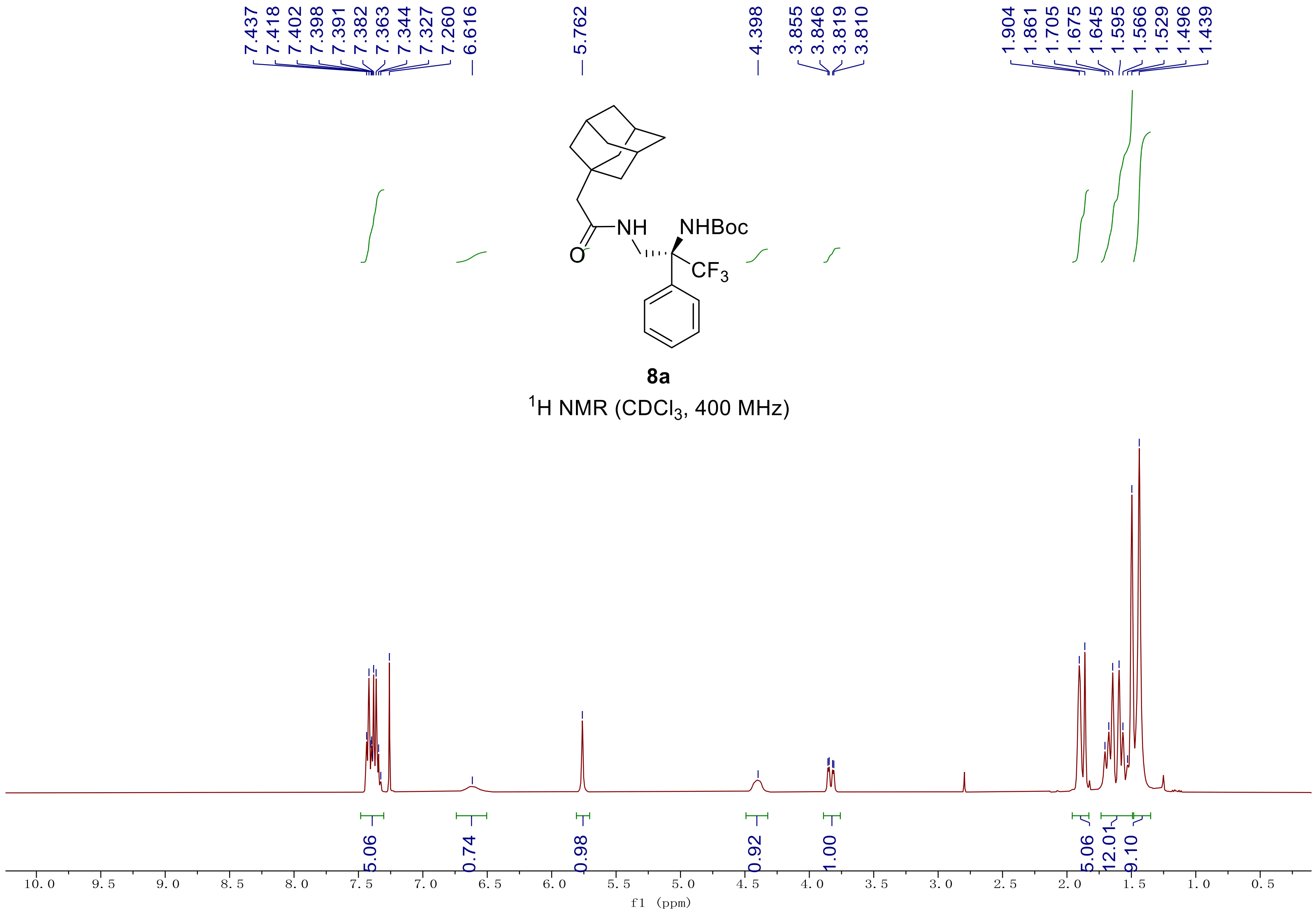

8a

^{1}H NMR ($CDCl_3$, 400 MHz)

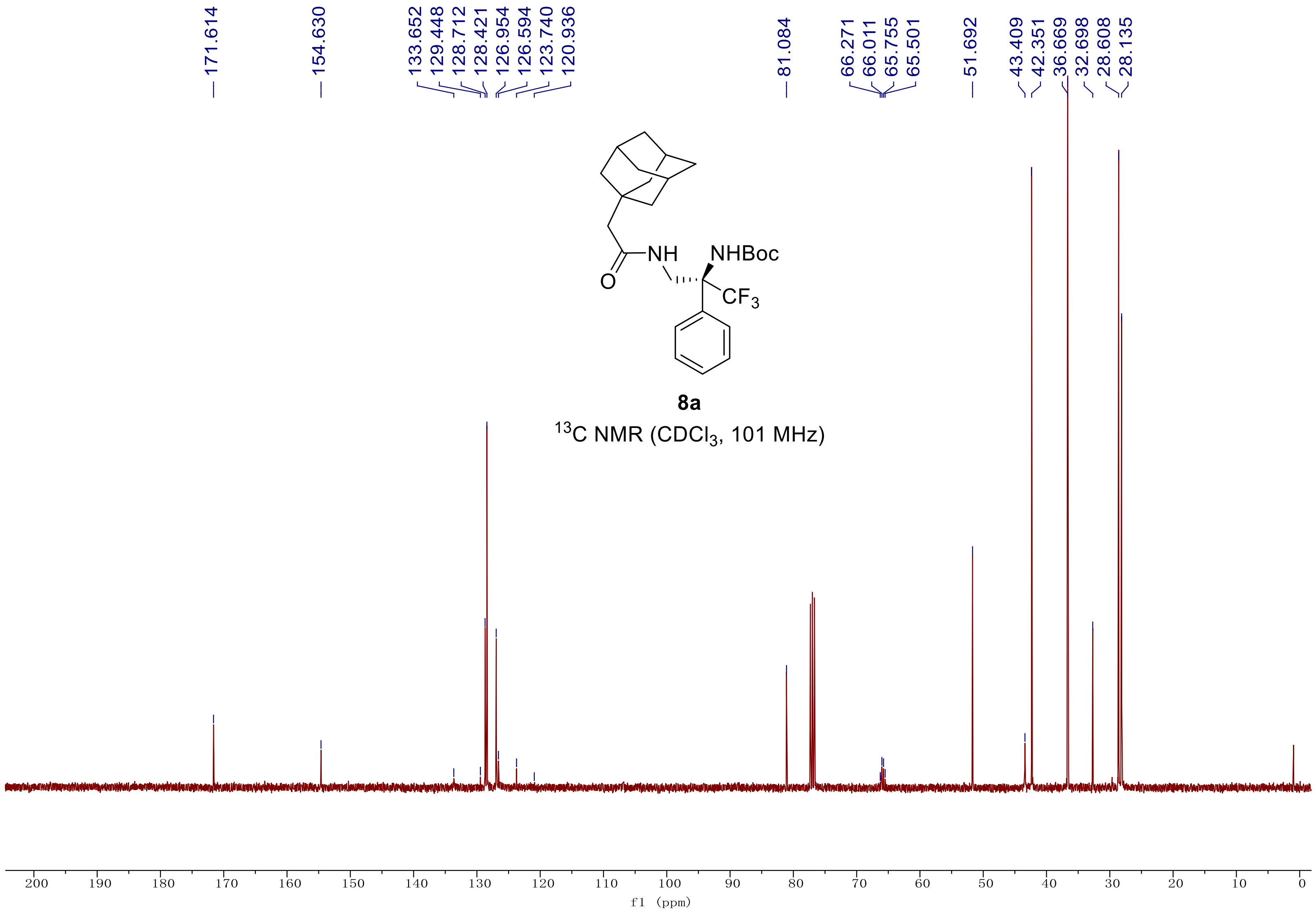

171.614
154.630
133.652
129.448
128.712
128.421
126.954
126.594
123.740
120.936
81.084
66.271
66.011
65.755
65.501
51.692
43.409
42.351
36.669
32.698
28.608
28.135
NHBoc
NH
O
CF3
8a
13C NMR (CDCl3, 101 MHz)
f1 (ppm)

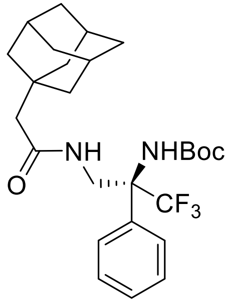

8a

^{19}F NMR ($CDCl_3$, 376 MHz)

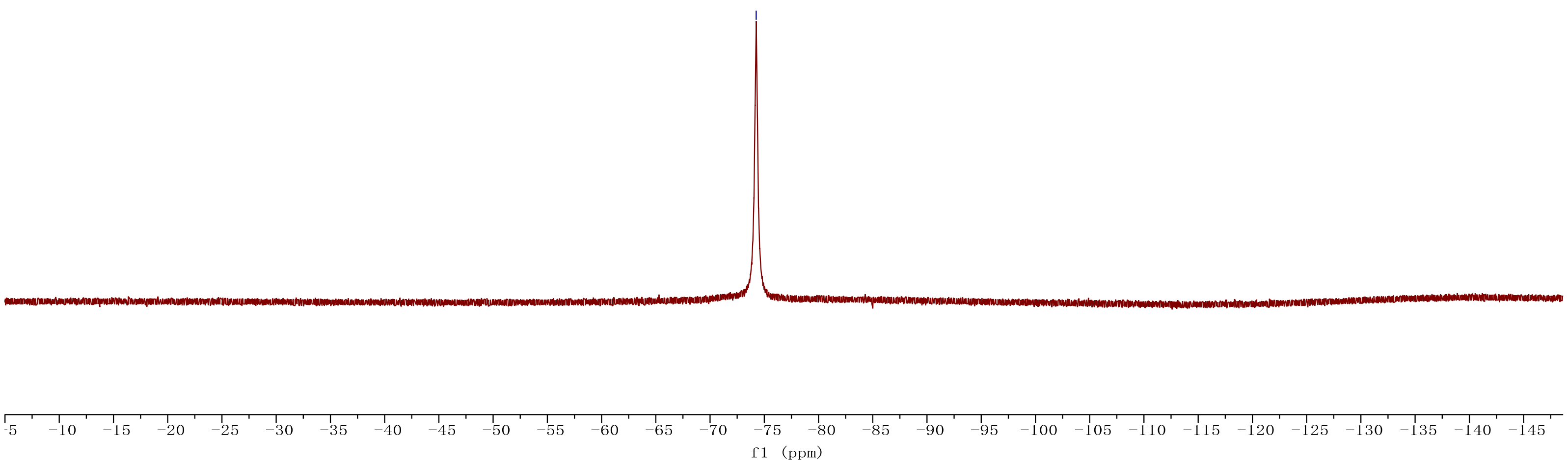

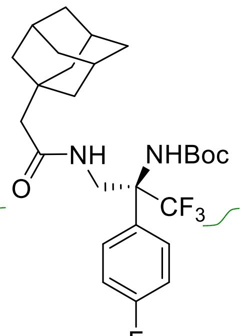

8b

^{1}H NMR ($CDCl_3$, 400 MHz)

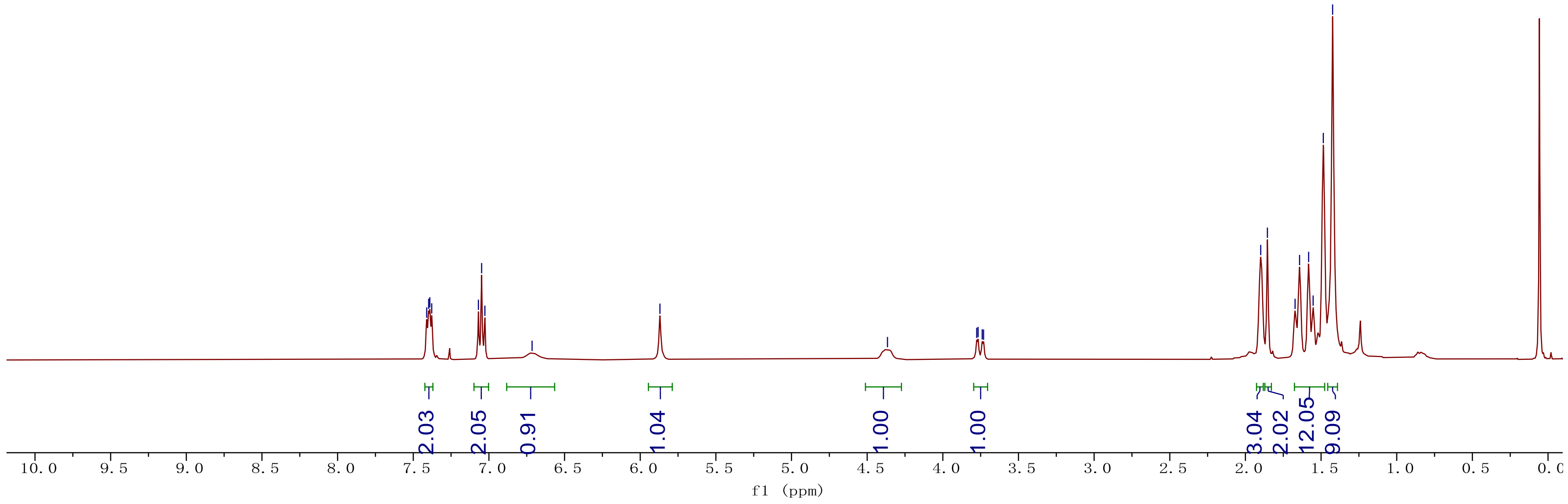

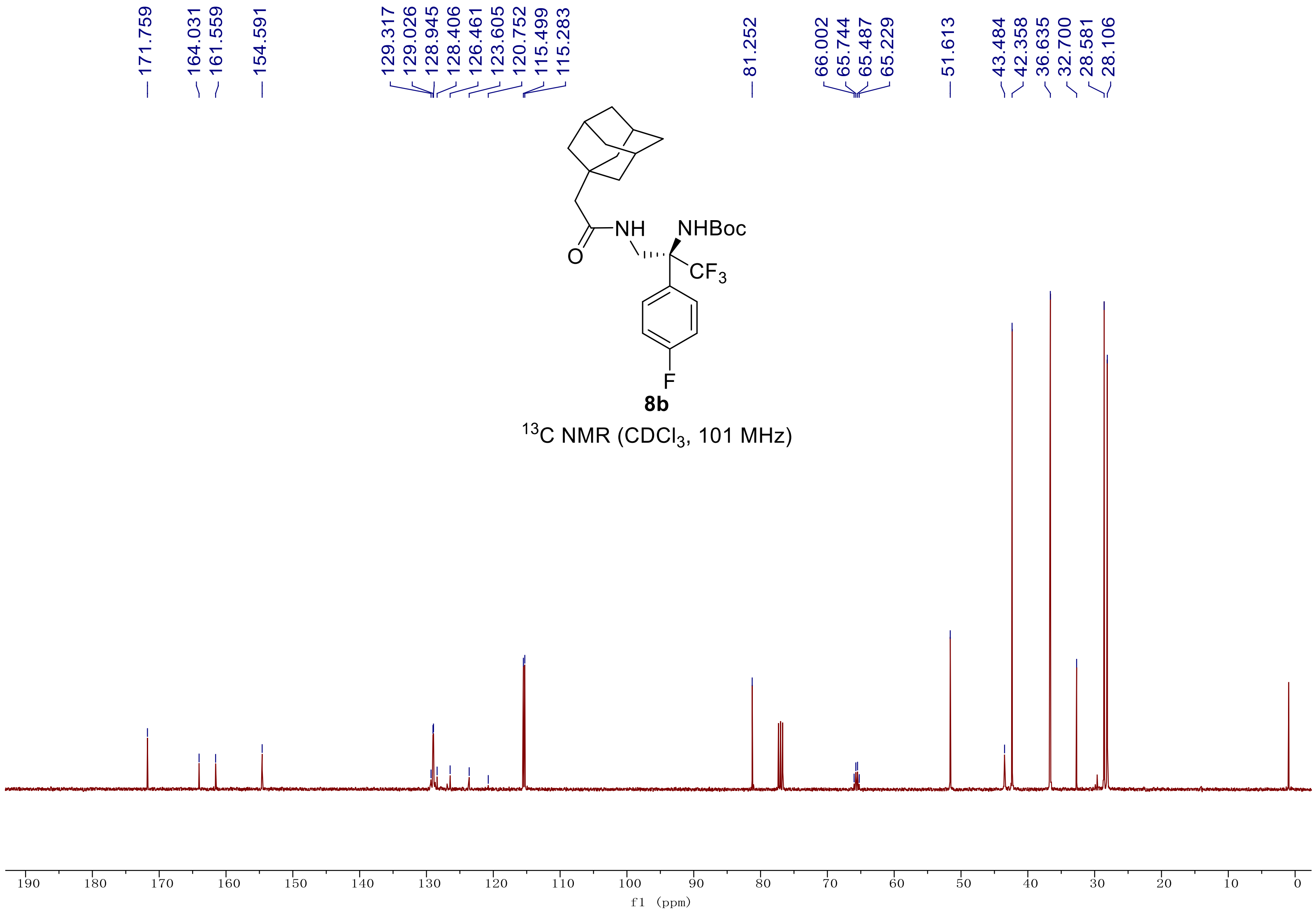

8b

^{13}C NMR ($CDCl_3$, 101 MHz)

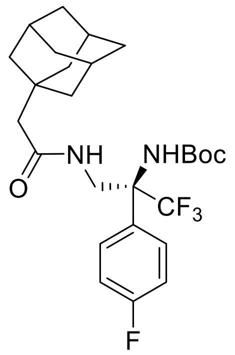

8b

^{19}F NMR ($CDCl_3$, 376 MHz)

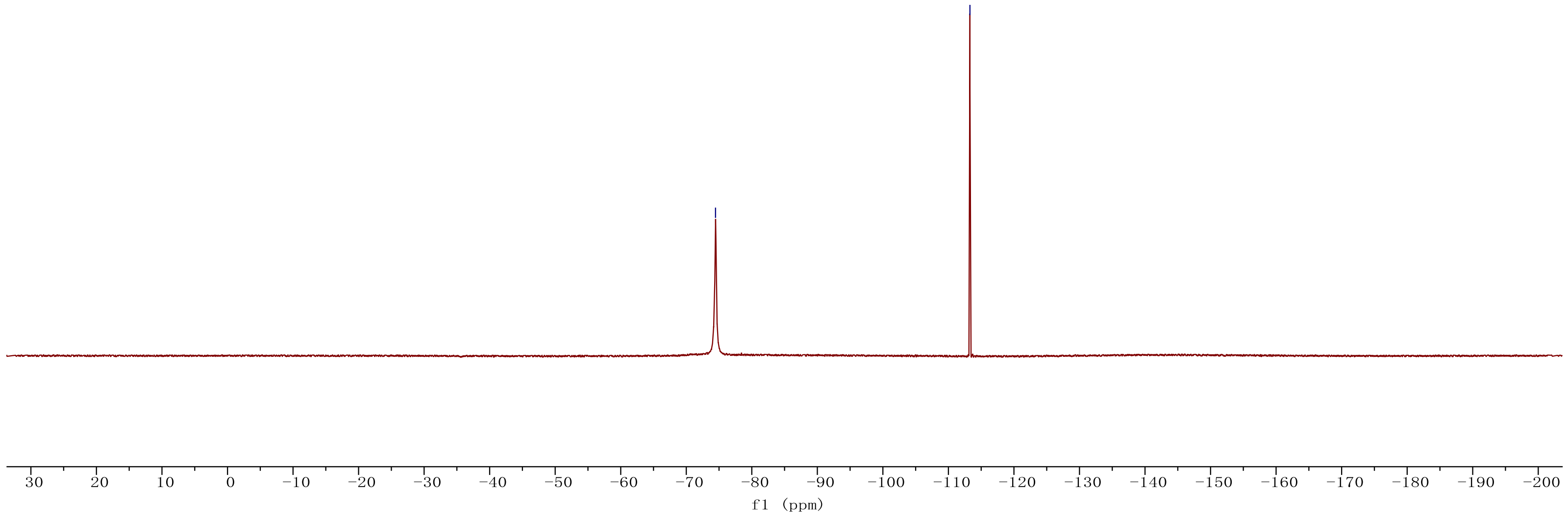

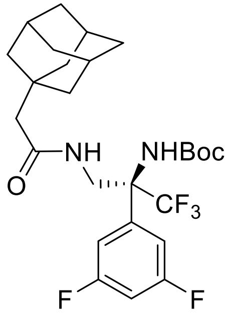

8c

^{1}H NMR ($CDCl_3$, 400 MHz)

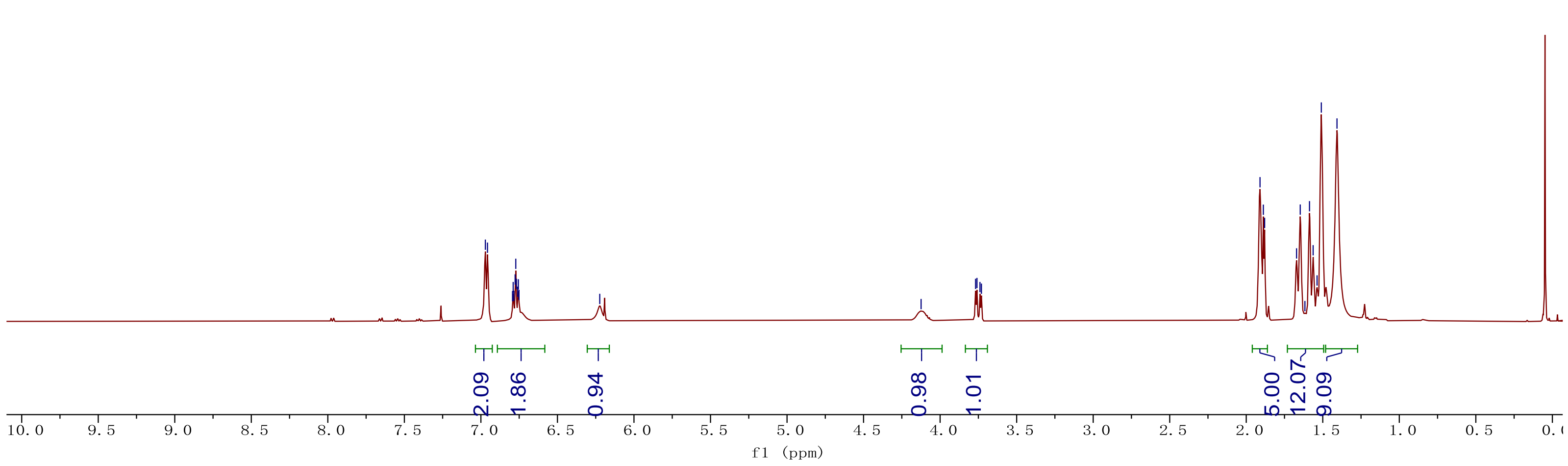

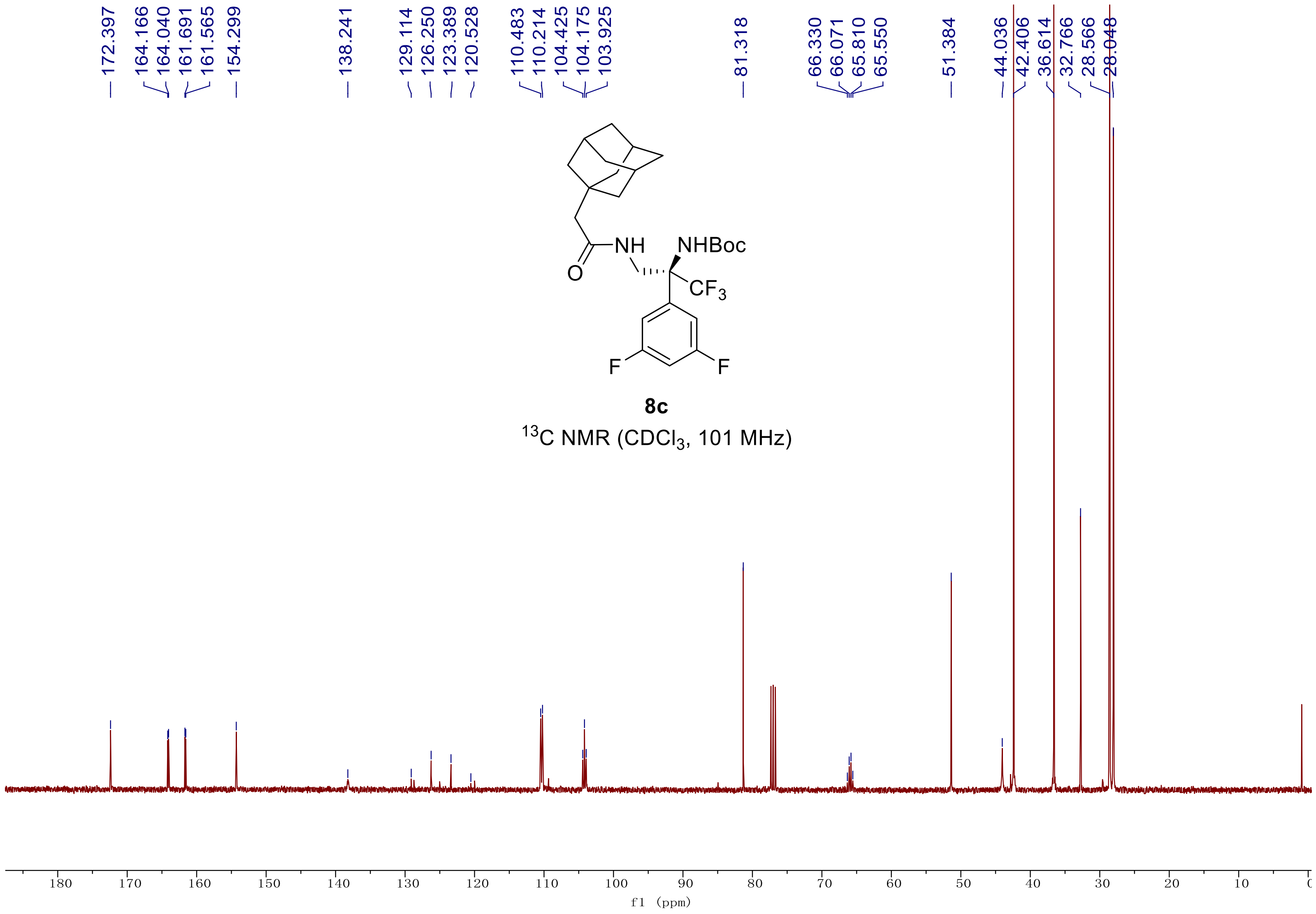

8c

^{13}C NMR ($CDCl_3$, 101 MHz)

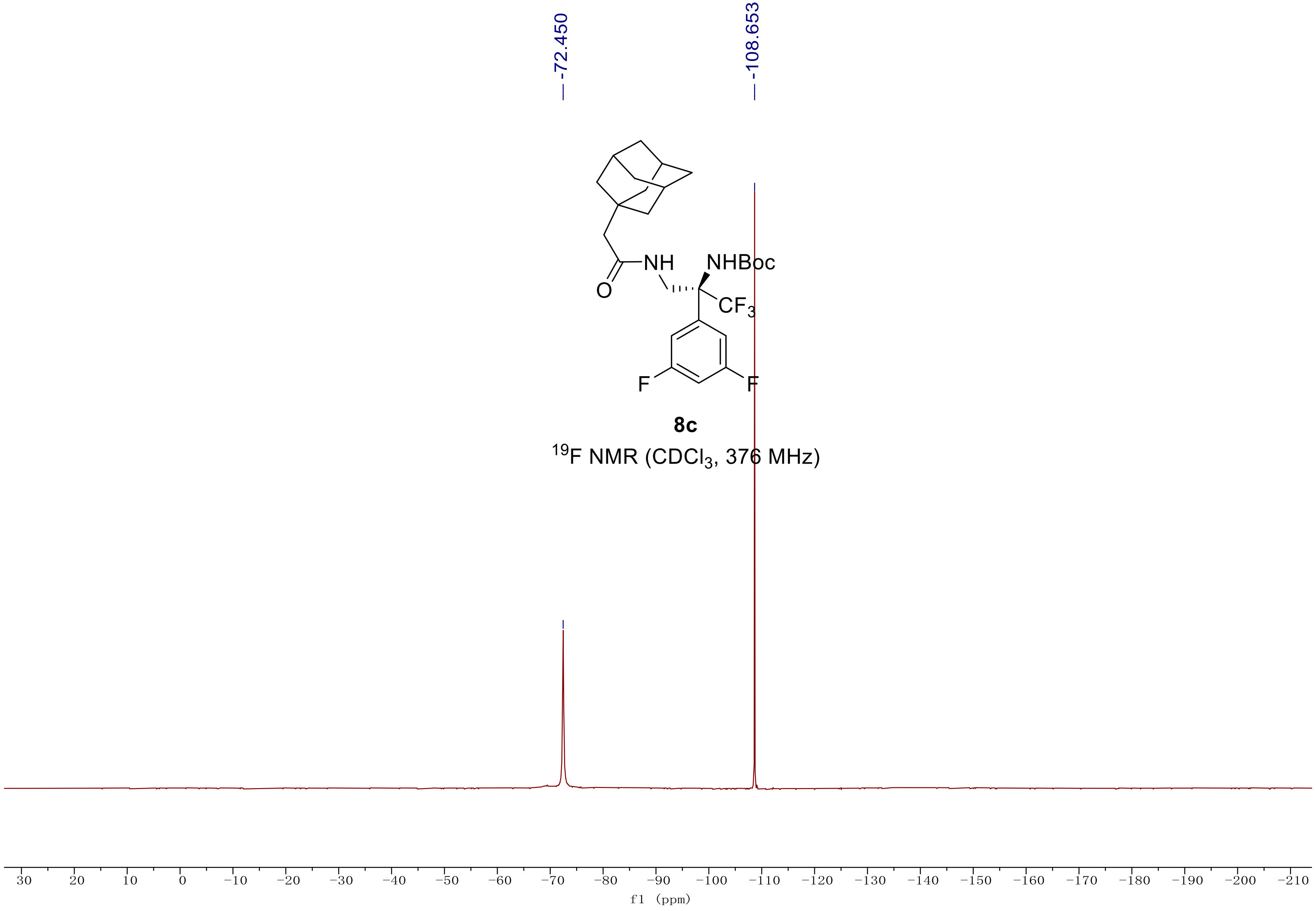

8c

^{19}F NMR ($CDCl_3$, 376 MHz)

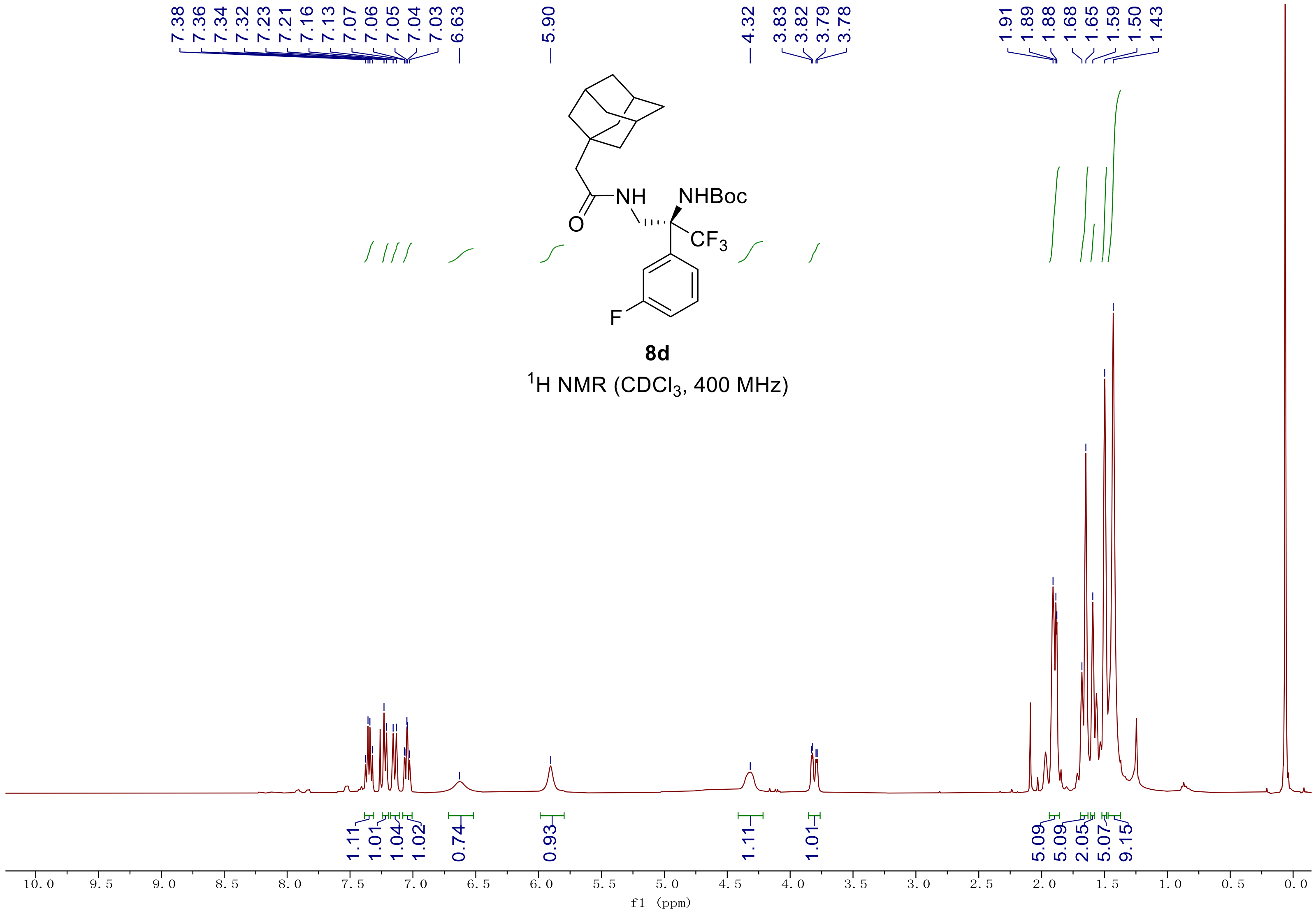
7.38 7.36 7.34 7.32 7.23 7.21 7.16 7.13 7.07 7.06 7.05 7.04 7.03 6.63
5.90
4.32 3.83 3.82 3.79 3.78
1.91 1.89 1.88 1.68 1.65 1.59 1.50 1.43
NH
NHBoc
O
CF3
F
8d
1H NMR (CDCl3, 400 MHz)
1.11 1.01 1.04 1.02 0.74 0.93 1.11 1.01 5.09 5.09 2.05 5.07 9.15
10.0 9.5 9.0 8.5 8.0 7.5 7.0 6.5 6.0 5.5 5.0 4.5 4.0 3.5 3.0 2.5 2.0 1.5 1.0 0.5 0.0
f1 (ppm)

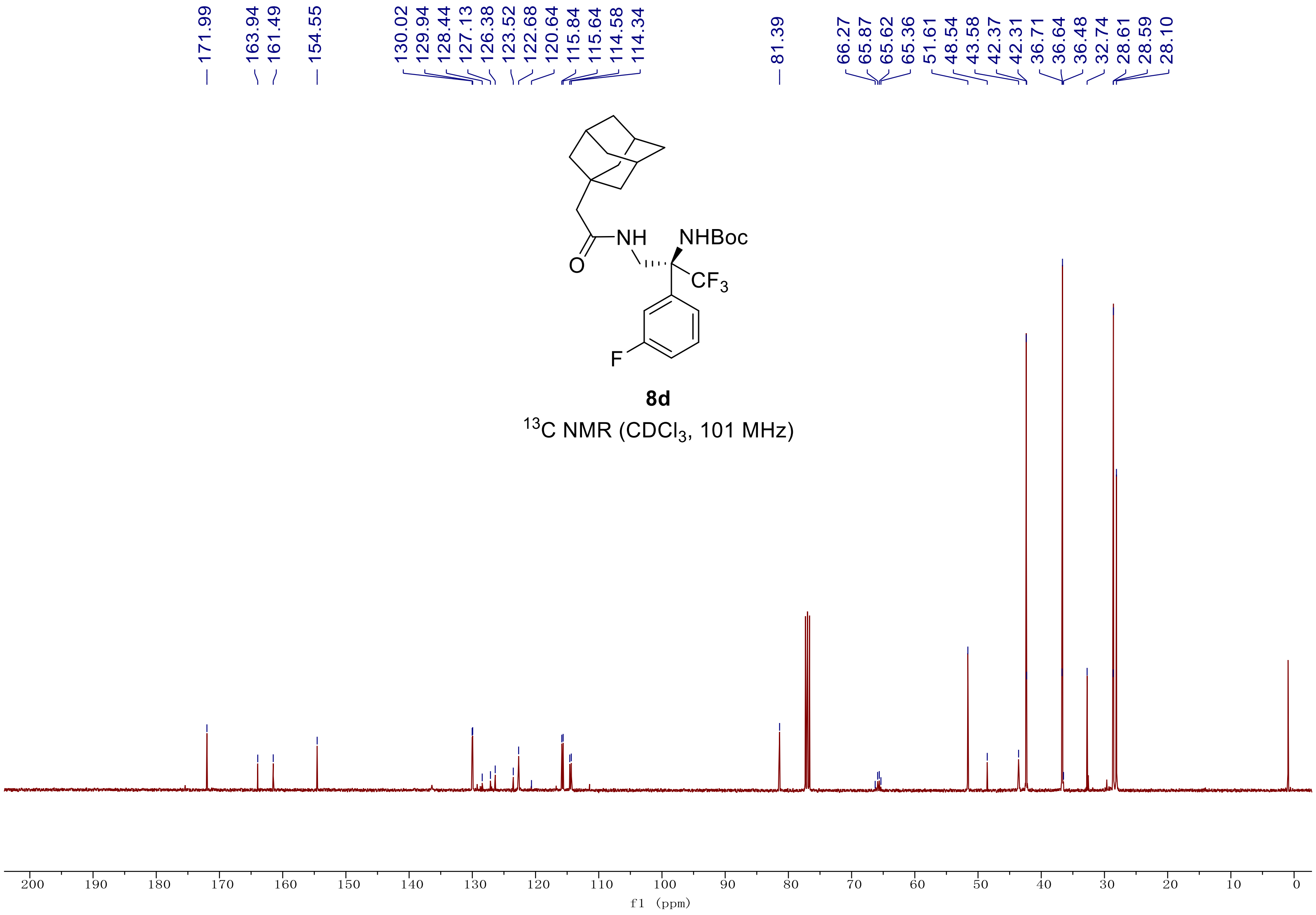

8d

^{13}C NMR (CDCl$_3$, 101 MHz)

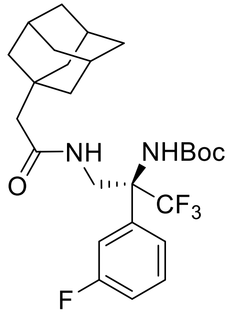

8d

^{19}F NMR ($CDCl_3$, 376 MHz)

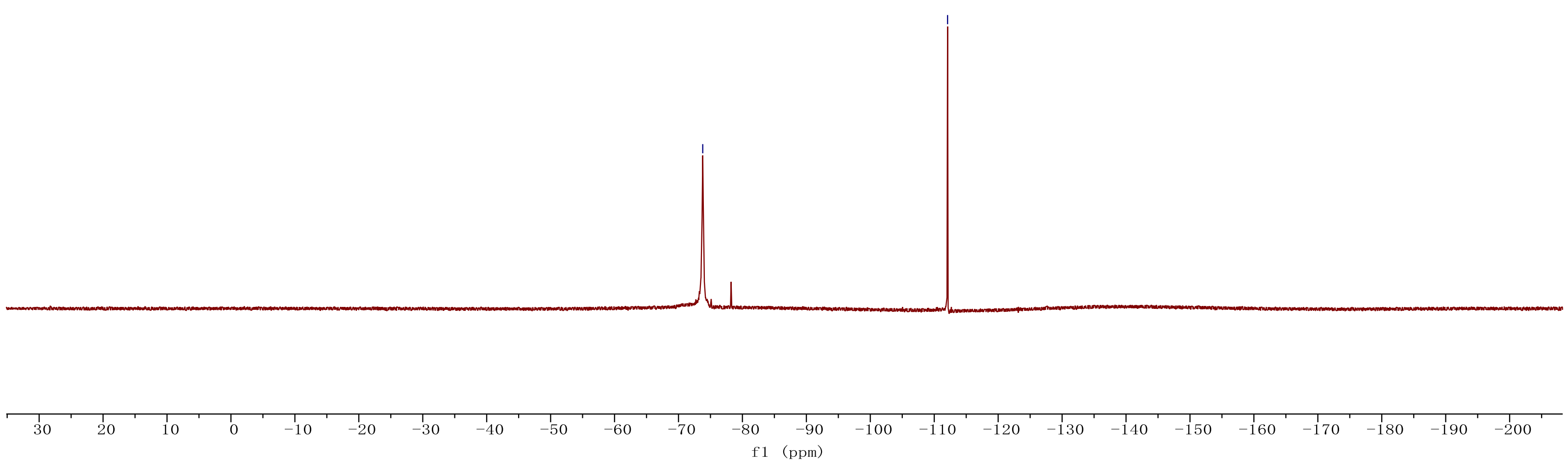

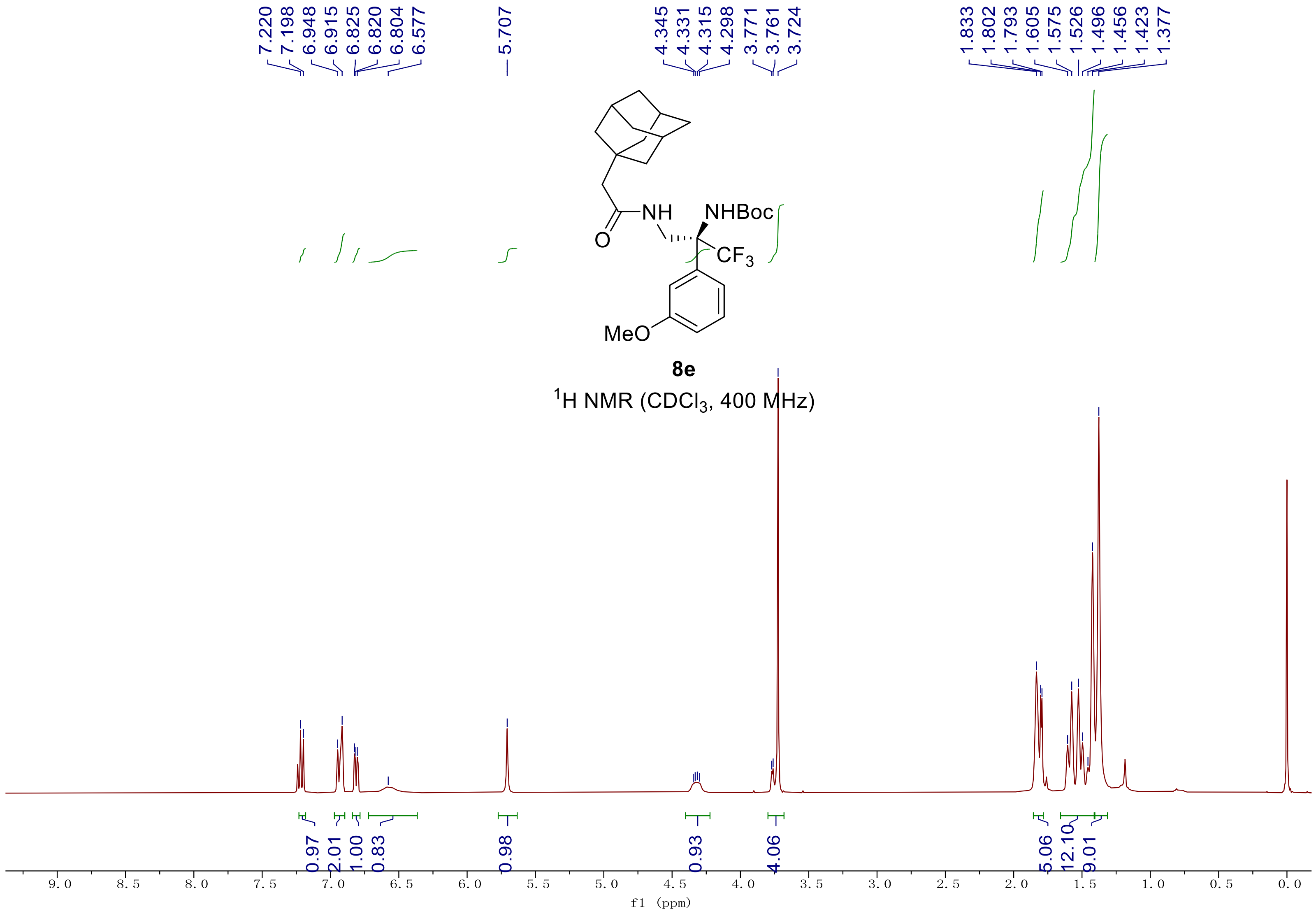

8e

^{1}H NMR ($CDCl_3$, 400 MHz)

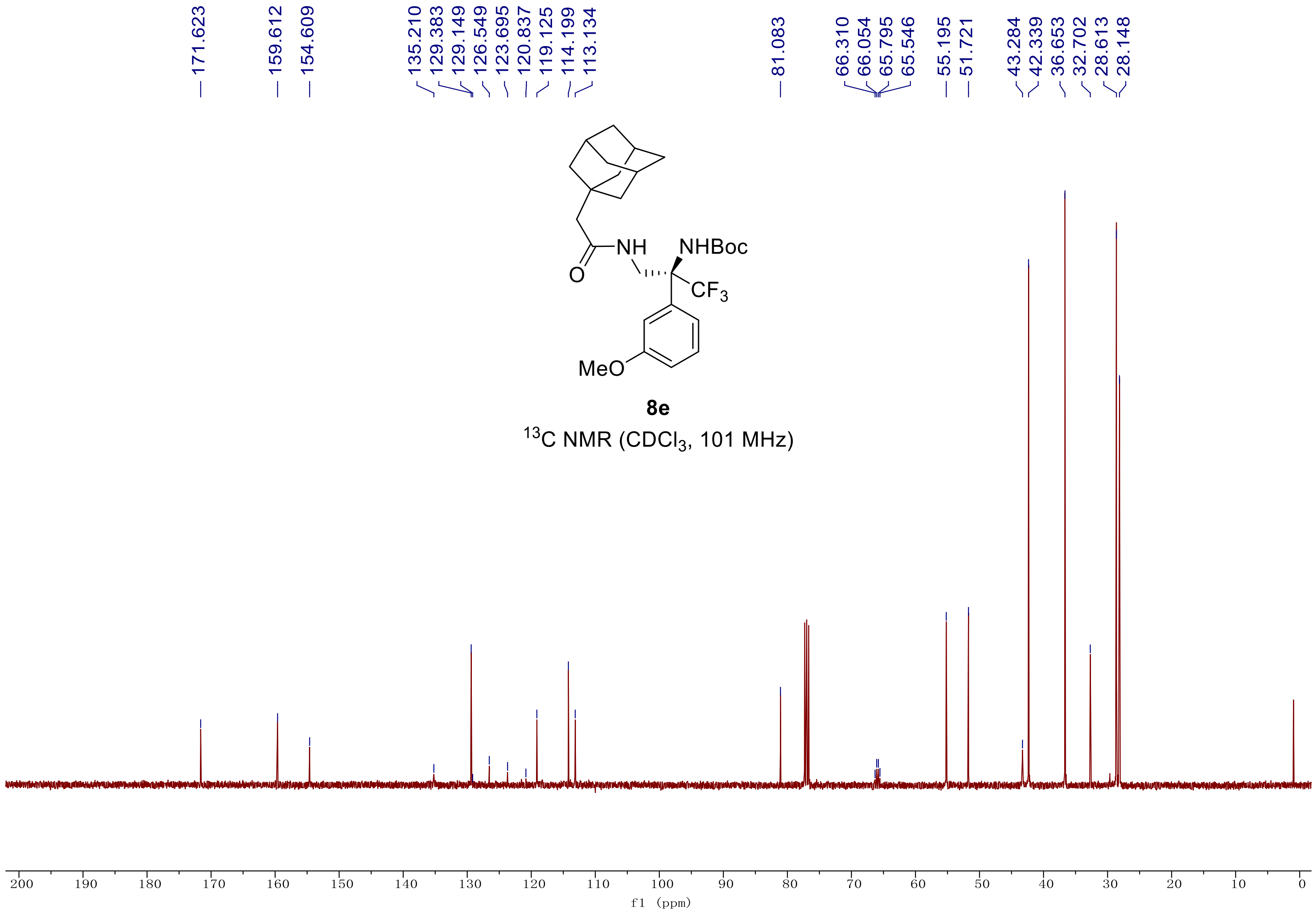

8e

^{13}C NMR ($CDCl_3$, 101 MHz)

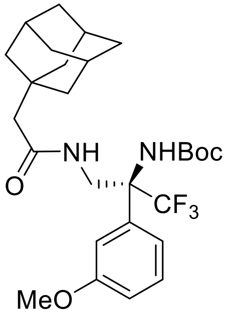

8e

^{19}F NMR ($CDCl_3$, 376 MHz)

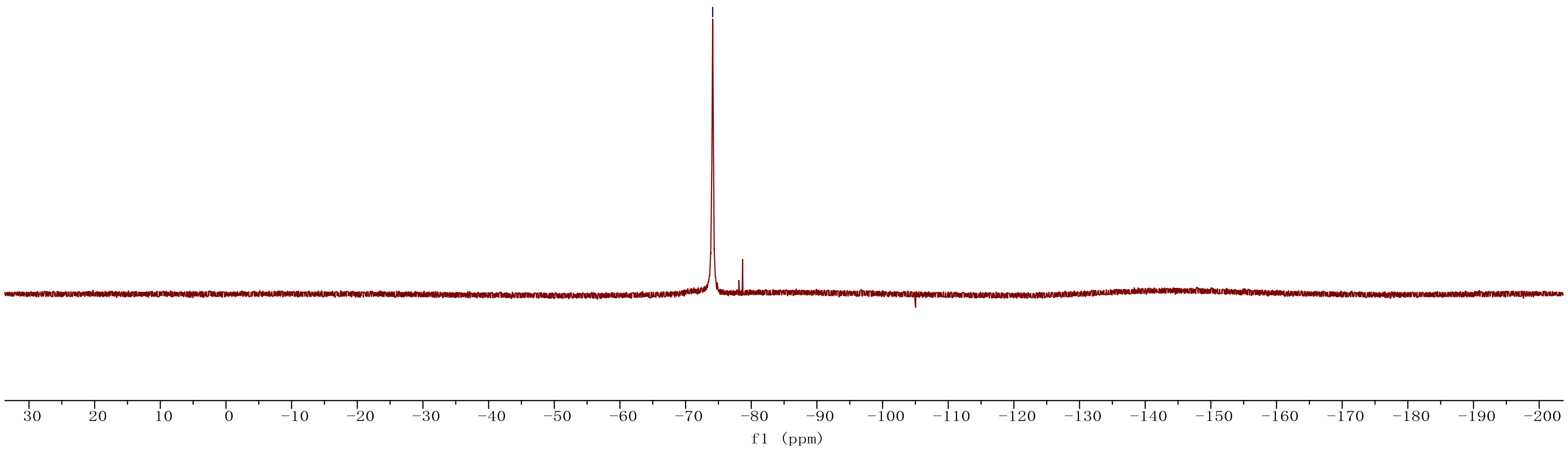

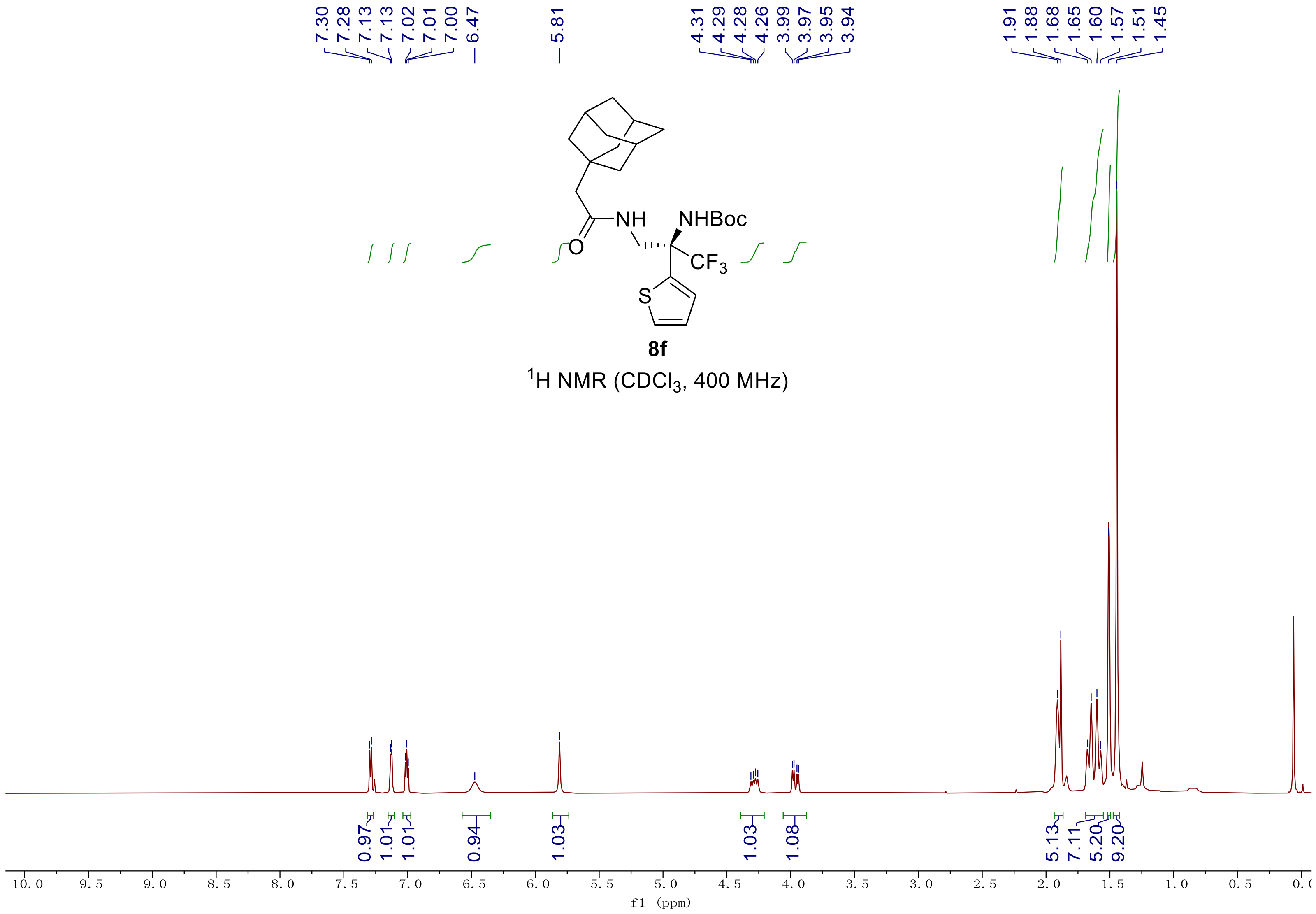
7.30
7.28
7.13
7.13
7.02
7.01
7.00
6.47
5.81
4.31
4.29
4.28
4.26
3.99
3.97
3.95
3.94
1.91
1.88
1.68
1.65
1.60
1.57
1.51
1.45
NH
NHBoc
O
CF3
S
8f
1H NMR (CDCl3, 400 MHz)
0.97
1.01
1.01
0.94
1.03
1.03
1.08
5.13
7.11
5.20
9.20
10.0 9.5 9.0 8.5 8.0 7.5 7.0 6.5 6.0 5.5 5.0 4.5 4.0 3.5 3.0 2.5 2.0 1.5 1.0 0.5 0.0
f1 (ppm)

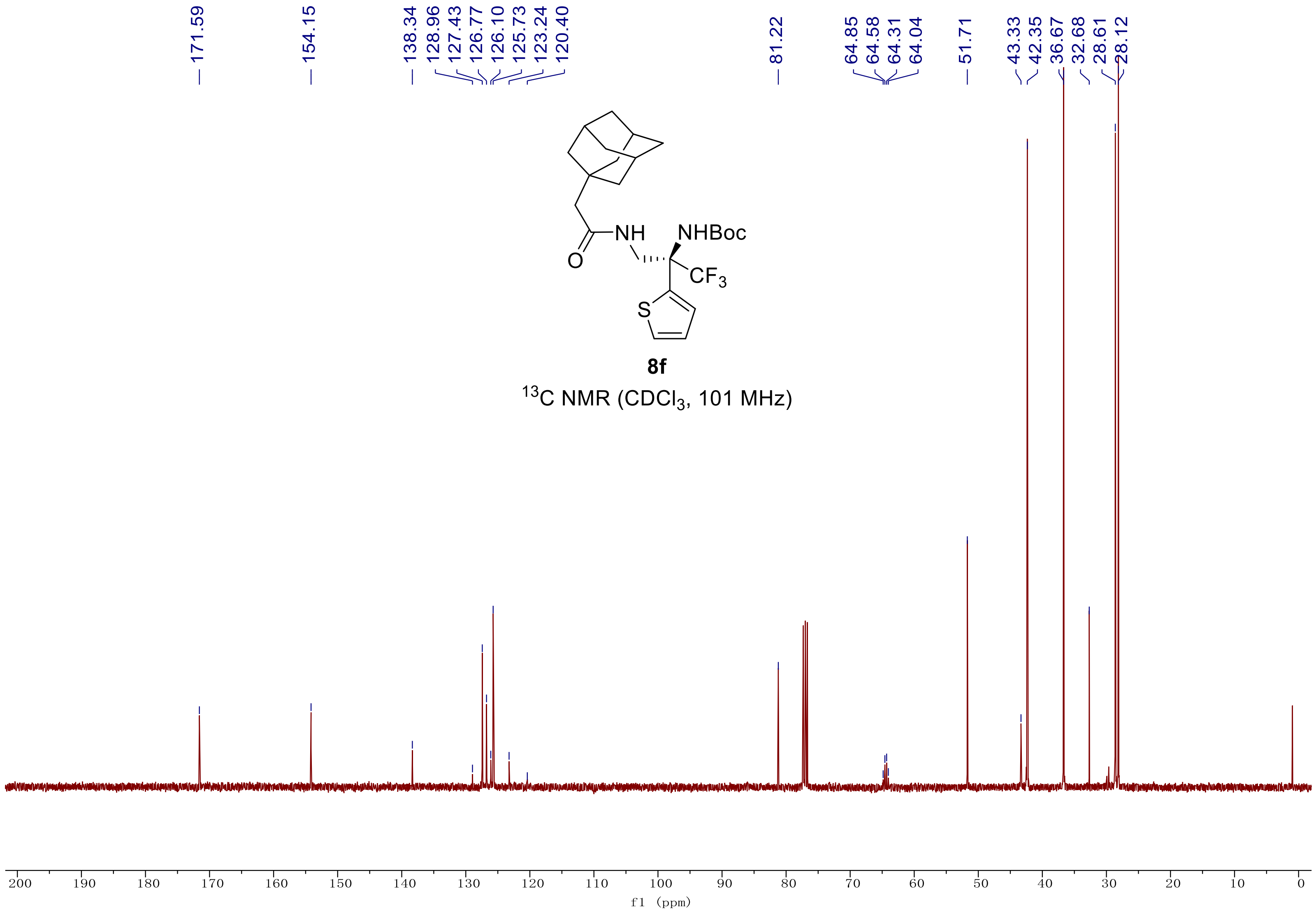
171.59
154.15
138.34
128.96
127.43
126.77
126.10
125.73
123.24
120.40
81.22
64.85
64.58
64.31
64.04
51.71
43.33
42.35
36.67
32.68
28.61
28.12
NH
NHBoc
O
CF3
S
8f
13C NMR (CDCl3, 101 MHz)
200
190
180
170
160
150
140
130
120
110
100
90
80
70
60
50
40
30
20
10
0
f1 (ppm)

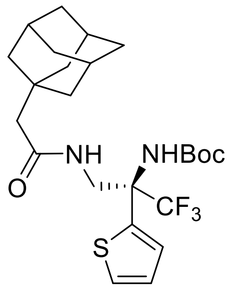

8f

^{19}F NMR ($CDCl_3$, 376 MHz)

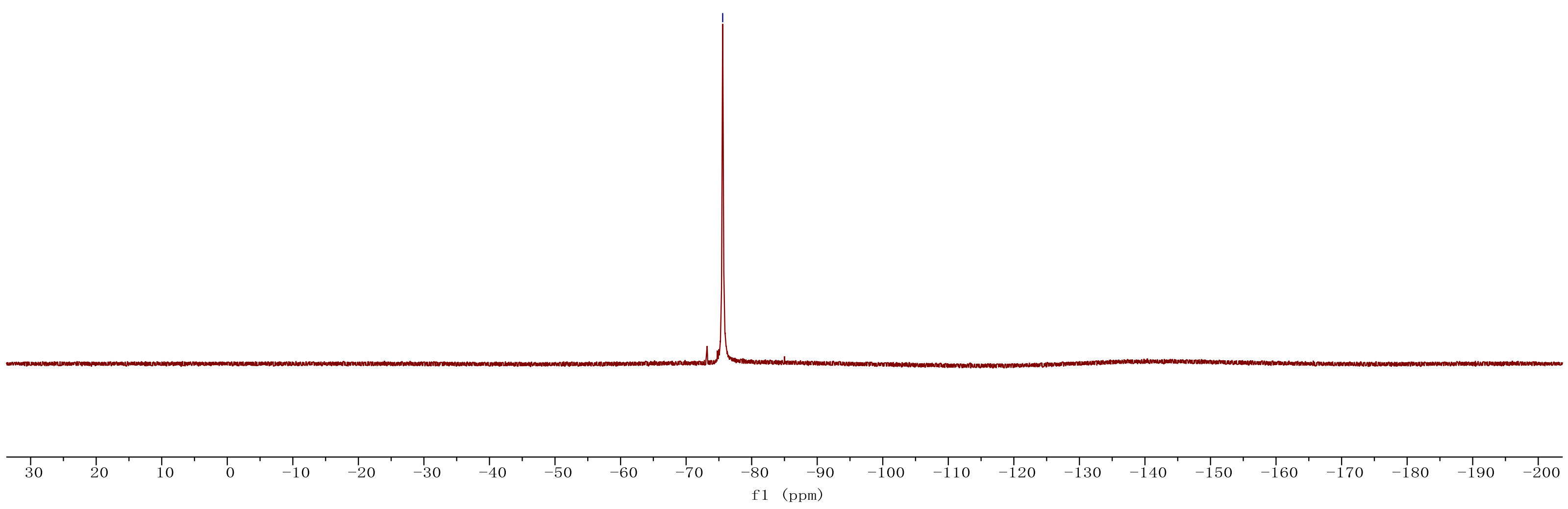

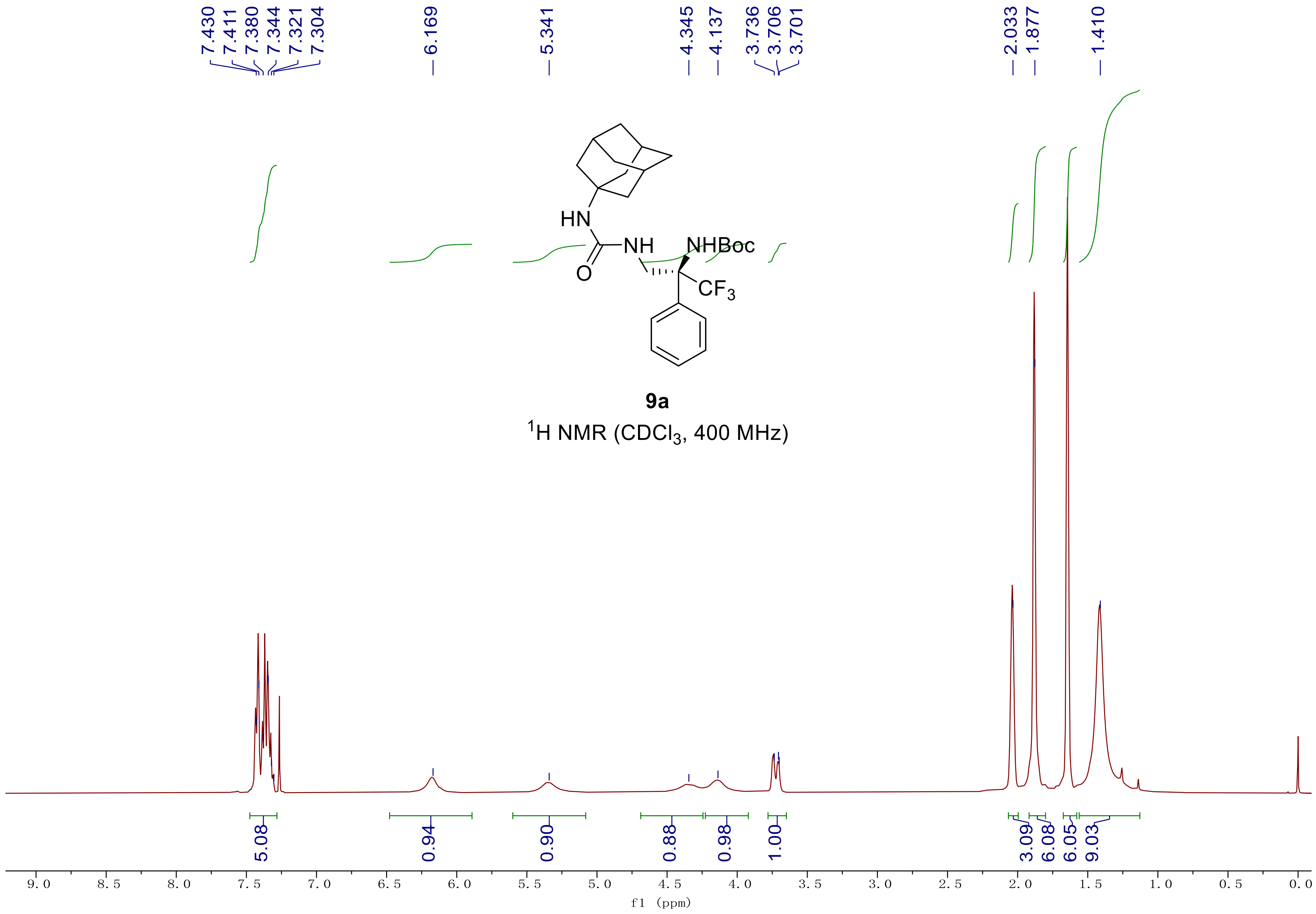
7.430
7.411
7.380
7.344
7.321
7.304
6.169
5.341
4.345
4.137
3.736
3.706
3.701
2.033
1.877
1.410
HN
NH
NHBoc
O
CF3
9a
1H NMR (CDCl3, 400 MHz)
5.08
0.94
0.90
0.88
0.98
1.00
3.09
6.08
6.05
9.03
9.0
8.5
8.0
7.5
7.0
6.5
6.0
5.5
5.0
4.5
4.0
3.5
3.0
2.5
2.0
1.5
1.0
0.5
0.0
f1 (ppm)

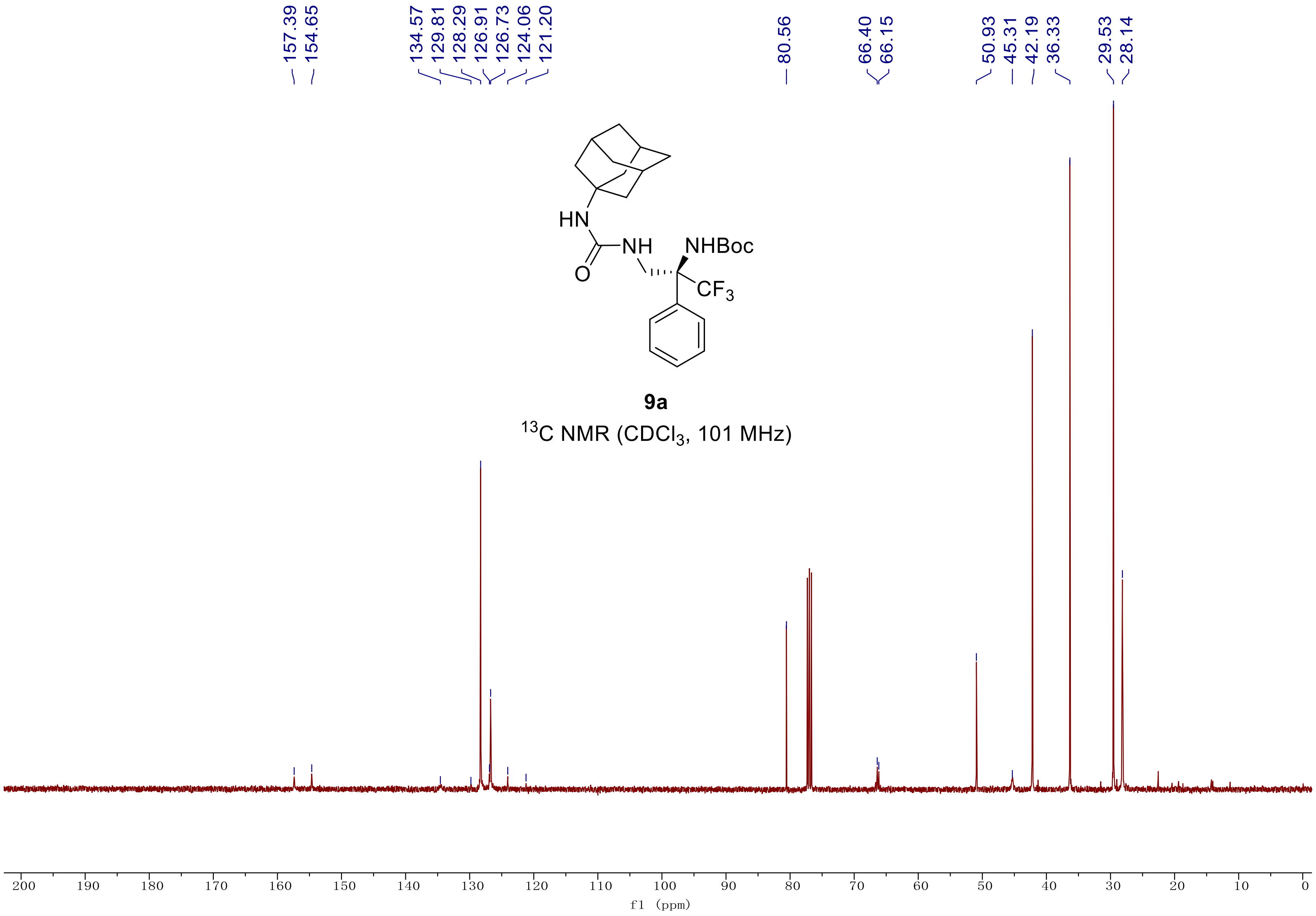

9a

^{13}C NMR ($CDCl_3$, 101 MHz)

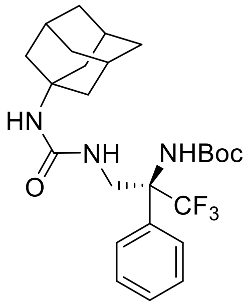

9a

^{19}F NMR ($CDCl_3$, 376 MHz)

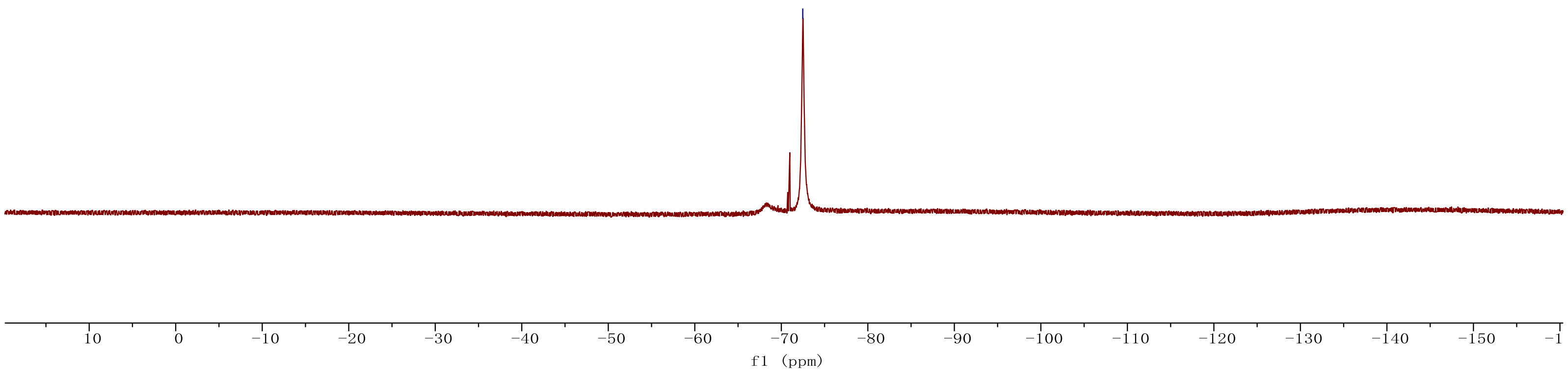

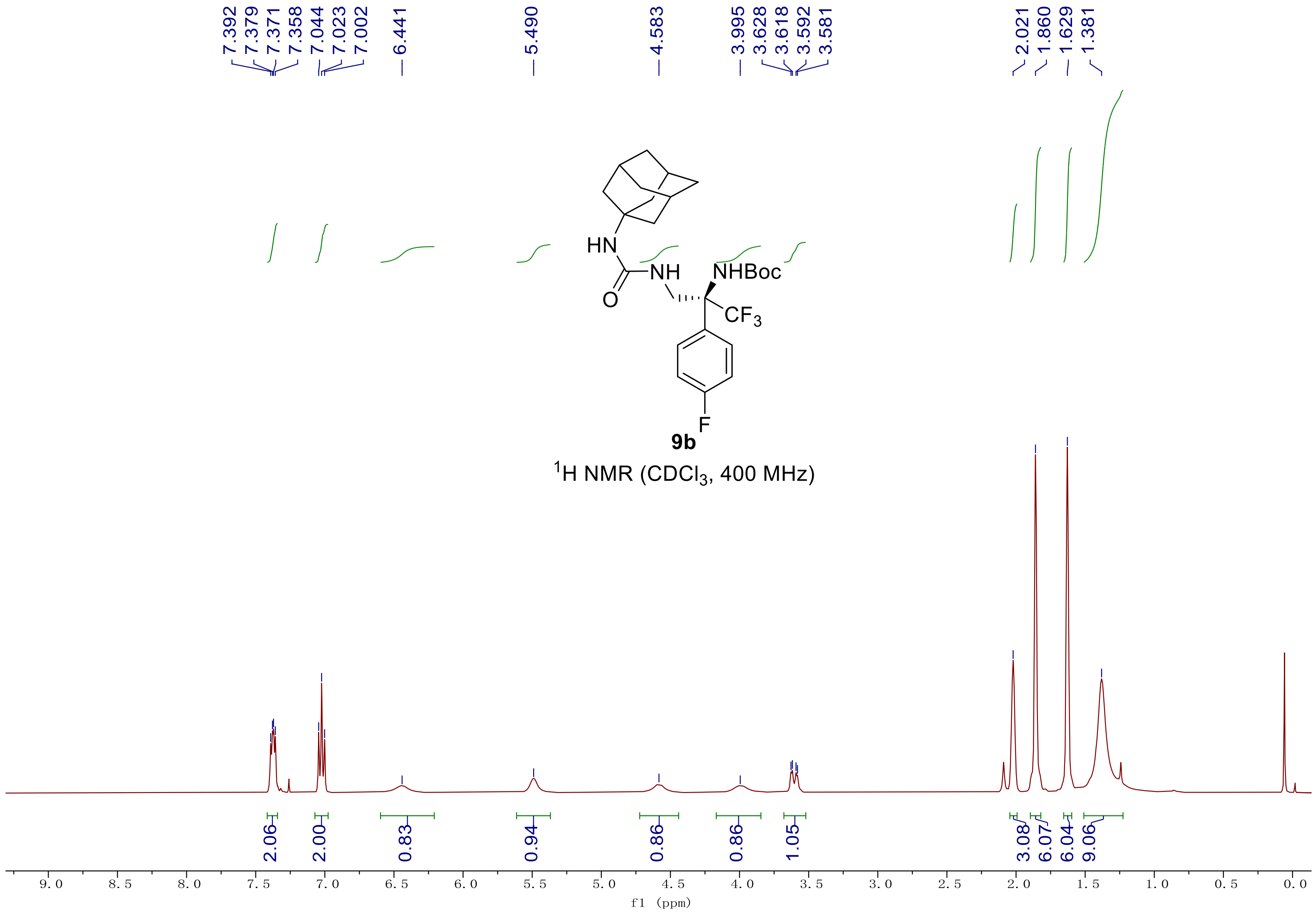
7.392
7.379
7.371
7.358
7.044
7.023
7.002
6.441
5.490
4.583
3.995
3.628
3.618
3.592
3.581
2.021
1.860
1.629
1.381
HN
NH
O
NHBoc
CF3
F
9b
1H NMR (CDCl3, 400 MHz)
2.06
2.00
0.83
0.94
0.86
0.86
1.05
3.08
6.07
6.04
9.06
9.0 8.5 8.0 7.5 7.0 6.5 6.0 5.5 5.0 4.5 4.0 3.5 3.0 2.5 2.0 1.5 1.0 0.5 0.0
f1 (ppm)

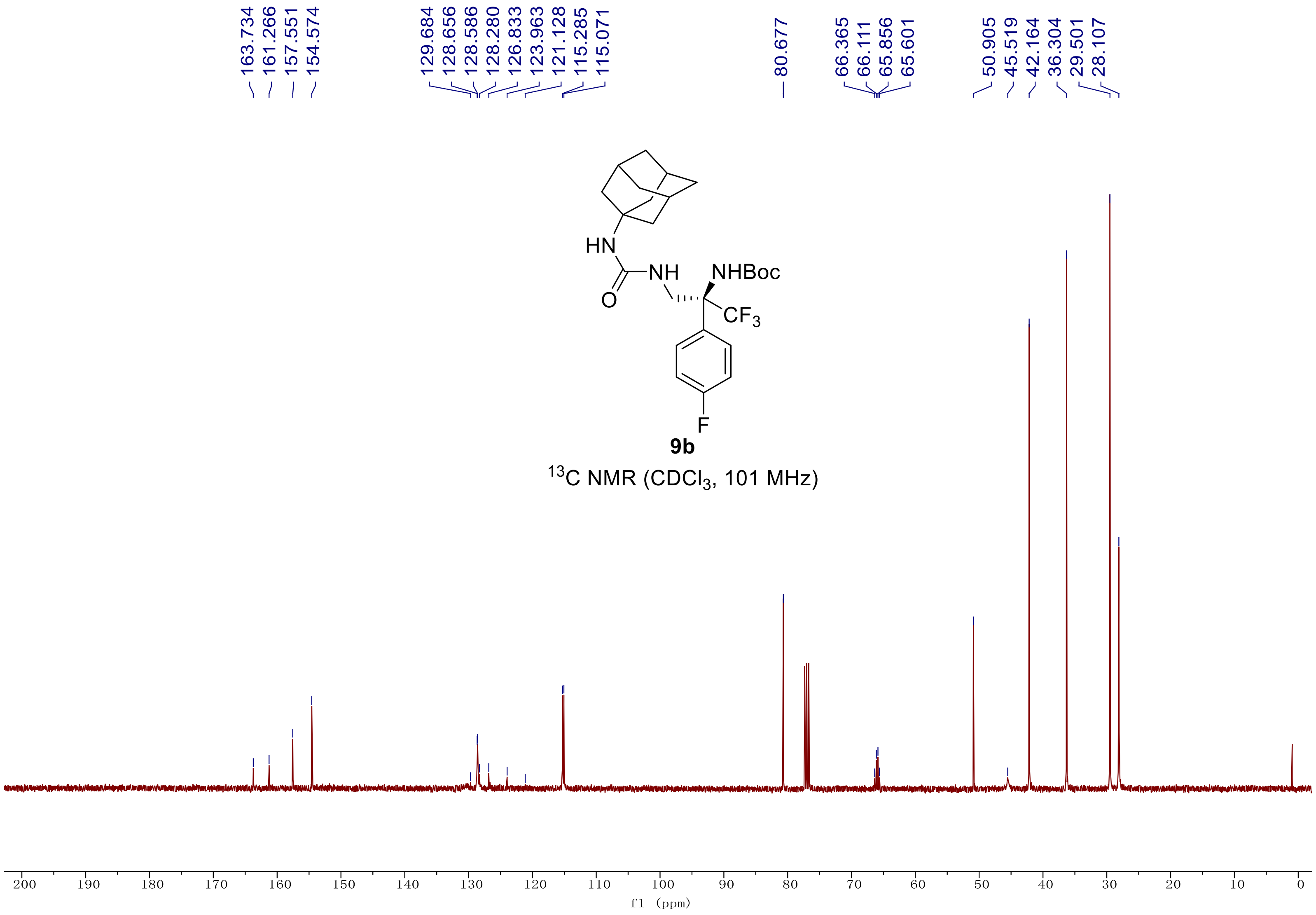

9b

^{13}C NMR ($CDCl_3$, 101 MHz)

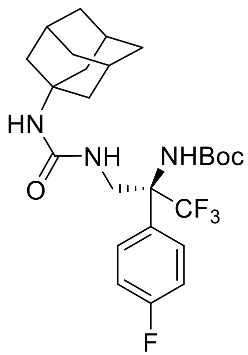

9b

^{19}F NMR ($CDCl_3$, 376 MHz)

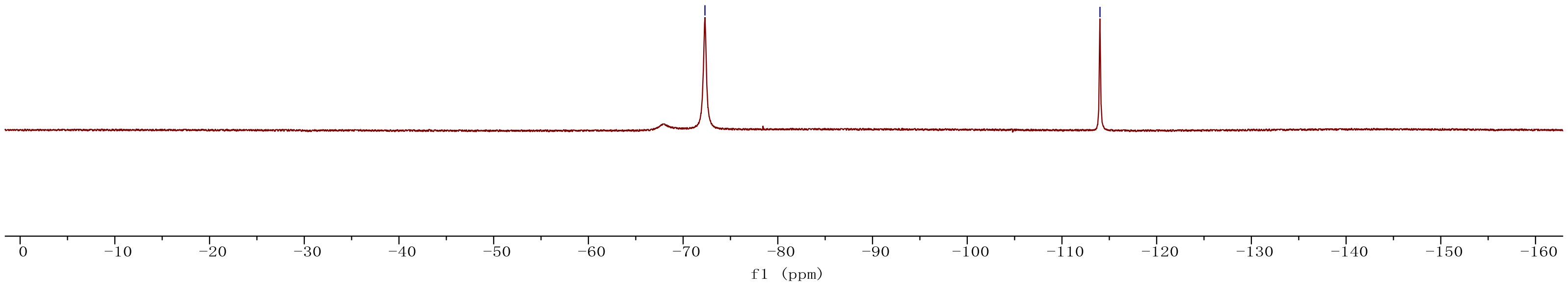

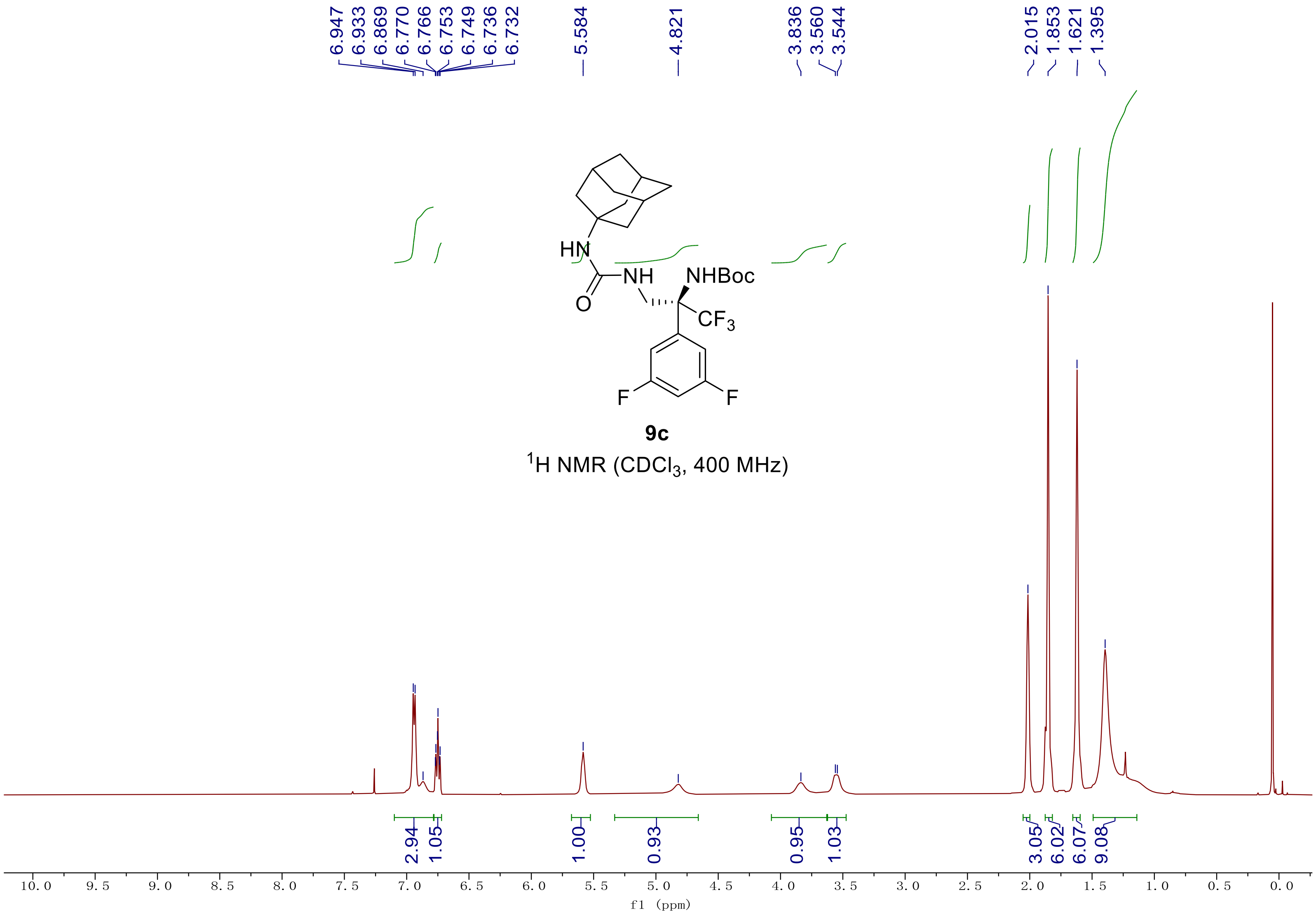

9c

^{1}H NMR ($CDCl_3$, 400 MHz)

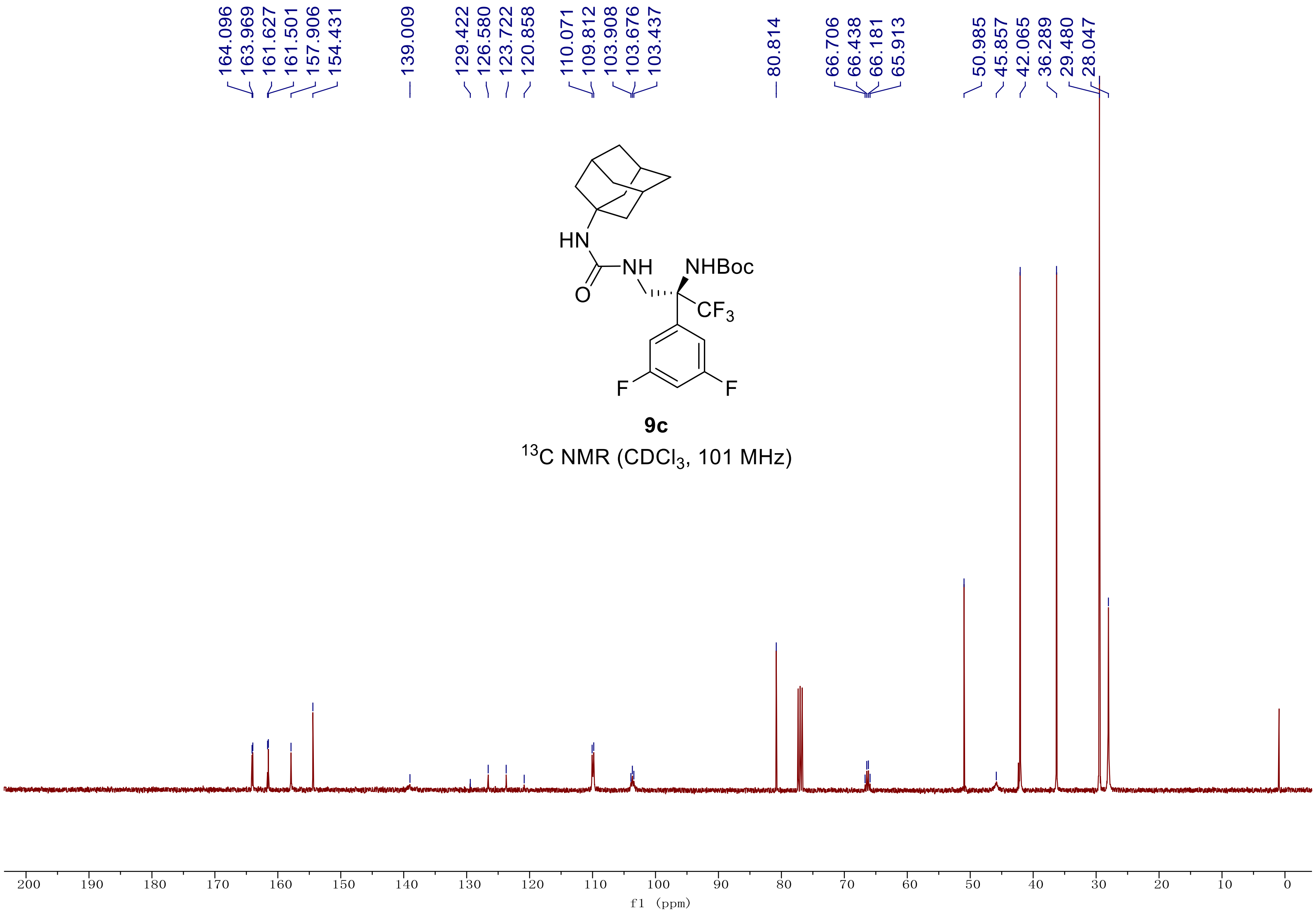

9c

^{13}C NMR ($CDCl_3$, 101 MHz)

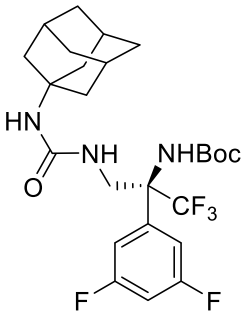

9c

^{19}F NMR ($CDCl_3$, 376 MHz)

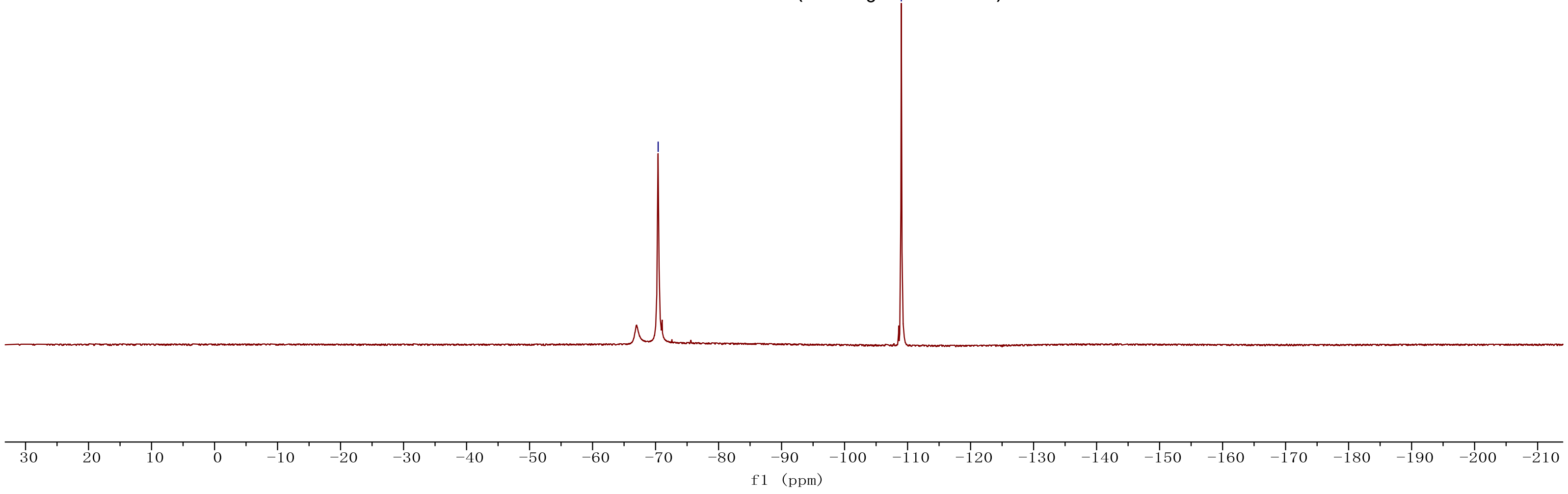

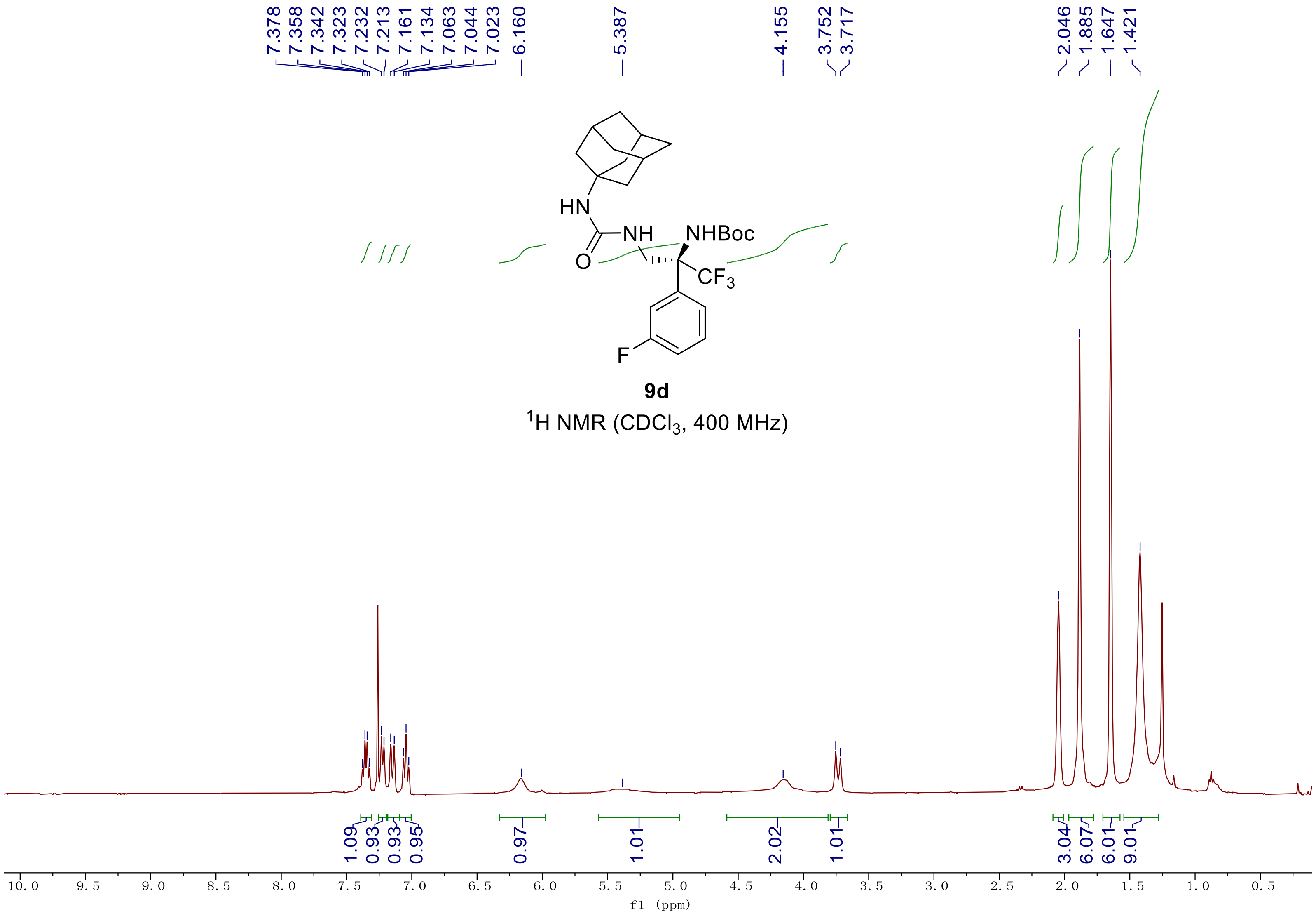

9d

^{1}H NMR ($CDCl_3$, 400 MHz)

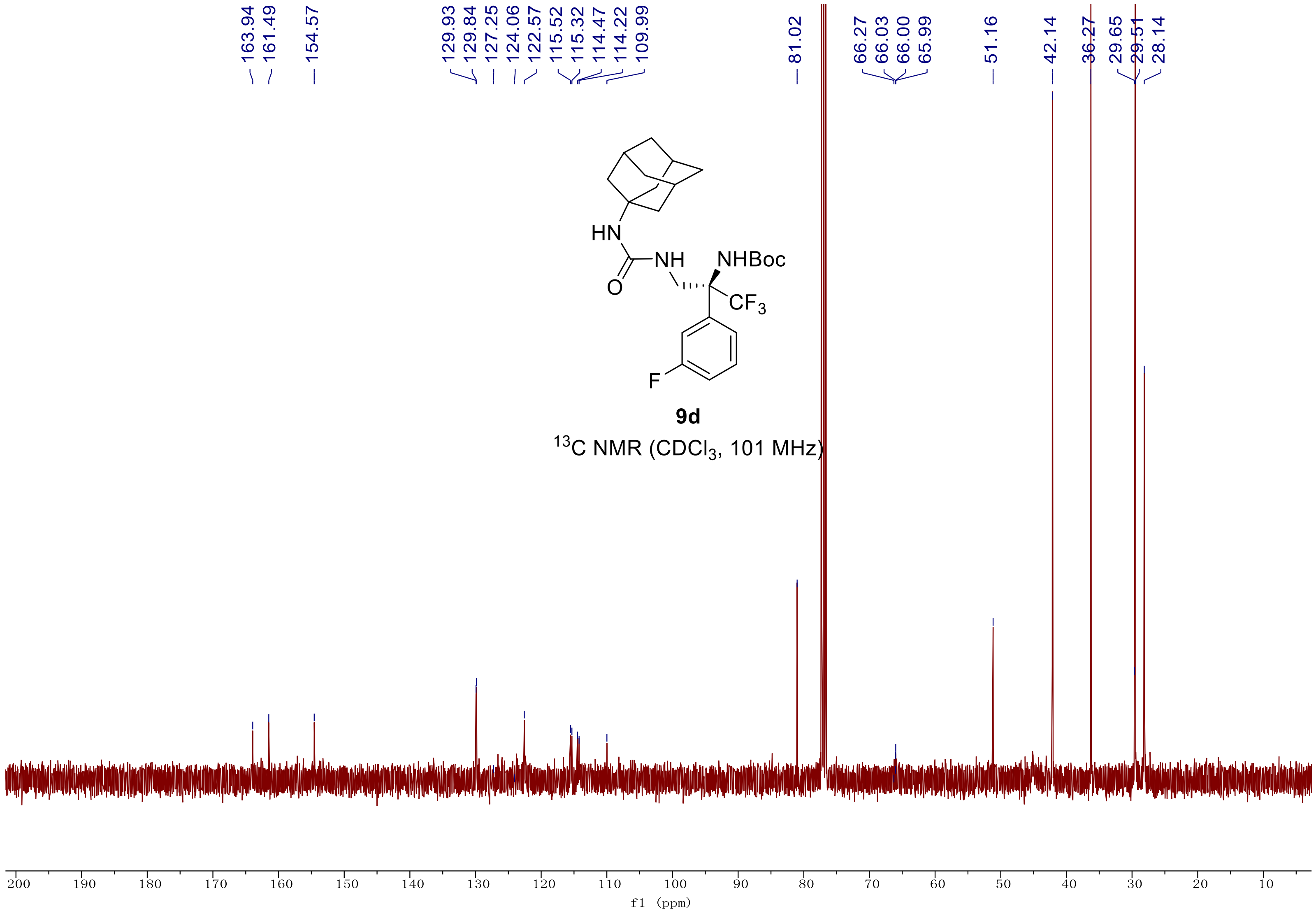
163.94
161.49
154.57
129.93
129.84
127.25
124.06
122.57
115.52
115.32
114.47
114.22
109.99
81.02
66.27
66.03
66.00
65.99
51.16
42.14
36.27
29.65
29.51
28.14
HN
NH
NHBoc
O
CF3
F
9d
13C NMR (CDCl3, 101 MHz)
200 190 180 170 160 150 140 130 120 110 100 90 80 70 60 50 40 30 20 10
f1 (ppm)

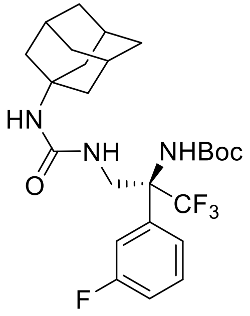

9d

^{19}F NMR ($CDCl_3$, 376 MHz)

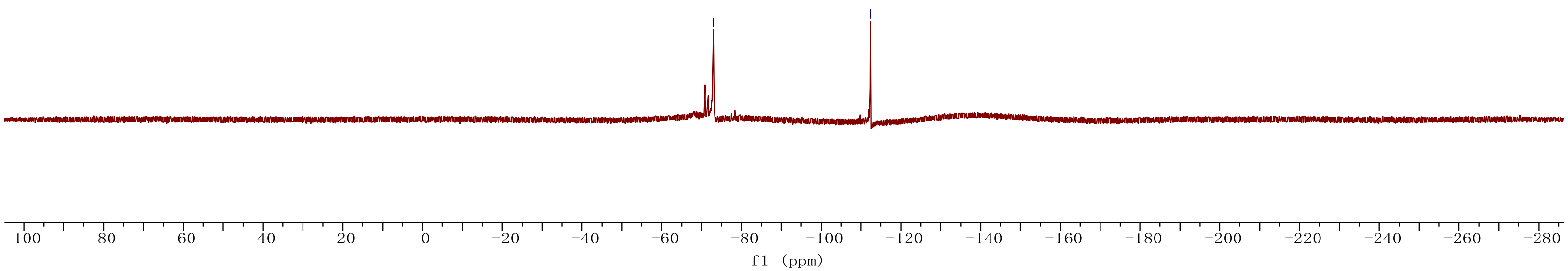

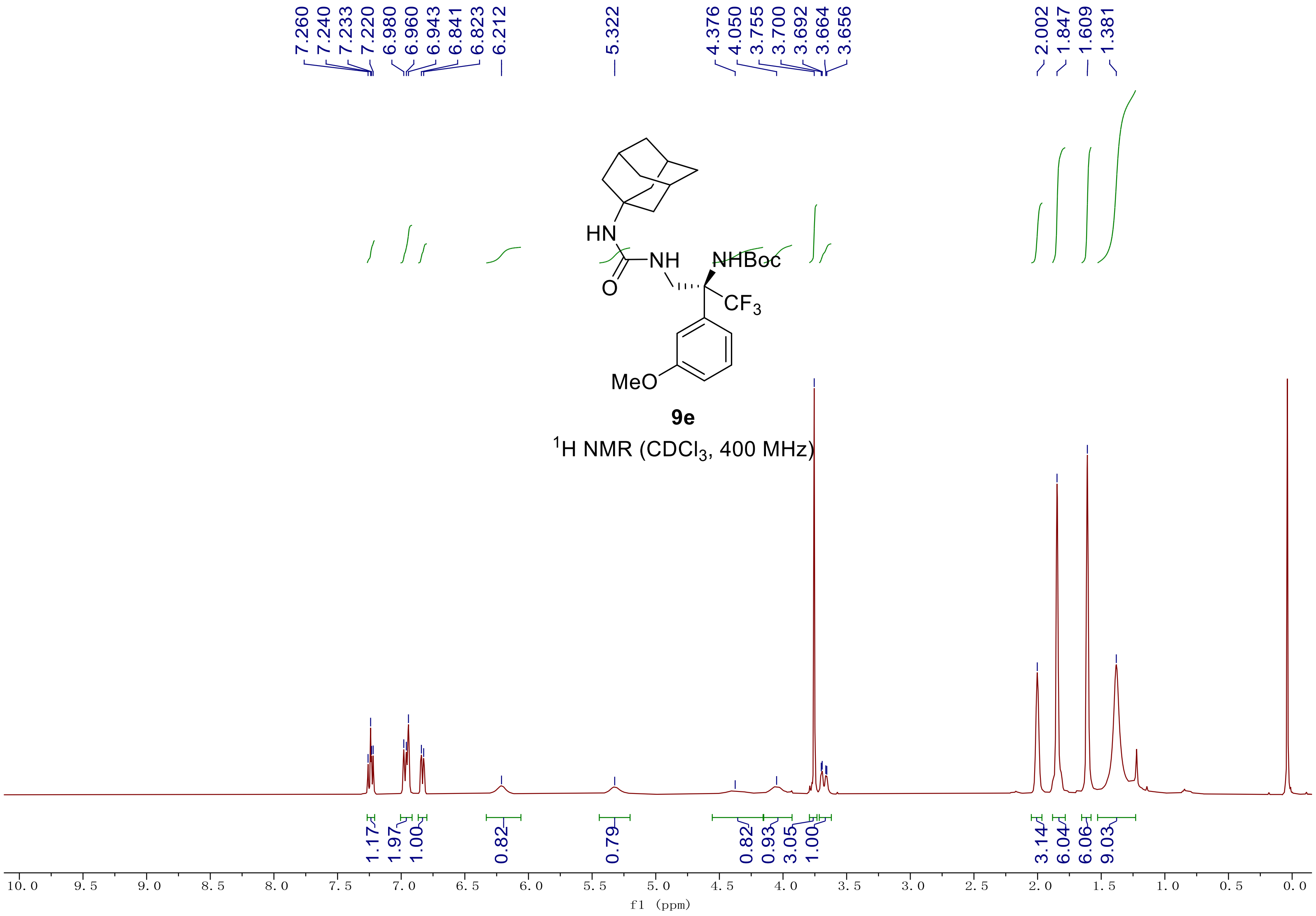

9e

^{1}H NMR ($CDCl_3$, 400 MHz)

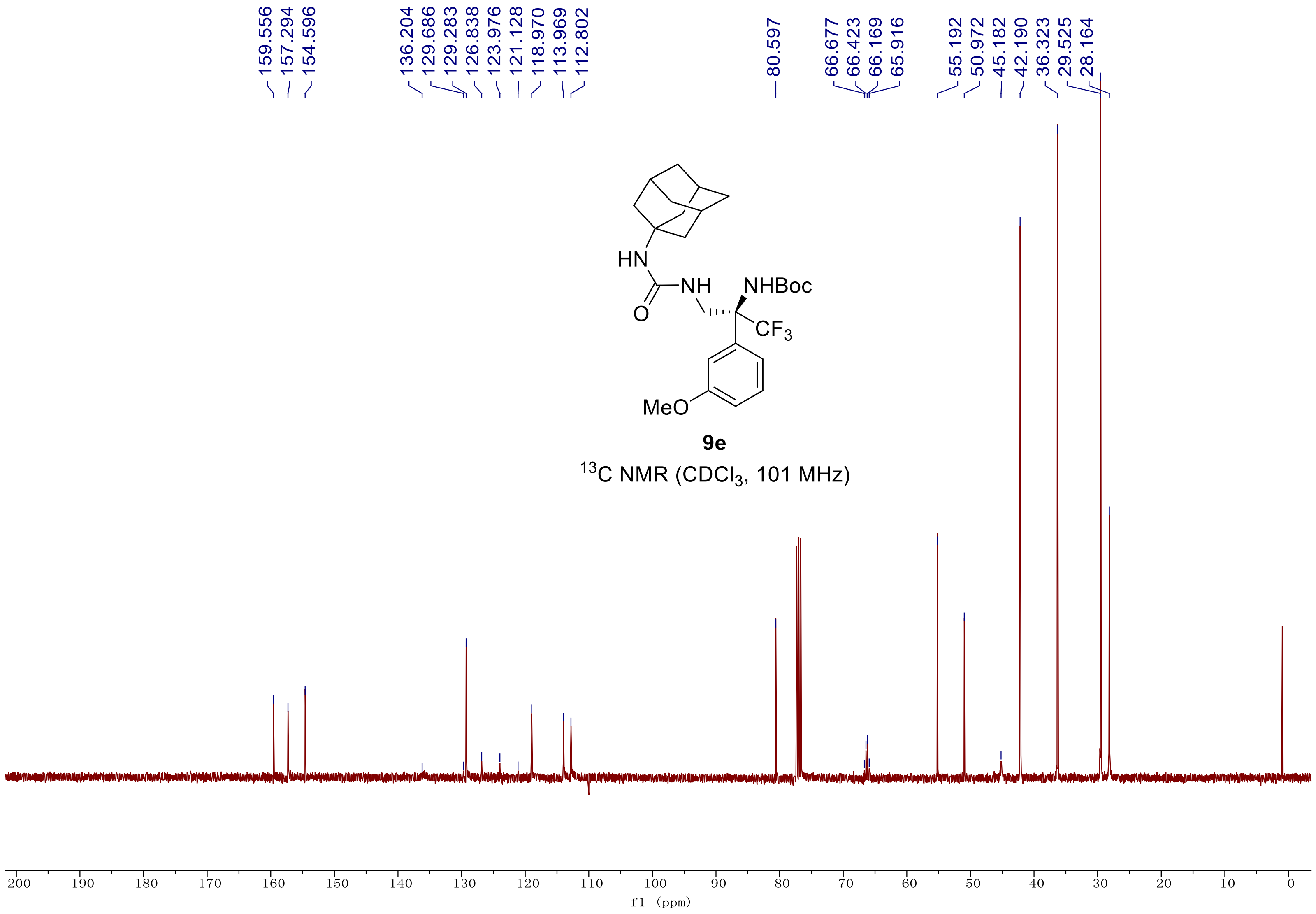

9e

^{13}C NMR ($CDCl_3$, 101 MHz)

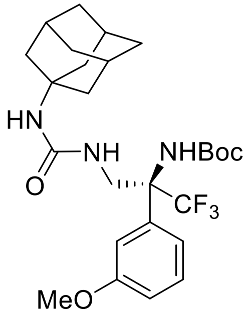

9e

^{19}F NMR ($CDCl_3$, 376 MHz)

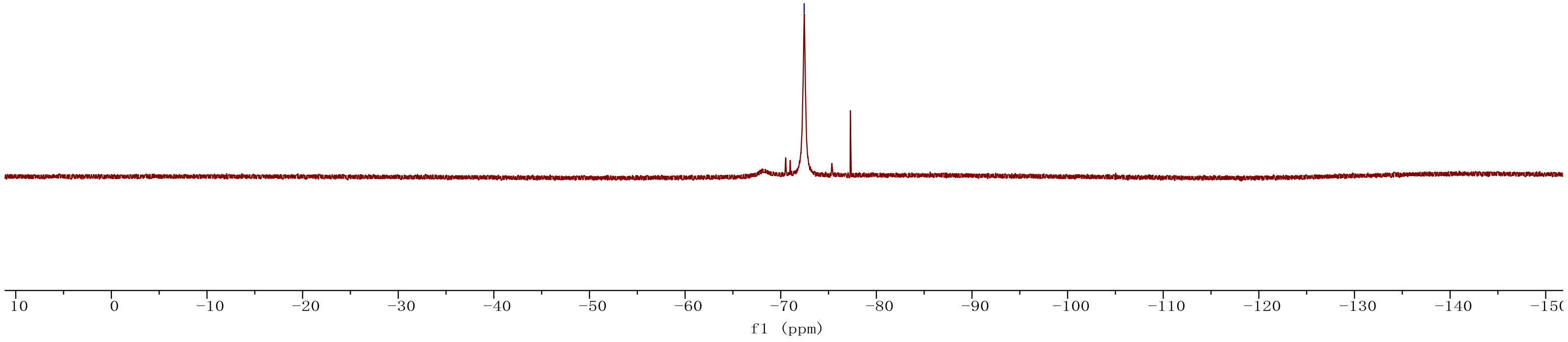

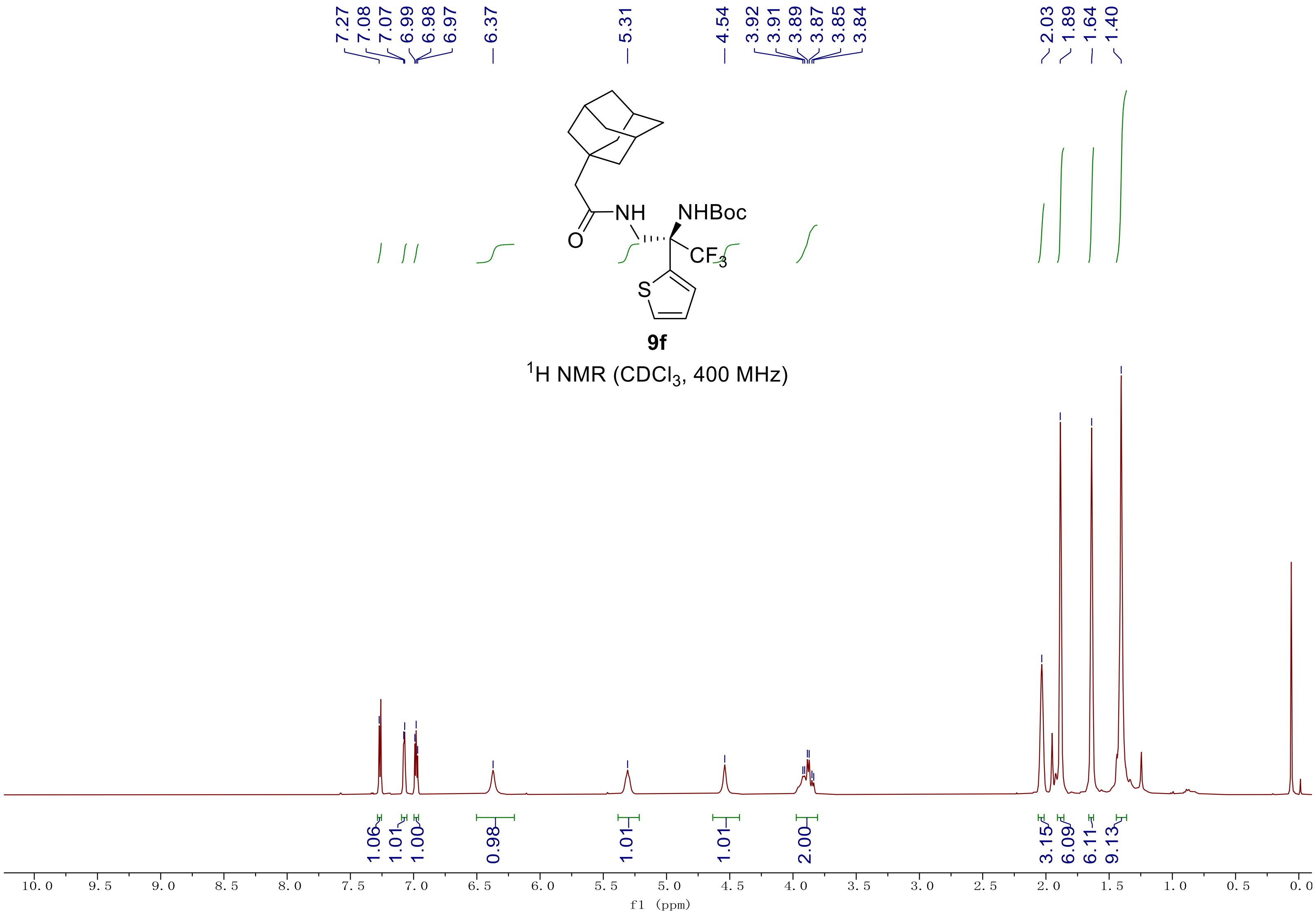

9f

^1H NMR ($CDCl_3$, 400 MHz)

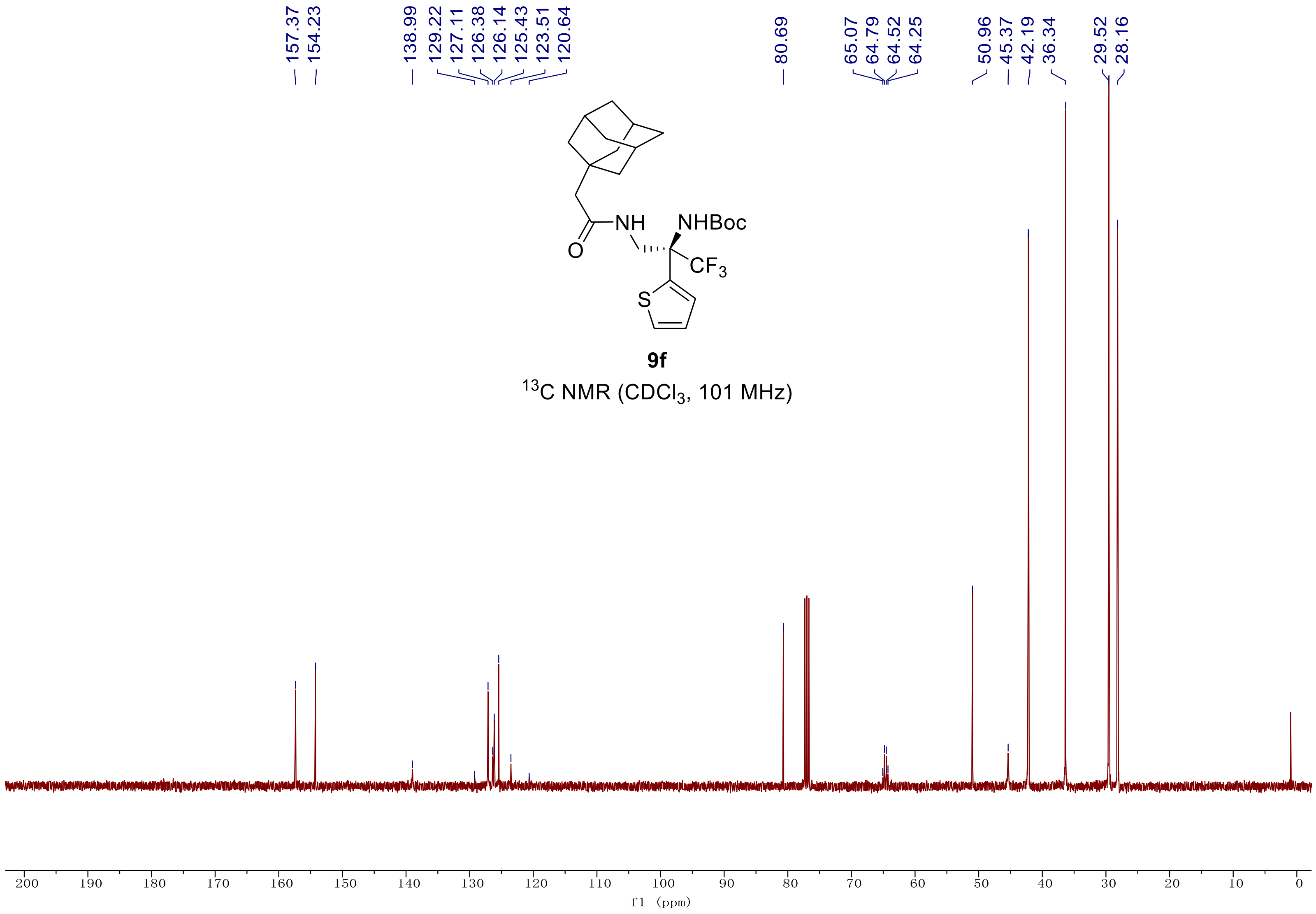

157.37
154.23
138.99
129.22
127.11
126.38
126.14
125.43
123.51
120.64
80.69
65.07
64.79
64.52
64.25
50.96
45.37
42.19
36.34
29.52
28.16
NH
NHBoc
O
CF3
S
9f
13C NMR (CDCl3, 101 MHz)
200 190 180 170 160 150 140 130 120 110 100 90 80 70 60 50 40 30 20 10 0
f1 (ppm)

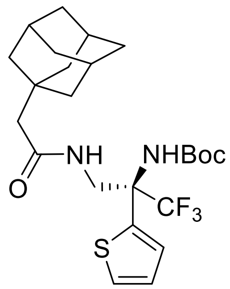

9f

^{19}F NMR ($CDCl_3$, 376 MHz)

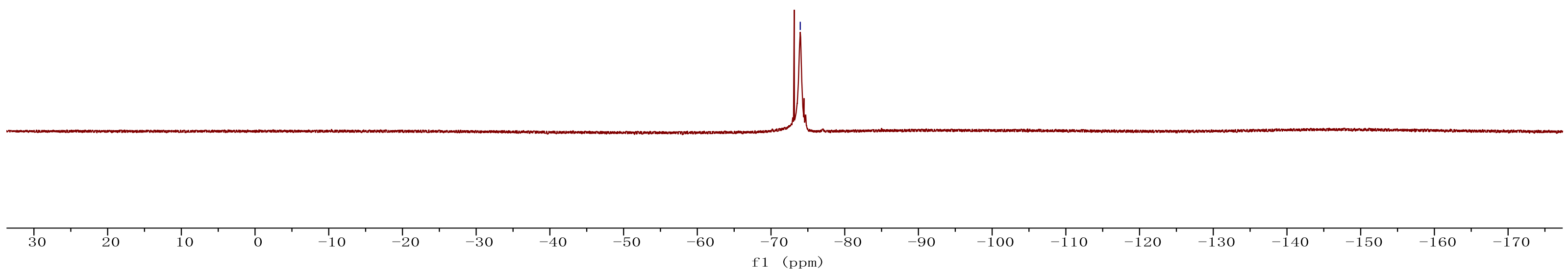

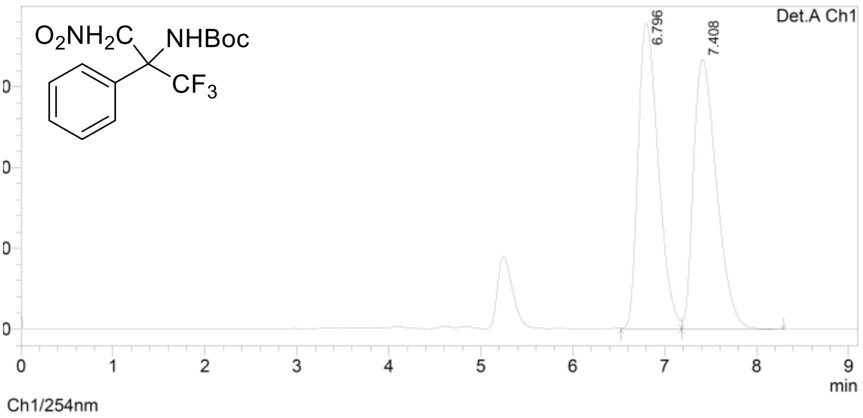

PeakTable

Detector A Ch1 254nm

Peak#	Ret. Time	Area	Height	Area %	Height %
1	6.796	580595	37849	49.878	53.183
2	7.408	583437	33318	50.122	46.817
Total		1164031	71167	100.000	100.000

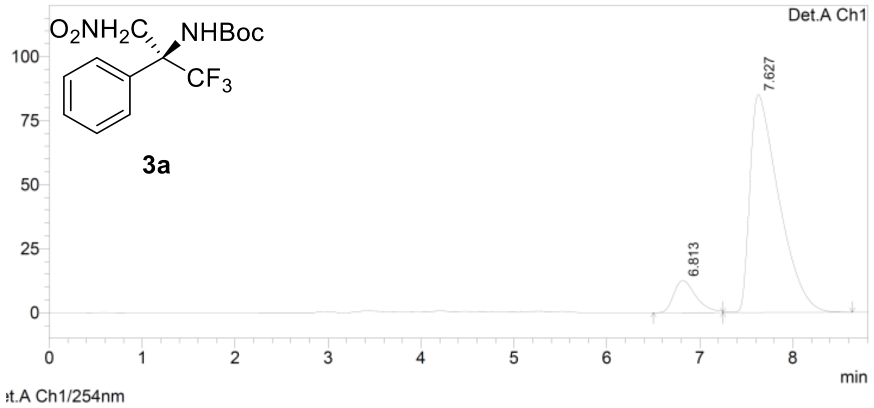

PeakTable

Detector A Ch1 254nm

Peak#	Ret. Time	Area	Height	Area %	Height %
1	6.813	208418	12568	10.150	12.876
2	7.627	1845054	85045	89.850	87.124
Total		2053472	97613	100.000	100.000

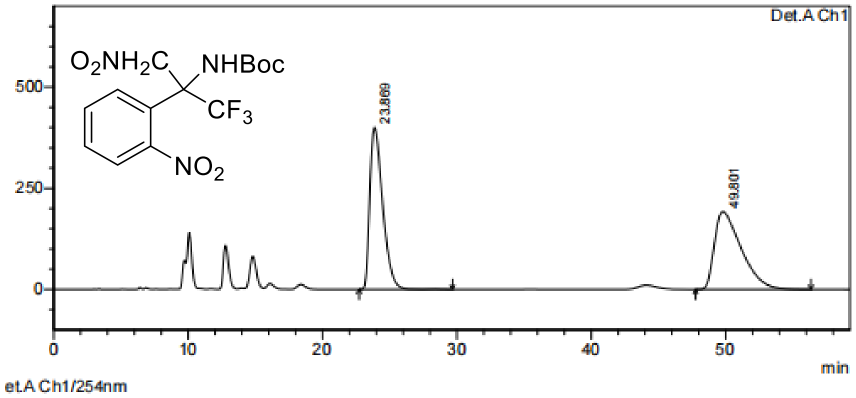

PeakTable

Detector A Ch1 254nm

Peak#	Ret. Time	Area	Height	Area %	Height %
1	23.869	26568788	400384	49.930	67.595
2	49.801	26642912	191941	50.070	32.405
Total		53211700	592326	100.000	100.000

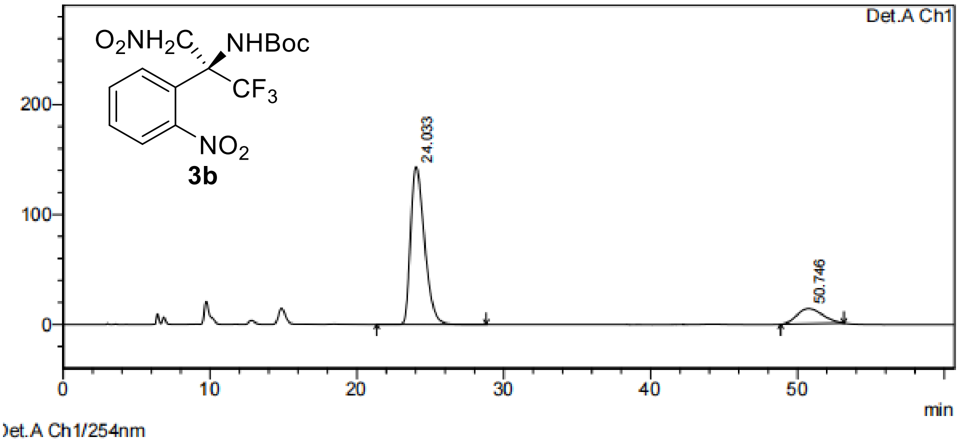

PeakTable

Detector A Ch1 254nm

Peak#	Ret. Time	Area	Height	Area %	Height %
1	24.033	9378513	143526	84.854	91.174
2	50.746	1673964	13893	15.146	8.826
Total		11052477	157419	100.000	100.000

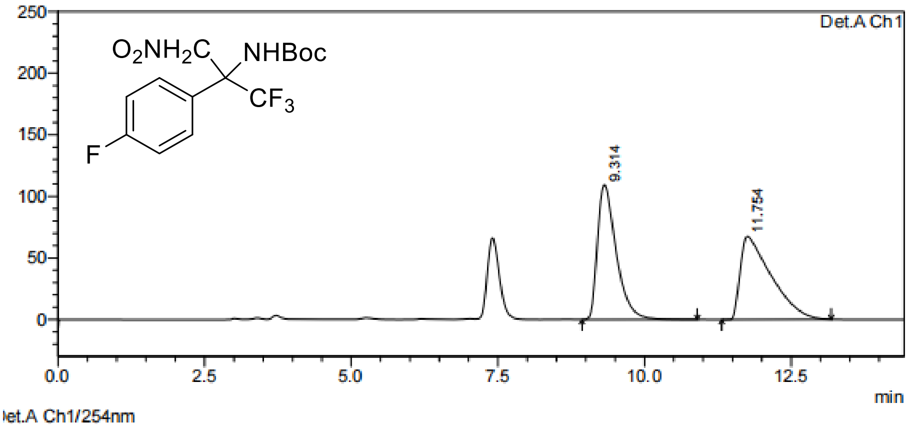

PeakTable

Detector A Ch1 254nm

Peak#	Ret. Time	Area	Height	Area %	Height %
1	9.314	2436556	109213	49.559	61.801
2	11.754	2479910	67505	50.441	38.199
Total		4916466	176718	100.000	100.000

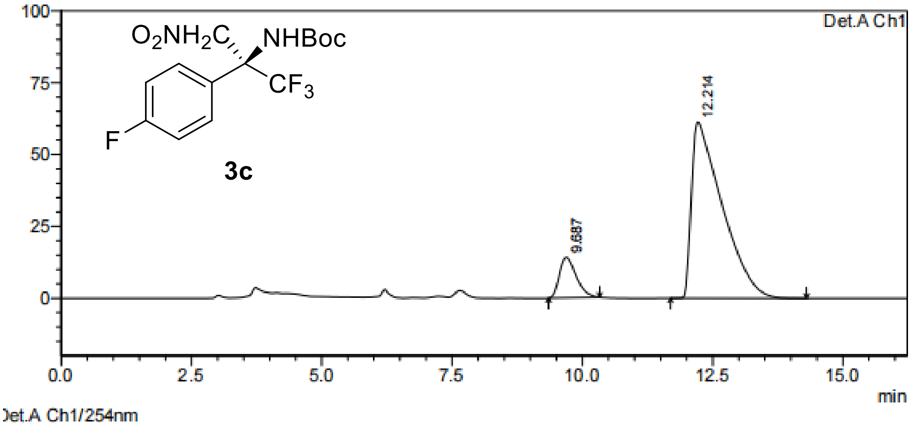

PeakTable

Detector A Ch1 254nm

Peak#	Ret. Time	Area	Height	Area %	Height %
1	9.687	315615	14041	11.202	18.643
2	12.214	2501805	61276	88.798	81.357
Total		2817420	75317	100.000	100.000

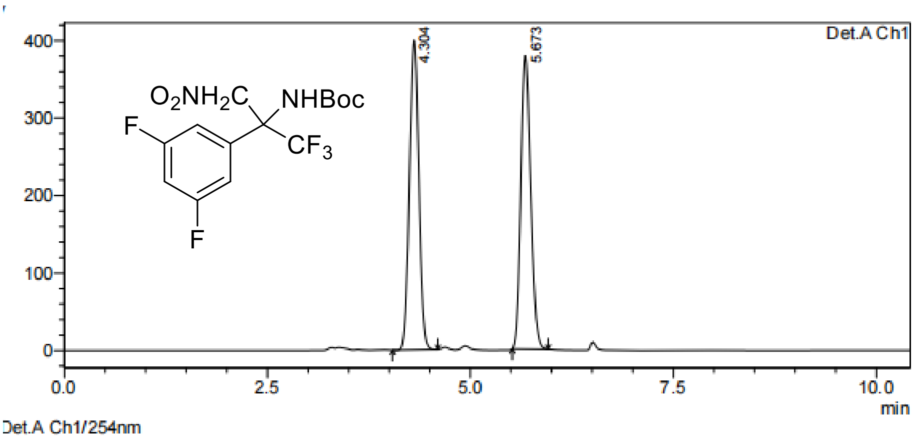

PeakTable

Detector A Ch1 254nm

Peak#	Ret. Time	Area	Height	Area %	Height %
1	4.304	3230025	399637	50.400	51.366
2	5.673	3178746	378387	49.600	48.634
Total		6408771	778024	100.000	100.000

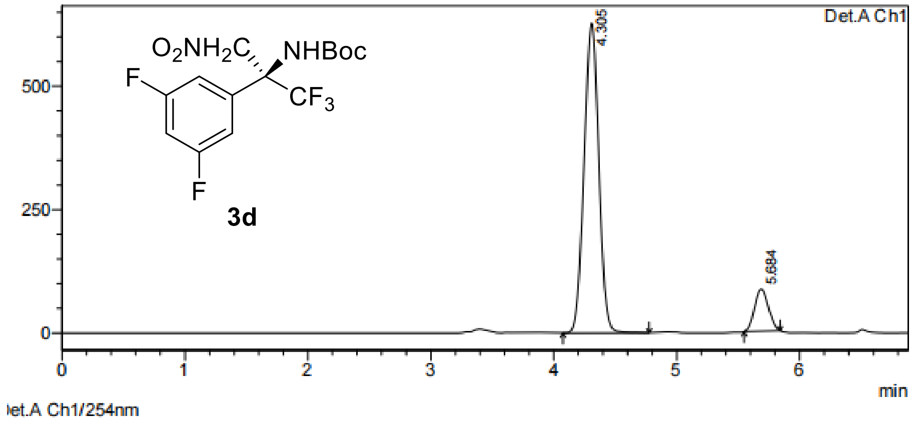

PeakTable

Detector A Ch1 254nm

Peak#	Ret. Time	Area	Height	Area %	Height %
1	4.305	5228249	627841	88.647	88.067
2	5.684	669585	85070	11.353	11.933
Total		5897833	712911	100.000	100.000

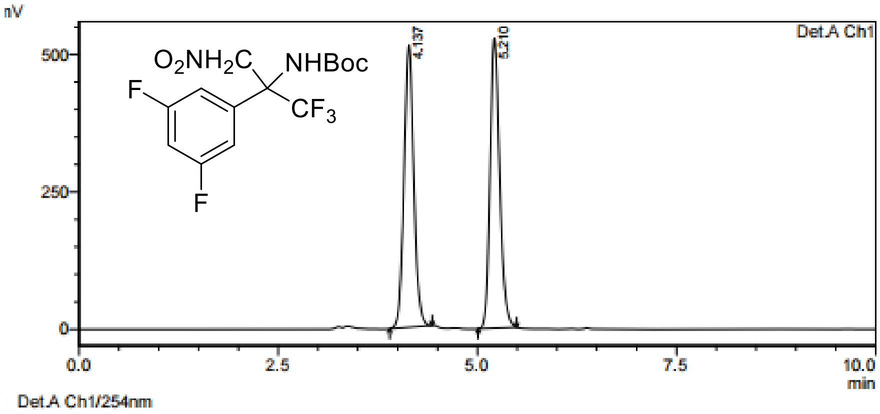

PeakTable

Detector A Ch1 254nm

Peak#	Ret. Time	Area	Height	Area %	Height %
1	4.137	4415787	514041	50.005	49.317
2	5.210	4414832	528283	49.995	50.683
Total		8830619	1042323	100.000	100.000

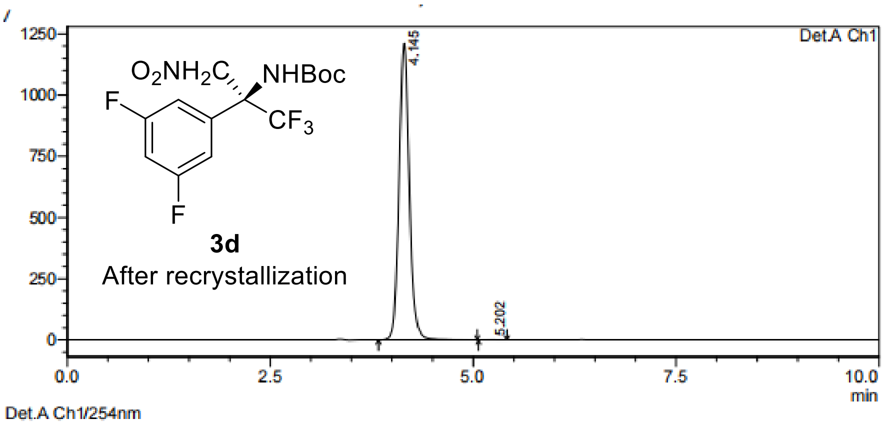

PeakTable

Detector A Ch1 254nm

Peak#	Ret. Time	Area	Height	Area %	Height %
1	4.145	10612657	1212351	99.957	99.948
2	5.202	4607	630	0.043	0.052
Total		10617265	1212981	100.000	100.000

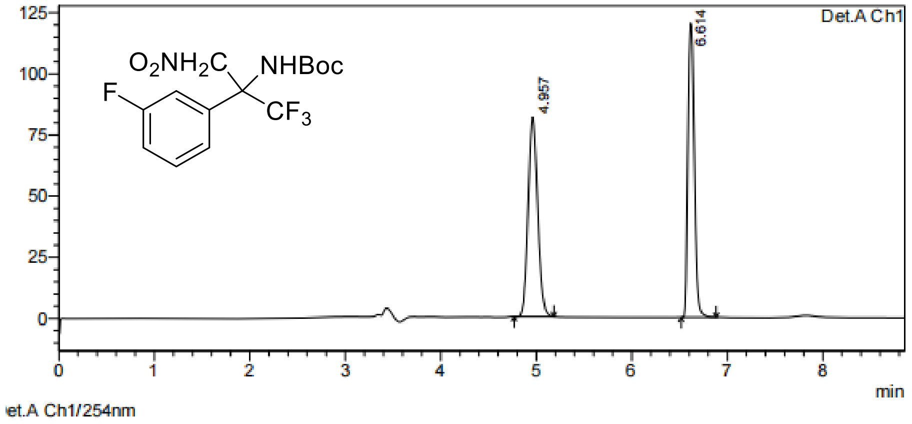

PeakTable

Detector A Ch1 254nm

Peak#	Ret. Time	Area	Height	Area %	Height %
1	4.957	565273	81479	50.362	40.393
2	6.614	557156	120234	49.638	59.607
Total		1122430	201713	100.000	100.000

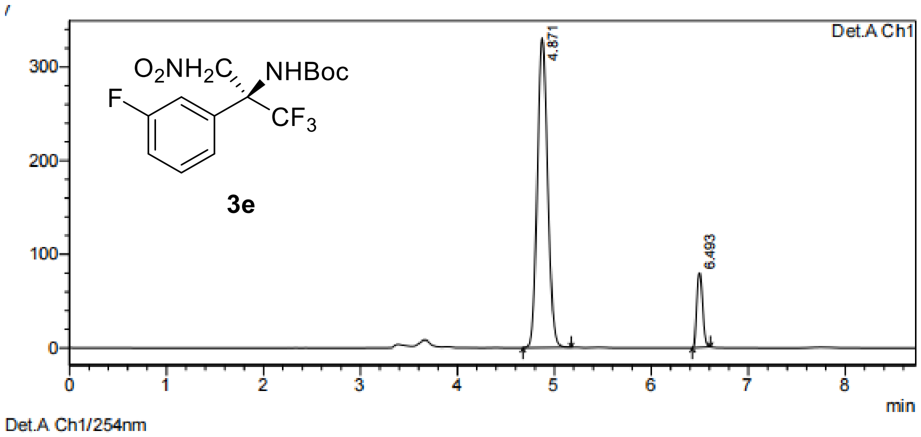

PeakTable

Detector A Ch1 254nm

Peak#	Ret. Time	Area	Height	Area %	Height %
1	4.871	2450817	330481	87.998	80.556
2	6.493	334257	79770	12.002	19.444
Total		2785074	410252	100.000	100.000

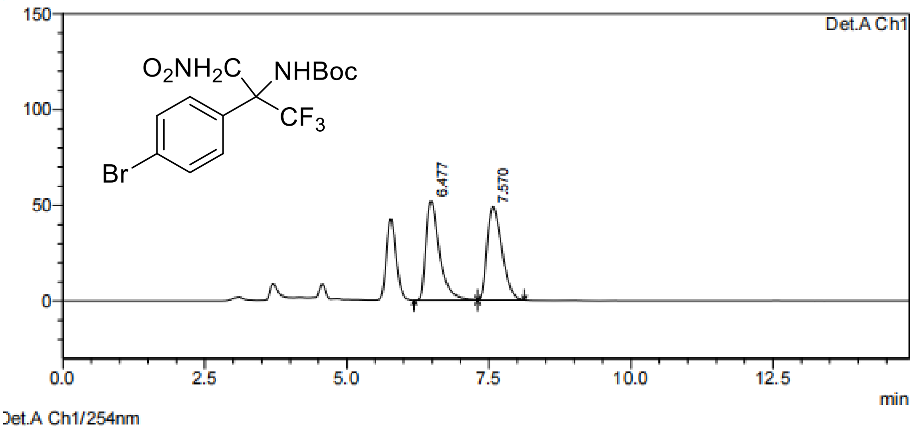

PeakTable

Detector A Ch1 254nm

Peak#	Ret. Time	Area	Height	Area %	Height %
1	6.477	868746	51884	49.551	51.580
2	7.570	884485	48705	50.449	48.420
Total		1753231	100589	100.000	100.000

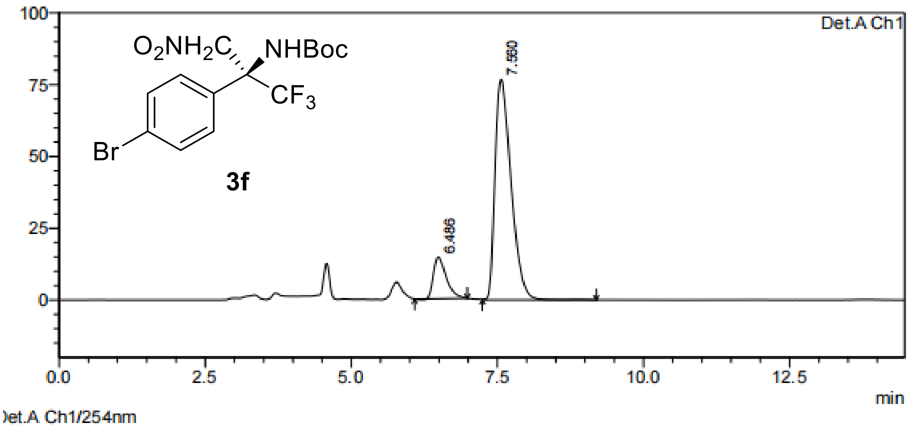

PeakTable

Detector A Ch1 254nm

Peak#	Ret. Time	Area	Height	Area %	Height %
1	6.486	218643	14422	12.979	15.786
2	7.560	1465943	76933	87.021	84.214
Total		1684587	91355	100.000	100.000

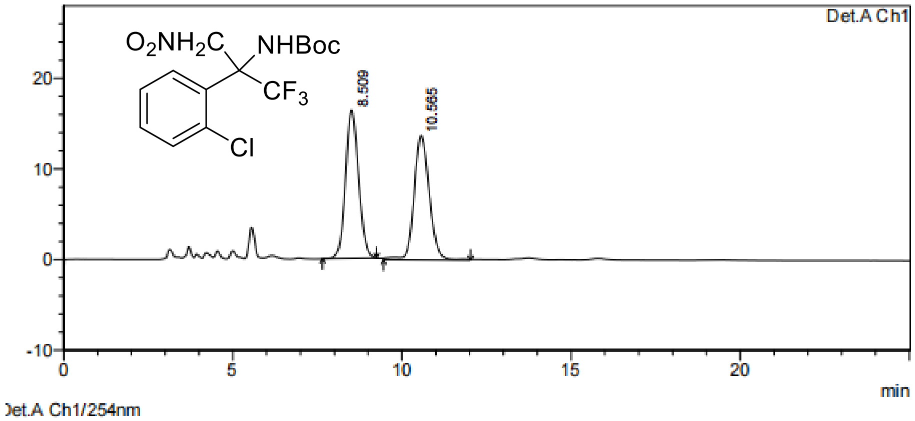

PeakTable

Detector A Ch1 254nm

Peak#	Ret. Time	Area	Height	Area %	Height %
1	8.509	439172	16343	50.306	54.374
2	10.565	433838	13713	49.694	45.626
Total		873010	30056	100.000	100.000

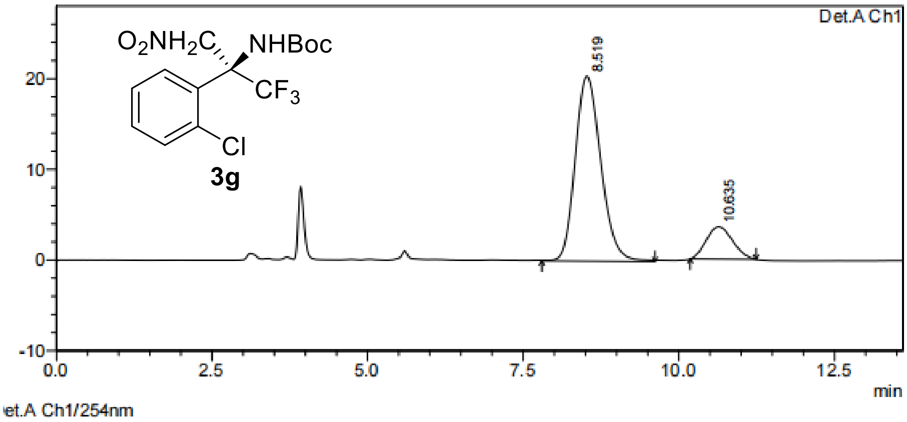

3g

PeakTable

Detector A Ch1 254nm

Peak#	Ret. Time	Area	Height	Area %	Height %
1	8.519	587112	20404	84.876	85.089
2	10.635	104620	3576	15.124	14.911
Total		691732	23979	100.000	100.000

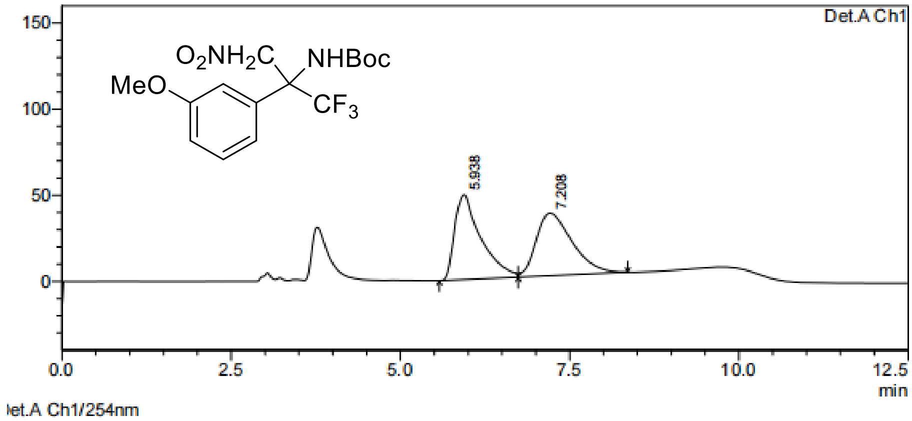

PeakTable

Detector A Ch1 254nm

Peak#	Ret. Time	Area	Height	Area %	Height %
1	5.938	1328348	49281	49.691	57.609
2	7.208	1344873	36263	50.309	42.391
Total		2673221	85544	100.000	100.000

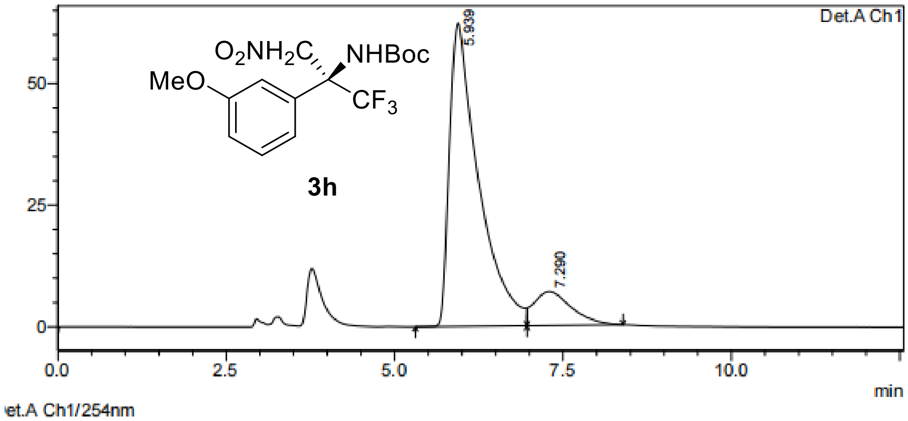

PeakTable

Detector A Ch1 254nm

Peak#	Ret. Time	Area	Height	Area %	Height %
1	5.939	1878807	62313	87.413	89.929
2	7.290	270543	6979	12.587	10.071
Total		2149350	69292	100.000	100.000

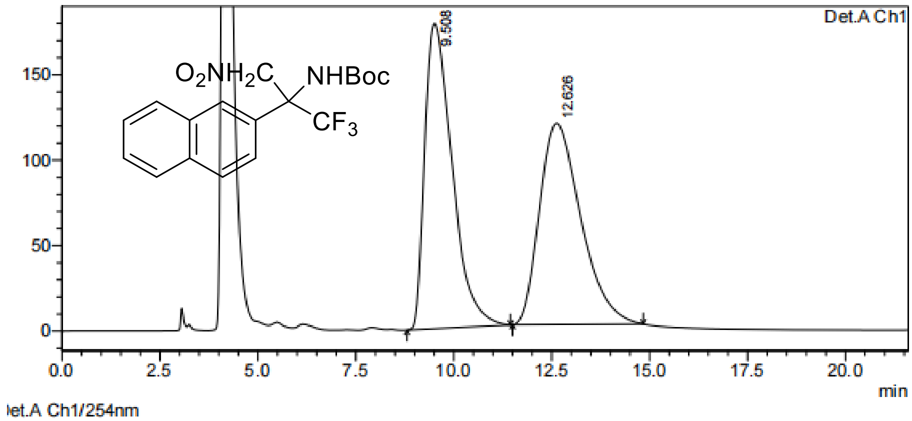

PeakTable

Detector A Ch1 254nm

Peak#	Ret. Time	Area	Height	Area %	Height %
1	9.508	8715784	178528	50.195	60.263
2	12.626	8647932	117720	49.805	39.737
Total		17363716	296248	100.000	100.000

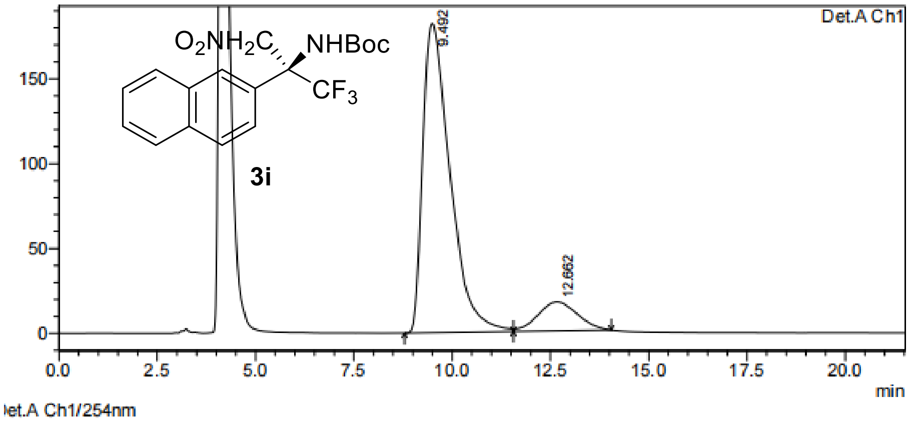

PeakTable

Detector A Ch1 254nm

Peak#	Ret. Time	Area	Height	Area %	Height %
1	9.492	8804170	182231	88.067	91.453
2	12.662	1192932	17031	11.933	8.547
Total		9997102	199263	100.000	100.000

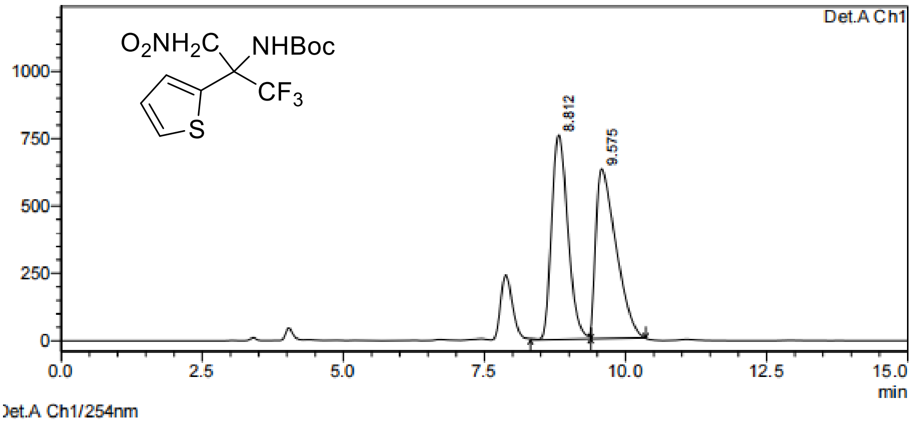

PeakTable

Detector A Ch1 254nm

Peak#	Ret. Time	Area	Height	Area %	Height %
1	8.812	15316336	759695	49.353	54.675
2	9.575	15717851	629787	50.647	45.325
Total		31034187	1389482	100.000	100.000

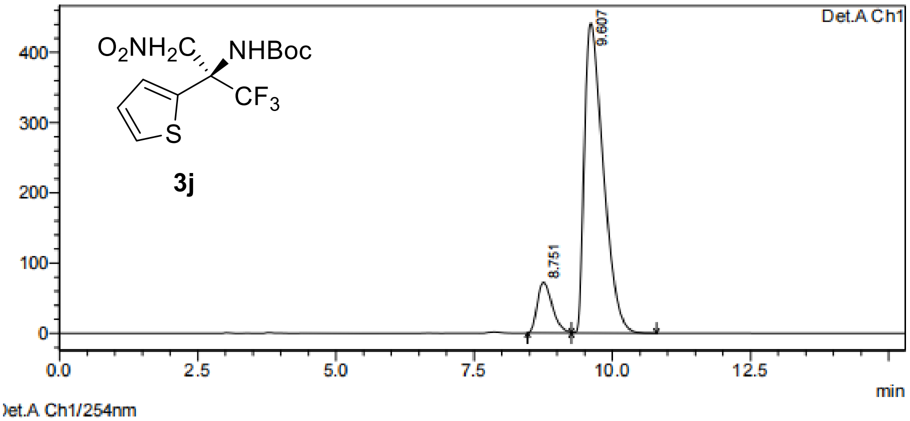

PeakTable

Detector A Ch1 254nm

Peak#	Ret. Time	Area	Height	Area %	Height %
1	8.751	1360800	71757	11.434	13.993
2	9.607	10540901	441045	88.566	86.007
Total		11901700	512802	100.000	100.000

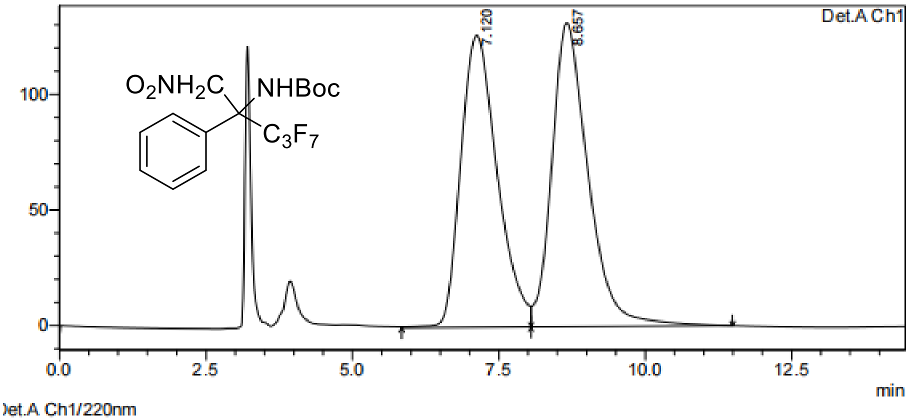

PeakTable

Detector A Ch1 220nm

Peak#	Ret. Time	Area	Height	Area %	Height %
1	7.120	5469729	126237	49.116	49.015
2	8.657	5666698	131311	50.884	50.985
Total		11136427	257547	100.000	100.000

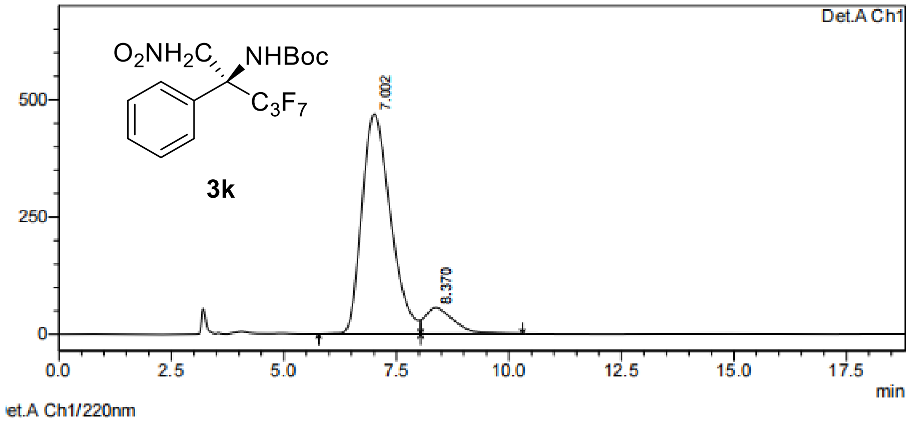

PeakTable

Detector A Ch1 220nm

Peak#	Ret. Time	Area	Height	Area %	Height %
1	7.002	20871485	468727	89.523	89.381
2	8.370	2442557	55690	10.477	10.619
Total		23314042	524417	100.000	100.000

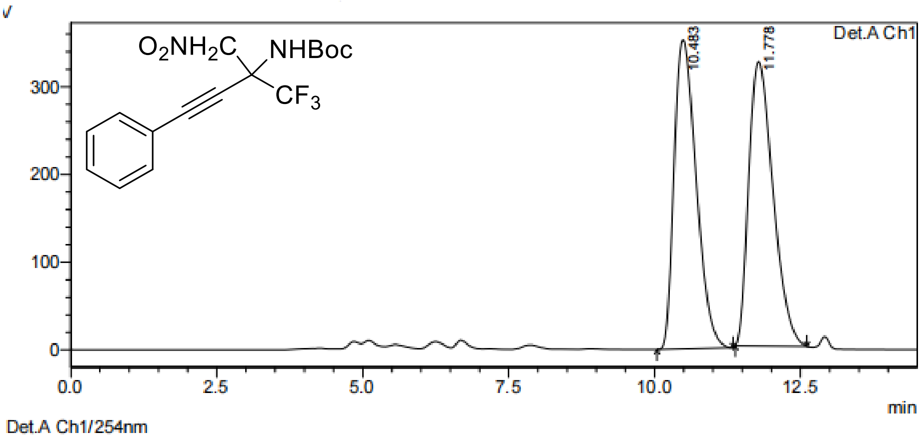

PeakTable

Detector A Ch1 254nm

Peak#	Ret. Time	Area	Height	Area %	Height %
1	10.483	9277190	352173	49.595	52.064
2	11.778	9428746	324252	50.405	47.936
Total		18705936	676426	100.000	100.000

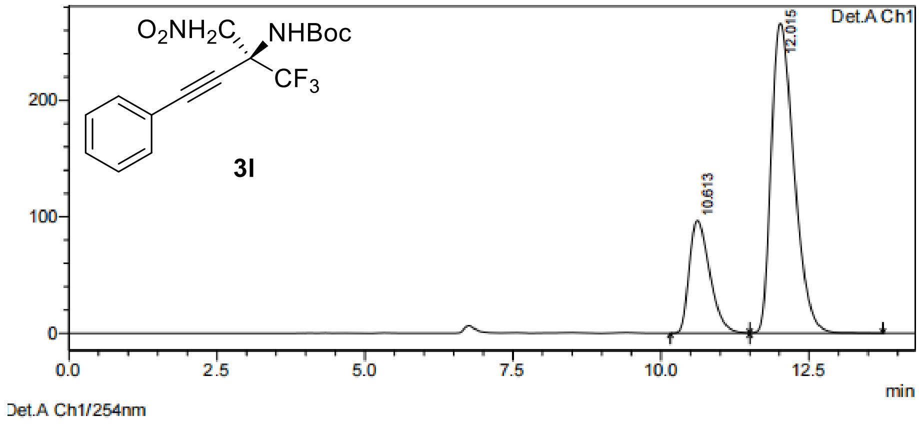

PeakTable

Detector A Ch1 254nm

Peak#	Ret. Time	Area	Height	Area %	Height %
1	10.613	2347998	96839	24.732	26.702
2	12.015	7145831	265829	75.268	73.298
Total		9493829	362669	100.000	100.000

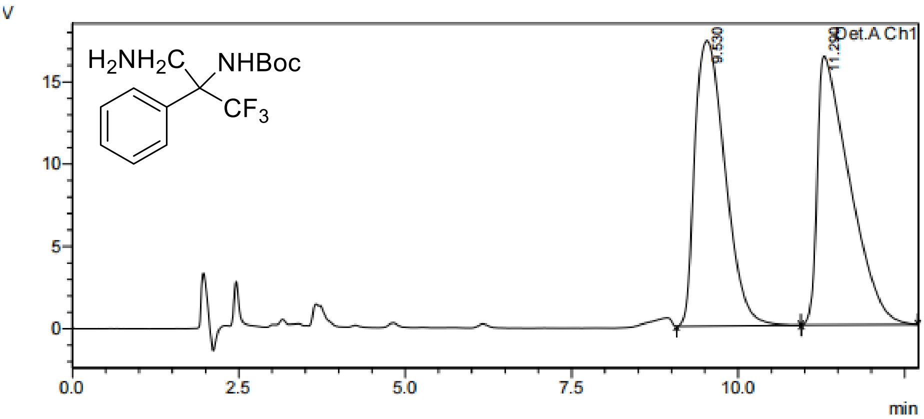

Det.A Ch1/254nm

PeakTable

Detector A Ch1 254nm

Peak#	Ret. Time	Area	Height	Area %	Height %
1	9.530	548301	17333	49.847	51.513
2	11.290	551677	16315	50.153	48.487
Total		1099978	33648	100.000	100.000

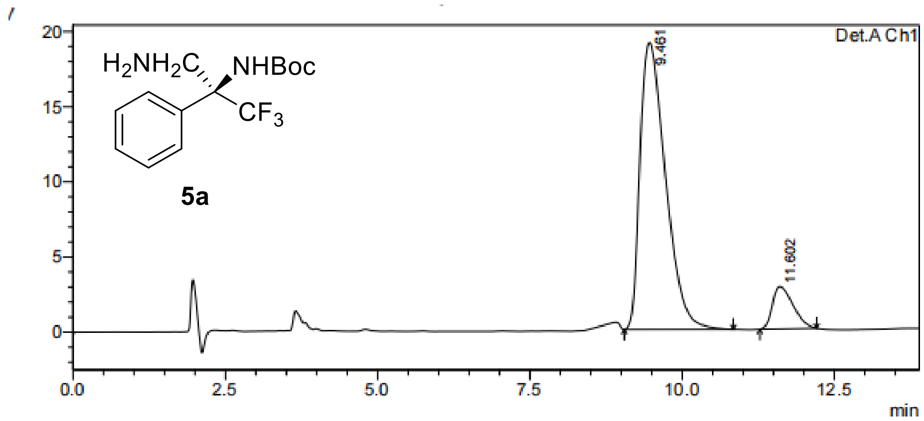

Det.A Ch1/254nm

PeakTable

Detector A Ch1 254nm

Peak#	Ret. Time	Area	Height	Area %	Height %
1	9.461	555588	19084	89.358	87.166
2	11.602	66168	2810	10.642	12.834
Total		621756	21894	100.000	100.000

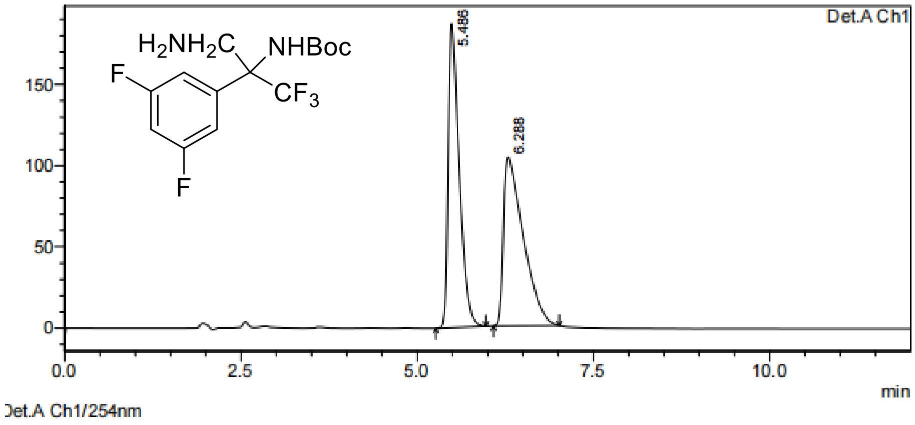

PeakTable

Detector A Ch1 254nm

Peak#	Ret. Time	Area	Height	Area %	Height %
1	5.486	2036005	187258	49.906	64.306
2	6.288	2043648	103942	50.094	35.694
Total		4079653	291200	100.000	100.000

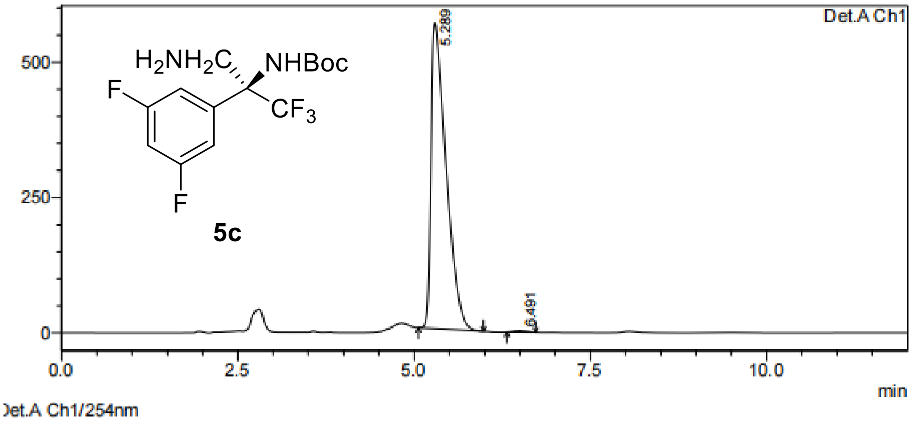

PeakTable

Detector A Ch1 254nm

Peak#	Ret. Time	Area	Height	Area %	Height %
1	5.289	8398280	564159	99.553	99.511
2	6.491	37696	2774	0.447	0.489
Total		8435976	566934	100.000	100.000

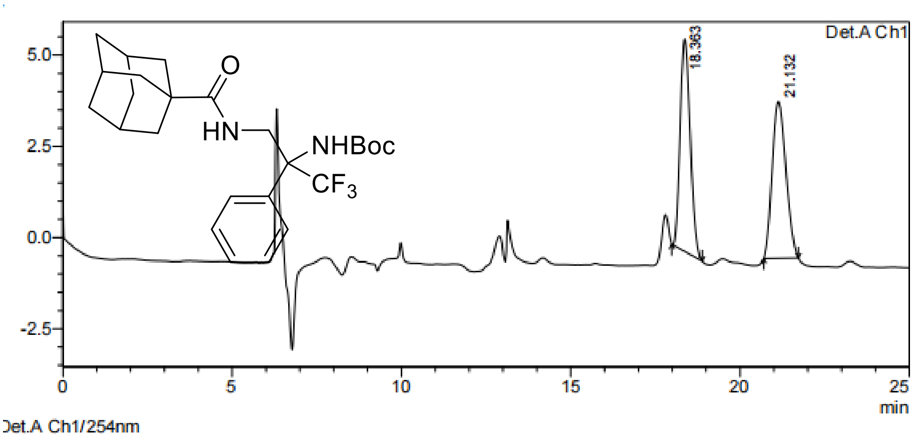

PeakTable

Detector A Ch1 254nm

Peak#	Ret. Time	Area	Height	Area %	Height %
1	18.363	120947	5813	49.929	57.549
2	21.132	121291	4288	50.071	42.451
Total		242238	10101	100.000	100.000

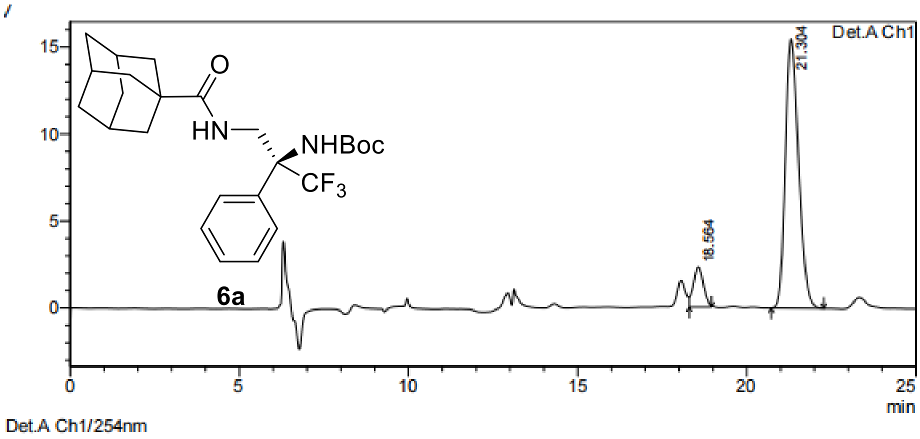

PeakTable

Detector A Ch1 254nm

Peak#	Ret. Time	Area	Height	Area %	Height %
1	18.564	47889	2301	10.215	12.949
2	21.304	420934	15465	89.785	87.051
Total		468823	17766	100.000	100.000

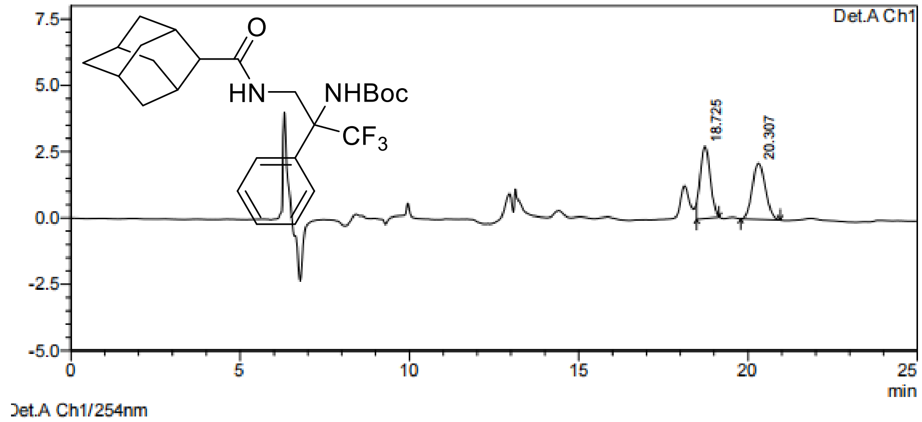

PeakTable

Detector A Ch1 254nm

Peak#	Ret. Time	Area	Height	Area %	Height %
1	18.725	56138	2713	49.691	56.226
2	20.307	56835	2112	50.309	43.774
Total		112972	4825	100.000	100.000

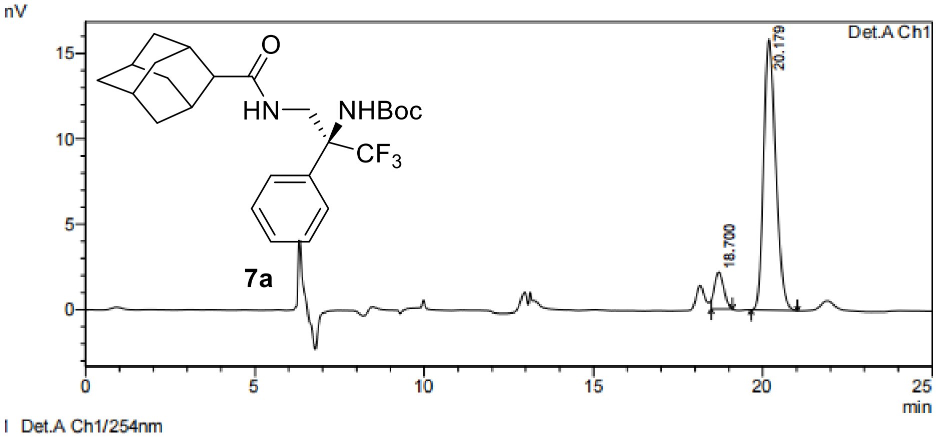

PeakTable

Detector A Ch1 254nm

Peak#	Ret. Time	Area	Height	Area %	Height %
1	18.700	41164	2141	9.449	11.901
2	20.179	394475	15850	90.551	88.099
Total		435640	17991	100.000	100.000

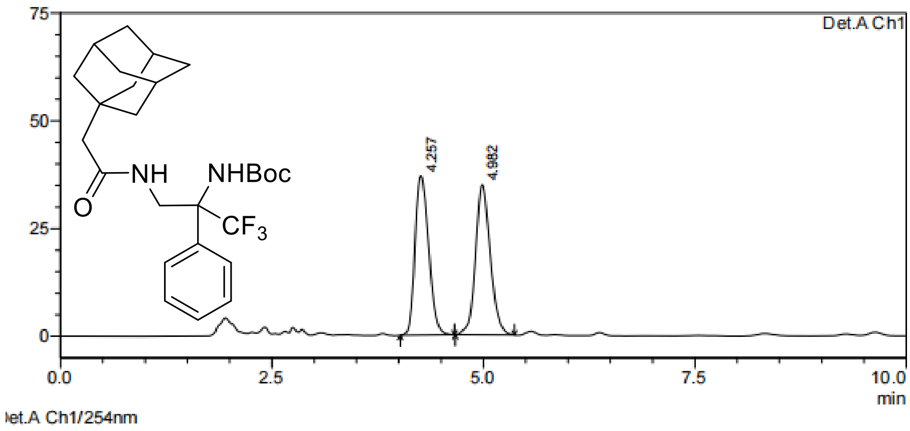

PeakTable

Detector A Ch1 254nm

Peak#	Ret. Time	Area	Height	Area %	Height %
1	4.257	415954	36955	49.590	51.471
2	4.982	422825	34843	50.410	48.529
Total		838779	71799	100.000	100.000

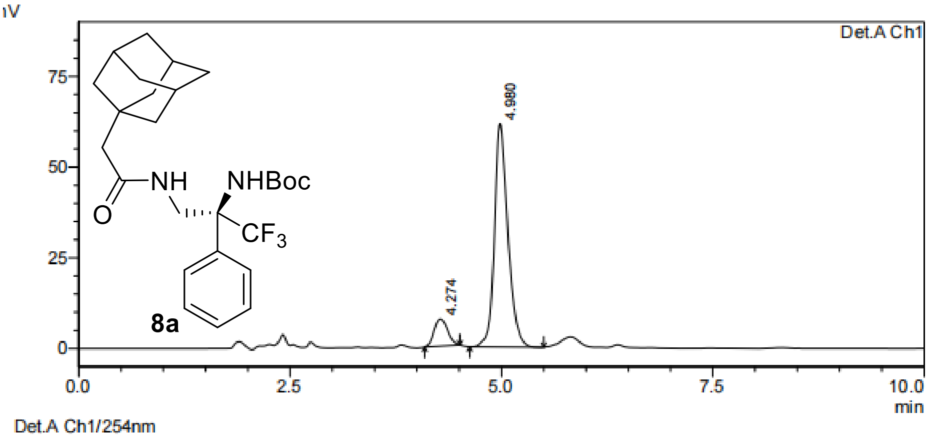

PeakTable

Detector A Ch1 254nm

Peak#	Ret. Time	Area	Height	Area %	Height %
1	4.274	83054	7468	10.912	10.817
2	4.980	678044	61568	89.088	89.183
Total		761098	69036	100.000	100.000

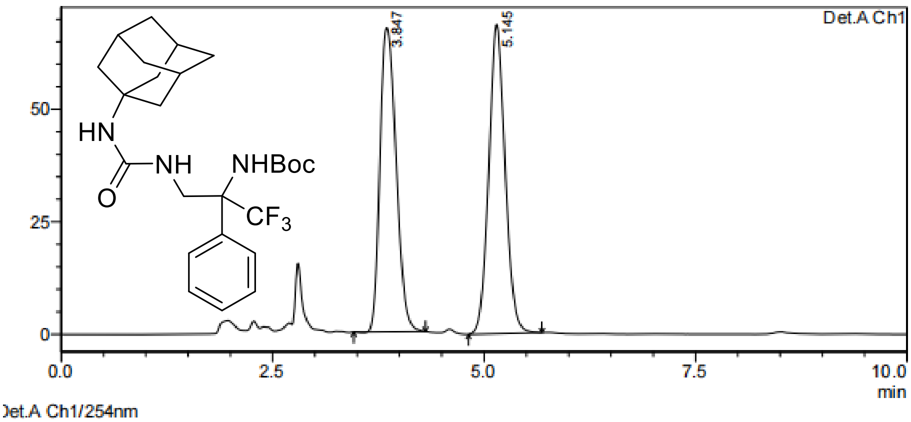

PeakTable

Detector A Ch1 254nm

Peak#	Ret. Time	Area	Height	Area %	Height %
1	3.847	899363	67551	49.305	49.596
2	5.145	924715	68653	50.695	50.404
Total		1824078	136204	100.000	100.000

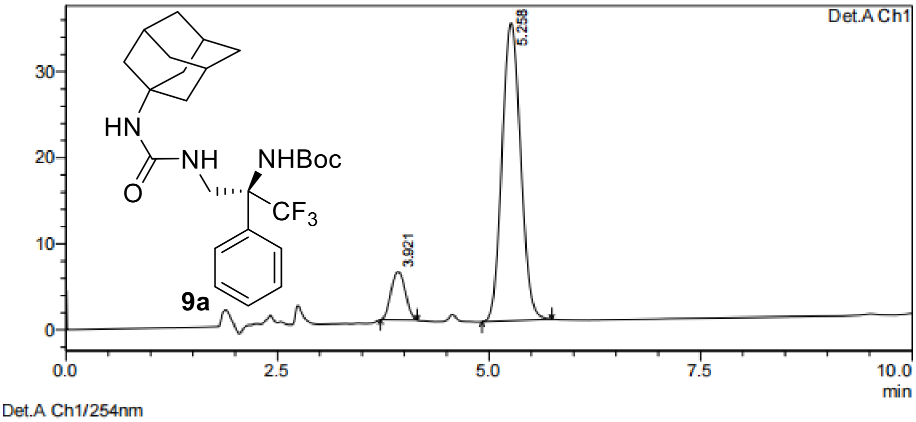

PeakTable

Detector A Ch1 254nm

Peak#	Ret. Time	Area	Height	Area %	Height %
1	3.921	65768	5596	11.031	13.925
2	5.258	530468	34590	88.969	86.075
Total		596237	40186	100.000	100.000

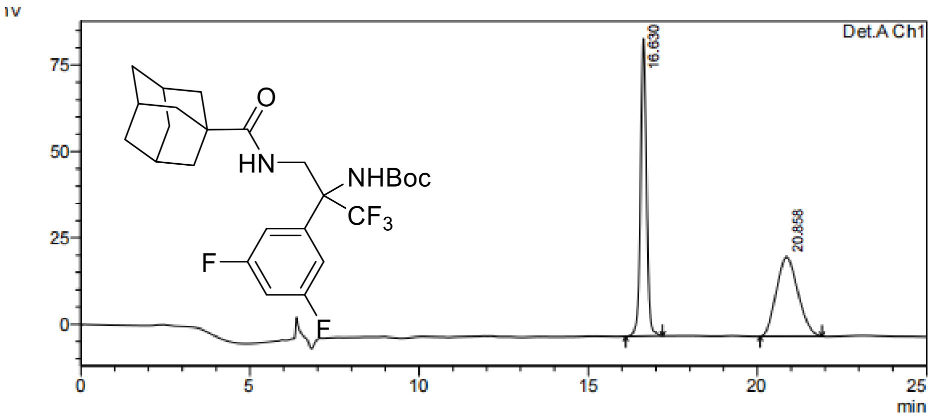

PeakTable

Detector A Ch1 254nm

Peak#	Ret. Time	Area	Height	Area %	Height %
1	16.630	1035176	86124	50.277	78.910
2	20.858	1023751	23018	49.723	21.090
Total		2058927	109142	100.000	100.000

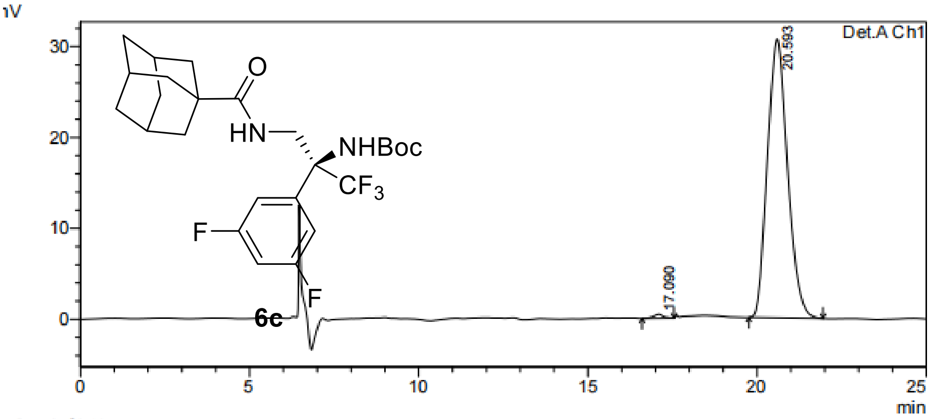

PeakTable

Detector A Ch1 254nm

Peak#	Ret. Time	Area	Height	Area %	Height %
1	17.090	8438	428	0.660	1.378
2	20.593	1270099	30648	99.340	98.622
Total		1278537	31076	100.000	100.000

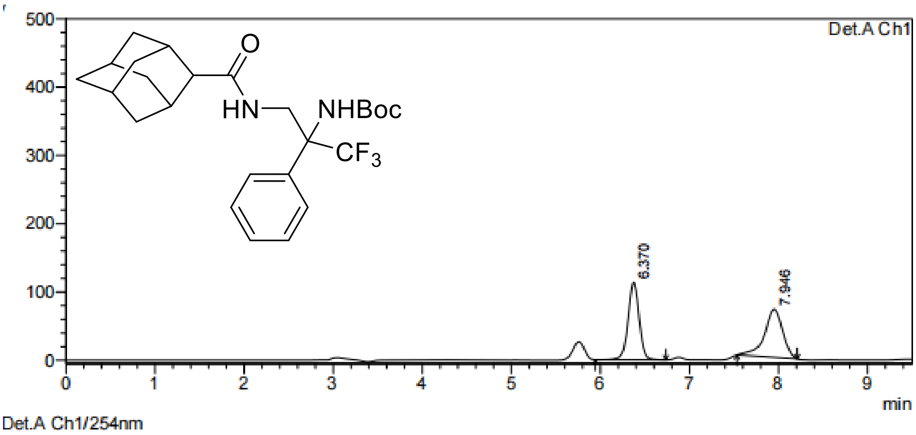

PeakTable

Detector A Ch1 254nm

Peak#	Ret. Time	Area	Height	Area %	Height %
1	6.370	985058	113573	49.853	61.758
2	7.946	990872	70326	50.147	38.242
Total		1975930	183899	100.000	100.000

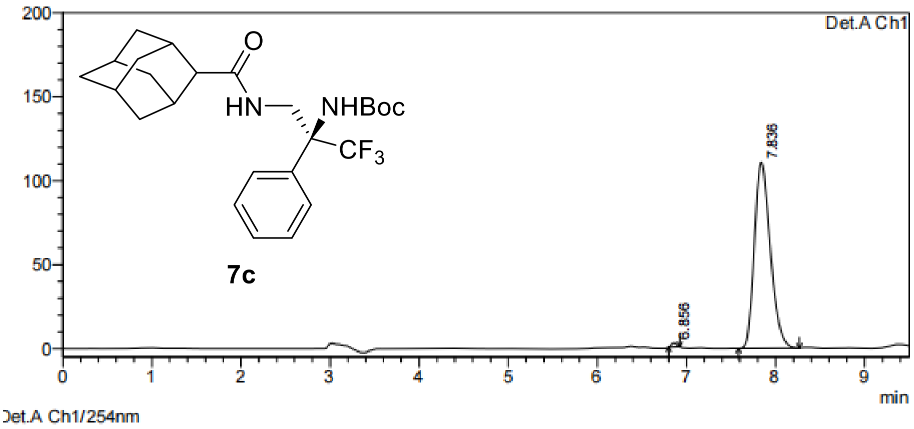

PeakTable

Detector A Ch1 254nm

Peak#	Ret. Time	Area	Height	Area %	Height %
1	6.856	10068	2328	0.727	2.058
2	7.836	1374325	110839	99.273	97.942
Total		1384393	113167	100.000	100.000

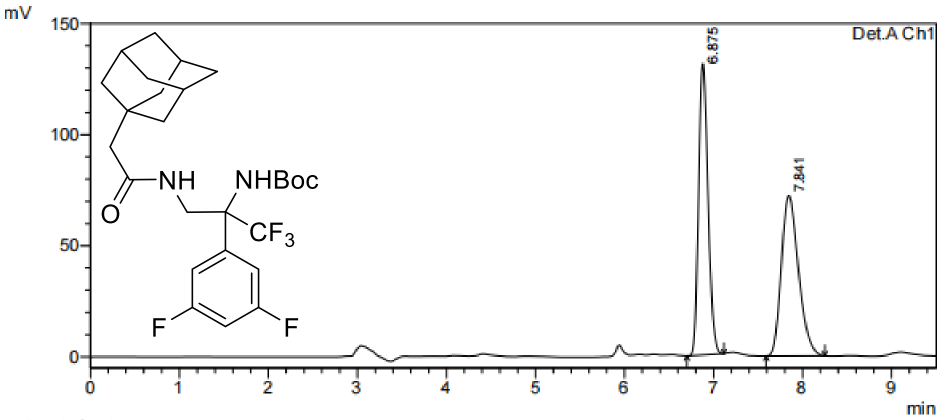

1 Det.A Ch1/254nm

PeakTable

Detector A Ch1 254nm

Peak#	Ret. Time	Area	Height	Area %	Height %
1	6.875	967757	131097	50.034	64.518
2	7.841	966454	72099	49.966	35.482
Total		1934211	203195	100.000	100.000

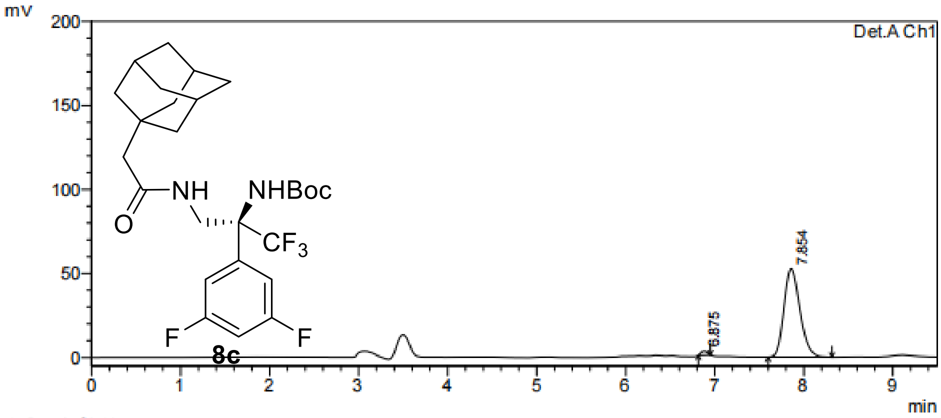

1 Det.A Ch1/254nm

PeakTable

Detector A Ch1 254nm

Peak#	Ret. Time	Area	Height	Area %	Height %
1	6.875	11209	2421	1.684	4.401
2	7.854	654431	52591	98.316	95.599
Total		665640	55012	100.000	100.000

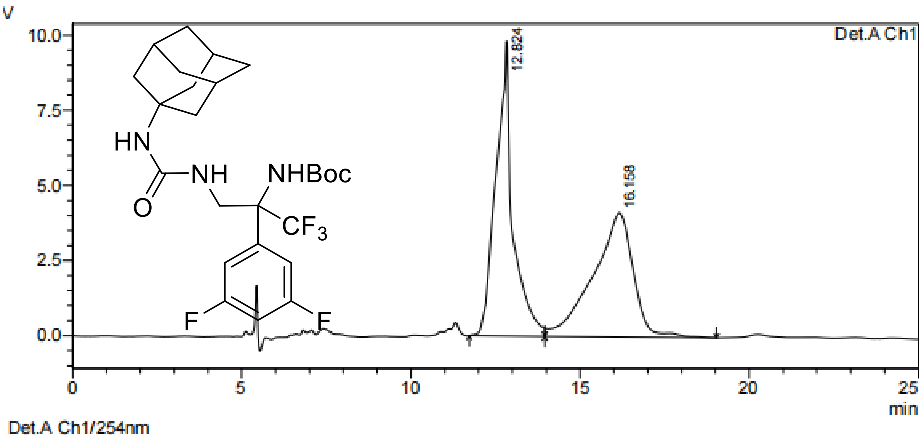

PeakTable

Detector A Ch1 254nm

Peak#	Ret. Time	Area	Height	Area %	Height %
1	12.824	337297	9809	49.798	70.355
2	16.158	340030	4133	50.202	29.645
Total		677327	13942	100.000	100.000

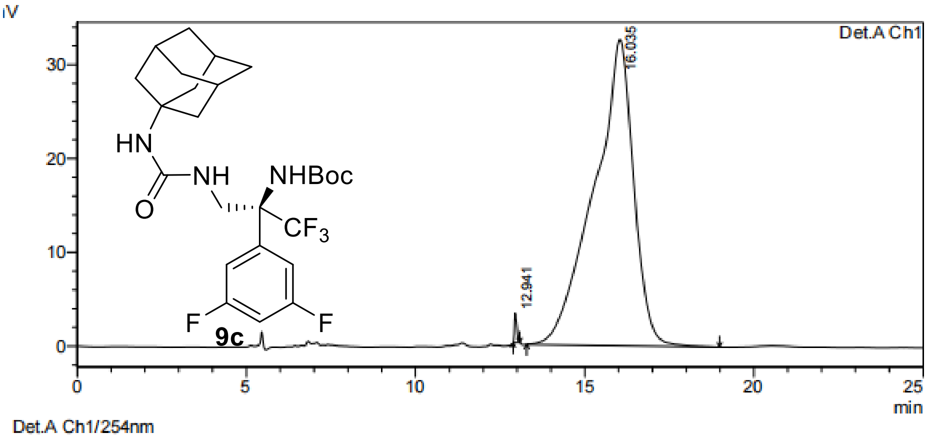

PeakTable

Detector A Ch1 254nm

Peak#	Ret. Time	Area	Height	Area %	Height %
1	12.941	15606	3130	0.585	8.758
2	16.035	2654119	32602	99.415	91.242
Total		2669725	35732	100.000	100.000

Cell viability Assay

Murine melanoma cells B16F10, human hepatocellular carcinoma cells HepG2, and human breast cancer cells MCF-7 were used, and the cells were uniformly seeded in 96-well plates with DMEM medium (with 10% FBS) at a density of 6×10^3 cells/well, and incubated for 24 h at 37 °C with 5% CO_2. Cell culture medium was replaced with fresh medium (100 μl/well) which contained the preset concentrations of compounds (from 3.125 μM to 50 μM). DMSO was set as the solvent control and the same volume medium was used as the experimental blank control. Each group was made in triplicate (n=3). Then incubation was continued for another 48 h at 37 °C, 5% CO_2 conditions. The cell culture medium was carefully aspirated, and 100 μl of fresh cell culture medium (FBS-free) containing 10% CCK-8 reagent was added to each well. Incubation was continued at 37 °C for 2 h, and then the OD value at 490 nm and the reference wavelength 630 nm were measured). The obtained data were subjected to viability calculation according to manufacturer's manual and IC50 calculation was implemented by GraphPad Prism 5 software respectively.

Cell survival rate (50 uM)

	**6a**	**6b**	**6c**	**6d**	**6e**	**6f**	**6g**	**6h**	**6i**	**6j**	**6k**	**6l**
B16F10	54	51	21	32	34	68	10	19	22	24	17	28
HepG2	65	45	22	33	25	58	24	32	20	39	20	26
	**6m**	**6n**	**6o**	**6p**	**6q**	**6r**	**6s**	**6t**	**6u**	**6v**	**6w**	**6x**
B16F10	15	29	29	34	28	20	31	37	32	31	35	25
HepG2	24	35	29	33	29	29	49	56	33	35	39	25

IC50

	8a	7a	7c	7d	7b	6b	7e	8f
Cell B16F10	6.512uM	4.963uM	8.605uM	5.304uM	8.264uM	5.618uM	4.117uM	6.818uM
	8a	7a	7c	6b	7e	6e		
Cell HepG2	8.679uM	8.994uM	8.177uM	7.593uM	12.41uM	7.631uM		

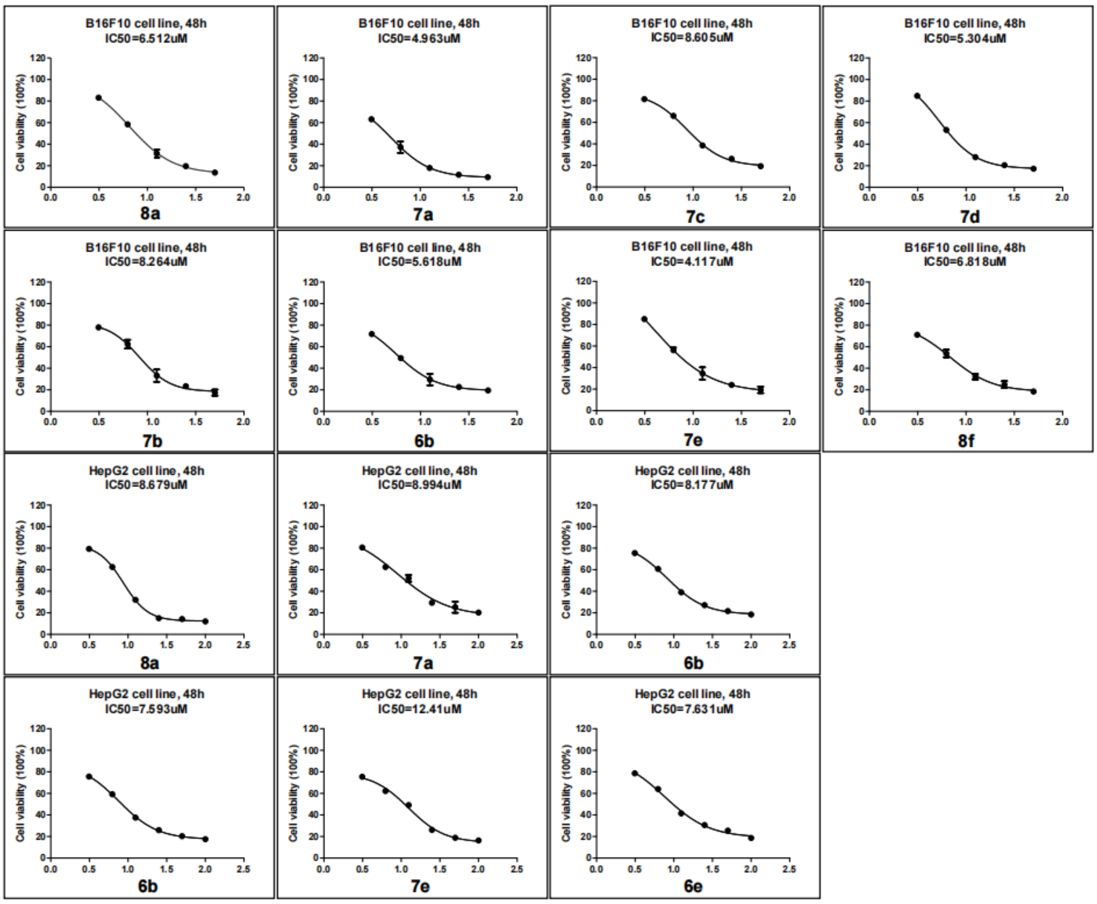

Supplement: Supplementary file 1 [file DataSheet1.PDF]
